# Acknowledgment to the Reviewers of *International Journal of Environmental Research and Public Health* in 2022

**DOI:** 10.3390/ijerph20031979

**Published:** 2023-01-20

**Authors:** IJERPH Editorial Office

**Affiliations:** MDPI AG, St. Alban-Anlage 66, 4052 Basel, Switzerland

High-quality academic publishing is built on rigorous peer review. *International Journal of Environmental Research and Public Health* was able to uphold its high standards for published papers due to the outstanding efforts of our reviewers. Thanks to the efforts of our reviewers in 2022, the median time to first decision was 21 days and the median time to publication was 43 days. Regardless of whether the articles they examined were ultimately published, the editors would like to express their appreciation and thank the following reviewers for the time and dedication that they have shown *IJERPH*:
A. Belen Del RioKhashayar KhoshmaneshA. G. GorshkovKhattak NisarA. HalimKhaula KhatlaniA. M. HodgeKhawaja Shafiq AhmadA. N. M. Mamun-Or-RashidKhayriyyah Mohd HanafiahA. R. M. Saifuddin EkramKhayrutdinov MaratA. S. M. Monjur Al HossainKhoa TranA. SchaafsmaKhurram Shahzad BaigAalap ChokshiKhurshid JahanAaqif AfzaalKhushboo VermaAaron FernandezKhushwant Singh BhullarAaron FoxKhyrina Airin Fariza Abu SamahAaron H. JoslinKi Han SongAaron LawsonKi Joon HeoAaron M. EakmanKi Kwang OhAaron M. WendelboeKi Ok AhnAaron ManzanaresKiadtisak SaenboonruangAaron MayKiara LewisAaron SparksKiat NgAarti SharmaKichol LeeAbad KhanKielan Darcy McAlindenAbayomi OyekaleKieu The Loan TrinhAbazar GhorbaniKifayat UllahAbbas Abbaszadeh ShahriKiffer CardAbbas Ali ChandioKihong KimAbbas Ali GaeiniKihong ParkAbbas F. AlmullaKijin KimAbbas MalekiKikombo NgoyAbbas MardaniKillian KinneyAbbe Maleyki Mhd JalilKim An NguyenAbd Alwali LutfiKim Anh NguyenAbdallah NaserKim BlomqvistAbdalrahman AlblwiKim BloomfieldAbdelhak MerizigKim BuchholtzAbdeljalil MétiouiKim Chun-BaeAbdelkader DerbaliKim DooleyAbdelmotagaly ElgaladKim DorschAbdelouahab MoussaouiKim Dupree JonesAbdelouahed Alla HamouKim Hung LeungAbdelrahim AlqudahKim JoesAbdel-Rahman AklKim LichtveldAbderrahmane AbbouKim MilanoAbderrahmane AyadiKim OuwehandAbderraouf HilaliKim RobienAbdiel Martín-ParkKim RobinsonAbdo Abdullah Ahmed GassarKim SchulteAbdolnasser SadeghkhaniKimberley WangAbdolreza GhodsKimberley WilsonAbdolreza HosseindoustKimberly A. HoffmannAbdolreza JamilianKimberly A. RollingsAbdonas TamošiunasKimberly CoyAbdugheni AblizKimberly E. StephensAbdul Awal Chowdhury MasudKimberly G. FuldaAbdul Aziz Abdul RahmanKimberly HoagwoodAbdul BasitKimberly KaphingstAbdul HaleemKimberly KellyAbdul Kaium MasudKimberly LewisAbdul Majeed NadeemKimberly R. MiddletonAbdul MuhithKimberly Rapp HartsonAbdul Muntaqim NajiKimbo E. YeeAbdul Rahim RidzuanKimmo VirtanevaAbdul Rashid AzizKimon DivarisAbdul RaufKin Kui LaiAbdul Rauf BhattiKin Sun ChanAbdul Razak MariatulqabtiahKin-Fai HoAbdul Salam ShahKing Fai HungAbdul WaheedKinga GawelAbdulaziz AlghamdiKinga Humińska-LisowskaAbdulaziz Hadi Saleh AborujilahKinga KimicAbdulaziz SaddiqueKinga Kostrakiewicz-GierałtAbdulellah Al ThobaityKinga M. SzilágyiAbdulkadir AtalanKinga Nagy-PércsiAbdulkerim KaraaslanKinga Rybak-NiedziółkoAbdullah AkpinarKinga SzabóAbdullah Al MarufKinga SzopińskaAbdullah AleneziKingman StrohlAbdullah Al-MarufKingsley YinAbdullah AlsharefKingston RajiahAbdullah BeyazKinoshita KaoriAbdullah Bin ShamsKira V. VyatkinaAbdullah Hussein AlamoodiKiran KumarAbdullah MuratogluKiran Kumar GanjiAbdullah UsluKiran Kumar SoniAbdullahi AdamuKiran KunduAbdullatif KabanKiran LiversageAbdulrahman AlwarthanKiran NandalikeAbdulrahman BasahelKiri RodgersAbdulrazaq YahayaKiril ManevskiAbdul-Sahib Al-MadhhachiKirill LarionovAbdulvahed Khaledi DarvishanKirill MiroshnikAbdus SaboorKirk MillerAbebe Debele TolcheKirsten CouplandAbel LermaKirsten PerssonAbel López DíezKirsten SpencerAbeynayaka Helayaye Damitha LakmaliKirstie AndersonAbhaya Chandra DasKirti KhuntiAbhijeet SinghKisha PiggottAbhijit BarmanKishor HadkhaleAbhinav JainKishwar AliAbhinav KaushikKíssila RabeloAbhiram KannegantiKi-sun LeeAbhirami K. IyerKitamura MineakiAbhiroop ChowdhuryKiwamu MatsuokaAbhishek ChatterjeeKiwon LeeAbhishek GuptaKiwon LimAbhishek KandwalKiyohiro OshimaAbhishek KumarKiyonori KawasakiAbhishek MishraKiyoshi TakagiAbhishek ShankarKjell ØvergårdAbid Ali KhanKjerstin StigmarAbid BhatKlara CermakovaAbigail L. WhitehouseKlára KovácsAbigail MulcahyKlara Smith-EtxeberriaAbigail ReiterKlara SzitaAbijeet MehtaKlas-Göran SahlenAbiodun Kolawole OyetunjiKlaudia GurkowaAbiodun Morakinyo AdeolaKlaudija ViškovićAbiodun Olusola OmotayoKlaus GeiselhartAbiola S. LawalKlaus H. HoffmannAbir NagataKlaus MoserAbolfazl MollaloKlaus NeefAbraão Almeida SantosKlaus SchäferAbraham BernárdezKlaus SchneiderAbraham Boby RibyKlavdia Oleschko LutkovaAbraham DibKlazien Matter-WalstraAbraham López VivancosKleber Campos Miranda-FilhoAbraham Méndez-AlboresKleftodimos AlexandrosAbraham P. BuunkKleisiaris F. ChristosAbraham RudnickKlejda HarasaniAbraham TsitlakidisKlemen BohincAbraham Wall MedranoKleoniki Natalia PetrouAbram MusinguziKleopatra LeontidouAbrar YousufKleopatra NikolopoulouAbu Al Nasr SobaihKloos HeidiAbu KibriaKnut Anton MorkAbu Yousuf Md AbdullahKnut Asbjørn Alexander ØymarAbul Kalam AzadKnut Ragnvald SkulbergAbul RahmanKobayashi DaisukeAcga ChengKobus HerbstAchilleas KostoulasKodai KitagawaAchim BurrerKodjovi D. MlagaAchim DrebsKofi AgyekumAchintyamugdha SharmaKoh MizunoAchmad NurmandiKohei MakitaAchmad Pratama RifaiKohei MaruyaAchmad RizalKohei OkuyamaAda M. Pérez-PicoKoichi HashimotoAda Margarida Correia Nunes Da RochaKoichi NaitoAdam AnczykKoichiro TakagiAdam BujnowskiKoji UchiyamaAdam ChoryńskiKoji YamatsuAdam CudowskiKok Ben TohAdam CzarneckiKok Hian TanAdam CzudecKok Sin WoonAdam D. BramowethKomali KantamaneniAdam Edward LangKomali YennetiAdam GawrylukKon Shik KimAdam GolabKonarski WojciechAdam GórnyKongkea PhanAdam KalksteinKongkiti PhusavatAdam KernKongpan LiAdam KlimowiczKongtae RaAdam KonczKongzheng LiangAdam LewisKonosuke NakajiAdam ŁukowskiKonrad CzapiewskiAdam M. CroomKonrad FurmańczykAdam McDonnellKonrad FutymaAdam MetelskiKonrad HuppiAdam NadolnyKonrad Leszek MendralaAdam OckelfordKonrad StępieńAdam PacławskiKonrad Wolfgang KallusAdam PawlewiczKonstadinos AbeliotisAdam PyzikKonstantin B. IlijevićAdam R. SzromekKonstantin BrazovskiiAdam ReichKonstantin Johannes ScholzAdam SchifferKonstantin MarinovAdam SenetraKonstantin P. GaikovichAdam SukiennikKonstantin SchwarzÁdám SzalaiKonstantin SharovAdam T. CarpenterKonstantina PoulianitiAdam WichniakKonstantinia AlmpaniAdam WiśniewskiKonstantinos A. BanitsasAdam ZającKonstantinos A. GatzoulisAdcock TreyKonstantinos A. LioliosAddisalem Workie DemsashKonstantinos ChristopoulosAddolorata CorradoKonstantinos DritsasAdeb Qaid Al-AmeriKonstantinos G. KyriakoulisAdebayo AdeyinkaKonstantinos KafetsiosAdebayo O. SojobiKonstantinos KontoangelosAdebowale OgunjirinKonstantinos LalenisAdeel ArshadKonstantinos NinikasAdegboyega SaparaKonstantinos PapadimitriouAdel A. MohamedKonstantinos PlakasAdel Al-GheethiKonstantinos ProkopidisAdel JuaidiKonstantinos SalonitisAdel S. AlaglKonstantinos SteirosAdela AndryskovaKonstantinos SyriopoulosAdela BadauKonstantinos TasoulisAdela CiobanuKonstantinos TepetesAdela HobleKonstantinos TosiosAdela KrivohlavekKonstantinos TourlakopoulosAdela Laura PopaKonstantinos TsiakasAdela Magdalena CiobanuKonstantinos TsioufisAdelaida AvinoKonstantinos VamvakarisAdelaide MaderaKonstantinos ZafeirisAdele HoughtonKöpke SaschaAdele SaterianoKorah Pushpamangalam KuruvillaAdeleye Ayoade AdeniranKoral JeromeAdeline DivouxKorede Kafayat YusufAdelle McArdleKornanong YuenyongchaiwatAdem ÖcalKornelia BatkoAdesh SainiKornelia Kuźnik-TrochaAdewale GiwaKornelia ZarębaAdiele DubeKosha J. MehtaAdil AbdullahKosuke MiyaiAdil AsgharKota KodamaAdil MansoorKotaro MatsumotoAdilia GasparKotel DadonAdilson MarquesKotryna FraserAdina BrahaKoulla ParpaAdina Carmen IlieKoumantakis GeorgeAdina CristeKourosh SheibaniAdina FrumKousalya PrabaharAdina Magdalena MusucKousho WakeAdina SchneeweisKoutlianos NikolaosAdina ThomaidisKow-Tong ChenAditi ChopraKoyin ChangAdjani A. PeraltaKozeta MilikuAdli AliKozo TannoAdnan AkhterKraichat TantrakarnapaAdnan AynaKraiwuth KallawichaAdnan KhanKrasimira Borislavova IvanovaAdnan KisaKrassimir MarkovAdnan Raza AltafKrešimir DolićAdnan Ul HaqueKresimir PavlinaAdolfo Cristóbal CampoamorKrim K. LaceyAdolfo Di FioreKrishan Chanaka WickramasingheAdolfo RomeroKrishan SoodAdoración GraciaKrishanu RoyAdrian BoiceanKrishna Mohan SurapaneniAdrian BurlacuKrishna P. HortAdrian Catalin PuitelKrishna RokaAdrian CurtoKrishnaji R. KulkarniAdrián CurtoKrishnan GanapathyAdrian G. ValverdeKrisna PiravejAdrián González-CastilloKristen E. McLeanAdrián González-MarrónKristen H. WalterAdrian GrozeaKristen LwinAdrian HarrisonKristen N. SobbaAdrian HatosKristen PammerAdrian J. GreenKristen Pogreba-BrownAdrian Kah Heng ChiowKristen R. BreitAdrian LabarKristen WyckoffAdrian M. HallKristi HuikAdrián Mateo-OrcajadaKristian RotaruAdrian MidgleyKristiina Korjonen-KuusipuroAdrian NagyKristijan TomljanovicAdrian OpreKristin A. DrexlerAdrian RosuKristin HaglundAdrian StancuKristin HoranAdrian T. RadulescuKristin JankeAdrian TanKristin L. JonvikAdrián Varela SanzKristin L. SainaniAdrian Vasile MuresanKristin M. NiemanAdrian WlodarczakKristina AndrijauskaiteAdrian Zaragoza-BastidaKristina Batelja LodetaAdriana Aurelia ChișKristina BazienėAdriana BiedrońKristina BertlAdriana Burlea-SchiopoiuKristina BerzinaAdriana Caldo-SilvaKristina BučarAdriana CoelhoKristina DissingAdriana DeheleanKristina KatsarosAdriana DerejkoKristina L. Allen-BradyAdriana Díez-GómezKristina LindvallAdriana DimaKristina MattssonAdriana FranzeseKristina MatuzeviciuteAdriana G. Nevarez-FloresKristina MeyerAdriana GrigorașKristina Pogrmic-MajkicAdriana HernándezKristina TurnerAdriana KaplánováKristine KrajnakAdriana LisKristine M. KrajnakAdriana Matutino KahnKristine RuppertAdriana MikaKristine SorensenAdriana MontealegreKristy BuccieriAdriana Patricia Corredor-FigueroaKristy HowellsAdriana RadosavacKristyn KamkeAdriana ReveiuKrisztián KisAdriana TeodorescuKrisztina DajnokiAdriana Teresa Silva SantosKrisztina MarthaAdriane Elisabete Costa AntunesKrisztina MonoryAdrianna BanioKrit PhankitnirundornAdrianna GardzińskaKritika KothariAdriano BressaneKrsto LipovacAdriano De LeveranoKrystal LynchAdriano FiorucciKrystelle ShaughnessyAdriano FriganovicKrystian HeffnerAdriano PiattelliKrystian ObolewskiAdrie VerhoevenKrystina ChoinskiAdrienne AlayliKrystle MartinAdrienne ClementKrystyna KurowskaAdrienne L. CohenKrystyna Mazurek-ŁopacinskaAdrienne SalmKrystyna OlczykAdrienne Vanessa LevayKrystyna PawlasAe HaKrystyna PoznańskaAely ParkKrystyna RejmanAfia Zubair RajaKrystyna Szymandera-BuszkaAfona ChernetKrzesimir CiuraÁfrica Peral-SuárezKrzysztof BiernatAfsane RiaziKrzysztof BoryczkoAfshan BeyKrzysztof BrzezińskiAfsin SahinKrzysztof DowgierdAftab Aslam Parwaz KhanKrzysztof DrozdzolAfzal MisraniKrzysztof Durkalec-MichalskiAfzal ShaikKrzysztof FilipiakAgata BalińskaKrzysztof GedigaAgata Białecka-DębekKrzysztof GomułkaAgata BonenbergKrzysztof GorynskiAgata Chudzicka-CzupałaKrzysztof J. CzarnockiAgata GabryelskaKrzysztof J. RudzińskiAgata GajdekKrzysztof JamroziakAgata GawlakKrzysztof JurekAgata GaździńskaKrzysztof KaczmarekAgata Jabłońska-TrypućKrzysztof KaneckiAgata JanowskaKrzysztof KassolikAgata KobyłkaKrzysztof KilAgata KociaKrzysztof KowalAgata M. NiewczasKrzysztof KrukowskiAgata MałysiakKrzysztof KrystaAgata MatarazzoKrzysztof KudAgata Mesjasz-LechKrzysztof KusAgata MichalakKrzysztof KusmierekAgata NawrockaKrzysztof LetachowiczAgata NicolosiKrzysztof LewandowskiAgata OrzechowskaKrzysztof MitkoAgata Poniewierska-BaranKrzysztof NawratekAgata Skrzat-KlapaczyńskaKrzysztof NęckaAgata StanekKrzysztof NowackiAgata StempkowskaKrzysztof PańcikiewiczAgata Szpera-GoździewiczKrzysztof ParczewskiAgata SzymańskaKrzysztof PiaskowskiAgata Szymańska-PulikowskaKrzysztof PrzednowekAgata TrzcionkaKrzysztof RostańskiAgata WawrzyniakKrzysztof RząsaAgata WitczakKrzysztof Sas-NowosielskiAgata Wypych-ŚlusarskaKrzysztof SkrzypkowskiAgata Zdun-RyżewskaKrzysztof SmykowskiAgata ŻółtaszekKrzysztof StepaniukAgbotiname Lucky ImoizeKrzysztof TargielAggeliki VerveniotiKrzysztof WięckowskiAgime GerbetiKrzysztof ZdziarskiAgineszka KozlowskaKsakousti SkyllakouAgita LīviņaKsenija Rener-SitarAglaia ZafeiroudiKsenija Tušek-BuncAglaja StirnKsenija VitaleAgnė BrandišauskienėKszysztof ChmielowiecÁgnes Csiszárik-KocsirKuan Rong ChanÁgnes CsivincsikKuang-Chung TsaiAgnes E. DoddsKuang-Ming KuoAgnes LinnérKuang-Ting HuangAgnes PeterfalviKuan-Hui ChenAgnes SanthaKuan-Ying HsiehAgnese PiniKuba PtaszkowskiAgneta Malmgren FängeKuei-I LeeAgnia D. GalachyantsKuei-Yu ChienAgnieska SkowronKuen-Chang HsiehAgnieszka A. KołodzińskaKuew Wai ChewAgnieszka AdamczukKugimiya YoshihiroAgnieszka BaranskaKui ZhangAgnieszka BarczakKukiat TudporAgnieszka BemKuk-Kyoung MoonAgnieszka Białek-DratwaKulasekaran RameshAgnieszka BieńKuldeep ShahAgnieszka BojanowskaKulthida TuamsukAgnieszka BoszczykKulyash MeiramkulovaAgnieszka Bronowicka-SzydełkoKumail Kumar MotianiAgnieszka ChwałczyńskaKumail MotianiAgnieszka CienciałaKumar Chandan SrivastavaAgnieszka Ćwirlej SozańskaKumar DebnathAgnieszka Cydzik-KwiatkowskaKumar VikrantAgnieszka CzerwińskaKumarasamy SudhakarAgnieszka Dettlaff-PokoraKumika TomaAgnieszka DrozdzikKumiya SugiyamaAgnieszka DudziakKumpei MizunoAgnieszka GenerowiczKumud KantilalAgnieszka GolaKun DaiAgnieszka GuligowskaKun GaoAgnieszka HejdukKun HuangAgnieszka HuterskaKun JiaAgnieszka Jagiello-GruszfeldKun LiangAgnieszka Jankowicz-SzymańskaKun SongAgnieszka JankowskaKun TianAgnieszka JastrzębskaKun WangAgnieszka Jaszczuk-MaciejewskaKun ZhangAgnieszka JaworowskaKun ZhaoAgnieszka KamińskaKunal AjmeraAgnieszka KarmanKunal DhimanAgnieszka KasiukiewiczKundi YangAgnieszka Klimkowicz-PawlasKunho LeeAgnieszka KłopotowskaKun-Hua LeeAgnieszka KomorKunihiko AnamiAgnieszka KordekKunihiko NakaiAgnieszka KrupaKunihisa MiwaAgnieszka KulikKuniyoshi ToyoshimaAgnieszka LasotaKunka Mohanram RamkumarAgnieszka LatochaKun-Ming ChenAgnieszka Lecka-AmbroziakKuno HottenrottAgnieszka LorekKunqiu ChenAgnieszka MagryśKuocheng ChungAgnieszka MakaraKuo-Jui WuAgnieszka Malkowska-SzkutnikKuo-Jung LeeAgnieszka MandziukKuo-Shing ChenAgnieszka MarekKuo-Tai ChenAgnieszka Matera-WitkiewiczKuo-Ting SunAgnieszka MatuszewskaKurt A. EscobarAgnieszka MlynarskaKurt FreyAgnieszka Napiórkowska-BaryłaKurt G. M. De CramerAgnieszka NawrockaKurt HeutschiAgnieszka Olszewska-GuizzoKurt LushingtonAgnieszka OrkuszKurtis Jai-Chyi PeiAgnieszka Owczarczyk-SaczonekKurubaran GanasegeranAgnieszka OzgaKurunthachalam KannanAgnieszka Pajdak-StósKushal AdhikariAgnieszka ParlinskaKushal GandhiAgnieszka PękalaKusheng WuAgnieszka Perec-MatysiakKvieskiene GiedreAgnieszka PiekaraKwadwo A. BoakyeAgnieszka PiwkowskaKwadwo Appiah-KubiAgnieszka PlutaKwan Keung NgAgnieszka PodolakKwan Soo ChenAgnieszka Policht-LatawiecKwang Joon KimAgnieszka PróchniakKwanghee JungAgnieszka PuszKwang-Ho LeeAgnieszka RolińskaKwang-Hoon KimAgnieszka RzepkaKwanuk LeeAgnieszka SergielKwok Kuen TsangAgnieszka Siomek-GoreckaKwok Tai ChuiAgnieszka SmalecKwok Wing ChauAgnieszka Sobierajska-RekKwong Nui SimAgnieszka Sompolska-RzechułaKwo-Ting FangAgnieszka SpringerKyeongmin KwakAgnieszka StachowiakKyeongmo KimAgnieszka StanimirKyle B. BillsAgnieszka StarzykKyle BurghardtAgnieszka StrączyńskaKyle D. HopeAgnieszka StrzeleckaKyle K. H. TanAgnieszka Synowiec-WojtarowiczKyle L. ThompsonAgnieszka SzczepańskaKyle MonahanAgnieszka SzczepekKyle MoodyAgnieszka Szlagatys-SidorkiewiczKyle PierceAgnieszka Szmelter-JaroszKyle SueAgnieszka TabernackaKylie GwynneAgnieszka TruszkowskaKyly MillsAgnieszka WareńczakKyoko NagaoAgnieszka WawrzyniakKyonghwa KangAgnieszka WerenowskaKyongok ParkAgnieszka WiśniewskaKyoung KimAgnieszka Wiśniowska-SzurlejKyoung-Chan ParkAgnieszka WnękKyoungkyu JeonAgnieszka Wojewodzka-WiewiorskaKyoung-Min ParkAgnieszka Wojtczuk-TurekKyoungrean KimAgnieszka WoynarowskaKyoungrim KangAgnieszka WsółKyriaki MyrissaAgnieszka ZagórskaKyriakos FlourisAgnieszka Zalejska JonssonKyu Young ChoiAgnieszka Zelek-MolikKyu-haeng LeeAgnieszka Ziernicka-WojtaszekKyung Rae KoAgnitra Roy ChoudhuryKyunghee KangAgnius LiutkeviciusKyung-Hoi KooAgostino CarboneKyung-Hyun SuhÁgota KunKyung-Mee ParkAgripina RaşcuKyungo KimAgu EensaarKyung-Wan BaekAgus NursikuwagusKyungyeol (Anthony) KimAgus SetiawanKyuseok KimAgus UlinuhaKyuwoong KimAgustín Aibar AlmazánL. Douglas RiedAgustín Díaz-ÁlvarezLa Kesha AndersonAgustín HernándezLa Porchia CollinsAgustín Llopis-GonzálezLa Verne L. BrownAgustin Lugo-RadilloLabrini V. AthanasiouAgustín MontesLachezar FilchevAgustín R. MirandaLacrimioara SenilaAgustín Rodríguez-EstebanLadan RokniAhmad AfifyLadislav BláhaAhmad AlbaghdadiLadislav DokládalAhmad AlkhodreLadislav PilařAhmad AlnazzawiLadislav RozenskýAhmad AlsayedLadislav StankeAhmad AlshannaqLadislav ŠtěpánekAhmad BadeenezhadLadislav TomášekAhmad CharifaLady Catherine Cantor-CutivaAhmad Farhan Mohd SadullahLaffranchi MattiaAhmad H. AlomariLahoucine BahsisAhmad H. Juma’hLaia DomingoAhmad HamidovLaia Mas-ExpósitoAhmad HassanLaibané Dieudonné DahourouAhmad Hosseini-BandegharaeiLaibao ZhangAhmad JaliliLailea NoelAhmad JamlehLaima BulotaitėAhmad M. OsailanLaima Jesevičiūtė-UfartienėAhmad Mahdian ParranyLaima SapezinskieneAhmad Munir Che MuhamedLaisa Liane Paineiras-DomingosAhmad Numery Ashfaqul HaqueLaith AbualigahAhmad Razi Ahmad Razi OthmanLaith T. KhraisAhmad Reza MehrabianLajos BorosAhmad SalmanLakmini SenavirathnaAhmad Samed Al-AdwanLakshmi MenonAhmad SayasnehLakshmi VijayakumarAhmad Sufril Azlan MohamedLakshminarayan Reddy TeegalaAhmad Zamri KhairaniLaleh JamshidiAhmad Zare JavidLaleh YerushalmiAhmad ZareiLalit BatraAhmed A. KhalifaLalu Muhamad JaelaniAhmed AbdallaLambert SpaanenburgAhmed AbdelhakLamberto ReAhmed AbdouLamei LeiAhmed AgielLamia EL-GarouiAhmed Al MayyahiLampros PapadimitriouAhmed Al-ZubaydiLan NguyenAhmed Amine AzzazLana B. KarasikAhmed BaghatLana IvanitskayaAhmed BakryLana MinshewAhmed Eid AlprolLana MulierAhmed ElamragyLance KeeneAhmed ElbazLandy LuAhmed ElmassryLang TranAhmed ElmogyLanqing WangAhmed EltweriLanre Ridwan IbrahimAhmed F. KineberLapaire Jean-RémiAhmed FoulyLara ChristoforakosAhmed GaberLara CostantiniAhmed GadLara Marie ReimerAhmed GalhoumLara OrcosAhmed H. MekkawyLara SchlaffkeAhmed HazaziLara WarmelinkAhmed Jibril AbdiLara WöhlerAhmed KarmaouiLaraine WinterAhmed KhaledLarisa AnghelAhmed KheirLarisa MusićAhmed M. Abd-ElGawadLarissa Donatoni Da SilvaAhmed M. AlshehriLarissa Margareta BatranceaAhmed Mancy MosaLarissa StrathAhmed Mohamed ElbazLarissa Vivan CestonaroAhmed Mohamed Fahmy YousefLarissa WieczorekAhmed MouftiLarrell WilkinsonAhmed MustafaLarry NackerudAhmed RakibLarry NelsonAhmed S. El-ShafieLars De VroegeAhmed S. MandourLars DietrichAhmed SamourLars HenningAhmed ShabanLars JacobssonAhmed Sherif AttiaLars JødalAhmed ThabetLars NorgrenAhmed ZahidiLars QvortrupAhmed Zainul AbideenLasse ChristiansenAhmer SiyalLasse LehtonenAhmet AyazLaszlo AlekszaAhmet BaydurLászló AntalAhmet Emre EskazanLászló BaloghAhmet GüneyliLaszlo Barna IantovicsAhmet KoçLászló BencsAhmet ÖzLászló BerényiAhmet ÖzmenLaszlo KornyaAhmet TanhanLászló KótaiAhsan AnwarLászló L˝orinczAhsan UllahLászló ÓzsváriAi Ni TeohLászló SiposAi-Ching YenLászló TóthAida BairamLaszlo ZavoriAida MetoLatasha SmithAida SorianoLatefa DardasAidan CroninLatifah Abd ManafAidé Aracely Maldonado-MacíasLatifah Abdul GhaniAidel SalihLatika NagpalAikaterini GkoltsiouLaura A. DyerAikaterini KaragianniLaura A. ReeseAikaterini KlonariLaura Álvarez De PradoAikaterini PetropoulouLaura Ambra NicoliniAila KhanLaura Andrea Rodríguez-VillamizarAi-Lun YangLaura Andreu-PejóAimee MiddlemissLaura Annabelle BugeningsAimei MaoLaura Antonio-ZancajoAimen KhacharemLaura BragonzoniAimilia KarapostoliLaura BulgariuAimilia MantzouLaura BurattiAinara Díaz-GeadaLaura CasulaÁine BrislaneLaura CataldiAinhoa Fernández AtutxaLaura CerviAinhoa IzaguirreLaura CocoAinhoa Ulibarri OchoaLaura Cortés-GarcíaAinoa Biurrun GarridoLaura DaglioAinoa Roldán AliagaLaura Delgado-LobeteAiqin ShiLaura Domínguez DíazAiri HakkarainenLaura E. Crotty AlexanderAirlane Pereira AlencarLaura E. WalkerAi-Ru HsiehLaura EstebanAisha S. DickersonLaura FedeliAistė PranckevičienėLaura Fernández-RodrigoAitor Garay-SánchezLaura FreemanAitor Oyarbide-ZubillagaLaura FruggeriAivars SpilbergsLaura Garcia-DescalzoAiwei WangLaura Garza MorenoAiya ChantarasiriLaura GibsonAiyoub PezeshgiLaura GuerreroAja AravamudhanLaura GutiérrezAjai AgrawalLaura HemmyAjay Kumar SinghLaura IosifAjay Kumar VermaLaura J. RushAjay MishraLaura KatusAjay MyneniLaura KeysAjaz AhmadLaura Lacomba-TrejoAjda PretnarLaura LessardAjitanuj RattanLaura Louise NicklinAjjampura C. VinayakaLaura Lucas-PalaciosAjoy Prasad ShettyLaura M. EstevesAkane YoshimuraLaura M. RoaAkanksha SharmaLaura MangiaviniAkansha SinghLaura MarchiAkash D. DubeyLaura María CompañAkash DaniaLaura María Varela CrespoAke NilssonLaura MartínAkebe Luther King AbiaLaura MaziluAkemi SawaiLaura MorettiAkhila VeerubhotlaLaura MoschinoAkhmad HabibiLaura Muñoz-BermejoAki TsuchiyagaitoLaura N. CushleyAkifumi EguchiLaura NathansAkihiko HiyamaLaura NunesAkihiko KatayamaLaura OrsoliniAkihiko MurayamaLaura Parra-AnguitaAkihiko YamamotoLaura PiniAkihiro TamuraLaura PompeiAkikazu ShinyaLaura Ramos-PetersenAkinobu UsamiLaura Redondo-GutiérrezAkinola AkinwumijuLaura RizziAkinori NakataLaura RodriguezAkio NozawaLaura Sala-ComoreraAkira IkumiLaura SalernoAkira IshidaLaura SalvadoriAkira KanekoLaura SokalAkira MinouraLaura SparaciAkira ShibanumaLaura StanAkira TaiLaura StefAkira YoshidaLaura StefaniAkito NakagawaLaura Sujo-MontesÁkos PethőLaura SyronAkpovire OduaranLaura Teresa Hernández-SalazarAkram AshamesLaura Torres-ColladoAkram Hernández-VásquezLaura TrifanAkram SeifiLaura UmphreyAkshar SaxenaLaura Valeria FerrettiAkshaya BhagavathulaLaura VicenteAkshyaya PradhanLaura Vidaña MoyaAktas AhmetLaura VismaraAkula VenkatramLaura WilliamsonAkvilė SkirienėLaura ZucconiAl Amin Bin Mohamed SultanLaura-Diana RaduAl Baraa Abdulrahman Al-MekhlafiLaura-María Compañ GabucioAlaa Abd-Alazeez Abu-SrhanLaure Peyro-Saint-PaulAlaa Abdel-HakimLaurel GlennAlain Favre-RéguillonLauren A. FowlerAlain FreyLauren A. ZimmaroAlain JailletLauren BeasleyAlain LescureLauren ButaricAlain MassartLauren DemersAlain RobichaudLauren DundesAlamgir KhanLauren FergusonAlan ApterLauren Hart RollinsAlan Avery-PeckLauren HartsteinAlan BarkerLauren LinesAlan BorthwickLauren O’ConnorAlan Chi Chung FungLauren PetrassAlan CrouchLauren RaineAlan EwertLauren SmithAlan H. DeCherneyLauren WeidnerAlan HallLaurence HamardAlan HuebnerLaurence JolivetAlan LillLaurène LestienneAlan Lins FernandesLaurent BailletAlan M. BeckLaurent CarnisAlan Ricardo Da SilvaLaurent DonzéAlan Santinele MartinoLaurent GallonAlan T. LitchfieldLaurent M. ArsacAlana RojewskiLaurent MourotAlanderson Alves RamalhoLaurent PicotAlasdair HendersonLaurent PlantierAlba Elena Martinez SantosLaurent SuppanAlba García-CidLaurentiu MihaiAlba García-RodríguezLaurentiu TiruAlba Martínez GarcíaLauretta RubiniAlba Paris-AlemanyLauri O. ByerleyAlba Viana LoraLaurie BuysAlban Fouasson-ChaillouxLaurie HofmannAlban KuriqiLaurie Kennedy-MaloneAlban RametteLaurie M. ElitAlbano GonzalezLauro Velazquez-SalinasAlbérico RosárioLavinia CalinoiuAlbert AyeniLavinia DuicăAlbert BachLavinia FaleseAlbert DueggeliLavinia Schuler FacciniAlbert E. ParkerLaw HuaAlbert F. SmithLawrance AnburajanAlbert Kobina MensahLawrence BurnsAlbert LeeLawrence KuroshiAlbert Linton CharlesLawrence Larry MweetwaAlbert MoralesLawrence MabasaAlbert NienhausLawrence MugishaAlbert Pagès BachLawrence PalinkasAlbert PoaterLaxmi Kant SharmaAlbert PostmaLayth Mula HussainAlbert RossiLazar B. DavidovicAlbert SabaterLázaro Florido-BenítezAlbert SabbanLe Hoang PhongAlbert Sanchez-NiuboLea M. MondayAlbert W. WuLea Petrović KrajnikAlbert WabneggerLea SaccaAlbert WhataLea ScherschinskiAlberto AngioniLeah M. HaverhalsAlberto Arnedo-PenaLeah Mae JabillesAlberto CellaLeah R. AbramsAlberto Comesaña-CamposLeah Stein DukerAlberto Enrico MaraoloLeana EsterhuizenAlberto FerraroLeana Ioana CofaruAlberto Ferriz-ValeroLeandro ÁlvarezAlberto Frutos Pérez-SurioLeandro Augusto CalixtoAlberto García GómezLeandro Dias Da SilvaAlberto Gómez-MármolLeandro Miletto TonettoAlberto González-GarcíaLeandro PereiraAlberto M. Torres-CanteroLeandro Torres Di GregorioAlberto MacchiLeanna WoodsAlberto Maria GambelliLebaka Veeranjaneya A. ReddyAlberto Michele FelicettiLech StańczykAlberto ModeneseLechosław DworakAlberto Molina-PérezLechosław Pawel ChmielikAlberto Moreno DoñaLechuang ChenAlberto Quílez-RobresLee FithianAlberto Rodríguez-ArchillaLee Samüel WannAlberto Rodríguez-BarcónLee T. DonohueAlberto Rodríguez-CayetanoLee Wei LimAlberto RomanoLee Wei Rhen WarrenAlberto Ruano-RavinaLee-Ching HwangAlberto Rubén OsellaLee-Kuen ChuaAlberto T. EstevezLeela Subhashini Choudary AlluriAlbina KepalaiteLeena FormaAlbina TummoloLeena KoivusiltaAlcindo NeckelLeena MaunulaAldana RossoLeena ShewadeAldijana BunjakLefteris ZachariaAldo Alvarez-RiscoLei CongAldo Bazán-RamírezLei GaoAldo Bruno GiannìLei HanAldo Muro JrLei HangAldo PezzutoLei JiAldo RoccaLei LiAldona AugustinieneLei ShiAldona Maria PiwkoLei SunAldona ZawojskaLei TanAlec MortonLei WanAleca Ofela-EmaLei XuAlejandra Alonso-CalveteLei YangAlejandra Donají Benítez-ArciniegaLei YaoAlejandra Giselle Juárez-RebollarLei YuAlejandro Alvarado-LassmanLei ZhangAlejandro AmillanoLei ZhaoAlejandro Botero-CarvajalLeigh A. FrameAlejandro Canedo-GarcíaLeigh Ann GanzarAlejandro Corbea-PérezLeigh HaleAlejandro De La ParteLeigh WardAlejandro Fernández GilLeigh WilsonAlejandro GallardLeign-Anne H. KrometisAlejandro Gil-SalmerónLeila AllahqoliAlejandro Guillén RiquelmeLeila MoslehAlejandro Heredia BarberoLeila RahnamaAlejandro Heredia CiuroLeila TahmooresnejadAlejandro Leiva-ArcasLeili MohammadiAlejandro López GonzálezLeire AperribaiAlejandro Lorenzo LledóLeire GordoAlejandro Martínez-RodríguezLeise Kelli OliveiraAlejandro Martín-GorgojoLekshmi R. NathAlejandro MedinaLena HatchettAlejandro Miguel Figueroa-LópezLena HofmannAlejandro MonterrosoLena KaufmannAlejandro Olivares-HernándezLena LämmleAlejandro Pieto AyusoLena Lipskaya-VelikovskyAlejandro Rodríguez-MartínLena Marmstål HammarAlejandro Rodriguez-SanchezLena SchindlerAlejandro Rodríguez-VázquezLena SerafinAlejandro Ruiz-PadilloLena-Karin ErlandssonAlejandro Samhan-AriasLenard ConradiAlejandro San Juan FerrerLenka BranskáAlejandro Sánchez-AtondoLenka ŠindlerováAlejandro Santos-LozanoLenka SokolováAlejandro Sanz-ParisLenka ŠtofováAlejandro StarkLenka SvecovaAlejandro Suárez-PérezLenka VorlováAlejandro Vallina RodríguezLenna Dawkins-MoultinAleka PapadopoulouLennard SchröderAleksandar MiladinovićLenny NawangsariAleksandar StojsavljevićLeo H. ChanAleksandar TrifunovićLeo PruimboomAleksandar V. KlašnjaLeon BrenAleksandar ValjarevićLéon Etienne ParentAleksander GórniakLeon O. H. KroczekAleksander JakimowiczLeonard StoicaAleksander KobylarekLeonardo BascoAleksander LotkoLeonardo BoncinelliAleksander OwczarekLeonardo Catalano IniestaAleksander PanasiukLeonardo CerasinoAleksander StułaLeonardo CesanelliAleksandr BulaevLeonardo DurantiAleksandr I. AbakumovLeonardo Henrique Dalcheco MessiasAleksandr PoliakovLeonardo Jose Mataruna-Dos-SantosAleksandr RomanovLeonardo Juan Ramirez LopezAleksandr RyakhovskyLeonardo LamasAleksandr S. KazachenkoLeonardo Marquez PedroAleksandr VasilievLeonardo MercatantiAleksandra AsaturovaLeonardo MessiasAleksandra BadoraLeonardo NaniaAleksandra BlachnioLeonardo OrnellaAleksandra Buha ĐorđevićLeonardo PellicciariAleksandra CzumajLeonardo PuppulinAleksandra Deluka-TibljašLeonardo RestaAleksandra FucicLeonardo RibeiroAleksandra GawelLeonardo SantosAleksandra Gaworska-KrzemińskaLeonardo SchippaAleksandra GulcLeonel GarcíaAleksandra Jadach-SepiołoLeonel PereiraAleksandra JaremkówLeonel PrietoAleksandra Jezierska-ThöleLeonid KalichmanAleksandra JoticLeonid V. AzarnertAleksandra JungLeonidas EfthymiouAleksandra Karczmarska-WódzkaLeonidas G. IoannouAleksandra KlichLeonidas PetridisAleksandra KnezevLeonie AsconeAleksandra KołotaLeonie BrummerAleksandra KołtuniukLeonie HalloAleksandra KovačevićLeonor Bacelar-NicolauAleksandra KowalskaLeonor SanchezAleksandra KrólikowskaLeonor TeixeiraAleksandra KulisLeonor WardAleksandra ŁapkoLeopoldo FornerAleksandra LedwońLeopoldo Medina SánchezAleksandra Lewandowska-WalterLeping LiAleksandra LisLesley AndrewAleksandra LovyaginaLesley BaillieAleksandra Matuszewska-JanicaLesley Biediger-FriedmanAleksandra Nadgórska-SochaLesley CharlesAleksandra NowyszLesley MachekaAleksandra PawlaczykLesliam Quirós-AlcaláAleksandra RabczunLeslie C. HusseyAleksandra RadeckaLeslie CameronAleksandra RogowskaLeslie Chavez-GalanAleksandra RomanowskaLeslie D. WilliamsAleksandra RyłLeslie ElliottAleksandra S. KristoLeslie J. HinyardAleksandra Sałagacka-KubiakLeslie Jordan AlbertAleksandra SędzikowskaLeslie Ramos SalazarAleksandra StevanovićLeslimar Rios-ColonAleksandra StupakLeso MunalaAleksandra SzajaLeszek ButowskiAleksandra SzczukaLeszek ChomackiAleksandra SzydłowskaLeszek DrabikAleksandra SzylińskaLeszek SłowikAleksandra Tłuściak-DeliowskaLeszek SzalewskiAleksandra WdowczykLeszek WanatAleksandra WilkLeticia A. Neira TovarAleksandra ŻebrowskaLeticia M. Márquez-MagañaAleksandra ZeljkovicLetizia AppolloniAleksandras VilionskisLetizia BindiAleksandre MaisashviliLetizia CasoAleksei RozhnovLetizia CremoniniAleksei TikhonovLetizia GalassoAleksey A. VedyaginLev D. LabzovskiiAleksey FilippovLev Guzmán-VargasAleksy KwilinskiLevent ÇalliAlemayehu GebissaLevente HufnagelAlen ManyevereLewis CheungAlena A. ZolotarevaLewis HsuAlena HricováLewis J. MacGregorAlena OulehlovaLewis Mehl-MadronaAlenka ŠkerjancLeyla Angelica Sandoval-HamonAleš DolnýLeyu WangAleš ObrezaLi BaiAleš SmrekarLi ChaiAleš UrbanLi JinAlessandra AmatoLi LiuAlessandra AmendolaLi ShenAlessandra BoccacciniLi TaoAlessandra CarioliLi TianAlessandra CepparuloLi XuAlessandra ConsalesLi YangAlessandra CostanzaLi YiAlessandra D’AmicoLi ZhaoAlessandra FilippiLia AraujoAlessandra FiorettiLía Celina Méndez RodríguezAlessandra FrigerioLia E. TsotsosAlessandra LaforgiaLiana BadeaAlessandra Lucia FlucaLiana MareeAlessandra M. FerraroLiana NagyAlessandra MedeirosLiana PleşAlessandra PersichettiLianfeng ZhaoAlessandra RinaldiLiang En WeeAlessandra SannellaLiang GongAlessandra SaporitiLiang LiangAlessandra SinopoliLiang LiuAlessandra SorienteLiang PeiAlessandra SpagnoloLiang Ting LinAlessandra StacchiottiLiang WangAlessandra VitulloLiang XuAlessandra ZanolettiLiang YeAlessandro AgostiniLiang ZhangAlessandro AntonelliLiang ZhouAlessandro ArenaLiang-Chih ChangAlessandro BartoloniLiang-Jen WangAlessandro BlasiLiangpeng GaoAlessandro BonadonnaLiangqun LuAlessandro BuonomoLiang-Tseng KuoAlessandro CapucciLianne TrippAlessandro CostantiniLianne UradaAlessandro de SireLiao LiaoAlessandro FailoLiat Hen-HerbstAlessandro FancelluLibby FlynnAlessandro FeolaLibby MooreAlessandro FerrariniLibin JiaAlessandro GermaniLibin Thaikkattil LouisAlessandro GhezzoLibo LiuAlessandro GiorgettiLibo ZhangAlessandro MalobertiLibor AnsorgeAlessandro MantiniLiborija Lugović-MihićAlessandro MarinaccioLiča LozicaAlessandro NotaLichen ChouAlessandro Oronzo CaffòLi-Ching ChangAlessandro PaganoLi-chun HoAlessandro PerrellaLicia P. CacciariAlessandro PetrontinoLícia Passos Dos Santos CruzAlessandro PigoniLicun WuAlessandro RizzoLida FanAlessandro RodolicoLida MademliAlessandro SapienzaLidia BossAlessandro ScanoLidia CaporossiAlessandro SicoraLidia GavicAlessandro StefaniniLidia HrnčevićAlessandro TavelliLidia MaynerAlessandro TonacciLidia MierzejewskaAlessandro VecchiatoLidia PerencAlessandro ViganòLidia PottasAlessandro VittoriLidia ReczekAlessandro ZorziLidia WolnyAlessia D’AndreaLien Rodríguez LópezAlessia Di BlasioLienite LitavnieceAlessia FaggianLiepollo NtlhakanaAlessia GrigolettoLiezhou ZhongAlessia MoroniLifeng WuAlessia PaganelliLifeng ZhouAlessia RemiganteLiga PaulaAlessia RenziLigang LvAlessia SpadaLígia AmâncioAlessio BocediLígia Isoni AuadAlessio CardaciLígia Torres SilvaAlessio ContiLiguo CaoAlessio FacchinLih-Ming YiinAlessio FranchinaLihu YangAlessio GoriLihua LiAlessio MoscaLijie GaoAlessio MylonasLijun WangAlessio PaladiniLikai HuangAlessio RossiLikius DanielAlessio TurcoLikke PutriAlessio ZulianiLikwang ChenAlethea S. MadgettLilac Lev-AriAlex CostleyLilach SoreqÁlex FerrerLili LeiAlex GalanisLili Nemec ZlatolasAlex Harley CrispLili QuAlex J. Van DuinenLili ZhouAlex JosephLilia LesykAlex M. HankeyLilia LukowskyAlex McDowellLilian AzzopardiAlex OkaruLilian KristAlex Richard CookLilian RigoAlex RowlandsLiliana A. EnríquezAlex Siu Wing ChanLiliana CoriAlex SoldererLiliana CunhaAlex TomyLiliana Dell’ossoAlex TorkuLiliana HawryszAlex ViguerieLiliana JacottAlex Yong Kwang TanLiliana KiczakAlexander A. PugovkinLiliana Marquez-BenavidesAlexander BolshoyLiliana MâțăAlexander BungeLiliana PitachoAlexander CancelliLiliana RaduAlexander ChanLiliana Reynoso-CuevasAlexander FaizulinLiliana Szyszka-SommerfeldAlexander GardnerLiliana Veronica DiaconescuAlexander GorokhovskyLilianna BartoszekAlexander HemprichLiliya A. PasechnikAlexander HodeckLiliya Eugenevna ZiganshinaAlexander HošovskýLiliya PoskotinovaAlexander IvannikovLilla KorenovaAlexander J. SchmidtLilla MakszinAlexander John BondLilly AugustineAlexander K. ConverseLilybeth FontanesiAlexander KaltenboeckLim Hwee SanAlexander KerstenLimin MaoAlexander KirpichLiming WangAlexander KrannichLimor GoldnerAlexander L. SemenovLin DongAlexander LaiLin GaoAlexander LogemannLin LuAlexander MaierLin MaAlexander MangoldLin WangAlexander Maniangat LukeLin XuAlexander MeigalLin ZhangAlexander MininLin ZhuAlexander OtahalLina BegdacheAlexander R. LucasLina Díaz-CastroAlexander RobitzschLina GarcésAlexander RufLina HandayaniAlexander S. HatoumLina HeierAlexander SerguninLina JovarauskaiteAlexander SeymerLina LiuAlexander T. GalloLina SpirgienėAlexander TamalunasLina TolmaAlexander ToetLinas BalčiauskasAlexander TrulsonLinbo ZhaoAlexander UngerLinchen HeAlexander WolfLinchuan YangAlexander WuLincoln Da SilvaAlexander ZherebkerLinda Azevedo KauppilaAlexandr Anatoljevich StekolnikovLinda BelauAlexandr CeasovschihLinda BenningtonAlexandr KolesnikovLinda Björkhem-BergmanAlexandr V. KrylovLinda BrennanAlexandra AgostiniLinda DynanAlexandra Cosima BudabinLinda F. MartinAlexandra Courtin-NomadeLinda G. KahnAlexandra Danial-SaadLinda H. NieAlexandra DevineLinda HersheyAlexandra EconomouLinda HouhamdiAlexandra Ferreira-ValenteLinda J. Larson-PriorAlexandra FidalgoLinda K. KayeAlexandra GambaryanLinda K. RauschAlexandra Garr-SchultzLinda Katherine JonesAlexandra González AguñaLinda KuranAlexandra HennesseyLinda LayneAlexandra HoffmannLinda NubaniAlexandra HorobetLinda SaraivaAlexandra Ioana MihăilescuLinda SelveyAlexandra IoanidLinda TizekAlexandra J. MayhewLinda VignozziAlexandra JitǎreanuLinda WidarAlexandra KlimovaLindawati S. KusdhanyAlexandra KordaLindita BandeAlexandra LopesLindsay BottomsAlexandra M. MontoyaLindsay E. YoungAlexandra M. PreisserLindsay GalwayAlexandra MateiLindsay R. ArcurioAlexandra PapadopoulouLindsay RosenfeldAlexandra PehlkenLindsey GreviskesAlexandra PeregrinaLindsey MillerAlexandra PereiraLindsey ShieldsAlexandra Rodrigues GonçalvesLindsy KassAlexandra SawyerLine HillersdalAlexandra Schleyer-LindenmannLing Min TanAlexandra Sofia MendesLing Tim WongAlexandra V. KulinkinaLing ZhangAlexandra Vink-NieseLingen WangAlexandra ZbucheaLinghua DuoAlexandra-Dana ChițimusLingpeng ShanAlexandre A. GuerinLingping ZengAlexandre CorreiaLingsha JuAlexandre Cunha CostaLing-Wen DingAlexandre Garcia-MasLingxin ChenAlexandre GiacobboLingyun LiaoAlexandre González-RodríguezLinh N. HoangAlexandre HudonLinh PhanAlexandre LamasLinhua WangAlexandre MaroisLin-Ju KangAlexandre Mathieu-FritzLinjun YaoAlexandre Morais NunesLinlin FanAlexandre O. Fernande Fernandes Da SilvaLinlin PanAlexandre OliveiraLinlin XieAlexandre PalmaLinnéa HedmanAlexandre R. VieiraLinnea LaestadiusAlexandre RepkineLino FigueiredoAlexandre RibeiroLino Meraz RuizAlexandre SidorenkoLinpeng Andrew ShanAlexandre ValléeLins-E-Silva Ana Carolina BorgesAlexandre VetcherLinsheng YangAlexandre VieiraLinu Sara GeorgeAlexandre Wesley Carvalho BarbosaLinus NyiwulAlexandrina MunteanLinus OlsonAlexandro J. MartagónLinyi WangAlexandros ArgyriadisLinying DongAlexandros DaponteLinyu XuAlexandros GarefalakisLinzi ZhengAlexandros LaiosLionel ObadiaAlexandros MaziotisLionel Sanchez-BolivarAlexandros PoutoglidisLior FuchsAlexandros PsarrisLioua KolsiAlexandros RovasLip Yee PorAlexandru AvramLiping LiAlexandru BănicăLiping LuAlexandru BejinariuLiping SunAlexandru BodislavLipskaya-Velikovsky ElenaAlexandru BucurLiron RozenkrantzAlexandru BurceaLisa A. JuckettAlexandru BurlacuLisa BauleoAlexandru Gabriel BejinariuLisa BirnbaumAlexandru IlieșLisa BricknellAlexandru NedeleaLisa DeLouiseAlexandru Nicolae UngureanuLisa Di BlasAlexandru PavelLisa F. ShubitzAlexandru SalceanuLisa GrenAlexandru Silvius PescariuLisa GugglbergerAlexandru TibaLisa HaleAlexandru UliciLisa HendersonAlexandru VlasaLisa ItoAlexandru-Ionut PetrisorLisa JackaAlexei SlepzofLisa M. MuratoriAlexei VoinovLisa M. RanzenhoferAlexey AfoninLisa MacintyreAlexey BoykoLisa Mary SheehyAlexey DudarevLisa McFetridgeAlexey PenenkoLisa MeierottoAlexey SafonovLisa MillerAlexey SarapultsevLisa ParuchAlexey ShmatkoLisa Pescara-KovachAlexey SuminLisa RubinAlexia RoulandLisa TangAlexios LolasLisa WigginsAlexis DescathaLisa WittenhagenAlexis L. MrazLi-San HungAlexis LionLisandro LovisoloAlexis M. TemkinLisardo BoscaAlexis MrazLisbeth HybholtAlexis Murillo CarrascoLisbeth NilssonAlexis PapathanassisLise Ann St. DenisAlexis RodríguezLise Øen JonesAlexis StamatikosLi-Shiue GauAlfaro RodrigoLisiane Morelia Weide AcostaAlfonsina D’IorioLissa SpencerAlfonso Alvarado-LorenzoLi-Ting KaoAlfonso Garcia De La VegaLiu YiqingAlfonso Gastelum-StrozziLiubov TkachevaAlfonso Gómez-EspinosaLiudmila Gerasimova-MeigalAlfonso Gutiérrez-SantiagoLiudmila GolobokovaAlfonso Infante-MoroLiudmila LiutskoAlfonso José Lopez RiveroLiuqingqing YangAlfonso LorenzoLiv Wergeland SørbyeAlfonso Maria PonsiglioneLīva AumeistereAlfonso Martínez MorenoLivia BurattaAlfonso Martínez-NovaLivia CarvalhoAlfonso Meneses MonroyLivia Ionela BobuAlfonso Penichet-TomásLivia OttolenghiAlfonso Salgado RuizLivia TavernaAlfred GattLiviu Mihai IrimiaAlfred Muluan WuLiviu MoldovanAlfred O. AnkrahLiwei YangAlfred SimonLixia ShangAlfredo BrunoLiya JinAlfredo CoelhoLiyan TianAlfredo G. CasanovaLiyan XuAlfredo IandoloLiyang YangAlfredo Larrosa HaroLiyaning Maggie TangAlfredo MainarLi-Ying LinAlfredo Merino-LunaLiying SunAlfredo Miguel Cabezas AresLi-Yun SunAlfredo Pinedo-AlvarezLiyuwork Mitiku DanaAlgimantas DanilevičiusLiz BreenAli AhmadianLiz FalconerAli AhmedLiz TrenchardAli AkalinLiza JachensAli AlqahtanyLiza LeeAli AlRifaiLiza MortonAli BoolaniLizandra Garcia Lupi VergaraAli BozorgiLizbeth EscobedoAli Danandeh MehrLizeth Guadalupe Parra-PérezAli DaraeiLizethly Cáceres JensenAli Darub KassarLjilja TorovićAli DerakhshanLjiljana NikolićAli DolatiLjubica AndjelkovicAli Enes DingilLjubica Ivanovic-BibicAli FakihLjubica KonstantinovićAli FarnoudLjubiša Šaric̈Ali GoliLjupko ŠimunovićAli Hassan SodhroLluís SanmiquelAli HussainLobina PalamuleniAli ImranLobo Hung-Tak LouieAli JazirehiLobo LouieAli KasalLois JamesAli Kerim YılmazLoknath Sai AmbatiAli KhademLola NeufcourtAli MalekiLolaev AlanAli MawofLoleny TavaresAli Mohammad AlqudahLoliza L.F.H. ChalubAli NadzalanLombart M. Maurice KouakouAli RazaLonda NwadikeAli Sadeghi HabibabadLone Winther LietzenAli Selcuk CanLong ChenAli SenLong Fei WangAli Tan Kee ZuanLong SunAli Vatankhah BarenjiLong T. TruongAli ZolaitLong WuAlia TelliLong ZhaoAliah Zannierah MohsinLongfei YuAliakbar HassanpouryouzbandLongina Chojnacka-OżgaAlice BonomiLongxiang LiAlice BullasLoraine BusettoAlice CampbellLóránd KissAlice Chiara ManettiLóránt Dénes DávidAlice Fiddian-GreenLoredana CerbaraAlice HorowitzLoredana IvanAlice Kit Ying ChanLoredana Marcela TrancaAlice MannocciLoredana R. Diaconu-GherasimAlice MasiniLorel BurnsAlice MicheluttiLorella CannavacciuoloAlice MonzaniLorena AlcarazAlice NcubeLorena Caridad Y. López Del RíoAlice NoblinLorena CharrierAlice OrdeanLorena DadićAlice PorterLorena De Medina-SalasAlice RaffetinLorena Elena MeliţAlice VerticchioLorena Gutiérrez PuertasAlice Wai Yi LeungLorena Gutiérrez-GarcíaAlice Ym JonesLorena Gutiérrez-HermosoAlicia Aguilar MartínezLorena ManeiroAlicia Alonso CarrilloLorena Parra-RodríguezAlicia Blanco-GonzalezLorena Patricia Gallardo-PeraltaAlicia Busto MiramontesLorena RossiAlicia Garcia-TestalLorenz EpprechtAlicia Gil TorresLorenz S. NeuwirthAlicia Martínez-RodríguezLorenzo BrognaraAlicia Mas-TurLorenzo CarusoAlícia Molina-AndújarLorenzo De CarloAlicia NunezLorenzo FaggioniAlicia PerdigonesLorenzo FerriAlicia Ruiz-PomedaLorenzo FranceschettiAlicja BortkiewiczLorenzo GianquintieriAlicja DomagałaLorenzo LippiAlicja GackowskaLorenzo MalatinoAlicja GrześkowiakLorenzo PaglioneAlicja KalusLorenzo PelizzaAlicja KamińskaLorenzo Ricciardi CelsiAlicja KicińskaLorenzo Rivas GarcíaAlicja Kot-NiewiadomskaLorenzo RomanoAlicja Małgorzata GraczykLorenzo Ros-McDonnellAlicja WalosikLorenzo RusticoAlicja Wolny-DominiakLorenzo SpagnoloAliete Cunha-OliveiraLorenzo StacchiniAlim SyariatiLorenzo TonettiAlimujiang KasimuLoreta Buksnyte–MarmieneAlin ArteneLoreta CannitoAlin OpreanaLori BanksAlina BetlejLori GrayAlina BorkowskaLori-Ann R. SacreyAlina CernasevLoriena A. YancuraAlina ChiracuLorin MaletskyAlina Cristina NutaLorna CameronAlina DanetLorna MontgomeryAlina Delia PopaLorraine A. CaleAlina DrozdowskaLorraine A. T. BoakyeAlina Elena MoacaLorraine PhillipsAlina HerrmannLorraine ShackAlina Ioana BadanoiuLotta TikkanenAlina Kałużna-WielobóbLotte HughesAlina Kowalczyk-JuśkoLotte Vallentin-HolbechAlina LatoLouie Mar A. GangcuangcoAlina MomotLouis ArnouldAlina PancewiczLouis FavrilAlina PrussLouis KleinAlina SchartnerLouis MoustakasAlina Simona RusuLouis Shing Him LeeAlina SoceanuLouis Wy LiuAlina VedutaLouisa M. HolmesAlina ZajadaczLouise BrådvikAlina-Cerasela AluculeseiLouise BroughAlina-Petronela HallerLouise Brown NichollsAline Alves FerreiraLouise ChawlaAline Braga Galvão Silveira FernandesLouise HopperAline GratienLouise HughesAline HoninghLouise MarstonAline Ramond-RoquinLouise McCabeAline ViancelliLouise MobergAlin-Sasa TisanLouise MoodyAlipasha MeysamieLouise MurphyAlireza AbouhosseinLouise Norman JespersenAlireza GoliLouise PittAlireza MoghayediLouise TannerAlireza MohammadiLouise TownsinAlireza Nikbakht NasrabadiLouise TraceyAlireza SahebgharaniLouise TullyAlisa GrigorovichLouise UnderdahlAlisa WrayLouiza DimowaAliseri GovardhanLoukas ChatzisAlisha PrasadLoukia ChrysikouAlison ClappLounassaari Anne Kristiina KurjenojaAlison EastmanLourdes Chocarro GonzálezAlison HerrmannLourdes LledóAlison MitchellLourdes Martinez-NietoAlison OrmsbyLourence Misedah-RobinsonAlison PurvisLovorka BrajkovićAlison R. CoelhoLu JiangAlison Sally PoultonLu WangAlissa Der SarkissianLu ZhangAlissa GreerLua Perimal-LewisAlissa M. BanyaszLuan SantosAlisu Schoua-GlusbergLuana Andreea MacoveiAlix WoolardLubna Abu-NiaajAlix YoungĽubomíra TkáčováAliya NazĽuboslav DulinaAlla BelousovaLuc E. H. VanlinthoutAlla KasychLuc HensAllan BennettLuc IngenbleekAllan G. HillLuca AltamoreAllbens AtmanLuca AmbrosioAllen KrautLuca BattagliniAllen MalloryLuca BattistiAllen Sam TitusLuca CandeloroAllison AndrukonisLuca CavaggioniAllison FordLuca CegolonAllison HodgeLuca CerritelliAllison Huff MacPhersonLuca CoppetaAllison RossLuca D’AcciAllison SmithLuca D’AciernoAlma EspartinezLuca Di GiampaoloAlMahamdy MohammedLuca Di GianfrancescoAlmir KarabegovicLuca FacontiAlmotasembellah AbushabanLuca FalzoneAlmudena Buesa-EstellézLuca FerraroAlmudena Cotán FernándezLuca FlesiaAlok MishraLuca FontanaAlok PandeyLuca FreddiAlok PaulLuca FredianelliAlon BergmanLuca GallelliAlon DavidyLuca Giovanni LocatelloAlona Emodi-PerlmanLuca ListaAloys BorgersLuca MagistrelliAlpamys IssanovLuca MarinAlper TuğralLuca MiceliAlperen S. BingoelLuca Nicola Cesare BianchiAlpo VuorioLuca OrtensiALTI AdelLuca PaduaAltug HasozbekLuca Paolo ArdigòAlvan Emeka Kelechi UkachukwuLuca Paolo WeltertAlvarado-Ibarra MarthaLuca PetrignaÁlvaro Antón-SanchoLuca PiccoliÁlvaro Astasio PicadoLuca PoliÁlvaro Briz-RedónLuca RollèÁlvaro Campos Cavalcanti MacielLuca RussoÁlvaro Clemente VivancosLuca SalvatiAlvaro Del RealLuca Salvatore De SantoÁlvaro Edgar González-Aragón PinedaLuca Salvatore MengaÁlvaro FernándezLuca SimioneÁlvaro Francisco Lopes De SousaLuca SoraciAlvaro García Del CastilloLuca TestarelliÁlvaro Huerta OjedaLuca TianoAlvaro InfantesLuca TóthÁlvaro Lopes DiasLucas A. SalasAlvaro Lopez-EscamillaLucas D. DiasAlvaro PérezLucas Kniess DebarbaAlvaro VergésLucas Melo NevesÁlvaro Zubizarreta-MachoLucas Raphael Bento E. SilvaAlvin Kai-Xing LeeLucia BednárováAlvin LeeLucia BettediAlyona IvanovaLucia BottiAlyssa R. MorseLucia CarboniAlyssa SbisaLucia CarichinoAlyx TaylorLucia CastelliAlžbeta PultznerováLucia CiciollaAlzira Maria Paiva de AlmeidaLucia CugusiAmador Durán-SánchezLucía García-San MiguelAmador Jesús Lara SánchezLucia GozzoAmagliani GiuliaLucía Granados AlósAmaia IrizarLúcia Helena Gomes CoelhoAmaia YurrebasoLucía I. LinaresAmal M. El-NaggarLucia KaiserAmal MitraLucia KantorováAmal SabaLucia KendrováAmalia CorneaLucia KundisovaAmalia GordanoLucia Lomba-PortelaAmalia Luque-SendraLucia LombardiAmalia Raquel Pérez-NebraLucia MangoneAmalia Sillero-SilleroLucia MirabellaAman KarimLucía MireteAman SinghLucía Moure-RodríguezAmand FührerLucia MuracaAmanda B. CooperLucia Nacinovic PrskaloAmanda BradberyLucia Peixoto-PinoAmanda CalebLucia RatiuAmanda ChamiehLucia RoccoAmanda Du PlessisLucía Rodríguez-LópezAmanda HellstromLucia RonconiAmanda KowalczykLucia SchiavonAmanda LosiLucia SlovinskáAmanda M. Farias ZunigaLucia StančiakováAmanda M. WilsonLucia TabacuAmanda Martín MariscalLucia TerzuoliAmanda MisselLucia Tomas-AragonesAmanda N. Szabo-ReedLucia Vieira HoffmannAmanda PacholakLucian CopoloviciAmanda PalmerLucian Toma CiocanAmanda RamosLuciana B. NucciAmanda RoomeLuciana CaenazzoAmanda V. SardeliLuciana Diniz SilvaAmanda WilsonLuciana LabancaAmandine JunotLuciana OliveiraAmani Al-OthmanLuciana Pereira XavierAmany HabibLuciana ReisAmany HassanLuciana RochaAmany SholkamyLuciana SpinelliAmar KanekarLuciana Varanda RizzoAmar KrishnaLuciane F. De GoesAmar Nath ChatterjeeLuciane Viater TureckAmar ParvateLucian-Ionel CiocaAmaranta De Miguel-RubioLuciano BossoAmaranta FocardiLuciano Ferreira Da SilvaAmaranta McGee-LasoLuciano SilvaAmarender ReddyLuciano SperandioAmauri Serrano-LázaroLucica BarbesAmaya Epelde-LarrañagaLucie Biehler-GomezAmaya Erro GarcésLucie DrábováAmaya Lobo García de CortázarLucie HympánováAmbar Lopez-MacayLucie ViktorovaAmbarish PawarLuciene Pimentel Da SilvaAmber BarnesLucija GosakAmber SousaLucija VejmelkaAmbereen Kurwa MehtaLucila IshitaniAmbrina A. QureshiLucille HeadrickAmbrish SinghLucille Joanna BorlazaAmeersing LuximonLucimere BohnAmel TaibiLucinda J. BessaAmelia Beata StaszowskaLucio CappelliAmélia CaldeiraLucio Della GuardiaAmelia DíazLucius BotesAmelia GulliverŁucja BieleninikAmelia ManutiŁucja Zielińska-TomczakAmélia MarchãoLucjan StrekowskiAmelia Maria GămanLucrecia Mena-MeléndezAmelia R. TuragabeciLucreția UdrescuAmélia SilvaLucrezia Ginevra LulliAmélia Simões FigueiredoLucy J. TroupAmelia TrematerraLucy O’MalleyAmelia V. García-LuengoLucy TullyAmenan Agnès KouaméLucy WebbAmérica Chávez-MartinezLucyna Błażejczyk-MajkaAmerigo GiudiceLucyna Polak-JuszczakAmey S. TilakLucyna PrzybylskaAmeya BehereLucyna ŚcisłoAmgad MuneerLucyna TomaszekAmi CohenLucyna WitekAmi RokachLuděk KalužaAmi SeivwrightLudger HelmsAmichai Ben AriLudi Wishnu WardanaAmicizia DanielaLudivine MartinAmid P. KhodadoustLudmiła Daniłowicz‑SzymanowiczAmila AbeynayakaLudmiła Grzybowska-SzatkowskaAmin AlizadehĽudmila MajerníkováAmin KeramatiLudmila Silva GuimarãesAmin MojiriLudo VanopdenboschAmin Mousavi KhaneghahLudovic-Mohamed ZahedAmin N. OlaimatLudovico DipinetoAmin ShahrokhiLudwig ApersAmin Talebi Bazmin AbadiLudwik WickiAmina El YaagoubiLuguang WangAmina NasriLuigi Alberto PiniAmine GhramLuigi AldieriAminul Islam ApuLuigi BennardoAmir BegićLuigi BiaginiAmir BhochhibhoyaLuigi CarboneAmir HadiLuigi CastaldoAmir MinerbiLuigi CavannaAmir MohammadiLuigi CelaniAmir MuzurLuigi CipolloniAmir Reza Khavarian-GarmsirLuigi CorvoAmir S. SirajLuigi DattolaAmir TjollengLuigi de NapoliAmir WaseemLuigi Della CorteAmira AbdellatefLuigi Di FilippoAmira ElasraLuigi EspositoAmirhossein Khalili-GarakaniLuigi GrecoAmirhossein MirhashemiLuigi La ViaAmirmasoud AhmadiLuigi LeccaAmirreza MahbodLuigi MeccarielloAmirul MukmininLuigi NicolaisAmit AgarwalLuigi RuggieroAmit ChandelLuigi SabatiniAmit GuleriaLuigi SantacroceAmit K. JaiswalLuigi SenatoreAmit K. MaitiLuigi TommasinoAmit K. SinghLuigi VimercatiAmit KennyLuigi ViolaAmit KumarLuis A. RojasAmit Kumar JaiswalLuis Adrián Zúñiga AvilésAmit L. JainLuis Albendín-GarcíaAmit M. JoshiLuis Alberto Gaitán-CepedaAmit SharmaLuis Alberto Marco-ContrerasAmit SinghLuis Alejandro Lopez-AgudoAmit TripathiLuis AlvesAmit U. RaysoniLuis Andre MendesAmita AttleeLuis Andreu CaravacaAmita BhaktaLuis Augusto SilvaAmitava RakshitLuis Bernardo López-SosaAmith MaroliLuis BotellaAmjad AliLuis BranquinhoAmjad KhabazLuis Bravo-MoncayoAmjad MasoodLuís Carlos De Souza FerreiraAmlan HaqueLuís Carlos Lopes-JúniorAmm QuamruzzamanLuís Carlos ProlaAmmar Abdulrahman JairounLuis Carlos Sandoval-HerazoAmmar AltemimiLuis Carlos Venegas-SanabriaAmna AnjumLuis Carrasco PáezAmol S. PatwardhanLuis CarroAmpaiwan ChuansumritLuis Ceballos-LaitaAmparo HuertasLuis Cobos-PucAmparo Verdú-VázquezLuis Del Espino-DíazAmpol KaroonsoontawongLuis DouradoAmr Ahmed El‑ArabeyLuis E. EspinozaAmr FoudaLuis Eduardo Akiyoshi Sanches SuzukiAmr HassanLuis Enrique Valdez-JuárezAmrendra MishraLuis Espejo-AntúnezAmritpal Kaur Kaur KhakhLuis Exequiel IbarraAmro AlzghoulLuis F. AristizábalAmulya YaparlaLuis F. MartinezAmund MaageLuís FaíscaAmutha RamadasLuís Felipe Dias LopesAmy BrowerLuis Fernández-PozoAmy FehlLuis Fernando Jacinto-AlemánAmy HarmsLuis Fernando MachadoAmy HinkelmanLuís Fernando SchützAmy IshamLuis G. FernándezAmy JohnsonLuis Garcia-MarcosAmy L ShaverLuis Gerardo Zepeda VallejoAmy LeeLuis Gómez PérezAmy Miner RossLuis Ignacio Gonzalez GranadoAmy MooreLuis Ignacio Lopera GonzálezAmy PedenLuis LeitãoAmy SchuhLuís Lima SantosAmy WarhurstLuis M. Álvarez-SabucedoAna AleksićLuis Manuel Cerdá SuárezAna Alexandra GoraLuis Manuel Martínez ArandaAna Álvarez MuelasLuis Manuel Sánchez RuizAna AmaroLuís MargalhoAna BankoLuis Mariano EstebanAna Batlles-DelafuenteLuis Marqués MolíasAna Beatriz Furlanetto PachecoLuís MartinsAna Beatriz OliveiraLuis Miguel Cairós-VenturaAna Belén Fraile-BermúdezLuis Miguel CalvoAna Belén Santos-OlmoLuis Miguel Dos SantosAna Beleń Subirón-ValeraLuís Miguel MassuçaAna Bianca PavelLuis Miguel PérezAna BlascoLuis Miguel Sánchez LoyoAna BorovečkiLuís MonteiroAna Carla BatissocoLuis MontenegroAna Carolina Bernardes TerzianLuis Núñez-JaramilloAna CaruntuLuis Octavio Tierradentro-GarcíaAna Catarina MaiaLuis Ortiz-HernándezAna Cláudia CoelhoLuis PatinoAna Cláudia Mesquita GarciaLuís PinhoAna Cláudia Morais Godoy FigueiredoLuís Pinto FerreiraAna CoelhoLuís RamaAna CondeLuis Raúl Tovar-GálvezAna Cordellat MarzalLuis RodrigoAna Cornelia BadeaLuis Rodrigo MartínAna Corte-RealLuis Salvador-RodríguezAna Costa RosadoLuis Sánchez-LabradorAna Cristina Faria RibeiroLuis SantosAna Cristina GonçalvesLuis SordoAna Cristina MarquesLuis ToroAna Cristina Rodrigues LacerdaLuis Torrego EgidoAna Cristina Silva RebeloLuis V. GarcíaAna DascaluLuis VacaAna Diez-IzquierdoLuis Valero AguayoAna Elisa Madalena RinaldiLuis Vicente Amador MuñozAna Ercília JoséLuis Villalobos-GallegosAna Fernández-AraqueLuis Zavala-ArciniegaAna Filipa CardosoLuísa Bandeira LopesAna Filipa PoeiraLuisa D’AmoreAna Filipa SilvaLuisa Losada PuenteAna Flávia Pires Lucas D’OliveiraLuisa PastiAna FonsecaLuisa QuevedoAna FrancaLuisa SchneiderAna Fresán OrellanaLuísa SoaresAna Gascón-CatalánLuisa VarrialeAna GavrilovićLuiz Antonio Alcântara PereiraAna Gentil-GutiérrezLuiz Carlos De AbreuAna Gómez-TafallaLuiz Felipe SilvaAna GundićLuiz Fernando Romanholo FerreiraAna Hernandez De PolaczykLuiz Flávio Cordeiro FernandesAna I. Lopez NavasLuiz Gustavo Dos Anjos BorgesAna I. SoteloLuiz Henrique Castro DavidAna Iolanda VodăLuiz Palucci-VieiraAna Irina Lequeux DincaLuiz Peres-NetoAna Isabel Alvarez-MercadoLuiza Breitenfeld GranadeiroAna Isabel Beltrán-VelascoLuiza Marek-JózefowiczAna Isabel BorgesLuiza Mesesan SchmitzAna Isabel Córdoba-IñestaLuiza OchnioAna Isabel Fraguas-SánchezLuiza OssowskaAna Isabel González ContrerasLujun SuAna Isabel LouroLuk Hsiang NingAna Isabel MolinaLuk JimAna Isabel Ponce GeaLuka FlegarAna Isabel Rosa AlcázarLuka GrbcicAna Isabel SantosLuka Martin TomažičAna IspasLuka MatakAna IvaniševićLuka NovačkoAna J. Cañas-LermaLukas D. LandeggerAna Jimenez-GarciaLukas RichterAna Josefina Monjarás-ÁvilaLukas StreeseAna Junça-SilvaŁukasz A. PoniatowskiAna Jurinjak TušekŁukasz AlbańskiAna KezićŁukasz B. LewandowskiAna KorenŁukasz BakaAna Lanero CarrizoŁukasz BalwickiAna LeahuŁukasz BorekAna Leonor Abrahão NencioniŁukasz CichockiAna López-DuránŁukasz DembińskiAna Lúcia TerraŁukasz DobrekAna LuísŁukasz FijałkowskiAna Luísa Moreira CostaŁukasz JachAna Luísa Patrício da SilvaŁukasz JanuszkiewiczAna Luiza De Carvalho FelippiniLukasz JaskiewiczAna M. Cebrián-CuencaLukasz KaczmarekAna M. SahagunLukasz KolodziejAna M. Tur-PorcarŁukasz KozarAna MachadoŁukasz KrystAna MalfitanoLukasz MalekAna Manzano-LeónŁukasz MazurAna Marchena RodríguezLukasz MendykAna Margarida Ferreira‪Łukasz MokrosAna Maria Alexandra StanescuŁukasz MusiakaAna Maria Cebolla AlvarezŁukasz PałkaAna Maria Costa CarneiroŁukasz PietrzykowskiAna Maria Cristina TancuŁukasz PopławskiAna Maria DucasseŁukasz RadzimińskiAna María Fernández-AlonsoŁukasz RydzikAna Maria Gondim ValençaŁukasz RypiczAna María Hernández CarrteroŁukasz RzepińskiAna Maria Mantilla HerreraŁukasz SatołaAna Maria Mihaela IordacheŁukasz SikorskiAna María Pérez-ZuriagaLukasz SkowronAna María Ruiz-Ruano GarcíaLukasz SkrokAna María Salinas-MartínezŁukasz TomczykAna María Vázquez-CasaresŁukasz TotaAna María Velasco MolpeceresŁukasz TyburskiAna Marta AleixoŁukasz WiechetekAna Marta GonçalvesŁukasz WiktorAna MolinaŁukasz WirkusAna MonteiroŁukasz ZadrożnyAna Mudarra-FernándezŁukasz ZapałaAna NicolauLuke BalcombeAna NikezićLuke ConnellyAna P. AntunesLuke Del VecchioAna Patrícia DuarteLuke J. NeyAna Paula Cunha LoureiroLuke MontroseAna Paula MonteiroLuke WoonAna Paula Rodrigues CavalcantiLukus KlawitterAna PenezicLulu ChenAna PereiraLulu FanAna PlácidoLulu QuAna R. AriasLuma AkilAna Remesal OrtizLuminita MarmureanuAna Rita CarlosLuminita NicolescuAna Rita Xavier De Jesus GameiroLuminita PopaAna RochaLunda ShenAna RodriguesLung ChanAna Rodriguez LarradLuo MingyaoAna Rodríguez-MartínezLupak RuslanAna Ruivo AlvesLusi ZhangAna S. SousaLut TamamAna SabinoLütfü AşkınAna Serrano TelleríaLuuk Van KempenAna Seselja PerisinLuwanika MleraAna Sofia Ruivo AlvesLuyu XieAna Sofia VinhasLuz Garcia-LongoriaAna Susana AlmeidaLuz Maria Del Razo JiménezAna Teresa Corte-RealLuz Maria Sánchez-RomeroAna Teresa Ferreira-OliveiraLuz Zas VarelaAna Teresa GabrielLuzaan HamiltonAna Teresa MartinsLuz-María Martín-DelgadoAna Težak DamijanićLy Sy Phu NguyenAna TomasLydia Giménez-LlortAna VasićLydia SobotovaAna VazquezLydia Theresia RösslAna Victoria AriasLydi-Anne Vézina-ImAna VillarroyaLykourgos MagafasAna Virginia FilgueirasLyndon LimAna VuinLynette HodgesAna VukovicLynn ChenowethAna ŽivkovićLynn GrattanAnabel OrellanoLynn LevatteAnabela AfonsoLynn M. PezzaniteAnabela Baptista PaulaLynn PrestonAnabela Correia MartinsLynn RusyAnabela Marisa AzulLynne CohenAnabela MesquitaLynne MillarAnabela Salgueiro-OliveiraLynnette BrundermanAnagha MedsingeLynnette M. JonesAnagnoste SorinLyubka AleksievaAnahid Ahmadi BirjandiLyubov KhomyakovaAna-Isabel Corregidor-SánchezLyubov KlapkivAnalia KaradayianLyudmila DimitrovaAnália Maria De Matos ClérigoLyudmila Mikhailovna SoldatkinaAnalivia BarbosaLyudmila RachekAnam NyembeziM. Ariel Geer WallaceAnamaria BechirM. Chandra SekarAna-Maria CazanM. Filomena TeodoroAnamaria CiubaraM. J. Ruzmyn VilcassimAnamaria Cozma-PetruţM. Lenin Lara CalderónAna-Maria GeorgescuM. S. ZobaerAnamaria JurcauMa De Los Ángeles Merino GodoyAna-maria PelinMª Del Carmen Pegalajar PalominoAna-Maria PutzMa Easter Joy SajoAnamaria SavuMa’mon M. HatmalAna-Maria TaloșMac GardnerAnamarija Jurčev SavičevićMac KirbyAnanda SidartaMacarena Castellary LópezAnantha HarijithMacarena Donoso GonzálezAnanto AlhasyimiMachodi MathabaAnanya ChakrabortyMaciej BielińskiAnapaula Sommer VinagreMaciej BroszAnarul Haque MondolMaciej ChęcińskiAnas AhmadiMaciej CieplakAnastasia BauerMaciej CyranAnastasia ChristodoulouMaciej DobrzańskiAnastasia ChristopoulouMaciej DobrzyńskiAnastasia D. PournaraMaciej DyrbuśAnastasia DontiMaciej DzikućAnastasia MarkakiMaciej GliniakAnastasia MarshakMaciej J. NowakAnastasia MiklyaevaMaciej JedlińskiAnastasia NikologianniMaciej KlockiewiczAnastasia PapadimitriouMaciej KochmanAnastasia PapavasileiouMaciej KruszynaAnastasia PopelnukhaMaciej KubońAnastasia ProdromidouMaciej LasockiAnastasia SaadeMaciej OesterreichAnastasia Tellez InfantesMaciej PolakAnastasiia D. ShkodinaMaciej PrzybyłekAnastasiia KrivoruchkoMaciej RyczkowskiAnastasios SepetisMaciej SalagierskiAnastasiya KostryukovaMaciej SerowaniecAnastassia ZabrodskajaMaciej SikoraAnasua BhattacharyaMaciej StaszakAnat Yaskolka MeirMaciej StolarskiAnatolii KucherMaciej SydorAnatoliy GoncharukMaciej W. SochaAnatoly A. SidorovMaciej ZalewskiAnber RanaMaciej ZastempowskiAnbupalam ThalamuthuMaciej ŻukowskiAnca Adriana ArbuneMack ShelleyAnca Antoaneta VărzaruMacmanus NdukwuAnca BobircăMadakasira Vasantha Padma SrivastavaAnca BojanMădălina Simona BălțatuAnca Daniela IonitaMădălina-Gabriela IliescuAnca Francisca CruceruMadankumar GhatgeAnca MehedintuMaddalena FiordelliAnca Oana DoceaMaddalena IllarioAnca PaulencoMaddi Garmendia AntinAnca SimionescuMade SuciptaAnca TudorMadeeha MalikAnca TurtureanuMadeleine DobsonAnca ZanfirescuMadeleine FranceAnca ZguraMadeline SprajcerAnca-Florentina VatamanuMadelon KronemanAnca-Maria ClipaMadelon L. FinkelAnca-Ștefania MesaroșMadhavi Thombre KulkarniAncel JulienMadhavi VenkatesanAnchuelo Arturo CorbatónMadhuja SamaddarAncuța ChetrariuMadhulata Singh ChauhanAncuța RotaruMadhulika PradhanAncy JosephMadoc SheehanAnders HenningsenMaegala Nallapan Nallapan ManiyamAnders HjernMagagula Vusi MpenduloAnders KochMagali BoizumaultAnders LarssonMagda Guimarães De Araújo FariaAnders Van SandtMagdalena Anna Zwolińska-LigajAnderson AraMagdalena B. SkarżyńskaAnderson Ribeiro DuarteMagdalena BłaszczykAndi MabhalaMagdalena BłażekAndi RrokuMagdalena Cyma-WejchenigAndis KalvansMagdalena CzarnogorskaAndjela MarkovicMagdalena Elizabeth Bergés TiznadoAndoni Beristain IraolaMagdalena Emilia GrzybowskaAndoni Carrasco-UribarrenMagdalena GantnerAndra PorutiuMagdalena GarlinskaAndra ZvirbuleMagdalena Gayà-VidalAndras BanvolgyiMagdalena GębskaAndras BikovMagdalena Gizińska-GórnaAndrás BótaMagdalena GórnickaAndrás Donát KovácsMagdalena Graczyk-KucharskaAndrás FehérMagdalena GuzyAndrás István KunMagdalena HagovskaAndrás LakatosMagdalena Hartman-PetryckaAndras LekoMagdalena Iordache-PlatisAndrás MolnárMagdalena IorgaAndrás RabMagdalena JancAndrás SimonovitsMagdalena JasiniakAndraẑ DovnikMagdalena K. WekenborgAndré Alves DiasMagdalena KąkolAndre ComandonMagdalena Kogut-JaworskaAndré FringerMagdalena Kolańska-StronkaAndré Geraldo BerezukMagdalena KończakAndré L. L. BachiMagdalena KorżyńskaAndré Lima AiresMagdalena KostrzewaAndré Luis Costa-Da-SilvaMagdalena KowalówkaAndré Luís Ferreira De MeirelesMagdalena KozelaAndré Luiz Monezi AndradeMagdalena KrajewskaAndré M. CarringtonMagdalena Kubal-CzerwińskaAndre NienaberMagdalena Kurnik-ŁuckaAndré NohlMagdalena Kwiatosz-MucAndré NovoMagdalena LazarewiczAndré OttoniMagdalena LeszkoAndré Pimenta FreireMagdalena LitwinskaAndré RamalhoMagdalena Łukaszewska-KuskaAndré RamonMagdalena M. StussAndré Riani CostaMagdalena MaciaszczykAndré Ricardo Araujo Da SilvaMagdalena MadełaAndre RodackiMagdalena Mądra-SawickaAndré Souza OliveiraMagdalena Maria PopowskaAndré SteimersMagdalena Markowicz-PiaseckaAndre WirriesMagdalena MititeluAndrea A. PappalardoMagdalena MyszuraAndrea AgugliaMagdalena NarajczykAndrea Álvarez-SalaMagdalena OhajaAndrea BaldiniMagdalena OledzkaAndrea BeccioliniMagdalena Paplińska-GorycaAndrea BergerMagdalena PlandowskaAndrea BoscuttiMagdalena PlatisAndrea BotturiMagdalena Polak-ŚliwińskaAndrea BrambillaMagdalena PtakAndrea ButeraMagdalena RaczyńskaAndrea Cecilia Serge RodríguezMagdalena RaftowiczAndrea CeolinMagdalena Ramos Navas-ParejoAndrea ChiricoMagdalena Regel-RosockaAndrea CioffiMagdalena ReizerAndrea CiricugnoMagdalena RyśAndrea ClementsMagdalena SenzeAndrea ContiMagdalena SkoniecznaAndrea CortegianiMagdalena SkotnickaAndrea D. FurlanMagdalena Śmiglak-KrajewskaAndrea D. OlmsteadMagdalena SobieskaAndrea De VitoMagdalena SobocińskaAndrea Di CredicoMagdalena SzymańskaAndrea Di PaolantonioMagdalena Waszyk-NowaczykAndrea DincherMagdalena Weber-RajekAndrea Edoardo BianchiMagdalena Wilkosz-MamcarczykAndrea FalegnamiMagdalena Wojnowska-HeciakAndrea FarnhamMagdalena WróżyńskaAndrea FischbachMagdalena WrzesińskaAndrea FontanaMagdalena WyszynskaAndrea FontesMagdalena ZabochnickaAndrea FransonMagdalena ZaborowskaAndrea FrosoliniMagdalena ZajączkowskaAndrea FuscoMagdalena ZawadkaAndrea GalisovaMagdalena ŻegleńAndrea GamaMagdalena ZgliczynskaAndrea GažováMagdalena Zimakowska-LaskowskaAndrea GelemanovicMagdalena ŻychowskaAndrea GibbonsMaged M. A. FoudaAndrea GlässelMageswaran SanmugamAndrea GöhringMaggie Beiting-ParrishAndrea GuazziniMaggie Bohm-JordanAndrea Gutiérrez GarcíaMaggie Ka Wai LauAndréa Gutierrez MariaMaghsoodi Tilaki Mohammad JavadAndrea Hernández-MartínezMagne BråtveitAndrea Herrera-SantelicesMagnus AkerstromAndrea HickersonMagnus BlingeAndrea HobkirkMagnus Friestad Bjørkavoll-BergsethAndrea Hulina-TomaškovićMagnus SvartengrenAndrea JoensenMaha AliAndrea JorgensenMaha Emad IbrahimAndrea KaifieMaha HoteitAndrea KleindienstMahdi AfkhamiaghdaAndrea L. HildebrandMahdi KhosravyAndrea L. Huseth-ZoselMahdi ShariatiAndrea LampisMahdieh AlipourAndrea LazoMahesh KasthuriAndrea LoviselliMahesh KaushikAndrea M. P. RomaniMahesh PattabiramanAndrea M. PotocnyMahesh RachamallaAndrea ManciniMahfuzur RahmanAndrea ManconiMahip AcharyaAndrea ManfrinMahipal Singh SankhlaAndrea MarcinnòMahirah KamaludinAndrea Martín-VacasMahmood Al ShareedaAndrea MaugeriMahmoud ElbattahAndrea Milostić-SrbMahmoud Fawzy Mahmoud MoustafaAndrea MininiMahmoud IbrahimAndrea Monica OrtizMahmoud KandeelAndrea MulliriMahmoud Mohammad Rezapour TabariAndrea NavaMahmoud NasrAndrea NeriMahmoud R. SofyAndrea Norcini PalaMahmoud SherifAndrea NoveMahmudur RahmanAndrea PeruzziMahnoosh KholghiAndrea Piano MortariMahomet NjoyaAndrea PintorMahrous A. IbrahimAndrea PödörMahrous Abdelbasset IbrahimAndrea PoloMahsa OroojeniAndrea PozziMahyar ArefiAndrea QuadriMahyar Habibi RadAndrea R. TeufelbergerMai MatsumotoAndrea R. TitusMai Narasaki-JaraAndrea RebecchiMai Salah El Din Mohamed HelmyAndrea Rene Mary MohanMai TakaseAndrea RieblerMaider Kortajerena RubioAndrea RoccuzzoMaija Huttunen-LenzAndrea Roxanne J. AnasMaikel RosabalAndrea SalustriMaiko SakamotoAndrea SambriMaili MalinAndrea SansoneMainul HaqueAndrea SantarelliMaiorana AntonioAndrea ScatenaMaite Martín-AragónAndrea SegaliniMaitreyee MukherjeeAndrea SevcovicovaMaitreyi ShivkumarAndrea SpeltiniMaja AbramAndrea SpinazzèMaja Cigrovski BerkovićAndrea TamburranoMaja CupicAndrea TateoMaja KoraćAndrea TinelliMaja KrzeljAndrea TittarelliMaja MeškoAndrea TrevisanMaja MiskovicAndrea VázquezMaja MiskulinAndrea Vukic DugacMaja Ortner HadžiabdićAndrea WheelerMaja PtasiewiczAndrea YatscoMaja RochAndreas BrandMaja Rogić VidakovićAndreas DemetriouMaja RožmanAndreas F. BorkensteinMaja Ružić-BafAndreas FlourisMaja SmrduAndreas HamburgerMaja ŠubeljAndreas HeinMaja TurnsekAndreas IhleMajdy IdreesAndreas KochMajed AlamriAndreas LeichMajid HosseinzadehAndreas MeinzerMajid KhayatnezhadAndreas RösslerMajid KoozehchianAndreas RowaldMajid Mufaqam Syed-AbdulAndreas TsatsarisMajid MuradAndreas VilhelmssonMajid NiazkarAndreas VollmerMakama Andries MonyekiAndreas WahnerMakella S. CoudrayAndreas WaltherMaki Tei-TominagaAndreas WarnkeMakiko KouchiAndreas WibowoMakoni PedzisaiAndree HartantoMakoto AnrakuAndreea AndronesiMakoto EndoAndreea AvasiloaieiMakoto HasegawaAndreea BorleaMakoto KomiyaAndreea BrabeteMakoto KondoAndreea Catarina PopescuMakoto MorinagaAndreea Iuliana KuiMakoto TsukaiAndreea Maria IordacheMaksim MoskovskiyAndreea MiricăMaksim RebezovAndreea-Daniela MoraruMaksym ŁaszewskiAndreea-Loreta CercleuxMaksymilian MądzielAndrei BorșaMaksymilian SolarskiAndrei C. HolmanMalachi J. McKennaAndrei C. PopescuMalan Ketcha Armand KablanAndrei CrisanMalcolm KooAndrei DumitrescuMalcolm Scott DuthieAndrei GrigorievMaleda TeferaAndrei HolmanMalek Ben Ahmed AdouniAndrei Jean VasileMałgorzata AdamczukAndrei MitreaMałgorzata B. PańkowskaAndrei RusuMałgorzata BlatkiewiczAndrei ShpakouMałgorzata BuksińskaAndrei VasilateanuMałgorzata Buksińska-LisikAndreia CostaMałgorzata BurchardAndreia De Bem MachadoMałgorzata CharmasAndreia FreitasMalgorzata Cimochowicz-RybickaAndreia GeraldoMałgorzata ĆwiekAndreia LimaMałgorzata DȩbiakAndreia Maria Silva Vilela TerraMalgorzata DobrzynskaAndréia PelegriniMalgorzata DominoAndreia QueirozMałgorzata DudzińskaAndreia Rodrigues Moura Da Costa ValleMalgorzata DziembalaAndreina CarboneMałgorzata E. Górnik‑DuroseAndrej KirbišMałgorzata Ewa DrywieńAndrej MarkotaMałgorzata FlagaAndrej StarcMalgorzata Gamian-WilkAndrej ThurzoMałgorzata GolemanAndrej VelasMałgorzata GrabaraAndreja SinkovicMałgorzata GutAndrés Alonso Agudelo-SuárezMałgorzata J. GambinAndres Barajas-SolanoMałgorzata JabłońskaAndrés Barrios-RubioMałgorzata JamkaAndrés Caballero-CalvoMalgorzata JarossovaAndres Carmona HernandezMałgorzata KaczorowskaAndrés Fernando Avilés-DávilaMałgorzata KaczyńskaAndrés Gené SampedroMałgorzata Kęsik-BrodackaAndrés Godoy-CumillafMałgorzata KisilowskaAndres Pandiella-DominiqueMałgorzata KołodziejAndres Pastor FernandezMalgorzata KolpaAndrés RedchukMalgorzata Komorowska-KaufmanAndres Reinoso-CoboMałgorzata Kosicka-GębskaAndrés Rodríguez-SeijoMalgorzata KosteckaAndrés Rojo RojoMałgorzata KowalAndrés Sánchez-PradaMalgorzata KoziolAndrés Velástegui-MontoyaMałgorzata Lesińska-SawickaAndressa Melina Becker Da SilvaMałgorzata MacudaAndressa Silva MelloMalgorzata Maria JerzakAndreu Casero-RipollésMałgorzata MarkowskaAndreu Comas-GarciaMałgorzata Mikaszewska-SokolewiczAndrew A. GuccioneMałgorzata MizgierAndrew B. RosenblumMałgorzata MoszakAndrew BudsonMałgorzata MoszyńskaAndrew C. PoveyMałgorzata MusiałAndrew Chih Wei HuangMalgorzata NagórskaAndrew ClementsMałgorzata Natonek-Wi’sniewskaAndrew D. HathawayMalgorzata OkreglickaAndrew E. LittmannMalgorzata PankowskaAndrew Elohim LalooMałgorzata PasekAndrew GrannellMałgorzata Pawlaczyk-ŁuszczyńskaAndrew HatchettMałgorzata PawlakAndrew HollandMalgorzata PawulAndrew HursthouseMałgorzata PieniążekAndrew J. GoldbergMałgorzata PikalaAndrew J. OnesimuMałgorzata Plechawska-WójcikAndrew JenkinsMałgorzata Polz-DacewiczAndrew John ChapmanMałgorzata PoniewozikAndrew K. GormleyMałgorzata Przybysz-ZarembaAndrew KaczynskiMałgorzata Rutkiewicz-HanczewskaAndrew KingsnorthMałgorzata RutkowskaAndrew KiraguMalgorzata SkibinskaAndrew LafrenzMałgorzata Skucha-NowakAndrew LavenderMalgorzata SobieszczanskaAndrew LothianMałgorzata StachońAndrew M. LaneMałgorzata StasińskaAndrew MartelMałgorzata StefaniakAndrew MayMałgorzata SzczęśniakAndrew MitchellMałgorzata SztubeckaAndrew MitchelmoreMałgorzata TeleckaAndrew O’HaraMalgorzata TrochaAndrew O’ReganMałgorzata Trofimiuk-MüldnerAndrew ObergMałgorzata WaszakAndrew PapadopoulosMałgorzata WęgrzyńskaAndrew PeplowMałgorzata WiśniewskaAndrew PodgerMałgorzata WójcikAndrew PoveyMałgorzata WojtkowskaAndrew Rebeiro-HargraveMałgorzata Worwa̧gAndrew ReevesMałgorzata WoźnickaAndrew RossettiMałgorzata ZnykAndrew S. FranksMalia HoAndrew S. MerryweatherMalihe Mehdizadeh AllafAndrew ScottMalik AydinAndrew W. BartlowMalik JawarnehAndrew WhittingtonMalik SajidAndrew WillisMalik SallamAndrew WisterMalin ErikssonAndrey G. CherstvyMalin Lohela-KarlssonAndrey KozlovMalinee SriariyanunAndrey P. ZhdanovMalka MargalitAndrey SidorenkoMallesh KurakulaAndrey SivkovMallie J. PaschallAndriati NingrumMallikharjuna KoriviAndries S. KosterMalte BraackAndries Van Der BiltMalvin N. JanalAndrija BernikMalwina Michalik-ŚnieżekAndrija MatetićMaman TurjamanAndris FreivaldsMamoona HumayunAndriy SverstyukMan Fai LeungAndry ChowandaMan Lai CheungAndrzej AdamskiMan YuanAndrzej BąkMana RaoAndrzej BiłozorManal KleibAndrzej BłażejewskiManas KotepuiAndrzej BlecharczykManat SrivanitAndrzej BrudnickiMandakhbayar Nandin-ErdeneAndrzej BryśMandar KhanalAndrzej CzamaraMandeep S. JassalAndrzej DługońskiMandi LarsenAndrzej FalMandu EkpenyongAndrzej HeckerMandy StahreAndrzej J. PiotrowskiMandy VisserAndrzej JanowskiManela GlarcherAndrzej KacprzakManfred BornewasserAndrzej KasperskiManfred KöhlerAndrzej KicińskiManfred Man-Fat WuAndrzej KlasaManfred NeubergerAndrzej KlimczukManfred SagerAndrzej KobylińskiManfred SchmittAndrzej KoniecznyManfredo ManfrediniAndrzej KozłowskiManhai LongAndrzej KrasnodębskiManiaci MichaelAndrzej ŁachaczManikandan MuthuAndrzej M. ŚliwczyńskiManikandan RamachandranAndrzej MarcinkiewiczManikant TripathiAndrzej MichalskiManinder KaurAndrzej MikulskiManish BhattAndrzej MroczkowskiManish BhattaraiAndrzej MuczyńskiManish DhawanAndrzej MyśliwiecManish KumarAndrzej NowickiManish Kumar JainAndrzej NowrotManish PandeyAndrzej OstrowskiManish SharmaAndrzej PacanaManish TripathiAndrzej PokrywkaManish Vijaya KumarAndrzej R. SkrzypczakManja ZecAndrzej RaszkowskiManju M. ChandraAndrzej RybakManlio SantilliAndrzej SkrętManmeet SinghAndrzej SkwierawskiManoela FerreiraAndrzej ŚliwerskiManohar KodavatiAndrzej SzubaManoj PardasaniAndrzej SzymkowiakManoj RayarothAndrzej TomczakManoj SharmaAndrzej TomskiManolis AdamakisAndrzej TretynManolis KousloglouAndrzej WnorowskiManon LemondeAndrzej Za̧bekManon NayracAndy BeryMansoor SoomroAndy C. Y. TseMansour AsseryAndy ChanMansour HaddadAndy PringleManuel A. Ortega BecerraAndy SmithManuel A. ValdebranAndy Wai Kan YeungManuel Arlindo A. MatosAndy WaldhelmManuel AurelianoAndy Yen-Tung TengManuel Au-Yong-OliveiraAneela Zameer DurraniManuel Casal-GuisandeAnel Gómez GarcíaManuel Castelo BrancoAneliya MilanovaManuel Chavarrias OlmedoAneta AleksovaManuel Coheña-JimenezAneta Alicja Cymbaluk-PłoskaManuel De La SenAneta Bełdycka-BórawskaManuel Fernández-AlcántaraAneta GrochowskaManuel Ferrández-VillenaAneta KosztowniakManuel Florindo Alves MeirinhosAneta KowalskaManuel Gámez-GuadixAneta KrogulskaManuel García-GarcíaAneta OcieczekManuel García-SilleroAneta Ostróżka-CieślikManuel González-GómezAneta Pawłowska-LegwandManuel Herrero-MontesAneta PiechalakManuel IsornaAneta R. BorkowskaManuel J. Rodríguez OrtegaAneta SlodekManuel J. Ruiz MuñozAneta SpyraManuel Jesús Hermoso-OrzáezAneta Svozilíková KrakovskáManuel JiménezAneta WieczorekManuel Joaquín De Nova-GarcíaAneta WłodarczykManuel Jordan-VidalAneta WoźniakManuel Jose LisAnetta BarskaManuel José LopesAnetta MüllerManuel Lázaro PulidoAnette GanskeManuel Luís CapelasÁngel Alberto MagreñánManuel MadrazoAngel Alberto Valdés-CuervoManuel Marques FerreiraAngel AlgarinManuel MatosAngel BatuecasManuel Monfort-PañegoAngel Benigno Gonzalez AvilesManuel Orta-PerezAngel Buendía-RomeroManuel OttavianoAngel BurovManuel Pabón-CarrascoÁngel CastroManuel Palomo-DuarteÁngel Custodio Mingorance-EstradaManuel Pardo-RiosÁngel De-Juanas OlivaManuel PeralboÁngel Denche-ZamoranoManuel PereiraAngel F. GonzálezManuel PintoÁngel Gil De MiguelManuel Prado-VelascoAngel Gustavo Salas-LaisManuel RachÁngel L. GuerreroManuel Rebollo-SalasAngel López-GonzálezManuel Rich-RuizAngel Miramontes CarballadaManuel SabaAngel Paniagua MazorraManuel Salas-VelascoAngel R. BarchukManuel Sánchez De MiguelAngel Romero ColladoManuel Sanchez-DiazÁngel Rosa AlcázarManuel Sánchez-MollaÁngel Sabino Mirón SanguinoManuel Soriano-FerrerÁngel Torres-ToukoumidisManuel StruckÁngel Vicario-MerinoManuel Trinidad-FernándezAngela AmmirabileManuel Uceda-RodríguezAngela AndreellaManuel Vargas-VargasÁngela Ayén-RodríguezManuel Ventura-MarcoAngela CartaManuel WoschankAngela CastoliManuela AvadaneiAngela CesaroManuela BarretoAngela ColucciManuela BinaAngela CostabileManuela CasulaAngela Curtean-BănăducManuela De SarioAngela DaddowManuela Expósito-RuizAngela De LeonManuela FarrisAngela DewManuela HeimbeckAngela DieterichManuela HoedlAngela Georgia CaticManuela LarguinhoÂngela GonçalvesManuela LuccheseAngela Graciela Deliga SchroderManuela MauceriAngela HatteryManuela Moreira Da SilvaAngela L. RidgelManuela Ortega-GilAngela LoberManuela Reyes-EstebanezAngela LupattelliManuela SilvaAngela M. Garcia SanchezManuela Sofia Rodrigues MoratoAngela Martin GutierrezManuela VerissimoÂngela MartinsManuele ManciniAngela MeadowsManulani Aluli MeyerAngela MertigMao-Chou HsuAngela NevesMaoshan LiAngela Plaß-ChristlMapuana C. K. AntonioÁngela Sanjuán-QuilesMara ClementeAngela SantilioMara LombardiAngela SpoialăMara ManenteAngela StortiniMara Rúbia CelesAngela StufanoMarat RudakovAngela SyMarc A. FagelsonAngela TolottiMarc AtkinsAngela Turner-WilsonMarc BascomptaÁngeles SánchezMarc BirkhölzerAngélica Angélica Bulcão Portela-LindosoMarc DeanAngélica Juárez-LoyaMarc Eric S. ReyesAngelica Martins De Souza GoncalvesMarc JacquinetAngelica RutherfordMarc JarczokAngélica Saraí Jiménez-OsorioMarc KlimstraAngelika V. TimofeevaMarc KoyackAngeliki AngelidiMarc L. De BuyzereAngeliki PapageorgiouMarc LochbaumAngeliki SarellaMarc M. JacquinetAngelique M. BlackburnMarc SaezAngélique Nicolas MessiMarc TasséAngelo B. HookerMarc VidalAngelo BelardiMarc WeinsteinAngelo CagnacciMarc WittlichAngelo CapodiciMarc WittmannAngelo Earvin ChoiMarcel AdlerÂngelo JesusMarcel AebiAngelo MorretoMarcel FedákAngelo RegaMarcel HanischAngelo RiccioMarcel I. EjimoforAngelo RosaMarcel KunrathAngelo SabagMarcel LemireAngelo SpenaMarcel MinutoloAngelo Valerio MarzanoMarcel Olde RikkertAngelos SikalidisMarcel Rolf PfeiferAnggoro HartopoMarcel SchneiderAngharad Vernon-RobertsMarcel TwicklerAngia Sriram Pradeep RamMarcel Van De WouwAngie Herrera-RMarcela González-GrossAngkoon PhinyomarkMarcela KanovaAngom Ramcharan SinghMarcela Martín-Del-CampoAngus YuMarcela Petrová KafkováAnida Maria BabtanMarcela Sefora NemteanuAniello MaieseMarcello CandelliAnik YuestiMarcello CherchiAnil GumberMarcello CovinoAnil K. ChaudharyMarcello Di MartinoAnil Kumar JonnalagaddaMarcello M. VeigaAnil PoudelMarcello MaddaloneAnil TharappelMarcello NonnisAnindya GangulyMarcello PintiAnindya Sundar RayMarcello RubertiAniruddha HazraMarcello ValoriAniruddha RayMarcelo A. Mena-CarrascoAnis CerovacMarcelo BarrosAnish JantraniaMarcelo BertellotiAnisuz ZamanMarcelo CoertjensAnisuzzaman AnisuzzamanMarcelo De Paula CorrêaAnita A. DaviesMarcelo Dell’AringaAnita BorosMarcelo FerreiraAnita BregenzerMarcelo FurlanAnita ButeraMarcelo J. P. PaixãoAnita DeakMarcelo MatsudoAnita HabókMarcelo MedeirosAnita Jagroop-DearingMarcelo NascimentoAnita Kloss-BrandstätterMarcelo PalinkasAnita KukulskaMarcelo Sánchez-OroAnita LennoxMarchi LiaAnita MajchrowskaMarcia B. ErazoAnita MikolajczykMarcia ErazoAnita PadmanabhanunniMárcia Marília Gomes Dantas LopesAnita PešaMárcia P. SilvaAnita Silvana Ilak PeršurićMarcia Pescador JimenezAnita SinghMarcial Velasco GarridoAnita StasulaneMarcie MiddlebrooksAnita SubramanianMarcílio Vieira Martins FilhoAnita TanglMarcin ArciszewskiAnita TrezonaMarcin BanasiukAnita VetraMarcin Bartłomiej ArciszewskiAnja Edith GeislerMarcin BasiakAnja KolaričMarcin BogdańskiAnja KräplinMarcin BrzezickiAnja KühnelMarcin CichoszAnja MenzelMarcin DębowskiAnja Susanne MühlfeldMarcin DerwichAnja WeiseMarcin DrewekAnjali JayakumarMarcin F. CisekAnjana BairagiMarcin FeldoAnjana SinghMarcin GąsiorAnjani Ravi Kiran GollakotaMarcin GierachAnjul KhadriaMarcin GierekAnk de JongeMarcin GłodniokAnke NeumannMarcin JaneckiAnkie Tan CheungMarcin JanuszAnkit GilaniMarcin KalarusAnkit JhaMarcin KicinskiAnkit KumarMarcin KorzeńAnkit Kumar YadavMarcin KozakiewiczAnkit PatelMarcin KuniewiczAnkit SanejaMarcin LemanowiczAnkit SrivastavaMarcin MaciejczykAnkita PunethaMarcin MorońAnkita SrivastavaMarcin OlkiewiczAnkur JamwalMarcin PasekAnkur SharmaMarcin PełkaAnmol PahwaMarcin PotrykusAnn C. OlssonMarcin RelichAnn Charlotte FalkMarcin RóżewiczAnn HermanssonMarcin SchmidtAnn KirkmanMarcin SemczukAnn LargeyMarcin SidorukAnn M. BergerMarcin SiwekAnn M. DozierMarcin SochalAnn M. SwartzMarcin SpychalaAnn Marie ChizewskiMarcin SzerszeńAnn SvenssonMarcin SzymańskiAnna A. AntsiferovaMarcin TomsiaAnna Adamiok-OstrowskaMarcin WaligóraAnna Adamus-MatuszyńskaMarcin WitkowskiAnna Alekseevna MikhaylovaMarcin WnukAnna Arnal-GómezMarcin WołekAnna AseevaMarcin ZielinskiAnna AugustynowiczMarcio BasilioAnna AureliMarcio Costa De SouzaAnna Babicka-WirkusMárcio F. SimãoAnna Bach-FaigMárcio Falcão Santos BarrosoAnna BagieńskaMárcio Lima GrossiAnna BajerMarcio OliveiraAnna BaraMárcio Poletti LauriniAnna BartkowiakMarcio ValkAnna BartosiewiczMarco A. Jiménez-GonzálezAnna BednarekMarco AlfanoAnna BerardiMarco Alfonso PerroneAnna BiernasiukMarco Antonio Hernández-LepeAnna BizońMarco Antonio Loza-MejíaAnna BjärtåMarco Antonio Zamora AntuñanoAnna BocchinoMarco Aurelio M. FreireAnna Bocheńska-SkałeckaMarco Aurélio Pereira HortaAnna BrzeckaMarco BardusAnna CampanatiMarco BarozziAnna CapaldoMarco BertaminiAnna CapozziMarco BiagiAnna CarcellerMarco CalabròAnna ChallaMarco CapeceAnna Chrobak-ŽuffováMarco CarmignaniAnna CieślińskaMarco CascellaAnna ColucciMarco ClariAnna CybulskaMarco ContardiAnna CzopekMarco D’AlessandroAnna DanielewiczMarco De AngelisAnna De MarcoMarco Del RiccioAnna DegórskaMarco DettoriAnna Dewalska-OpitekMarco DevecchiAnna DipaceMarco Di SarnoAnna Drelich-ZbrojaMarco DolciAnna DubownikMarco E. M. PelusoAnna Duda-MadejMarco Echeverria-VillalobosAnna E. Van Der WathMarco EneaAnna EdgestonMarco EspositoAnna Escribà-SalvansMarco FaggellaAnna EspartMarco FerrariAnna FaustmannMarco FioreAnna FigasMarco GervasiAnna FuchsMarco GesiAnna Gardocka-JałowiecMarco GuicciardiAnna Garus-PakowskaMarco InvernizziAnna GitterMarco La MarraAnna GkioulekaMarco LauriolaAnna GliszczyńskaMarco Mario FerrarioAnna GolędzinowskaMarco MasoeroAnna Goodman HooverMarco MatassoniAnna GórkaMarco MeucciAnna Gotkowska-PłachtaMarco MeyerAnna GranàMarco MiraniAnna GrontkowskaMarco MontaltiAnna Grzywa-CelińskaMarco MontanariAnna HartonMarco NaiaAnna HaywardMarco NisiAnna HeffronMarco ParoliniAnna Irena SzymańskaMarco PortelliAnna J. MilewskaMarco PrenassiAnna JaglarzMarco RaceAnna JakubczakMarco RoccettiAnna Jakubska-BusseMarco Sanchez-GuerraAnna Jamroz-WiśniewskaMarco SapienzaAnna Janas-NazeMarco SeracchianiAnna JędrzychowskaMarco SiinoAnna Jupowicz-GinalskaMarco SpadaAnna Justyna Werner-JuszczukMarco TallaricoAnna Kabłak-ZiembickaMarco TatulloAnna KałużaMarco TerreniAnna Kamińska-DwórznickaMarco TjioeAnna KamzaMarco TogniAnna Karin LindqvistMarco TomiettoAnna Kasielska-TrojanMarco Trabucco AurilioAnna KilanowiczMarco TrifuoggiAnna KittaMarco ValeriAnna KitunenMarco VassalloAnna KłakMarco VecchiatoAnna Klara MocovaMarco VelicognaAnna KleczkaMarco ZaffanelloAnna KlepackaMarco ZeddaAnna Kołodziej-ZaleskaMarcos Antônio Pereira Dos SantosAnna KonstanciakMarcos Arturo Martínez-BanaclochaAnna KostkaMarcos BrioschiAnna KostopoulouMarcos Edgar HerkenhoffAnna Kovács-GyőriMarcos Eduardo ScheicherAnna KozajdaMarcos FerassoAnna KożuchMarcos Fernando Xisto Braga CavalcantiAnna KsiążekMarcos Marcos MichaelidesAnna KurbatovaMarcos Mecías-CalvoAnna Kurek-GóreckaMarcos Pazos-CouseloAnna KwasiborskaMarcus D. LanceAnna KwiatkowskaMarcus LancéAnna L. SchwartzMarcus LettauAnna Lenart-BorońMarcus RedaélliAnna Leńczuk-GrubaMarcus SchiltenwolfAnna ŁepeckaMarcus SchreinerAnna LeśkówMarcus StiemerAnna LevinssonMarcus Tullius ScottiAnna LewandowskaMardelle McCuskey ShepleyAnna LipertMare Lõhmus SundströmAnna LipowiczMaree L. InderAnna LocatelliMaree SimpsonAnna LubkowskaMareike ReimannAnna LuganiniMarek AngowskiAnna Luiza SzeszMarek AsmanAnna LyakhovitskyMarek BienkoAnna M. Zalewska-JanowskaMarek BodzianyAnna MachalińskaMarek ChaleckiAnna Machoy-MokrzyńskaMarek FolAnna MajdaMarek FraněkAnna Małgorzata KamińskaMarek GaworskiAnna ManidakiMarek IlzeckiAnna Marcinkowska-GapińskaMarek JaśkiewiczAnna Maria AddamoMarek JudaAnna Maria AnyżewskaMarek KonefałAnna Maria CostaMarek KozińskiAnna Maria GianniniMarek KruszewskiAnna Maria HeikkinenMarek LapkaAnna Maria MalagoniMarek LiszewskiAnna Maria MeyerMarek MajdanAnna Maria OlszańskaMarek MerhautAnna Maria PaganoniMarek MotykaAnna Maria Różańska-WalędziakMarek MotylewiczAnna Maria ZalewskaMarek NahajowskiAnna MarkiewiczMarek Patrycjusz OgryzekAnna Marzec-GrządzielMarek PetrášAnna Matras-BolibokMarek PieszkaAnna Matysik-WozniakMarek PopowczakAnna Mazurek-KusiakMarek RembierzAnna Mempel-ŚnieżykMarek RistAnna MichnikMarek RyczekAnna MnatsakanovaMarek ŠírAnna MolterMarek ŠnircAnna Moniuszko-MalinowskaMarek StawowyAnna MrzljakMarek StoroškaAnna N. BerlinaMarek SzafraniecAnna NapiórkowskaMarek SzajtAnna NieróbcaMarek SzelągowskiAnna NowakowskaMarek UrbanikAnna OdoneMarek VagašAnna Ogonowska-SlodownikMarek VochozkaAnna OlczakMarek WesołowskiAnna Olewicz-GawlikMarek WieruszewskiAnna Paradowska-StolarzMaren GoeckenjanAnna ParolaMaren SchniederAnna PartykaMaren WeissAnna PiekarskaMaresca Attard PizzutoAnna Pilewska-KozakMarey Allyson MacdonaldAnna PiroMarga GorisAnna Ploch-JankowskaMargalida Coll-AndreuAnna PolakMargaret A. ReadAnna Porczyńska-CiszewskaMargaret B. MitchellAnna PorterMargaret CookAnna PresicciMargaret Eleanor CruickshankAnna ProkopiakMargaret FitchAnna RichiedeiMargaret J. EggersAnna Rita IrimiásMargaret KayAnna RivaMargaret MacAndrewAnna RogalaMargaret MangionAnna RogalskaMargaret P. AdamAnna Rogozińska-PawełczykMargaret PedenAnna Romaszko-WojtowiczMargaret Shirley MutuAnna Rosa DonizzettiMargaret ThorntonAnna RosofskyMargaret WallenAnna RozaliyaniMargareta GregurovićAnna RozensztrauchMargarida Espírito-SantoAnna Rybarczyk-SzwajkowskaMargarida Pedroso De LimaAnna S. MakarovaMargarida PereiraAnna Sączewska-PiotrowskaMargarida QuinaAnna SaitiMargarida SantosAnna Sánchez-CaballéMargarida SaraivaAnna SaponeMargarida VieiraAnna ShutalevaMargarita A. MorozovaAnna SkórzewskaMargarita BakaevaAnna SobczakMargarita EcheverriAnna SojkaMargarita Gozalo DelgadoAnna Solé-LlussàMargarita Martín-MartínAnna Sołtysik-PiorunkiewiczMargarita Ortiz-TalloAnna Sonia PetronioMargarita PoteyevaAnna SorrentinoMargarita Rodríguez-PérezAnna StaniszewskaMargarita TorrenteAnna StarzyńskaMargherita IzziAnna StasiakMargherita Micheletti CremascoAnna StaszczukMargherita NeriAnna StaszewskaMargherita PallocciAnna Stefanowicz-BielskaMargit SerbanAnna StefańskaMargo TurnbullAnna Strychalska-RudzewiczMargot DudkiewiczAnna SykułaMargot Gage WitvlietAnna Sylwia KowalskaMargret Sibylle EngelAnna SzczegielniakMargrethe F. Horlyck-RomanovskyAnna Szczerba-TurekMargrit LoebnerAnna SzücsMaría A. Mendoza-BecerrilAnna SzumilewiczMaria A. PinoAnna SzwajcaMaría A. Vázquez-SánchezAnna TirkkonenMaria Adele GiamberardinoAnna TronoMaria Adriana HenriquesAnna TrubetskayaMaria Alejandra DecimaAnna TsvetkovaMaria Alessandra SotgiuAnna TurkinaMaria Alexandra BernardoAnna TychmanowiczMaria Alexandra PanăAnna Tyrańska-FobkeMaria Alexandra PapadimitriouAnna V. OppenheimerMaría Alicia CaminaAnna Vila-MartíMaría Alonso-GarcíaAnna Vittoria MattioliMaria Andrée López GómezAnna Vladimirovna KomarovaMaria AndronicAnna Wai San CheangMaria Angela PellegrinoAnna Waśniewska-WłodarczykMaria Angela Zaccarelli-MarinoAnna WichowskaMaria Angeles Caballero-MoraAnna Wieczorek-SzymańskaMaria Angeles Cuadrado-CenzualAnna WierzbickaMaria Angeles Martinez RuizAnna WłochMaria Ángeles Pérez MorenteAnna Wołoszyn-DurkiewiczMaria Angeles RojoAnna Wondołowska-GrabowskaMaría Angels Colomer CugatAnna WrzosekMaría Angustias Sánchez OjedaAnna Wziątek-StaśkoMaria Antonia BrighettiAnna ZalewskaMaria Antonia López-AntónAnna ZamkowskaMaría Antonia OvalleAnna ZellmaMaría Antonia Parra-RizoAnna Zielińska-ChmielewskaMaria AntoniadouAnna ZwierzchowskaMaria António CastroAnnabel Ahuriri-DriscollMaria ArmaouAnnabel Müller-StierlinMaría Arregui GambúsAnnabel WalshMaria AspriAnnabelle HoferMaria Assunta ZanettiAnnabelle NeallMaría Asunción Lara CantúAnnachiara AldrovandiMaría Asunción VicenteAnnachiara CavaliereMaria Augusta Rocha BezerraAnnah MosaloMaría Auxiliadora De Vicente OlivaAnna-Karina HermkensMaria AzradAnna-liisa TammMaria BagattiniAnnalina LombardiMaria BaltogianniAnnalisa AnzaniMaria BarsanAnnalisa BargelliniMaría Bastida DomínguezAnnalisa CappellaMaria Belén Prados-PeñaAnnalisa De BoniMaría Belén San Pedro VeledoAnnalisa De SilvestriMaria Blanco-DiazAnnalisa Di BernardinoMaria BogdanAnnalisa GrandiMaria Borszewska-KornackaAnnalisa LevanteMaria ButakovaAnnalisa MonacoMaria C. AlonsoAnnalisa PinsinoMaría C. FuentesAnnalisa QuattrocchiMaria C. LinderAnna-Maria AnderssonMaria C. T. CangussuAnnamaria FicaraMaria CamposAnnamaria GerardinoMaría Campo-ValeraAnnamaria ManciniMaria Carla TesiAnnamaria ZsakaiMaria Carmela RoccheriAnnarita Di MiseMarià CarullaAnnarita SignorielloMaria CasagrandeAnnarosa Del MistroMaria CassarAnne Beate ReinertsenMaria CeleiroAnne BertholdMaria Chiara CascheraAnne Christine WoolridgeMaria Chiara GallottaAnne Day LeongMaria Chiara GattoAnne EvensMaria Chiara TorricelliAnne Faber HansenMaria ChristouAnne GarveyMaria Claudia SurugiuAnne Geert Van DrielMaria Conceição MansoAnne HoneyMaria Concepción UnanueAnne JörnsMaria Concetta D’OvidioAnne JuengertMaría Consuelo Reguera SuárezAnne Kristin M. FellMaría Consuelo Velázquez-AlvaAnne KuhntMaria ContaldoAnne M. ForemanMaria Cristina BudaniAnne M. WeaverMaria Cristina BularcaAnne M. WhiteMaria Cristina CostaAnne MäkirantaMaría Cristina Del Rincón-CastroAnne Marie SowerbuttsMaria Cristina MaroldaAnne Marie ZimeriMaría Cristina Martínez-FernándezAnne MeißnerMaría Cristina OrtizAnne MillsMaria Cristina Triguero Veloz TeixeiraAnne Roback MorseMaria CristofaroAnne RoskenMaría CustodioAnne SimmenrothMaria Da Luz Ferreira BarrosAnne Sofie LansøMaria DaglaAnne Sophie BaudryMaria DâmasoAnne TaufenMaria De Fátima FerreiroAnne-Catherine Pierson-WickmannMaria De Fátima MarquesAnne-Christine PlankMaria De Fátima Martins Lorena De OliveiraAnnegret Pelchen-MatthewsMaria De Fátima Pereira Da SilvaAnne-Katrin JordanMaría De La Concepción Noriega MatanzaAnneke A. VeenstraMaría De La Cruz Del Río-RamaAnne-Laure Sutter-DallayMaría de las Nieves González GarcíaAnneli MatssonMaría De Los Angeles Rodríguez DomenechAnnelies RoggemanMaria De Los Ángeles Cardero-DuránAnnelise J. BlombergMaría De Los Ángeles Cosío-LeónAnne-Lise K. VelezMaría De Lourdes BolañosAnne-Maria LaukkanenMaría de Lourdes García AnayaAnne-Marie CallusMaria de Lurdes DinisAnnemarie Reinhardt VarmingMaría Del Carmen Carcelén-FraileAnnemarie WentzelMaría Del Carmen García-MendozaAnnemie SpoorenMaría Del Carmen García-RíosAnne-Priscille TrouvinMaría Del Carmen García-RodríguezAnne-Sophie MreyenMaria Del Carmen Lozano EstevanAnne-Sophie WoznyMaría Del Carmen Miranda DuroAnnet Ten BrugMaría del Carmen Olmos-GómezAnnett FrickMaria Del Carmen Ortega-NavasAnnette Madelene DăncilăMaria del Carmen Pérez LópezAnnette PfahlbergMaría Del Carmen Reyes-RodríguezAnnette RoggeMaría Del Carmen Rodríguez GarcíaAnneza YiallourouMaría del Carmen Terol CanteroAnnibal Parracho SantannaMaría del Carmen Valls MartínezAnnick Parent-LamarcheMaría Del Carmen Vargas-GarcíaAnnie DuchesneMaría Del Consuelo Díez-BedmarAnnie GendronMaría Del Mar BernabéAnnie George-PuskarMaría Del Mar Carlos-AlberolaAnnika C. MontagMaría Del Mar Fernández-ÁlvarezAnnika Helgadóttir DavidsenMaría Del Mar Fernández-MartínezAnnika KäckMaría del Mar Jiménez LasserrotteAnnika KünneMaría Del Mar Molero JuradoAnnis StensonMaría Del Mar Ramírez FernándezAnn-Marie PendrillMaría del Mar Sánchez-FuentesAnnunziata Esposito AmideoMaría del Mar Simón MárquezAnnunziata NuscaMaria Del Pilar IngunzaAnoop RawatMaría Del Pilar Salas-ZárateAnoop SheshadriMaría Del Rosario Navalón-GarcíaAnqi DengMaria DemetriouAnrzej PelcMaria DermikiAns VercammenMaria DietrichAnselm JordaMaria Do Carmo SobralAnselme MuzirafutiMaria Do Céu MarquesAnselmo MoriscotMaria Do Rosário MartinsAnshul SrivastavaMaria Do Rosário PintoAnson Chui Yan TangMaría Dolores Arguisuelas-MartínezAnte RađaMaria Dolores De-Juan VigarayAnteo Di NapoliMaría Dolores Díaz-NogueraAnthi OikonomouMaría Dolores Granado CastroAnthony AlbergMaría Dolores Guerra-MartínAnthony ChampionMaría Dolores López FrancoAnthony D. RedmondMaría Dolores Marrodán SerranoAnthony FirekMaría Dolores Martínez AiresAnthony K. WaruruMaría Dolores Ruiz FernándezAnthony KondrackiMaría Dolores Trigo AzaAnthony LamannaMaria Donata OrfeiAnthony LehmannMaria Dos Anjos DixeAnthony LynnMaria Dulce EstêvãoAnthony M. BeanMaria Dyah KurniasariAnthony O’HareMaria DzikućAnthony R. LupoMaria Economou-EliopoulosAnthony SuraceMaria Elena BontempiAnthony T. RederMaria Elena Cuartero-CastañerAnthony ValverdeMaria Elena Garcia RuizAntigoni ZafirakouMaria Elena NenniAntje Büttner-TeleagăMaría Elena Pérez-LópezAntje MissbachMaría Elena Rodrigo ClaveroAntje RisiusMaria Elisa CrestoniAntje TannenMaria Emilia GarbelliAnto ČartolovniMaría Emilia SantillánAntoaneta EneMaria Enrica BettinelliAntoine DalibardMaría Esther García BuadesAntoine LebloisMaría Esther Irigoyen-CamachoAntoine NaemMaría Estrella-GonzálezAntoine RaberinMaria Eugenia Martin PalacioAnton FicaiMaria Evarista Arellano-GarciaAntón García-MartínezMaria ExindariAnton IsaacsMaria FaulknerAnton Ming-Zhi GaoMaría Ferández-MartínezAnton MoritzMaría Fernanda CabréAnton MurzinMaría Fernanda Montenegro-LandívarAnton PetukhovMaria Fernanda Reyes RodriguezAnton PyzhevMaria Fernanda Soares NaufelAnton R. KiselevMaría Fernández CabezasAnton S. TsybkoMaria FerraraAnton StraubMaria FioreAnton TerasmaaMaria FlorenceAnton TyurinMaria FortofoiuAntonela MatanaMaria Francesca MilazzoAntonella AgodiMaria Francesca PiazzaAntonella ArghittuMaria Francisca Colella-SantosAntonella BevilacquaMaria Gabriella CampoloAntonella BodiniMaria Gabriella MelchiorreAntonella CaccamoMaria Gabriella VersoAntonella CecchettoMaria GacekAntonella De RomaMaria GavriilakiAntonella GuarinoMaría Gema Cid ExpósitoAntonella LopezMaría Gemma AlbendínAntonella RosiMaria GeorgiadiAntonella TassoneMaria GeorgiadouAntonella VastolaMaria GhitaAntonella ZizzaMaria Gil UlldemolinsAntoni BorrásMaria Giovanna ConfettoAntoni Martínez BallestéMaria Giulia MariniAntoni MorralMaria Giuseppina BartoloAntoni NiedzielskiMaria Giuseppina PetruzzelliAntoni WontorczykMaría Gloria Gallego-JiménezAntonia Baeza CaracenaMaria Gloria RossettiAntonia ErrazurizMaría González Cano CaballeroAntonia FeolaMaria Graciela Hernández Y OrduñaAntonia KaltsatouMaria GrajdianAntonia KondouMaría Granados-SantiagoAntonia M. CaleyaMaria Grazia CagettiAntonia MarsdenMaria Grazia Lourdes MonacoAntonia OdagiuMaria Grazia PiancinoAntonia PelegrínMaria Grazia PiccioniAntonia ResslerMaria Grazia PorporaAntonia SiogaMaria Grodzka-ŁukaszewskaAntonia SorgeMaria GrzelakAntonia YpsilantiMaría Guadalupe Frías-De-LeónAntonietta CosentinoMaria HełdakAntonietta IvonaMaria Helena SantosAntonija TadinMaria Hermínia DiasAntonije OnjiaMaria Hofman-BergholmAntonina ArgoMaria Idoia Ugarte-GurruxagaAntonina IvanovaMaria IliadouAntonina KaczorowskaMaria Inês Fernandes PimentelAntonina ShumakovaMaria Inês Rodrigues LobatoAntonino BiundoMaria Irene BelliniAntonino FiorentinoMaría Isabel Amor AlmedinaAntonino ManiaciMaría Isabel Carretero AbellánAntonino RomeoMaria Isabel Dias MarquesAntonino SpanuMaría Isabel Lamas GaldoAntónio AbreuMaría Isabel Mármol-LópezAntonio AlbuquerqueMaría Isabel Parra ArévaloAntonio Alejandro Lorca-MarínMaria Isabel Tomás-RodríguezAntonio Alvarez-SousaMaría Isabel Tovar-GálvezAntonio AmmendoliaMaria J. Casanueva-MarencoAntonio AngeloMaría J. GunnarsdóttirAntonio ApicellaMaría J. Morales-GázquezAntonio AquinoMaría J. Navas-MartínezAntonio AsciutoMaria JakubikAntonio AzaraMaria JanickaAntonio Bernal-GuerreroMaría Jesús Caurcel CaraAntonio BiondiMaría Jesús Delgado RodríguezAntonio BovoliniMaría Jesús FernándezAntónio CaleiroMaría Jesús HerreroAntonio Cano-OrellanaMaría Jesús Núñez-IglesiasAntonio CárdenasMaría Jesús Rodríguez-GarcíaAntonio Cardesa-SalzmannMaria Jesus Vinolo GilAntónio CardosoMaria João CebolaAntónio Carrizo MoreiraMaria João RamalhosaAntonio Cartón-LlorenteMaria João SousaAntonio ChiarenzaMaria João VelezAntonio CicchellaMaria JohannAntonio CoboMaria Jose Aliaño-GonzálezAntonio Córdoba FernándezMaría José Baeza-RiveraAntonio CorselloMaría José Barbé-VillarubiaAntonio CorteseMaría José Bautista-LlamasAntonio CristaldiMaría José BendekAntonio Daniel García RojasMaría José CasañAntonio Del GiudiceMaria José D. MartinsAntonio Diogo Silva VieiraMaria José De Oliveira SantosAntônio Domingos PadulaMaría José García-IglesiasAntónio Duarte SantosMaría José García-PolaAntonio DuranMaría José Ginzo VillamayorAntonio E. Aquino JuniorMaría José Hernández SerranoAntonio F. Rodríguez-HernándezMaría José LeraAntonio FernandesMaria José MadeiraAntonio Fernández ParadasMaría José Molina-GarridoAntonio Fernández ParraMaría José Muñoz-AlférezAntonio G. LentoorMaria José RosaAntonio GidaroMaría José Suárez-LópezAntonio Granero-GallegosMaria José VaradinovAntonio GrecoMaría Julia Ajejas BazánAntonio GuaitaMaria JustineAntonio Guerrero EspejoMaria KaczmarekAntonio Hernandes Chaves-NetoMaria KampezaAntonio J. Carcas-SansuánMaria KantereAntonio Jesus Mendoza-FernándezMaria Klonowska-MatyniaAntonio Jesús Molina FernándezMaria KocórAntonio José Carpio de los PinosMaria Kola-BezkaAntónio José Madeira NogueiraMaria KoroziAntónio José OsórioMaria KózkaAntonio Jose Ramos SilvaMaría Laura BoskoAntonio León García-IzquierdoMaria Laura MancaAntonio LloretMaria Laura SchilliròAntónio LopesMaria Laura TandaAntónio Luís AmaralMaria LavdanitiAntonio Luis Martínez PujalteMaria Leonor Abrantes PiresAntonio Luis Moreno-AlbarracínMaria Leonor SilvaAntonio M. Burgos-MolinaMaría Leticia Meseguer-SantamaríaAntonio ManentiMaria LianouAntónio Manuel Neves VicenteMaria LindholmAntonio Mario BulfamanteMaria LivanouAntonio Martínez RayaMaría Lourdes Gutiérrez-CarrilloAntonio Martínez-SabaterMaria Lousada-FerreiraAntonio Mendes-SousaMaria LucaAntonio Miguel Linares LujánMaria Lúcia PatoAntonio MincioneMaría Luisa de la Hoz TorresAntonio Molina-CarballoMaria Luisa Di MartinoAntonio MolinoMaria Luisa GennariAntónio MonteiroMaría Luisa Gonzalez-ElenaAntonio Moreno-PoyatoMaria Luisa IndianaAntonio Muñoz-GarcíaMaria Luisa MartinoAntonio Muñoz-LlerenaMaría Luisa Ojeda MurilloAntonio NarzisiMaria Luisa PedditziAntonio NesticòMaria Luísa Pereira SoaresAntonio NettiMaría Luisa Rico GómezAntonio Olivas-MartinezMaria Luisa Rodero-CosanoAntonio PacificoMaría Luisa Rodicio-GarcíaAntonio Palacios-GarciaMaría Luisa Zagalaz SánchezAntonio Peña-FernándezMaría Luisade Lázaroy TorresAntonio Pérez GonzálezMaria ŁukaszekAntonio Pérez-RuedaMaría Luz Sanchez-SanchezAntonio PetošićMaría M. Morales Suárez-VarelaAntonio RaffoneMaria MaccaroneAntonio Ramos MartinezMaría Maciá AndreuAntonio Ranchal SánchezMaria Maddalena MarrapodiAntonio RaschiMaría Magán-MagantoAntonio Roberto ZamunerMaria Magdalena Bujnowska-FedakAntonio Rodero SerranoMaria MalliarouAntonio Rodríguez FuentesMaria Malvina TsamouriAntonio RomanelliMaría Manuela Hernández-HerreroAntonio RosadoMaria Marcella LaganaAntonio Sánchez-PatoMaria MarinescuAntonio SantagostiniMaria MarinoAntonio SantanielloMaria MarkakiAntonio Sarasa CabezueloMaría Martínez-OlcinaAntonio Sarria-SantameraMaria MartinoAntonio SchiattarellaMaria MatsangidouAntonio Seijas-MaciasMaria MatveevaAntonio SermekMaria Melo AlmiñanaAntonio Silva Menezes JuniorMaría Membrive-JiménezAntonio Simone LaganàMaría Mendoza-MuñozAntonio SiniscalchiMaria MenshakovaAntonio SousaMaría Mercedes Díaz SomoanoAntonio Stabelini NetoMaría Mercedes Vázquez VílchezAntonio SteccoMaria Michela ChiarelloAntonio TacconeMaria Mihaela GrajdianAntonio TessitoreMaria Miklasińska-MajdanikAntonio TintoriMaría MoralesAntonio Torralba-BurrialMaría Moral-MoralAntonio ValentiMaria MorgeseAntonio VarrialeMaría NapalAntonio Victor Campos CoelhoMaría Nelia Soto-RuízAntonio Víctor Martín-GarcíaMaria NicastriAntonio Videira Da SilvaMaria Nicoleta TurliucAntonio ZayasMaría Nieves Pérez MarfilAntonio ZumelzuMaria NishevaAntonio-Jesús Marín-PazMaria NóbregaAntonios KonstantarasMaria NyholmAntonios PouliosMaría Orosia Lucha-LópezAntonis PeppasMaria P. BonasoniAntover SarmentoMaria PachesAntreas KantarosMaria PaçoAnts-Hannes ViiraMaria Paola BertuccioAntti TalonenMaria Paola GattiAntum A. PanjwaniMaria PapadakakiAntun BilošMaria PapadoliopoulouAnu Anna GeorgeMaria PapanikouAnu AsnaaniMaria PaparoupaAnu PolvinenMaria ParkerAnudeepa SharmaMaria PassanteAnugrah Sabdono SudarsonoMaría Patricia Hernández-MitreAnuj K. ChandelMaría Paz Aguilar-CaballosAnuja PandeMaria Paz García-CaroAnuli NjokuMaria PereiraAnup BastolaMaria Pia MaraghiniAnupam DasMaria Pia RiccioAnupam MukherjeeMaria Pia SammartinoAnuradha BhukelMaria Pietronilla PennaAnuradha JainMaría Pilar Aparicio-FloresAnurag KumarMaría Pilar ArrueboAnurag MisraMaría Pilar MatudAnusha AngajalaMaría Pilar Salguero-AlcañizAnushree PriyadarshiniMaria PilgunAnusna ChakrabortyMaría Piñeiro-IglesiasAnveshkumar NellaMaría Piñeros-LeañoAnwar A. SayedMaria PortarapilloAnwar UsmanMaria QuestaAnwen ShaoMaria RagostaAny RufaedahMaria RajskaAnying SongMaría Ramos-MonserratAnyu BaoMaria Raquel Marçal NataliAnže JapeljMaria Raquel Ventura LucasAnzhelika VoroshilovaMaria Rella RiccardiAo LiMaria RicciAo ShiMaria RicciardiAobo WangMaria Rosa Suñer SolerAoyong LiMaria Rosaria TumoloAparajita JaiswalMaria Rosaria VarìAparecido SouzaMaría RouraAparna ShankarMaría Rúa-AlonsoApinun KanpiengjaiMaria Salvina SignorelliApolinaras ZaborskisMaría Sánchez ZafraApostolos KarantanasMaría Sanz RemachaApostolos PapagiannakisMaria SarapultsevaAppel MahmudMaria ScaturroApurvakumar PandyaMaria Sebastiana SilvaAqeel M. AlenaziMaria SerraAqib Hassan Ali KhanMaría SilvaAraceli Escudeiro RossignoliMaria SkrzyszowskaAraceli Ortiz RubioMaria SofologiAras BozkurtMaria Sole FacioniArastoo Pour BiazarMaría Soledad Prats MoyaAraxi BalianMaria SomarakiArbaz SajjadMaría Sotelo PérezArcelina MarquesMaria Stefania SpagnuoloArchana MishraMaria Stella EpifanioArchana RaoMaria SterzyńskaArchontissa KanavakiMaria StrenacikovaArdalan ShariatMaria SuciuArdeshir BazrkarMaria SymeonakiAre Hugo PrippMaria SzargutArefin ShezanMaria SztachelskaArely Anaya-HernandezMaria T. BulzacchelliArely Vergara-CastañedaMaria T. PontoAremis Villalobos HernándezMaria T. Van HartenArezki IzriMária Takácsné HájosArfan GhaniMaria Tarico Bico IsabelArfan ShahzadMaria TatarusanuArgero ZerrMaria Teresa Barreiros Caetano TomásArghya DattaMaría Teresa Costado DiosArgyro MavrogiorgouMaria Teresa Dos SantosAriadna Angulo-BrunetMaria Teresa Ferreira MoreiraAriadna Hernaiz-SanchezMaría Teresa García OrdásAriadne Cristiane Cabral CruzMaría Teresa García-NietoAriana Anamaria CordosMaría Teresa García-SanzArianna AntonucciMaria Teresa GiglioArianna FonsatiMaria Teresa GuagnanoAriel E. TurciosMaria Teresa HerdeiroAriel FrajermanMaría Teresa Iglesias LópezAriel Soares TelesMaria Teresa InzitariArielle DeutschMaria Teresa Nestares PleguezueloArie-Willem De LeeuwMaria Teresa RestivoArif LuqmanMaría Teresa Solís-SotoArif RezaMaria Tereza Pepe RazzoliniArif Sabta AjiMaría Tomé-FernándezArifin HidayatMaria TomprouAriful HaqueMaria Torres VegaArijit NathMaria TotanArijit SenMaría Trinidad Sánchez NúñezArindam IndraMaria UkhanovaAristea GioxariMaria UrbaniecAristeidis BloutsosMaría V. EstellerAristeidis MertzanisMaría Vanessa Rodríguez-CornejoAristidis A. GaliatsatosMaria VaradinovAristidis MatsoukisMaria VasiloglouAristomenis ExadaktylosMaría Vázquez-GuimaraensAritra GhoshMaría Verónica BaezAriuna TaivanMaría Victoria De GálvezAriuntuul GaridkhuuMaría Victoria Del Barrio GándaraArja RimpeläMaría Victoria Ruiz-MallorquíArjan VissinkMaría Videgain-MarcoArjun R. SomanathanMaria VitielloArjun SenguptaMaria WakolbingerArkadiusz ArtyszakMaria ZakynthinakiArkadiusz DziedzicMaria ZangãoArkadiusz GendekMaria Zelinda RomanoArkadiusz HulewiczMaria Zuba-CiszewskaArkadiusz JanuszewskiMaria-Anca MaicanArkadiusz M. KowalskiMaria-Angelica BenczeArkadiusz NędzarekMaría-Arántzazu Ruescas-NicolauArkadiusz SadowskiMaría-Carmen Muñoz-DíazArkadiusz StanulaMaría-Carmen RicoyArkadiusz SzarekMariachiara CarestiaArkadiusz UrbanekMaria-Cleofé ZaragozáArkadiusz ZurawskiMaria-Crina RaduArkady KotlyarMaría-del-Mar Alonso-AlmeidaArkaitz CastañedaMariaelena TagliabueArkaitz MucientesMaria-Floriana PopescuArkalgud RamaprasadMariagabriella PuglieseArkers Kwan Ching WongMariagiovanna CantoneArlener D. TurnerMariagrazia D’EmilioArlette J. Ngoubene-AtiokyMaría-isabel OrtegaArlind MaraMaria-Jimena Mucino-BermejoArlind ReuterMaria-Jose Marquez-BallesterosArman Hashemi MonfaredMariam SetapaArman HoseinpurMarian BotchwayArmand CholewkaMarian Catalin VoicaArmand FaganelMarian EvansArmand KasztelanMarián Fernández-VillarinoArmando A. Rayas-AmorMarian FlisArmando AliuMarian Henryk LewandowskiArmando CalogeroMarian MroziewskiArmando CaseiroMarian NiezgodaArmel Zacharie Ekoa BessaMarián Pérez-MarínArmelle-Myriane NgueleuMarian Pompiliu CristescuArmin EsmaeilzadehMarian ProorocuArmin GemperliMarián RuckiArmin JentschMarian SimkaArmin Niklas FlinspachMarian Sorin PaveliuArmin RajabiMarian WoźniakArminda CarvalhoMariana Arias AvilaArmon TamateaMariana BondrescuArnaud AlvesMariana Buranelo EgeaArnaud PagesMariana CărămidăArne EideMariana Cernicova-BucaArne Martin JakobsenMariana Conte GrandArne SpeerforckMariana FloriaArnoldo De HoyosMariana G. FigueiroArnulfo Hernan Nava-ZavalaMariana Lopes De SousaArnulfo Ramos-JiménezMariana MagalhãesAroa Tardáguila-GarcíaMariana MocanuAron P. SherryMariana NicolaeAron SzekelyMariana PetrovaÁron TörökMariana Ramona CiolacÁrpád ÁmbrusMariana VerasArpan Man SainjuMariana VuțăArpit GuptaMariangela ManciniArpita GhoshMariangela PuciArpita RaiMariangela SpagnoliArrigo CiceroMariann GyöngyösiArryn A. GuyMariann HarangiArsene MekinianMarianna BellafioreArsenio PaezMarianna GiunchiArshad Arjunan NairMarianna K. TheodoraArshad JamalMarianna KulkovaArslaan KhalidMarianna MazzaArtan HoxhaMarianna MuravyevaArtem BelousovMarianna RaczykArtem MetlinMarianna TosatoArtemio Carrillo-ParraMarianne HollymanArthi VeerasamyMarianne KollerArthit PhosriMarianne MarkowskiArthur BacaMarianne SkovArthur BlaserMariano Amo SalasArthur FerreiraMariano CingolaniArthur FrankMariano FosArthur I. PetrovMariano GactoArthur KopylovMariano González GarcíaArthur L. BrodyMariano González-PérezArthur L. CantosMariano Meseguer De PedroArthur L. FrankMariano ScaglioneArthur WagnerMariantonietta FrancavillaArtur BadydaMaría-Pilar Molina-TorresArtur BanachMariarita BrancaccioArtur BoháčMariarosaria SimeoneArtur GalimovMaría-Yolanda González-AlonsoArtur GiełdońMaribel PinoArtur GonçalvesMarica CassarinoArtur HołujMaricarmen Leandra Lecuna TovarArtur IlukMaricel SantosArtur KruszewskiMarie BlackmoreArtur LemińskiMarie C. ConwayArtur NiedzielskiMarie ChalbotArtur PędziwiatrMarie ElfArtur StruzikMarie FlynnArtūras RazbadauskasMarie FriedelArturo DurazoMarie G. GantzArturo Edgar Zenteno-GalindoMarie HabitoArturo GonzalezMarie Herrmann-LuneckeArturo González GilMarie Luisa SchaperArturo LópezMarie MadsenArturo Realyvasquez VargasMarie MardalAruliah RajasekarMarie MurphyArulselvi SubramanianMarie NakladalovaArun Kumar Hanumana Gouda PatilMarie OzanneArun L. W. BokdeMarie P. BoltzArun Lal SrivastavMarie SjögrenArun RajasekaranMarie ThomasAruna V. SarmaMarie-José H. E. GijsbertsArunachalam MuthiahMarie-Josée FleuryArunaksharan NarayanankuttyMarieke DirksenArũnas RamanavičiusMarieke Joan Gerda Van HeuvelenArunava GhoshMariekie GerickeArunee AhantarigMariela CorralesArunima SikdarMariela PadillaArunkumar RanganathanMariella AquilinoArup ChakrabortyMarielle BrinkmanArve E. AsbjørnsenMarien GrahamArvin Haj-mirzaianMarie-Noelle DuquenneArvind DhakaMarie-Pierre FlamentArvind NuneMarie-Pierre TalovacciAryasree SudharajMarietta Van Der LindenÅsa EkMarife B. AnunciadoÅsa KneckMariia ErmilovaAsad KhanMariia RizunAsaf AchironMarija BurinskienėAsaf BilgoryMarija DrobnjakovićAsal Kamanl FardMarija GalicAsanka BulathwattaMarija JevticAsbjørn ÅrøenMarija KutlesicAscensión Palomares RuizMarija M. BožićAsel RyskulovaMarija MargušAsen GrytsaiMarija OpačakAsep K. SupriatnaMarija Radmilović-RadjenovićAsfandyar KhanMarija StjepanovićAsghar Afshar JahanshahiMarija VilotijevićAsghar AliMarija-Magdalena PetrinovicAsghar JahanshahiMarijan JakovljevićAsghar TalbalaghiMarijan ŽuraAsher FawwadMarijana Despotović-ZrakićAshesha MechineniMarijana MajdakAshfaque Ahmed ChowdhuryMarijana Marek SarićAshim GuptaMarijana PetkovićAshir JaveedMarijana VučinićAshis MondalMarijke TrappenburgAshish BadiyeMarijn PoortvlietAshish JainMarijn SpeeckaertAshish NoronhaMarika DemersAshkan MemariMarika MassaroAshley HazelMarika SyrrouAshley McAllisterMariko KikutaniAshley NgMariko MiyazakiAshley SchermanMarilena MangiardiAshley TovarMarilena StoianAshokkumar ThirunavukkarasuMarilene Brandão Tenório FragosoAshraf AliMarília Santos AndradeAshraf DewanMariliza NikolaidouAshraf El-BindaryMarilou OuelletAshraf KariminikMarilyn J. HammerAshril YusofMarin DarsieAshtyn ArealMarin SenilaAshu KediaMarin UgrinaAshu RastogiMarina AlbaneseAshuin Kammar-GarcíaMarina Amorim SousaAshutosh Dhar DwivediMarina BobkovaAshutosh SharmaMarina Cervera Alonso de MedinaAshwani KumarMarina ClericoAsier Arcos-AlonsoMarina Consuelo VitaleAsier Santas TorresMarina EfthymiouAsif IqbalMarina EmmanouilAsif KhaliqMarina Evrard MichelAsif M. HuqMarina FrontasyevaAsif QureshiMarina GaldeanoAsif ShajahanMarina GravitAsif SukriMarina Greghi SticcaAsil Ali ÖzdoǧruMarina GregoricAsima ShahMarina GulyaevaAsimina KouriatiMarina KostićAsle HoffartMarina KurnikovaAsli TelliMarina Letica CrepuljaAslina BaharumMarina M. VillalbaAsma BukhariMarina MaffoniAsma SghaierMarina Mat BakiAsmaa Mohammed HassanMarina MisciosciaAspasia E. SerdariMarina Nikolić TopalovićAspasia GoulaMarina NosikAsrat AmnieMarina PiscopoAsri MaharaniMarina RezendeAssed HaddadMarina RigilloAssunta TaverniseMarina Robas MoraAsta SavanevičienėMarina Sánchez-RicoAsterios KampourasMarina SartiniAsterios PatsiaourasMarina Soley-BoriAstrid-Marie De SouzaMarina TumoloAsunción Martínez MayoralMarina Umaschi BersAswatha Ram H.nMarina V. BurachevskayaAswi AswiMarina VasiljevaAta TaraMarina Villalón-MirAtef KhalilMarina Villanueva-PazAtef M. K. NassarMarina VukojeAtefeh AbediniMarina ZeldovichAtefeh AmerizadehMarinela IntaAtefeh VaeziMarinka Mravak-StipetićAthanasia ChatzipanteliMarino VilovićAthanasia K. TolkouMario A. LeivaAthanasia PapazafiropoulouMario Amatria JiménezAthanasia PrintzaMario Bernardo-FilhoAthanasia ProklouMario CaggianoAthanasia XirogianniMario Cruz GonzalezAthanasios Akis TheofilatosMario De FeliceAthanasios DalamitrosMario Di GioacchinoAthanasios DamialisMario Enrico CanonicoAthanasios GalanisMario Enrique Rojas-RussellAthanasios KampasMario FargnoliAthanasios KiourtisMario FernándezAthanasios KolovelonisMario FranchiniAthanasios KoutrasMário Gabriel Santiago dos SantosAthanasios N. TsartsalisMario GómezAthanasios TheofilatosMario J. Valladares-GarridoAthanasios TriantafyllouMário José Baptista FrancoAthanassios BissasMario José Costa De MacedoAthanassios TselebisMario Josue Valladares-GarridoAtheesha SinghMario LourençoAthena C. Y. ChanMario Luca MorieriAthena ProgiouMario LucianoAthicha UttajugMario MarendićAthikhun SuwannakhanMario Martínez PizarroAthos TrecrociMario MiniatiAtiah H. AlmalkiMario MontaninoAtif JahangerMario NavarroAtif Maqbool KhanMario Nikola MužekAtiphan PimkhaokhamMario NjavroAtiqa AmbreenMario Roberto Dal PozAtique-ur RehmanMario SafranAtiyeh AbdallahMario ŠporčićAtsuhiko MatsunagaMario SprovieriAtsuhiko OtaMário ToméAtsushi HIjikataMário VazAtsushi NagaiMario VerdicchioAtsushi SakumaMario Vincenzo RussoAtsushi SugamaMario Wedneyde Lima MoreiraAtsushi SugiuraMarioara IleaAtta UllahMariola BorowskaAttayeb MohsenMariola DrozdAtte Von WrightMariola GłowackaAttila János TrájerMariola JaniszewskaAttila KissMariola PawlaczykAttila OláhMariola PiłatowskaAttilio Fiorini MorosiniMariola Zielińska-BłajetAttinà AldaMarion Christine LantériAtul DhallMarion FelderAtul PandeyMarion HutinelAtul PranayMarion PanizzonAubrey E. JonesMarion SlackAubrey JonesMariona RiusAubrey L. GalushaMarios AntonakakisAudil RashidMarios GoudasAuditya Purwandini SutartoMarios RossidesAudra De WittMarios SpanakisAudrey GuenicheMaris KalinkaAudrey J. MurrellMarisa CarvalhoAudrey Opoku-AcheampongMarisa CorreiaAugust WrotekMarisa CruzAugustine Chuck ArizeMarisa FilipeAugusto AlvimMarisa GranatoAugusto AriasMarisa GuillénAuliya Abdurrohim SuwantikaMarisa PapalucaAung Zaw PhyoMaristela NepomucenoAura Emanuela DomilMaristella GussoniAura GabrielMarit Kristine HelgesenAura MihaiMarit SkogstadAura RuscaMaritta S. JaakkolaAura RusuMaritza PintadoÀurea Cartanyà-HuesoMarius BaranauskasAurea Ramírez-JiménezMarius ConstantinÁurea RodriguesMarius L. CrainaAurel BurciuMarius PislaruAurel MaximMarius Sorin DincăAurel NireșteanMárius Vicent Fuentes I. FerrerAurel PeraMarius-Cristian PanăAurelia OstrowskaMariusz AdamskiAurelian-Petrus PlopeanuMariusz Adynkiewicz-PiragasAurélie Van HoyeMariusz DrużbickiAurélie WagenerMariusz DuplagaAurélien DeniaudMariusz DzieńkowskiAurelija BlaževičienėMariusz FinkielszteinAurelio ArnedilloMariusz GujskiAurelio LunaMariusz JabłońskiAurelio OlmedillaMariusz KostrzewskiAureliusz KosendiakMariusz PtakAurora Castro-MendezMariusz RogulskiAurora Rosa-MasegosaMariusz RudyAušra KazlauskienėMariusz RzętałaAustin M. WilliamsMariusz SikoraAutumn MarshallMariusz SojkaAvcı Dilek KuşaslanMariusz StasiolekAvgis HadjipapasMariusz SzóstakAvi MagidMariusz SzymanowskiAviad Tur-SinaiMariusz TichoniukAvik ChatterjeeMariusz TomaniakAvik DuttaMariusz WysokińskiAvinash PremrajMariusz ZielińskiAvinash R. PatwardhanMariya A. SmetaninaAvipsa RoyMariya Konsulova-BakalovaAvraham ShotanMarja Silén-LipponenAwadh YadavMarja TiilikainenAwadhesh AryaMarja-Liisa LaitalaAwadhesh KumarMarjan DrukkerAwais JabbarMarjan SalariAxel SteigerMarjan Van Den AkkerAya YokoiMarjeta TerčeljAyako EdahiroMarjo BuitelaarAyako MatsudaMarjorie C. McCullaghAyako MoritaMark AbelAyako NakaneMárk Ádám AntalAyanka WijayawardenaMark AldersonAyataka FujimotoMark AttridgeAyaz KhanMark BaronAydan E. UrvaylıoğluMark BrimbleAydin Bordbar-KhiabaniMark BrommeyerAydın BüyüksaraçMark BrownAydin EresenMark ChenAyelet Zlotogorski-HurvitzMark Ching-Pong PooAyesha FahimMark D. SimmsAyesha Rahman AhmedMark D. WhitakerAygul KissalMark E. HartmanAyham AlkhachroumMark F. SommerfeldtAyla BilginMark F. WiserAyla YagdiranMark FelixAyman MahmoudMark G. LebwohlAyman RagabMark GoldAymé PinoMark HansAynaz LotfataMark HenricksonAyodeji Emmanuel IyandaMark J. WilsonAyodeji Olalekan AwobamiseMark Johann KingAyon ChakrabortyMark K. TimmonsAyse Giz GulnermanMark LedbetterAysegül AksanMark MalisaAysegul BaltaciMark PalmerAyşegül ErdoğanMark PaulyAysegul NalcaMark QuiggAyu Krishna YuliawatiMark R. WilliamsonAyuga-Téllez EsperanzaMark ReiserAyumi AraiMark Robin ArmstrongAyumi SugiuraMark S. FleisherAy-Woan PanMark SibagAyyanar SivananthamMark StemmlerAyyoob SharifiMark W. LuskAyyub A. PatelMark WalshAzad HaiderMark Wansbrough-JonesAzad R. BhuiyanMark WardAzadé AzadMark WillemsAzadeh LakMarkku TykkyläinenAzalia Avila-NavaMarko BaškovićAzam AslaniMarko D. M. StojanovićAzhar AbbasMarko DjapanAziah DaudMarko JoksimovićAzian Azamimi AbdullahMarko KumrićAzidah Abdul KadirMarko LucijanićAzita FarashiMarko MatulinAzizuddin MuhammadMarko PerićAziz-Ur-Rahim BachaMarko R. CincovićAzlin AhmadMarko StojanovićAzmawati NawiMarkus BredeAzmi M. DarwazehMarkus CanazeiAzumi IshizakiMarkus ChristinerAzwidowi LukhwareniMarkus FeserAzza El-HousseinyMarkus HepptB. N. Pavan KumarMarkus Hil­anderB. P. NaveenMarkus HoffmannBabafela AwosileMarkus KleinBabak AghelMarkus M. KnodelBabak ChehroudiMarkus PuchingerBabak J. FardMarkus RiesBabak NematMarkus StorvikBabett RamsauerMarkus StraußBabul PrasadMarkus TannheimerBach Q. HoMarla Andréia Garcia De AvilaBach TranMarleide Da Mota GomesBachir KassasMarlena BroncelBadar UmarMarlena RobakowskaBadiah BaharinMarlene CervantesBagus Setya RintyarnaMarlene LopesBagus Tris AtmajaMarlene LoureiroBahareh Ghane MotlaghMarlene PenzBahareh MansoorianMarlies E. C. ElfrinkBahman Jabbarian AmiriMarloes Dekker NitertBahman RamavandiMarloes HendrickxBahram ChoubinMarlon De Castro VasconcelosBai ZhongfeiMarlous FockerBakhtiar FeizizadehMarlyn BennettBala S. C. KoritalaMarni ManegreBalachandar VellingiriMarnie DobsonBalaji SadhasivamMaroof AlamBalakrishnan DeepanrajMarousa KouvelaBalamurugan RamatchandirinMarquis HawkinsBalan RathakrishnanMarshall ChasinBălănică Carmelia DragomirMarszowski RyszardBalaraman RajanMarta AbelhaBalasubramani Subramani ParanthamanMarta Aires-de-SousaBalasubramanian KarthickMarta AlcaideBalasubramanian ViswanathanMarta AltieriBalawanthrao JadhavMarta Amor-BarbosaBalazs FeherMarta Arroyo IzagaBalazs HankoMarta BonBalázs LemmerMarta Borowska-StefańskaBalázs P. SzabóMarta BrásBalázs ZsirkaMarta CavigliaBálint CsonkaMarta De SantisBalkrishna Eknath NarkhedeMarta DominiBallambhattu Vishnu BhatMarta Doval MiñarroBalram AmbadeMarta DziechciarzBaltasar González-AntaMarta Elena Losa-IglesiasBáltica CabiesesMarta Evelia Aparicio GarcíaBamidele RaheemMarta FijałkowskaBan A. MajeedMarta GaburjákováBang-Hoon ChoMarta García-RomeraBangwal DeepakMarta GimunováBang-Yuan ChenMarta GomàBanhi BiswasMarta GrossBanshi SabooMarta Gwiaździska-GorajBanu MüjdecıMarta HabánováBao HongyanMárta HockBao JiaMarta IscarBaojian ZhuMarta JaskulakBaoliang PengMarta Joanna Szandruk-BenderBao-Peng LiuMarta Kandziora-CiupaBaravan AsaadMarta KepinskaBárbara AngelMarta KiragaBarbara BaranowskaMarta KopańskaBarbara BarunMarta KożybskaBarbara BergierMarta KrasnyBarbara BlundellMarta KsiazczykBarbara CaciMarta LaskaBarbara CalabreseMarta Lisiak-ZielińskaBarbara CardoneMarta Makara-StudzińskaBarbara CharbotelMarta MaleszaBarbara CieślińskaMarta Malinowska-CieślikBarbara Czech-SzczapaMarta María Hernández MartínBarbara D’AmenMarta MarszałekBarbara D’AvanzoMarta MartinBarbara De GraaffMarta MasztalewiczBarbara De RosaMarta MatosBarbara ErminiMarta MazurBarbara FenesiMarta MelisBarbara ForresiMarta MuszalikBarbara FraczekMarta NalejBarbara FutaMarta Nowacka-ChmielewskaBarbara GherriMarta OpalińskaBarbara GołębiewskaMarta Parzeniecka-JaworskaBárbara GonzalezMarta Pereira AlvesBarbara GordonMarta PieczaraBarbara GrabinskaMarta PlichtaBarbara HanfstinglMarta RachelBarbara J. RyeMarta Rodríguez-HernándezBarbara J. TurnerMarta RoratBarbara JankowiakMarta RusekBarbara JasiewiczMarta RusnakBarbara KiełczawaMarta RybskaBarbara KlikMarta RzadkiewiczBarbara KosMarta Sainz GómezBarbara KozielskaMarta SajdakowskaBarbara LapinskaMarta Seiz PuyueloBarbara LondMarta SeratiBárbara Luque SalasMarta Serra PermanyerBarbara MavroidiMarta Sofía López RodríguezBarbara MognettiMarta ŠpakulováBarbara Nieradko-IwanickaMarta StanisławskaBarbara Novotná-BřezovskáMarta Stojić MitrovićBarbara PavlakovičMarta Sylvia Del Rio GuerraBarbara PawłowskaMarta SzkielaBarbara PenolazziMarta TalaveraBarbara PetracciMarta TomczykBarbara PieczykolanMarta Tomczyńska-MlekoBarbara PieperMarta TremoladaBarbara RemberkMarta VasconcelosBarbara RosołekMarta Waliszewska-ProsółBarbara RuffinoMarta WiśniewskaBarbara Ruszkowska-CiastekMarta Woldańska-OkońskaBarbara SaltzmanMarta ZnajmieckaBarbara SchilloMarta ZubiaurBarbara Schoepp-CothenetMarta-Marika UrbanikBarbara ŚlusarskaMarte Karoline Råberg KjøllesdalBarbara StalmachováMartha Alejandra Morales-SánchezBarbara SymanowiczMartha B. BairdBarbara SzymoniukMartha Dettl-RiveraBarbara Waldon-RudzionekMartha DriessnackBarbara WieliczkoMartha Graciela Ruiz-GutiérrezBarbara WiewióraMartha Idalí Saboyá-DíazBarbara ŻarskaMartha J. M. WellsBarbora StrouhalováMartha Krogh TopperzerBarclay T. StewartMartha LegorretaBarış Ethem SüzekMartha RhoadesBarkat KhanMarthias SilwambaBarkha YadavMarti LindseyBarliz WaissengrinMartijn J. J. FinkenBarrak AlahmadMartín A. MaraschioBarry H. RosenMartín AgrestBarry Lee ReynoldsMartín Aubert Hernández CalzadaBart PijlsMartin BalzanBart RuttemanMartin BeerBart VisserMartin Bjørn StausholmBartłomiej GlinaMartin BohleBartlomiej GorskiMartin ChanBartlomiej IglinskiMartin Christian MichelBartłomiej JefmańskiMartin DaumillerBartłomiej PtaszekMartin David RoseBartłomiej SkowrońskiMartin DodmanBartłomiej SoltysikMartin E. G. BlohmBartłomiej WośMartin EbnerBartłomiej ZieniukMartin EichlerBartolome Piza MirMartin F. ShermanBartolomé Pizà-MirMartin FiederBartosz CzarneckiMartin Gomez UllateBartosz CzubaMartin HenselerBartosz JaweckiMartín Hernandez-MarínBartosz JóźwikMartin HoltmannBartosz KruszewskiMartin Joh. MeyerBartosz ŁamaszMartin KendraBartosz M. WalczakMartin KomarcBartosz MalkiewiczMartin KraftBartosz PędzińskiMartin KralikBartosz RymkiewiczMartin LavallièreBartosz SawikMartin LehnertBartosz SzelągMartin LeníčekBartosz SzulczyńskiMartin MaierBartosz WielgomasMartin MotolaBartosz WilczyńskiMartin MüllerBartoszczuk PawelMartín Pérez-SantosBarun Kumar ThakurMartin PienkowskiBasanta Kumar BiswalMartin PlayfordBaseer U. AhmadMartin R. KaneBashir AdelodunMartin RootBasil KahwashMartin Sanchez-GomezBasil MathewMartin SchepelmannBasilio PueoMartin ScheuchBaskar MitraMartin SchmidtBaskaran ChandrasekaranMartin SchultzeBasma Al-AnsariMartin TauschmannBasma EllahiMartin TsuiBassam ElgamoudiMartin VasilevBassma ElwakilMartin WassenBastian MesterMartina BergantBasu Dev PandeyMartina BlaskovaBasudeb MondalMartina BlaškováBathrinath SankaranarayananMartina BosoneBatista PaulaMartina CaramentiBato KoracMartina ChiriacòBayda Atiya KalafMartina Dal BelloBayodé Roméo AdégbitèMartina DebiasiBayrakdar Muhammed EnesMartina FerićBayram CeylanMartina GažarováBayu Satria WiratamaMartina HedvicakovaBchir BrahimMartina KleberBeat GöpfertMartina KlucakovaBeata Bieszk-StolorzMartina Maria MensiBeata BucheltMartina MichaelisBeata BystrowskaMartina MorandoBeata DobrowolskaMartina NordhBeata DrużyńskaMartina OlceseBeata FerenczMartina OzbičBeata Fornal-PieniakMartina PavlíkováBeata Galińska-SkokMartina RagettliBeata Gierczak-KorzeniowskaMartina SoptaBeata HoffmannMartine H. HollanderBeata HornikMartine HamannBeata Hukowska-SzematowiczMartine HennequinBeata HysaMartine VallarinoBeata Jaworska-SzulcMartins AdelaideBeata Katarzyna PiętaMartti VastamäkiBeata ŁubiankaMartu SilviaBeata M. Gruber–BzuraMartyna Kasper-JędrzejewskaBeata MessyaszMaruniak-Chudek IwonaBeata MianowskaMarusia Rentería-VillalobosBeata MrugalskaMaruti Ram GudavalliBeáta NovotnáMarvin EdeasBeata NowakMarvin PowellBeata PawlowskaMarwa A. A. FayedBeata PiȩtaMarwa ElgendyBeata RaszkaMarwa EmamBeata RybojadMarwa GaberBeata Sarecka-HujarMarwa MadiBeata Skowron-MielnikMarwa MajdiBeáta SoltészMarwan Al-MomaniBeata SperkowskaMary A. FoxBeata SwieckaMary Ann EricksonBeata SzluzMary Ann JarvisBeata Szulc-MusiołMary BarkerBeata TarnackaMary Beth HolmesBeata Zatwarnicka-MaduraMary C. SheehanBeata Zofia FilipiakMary DavisBeate RitzMary DereshiwskyBeate VajenMary Etta MorrisBeatrice Adriana BalgiuMary HearstBeatrice AlbanesiMary HigginsBeatrice BiasiniMary HrywnaBeatrice CantoniMary J. KastenBeatrice FemminellaMary Jo IozzioBéatrice FerversMary JoyceBeatrice Gabriela IoanMary K. ButwinBeatrice HeimMary KamienskiBeatrice KuhlmannMary Katherine HoyBeatrice MoroniMary Kay O’RourkeBeatrice ThielmannMary L. WhiteBeatrice VincentiMary LashleyBeatrice VolelMary LynchBeatriz Álvarez-EmbarbaMary McDonnell-NaughtonBeatriz Arranz-MartínMary McKennaBeatriz Cabañas GalanMary N. WoessnerBeatriz Cuesta-BriandMary NorvalBeatriz FebreroMary O’ReillyBeatriz Gómez-MartínMary P. CorcoranBeatriz Helena TessMary RodriguezBeatriz LimaMary Schubauer-BeriganBeatriz Maria Bermejo-GilMary SheehanBeatriz Martinez-PascualMary VachonBeatriz MinghelliMary WhiteBeatriz Núñez AnguloMary YanBeatriz PereiraMary YannakouliaBeatriz PérezMary ZhangBeatriz Rodriguez-RocaMaryam AbarghoueinejadBeatríz Rodriguez-SanchezMaryam ArdalanBeatriz Sánchez-SánchezMaryam BaniasadBeatriz Servilha BrocchiMaryam BoshtamBeau BezaMaryam FakhariBeau MeyerMaryam GharachehBebiana CondeMaryam HasanpourBecca FeldmanMaryam K. Mohammadi-AraghBeckley IkhajiagbeMaryam MalekigorjiBecky D. ClarksonMaryam RahmatiBecky TalynMaryam RanjbarBedřich MoldanMaryam SaffarihaBee Kang TanMaryam SamimiBeenish Moalla ChaudhryMaryam TofangchihaBegoña Acha PiñeroMaryam VasefiBegoña EspejoMaryam Yazdan MehrBegoña GuiraoMaryani Mohamed RohaniBegoña Perez-FernandezMaryann D’AlessandroBegoña Polonio-LópezMaryland PaoBegümhan TurhanMarzena CzarneckaBehdad ShadidiMarzena GibczyńskaBehnam GhasemzadehMarzena Jeżewska-ZychowiczBehnam SadeghiMarzena KurpinskaBehnam VafadariMarzena SuchockaBehrooz GharleghiMarzena Szostakiewicz-HołowniaBehrouz BehnamMarzena WiernickaBehrouz M. Nezafat MaldonadoMarzena Wińska-KrysiakBehrus PuladiMarzia CottiniBehúnová AnnamáriaMarzia Di DonatoBehzad EsmaeiliMarzia DulalBehzad HeibatiMarzia MorenaBehzad IravaniMarzia SegùBehzad KianiMarzouk SheriefBehzad VaferiMarzulli MicheleBekir KocazeybekMaša BuljacBelen Alonso OrtizMaša Filipovič HrastBelén Callejón LeblicMaša PintaričBelén DerquMasa SasagawaBelén Díaz-PulidoMaša Skelin KlemenBelén López-FelicesMaša Vukčević MarkovićBelgin AkınMaša ŽdralevićBelinda HornbyMasaaki KurasakiBelinda WheatonMasafumi NodaBellu LuisaMasahiko SatohBen Abdallah IsmailMasahiro BannoBen BeagleholeMasahiro KawaharaBen CaveMasahiro KitamuraBen ClaytonMasahiro OginoBen CoetzeeMasahiro SaitoBen GardnerMasahiro SakataBen GrayMasahito FushimiBen HoldenMasahito HitosugiBen MorrisMasahito KatsukiBen SchwambergerMasakazu HirotaBen WooliscroftMasakazu MinetamaBen ZhangMasakazu TanakaBence Tamás SzabóMasako ShomuraBenedetta MarmiroliMasami MatsumuraBenedetta RagniMasamichi KamihiraBenedetto BarabinoMasamitsu IchihashiBenedetto LongoMasanao YokohiraBenedetto TerraciniMasanobu KiiBenedict FrancisMasanobu OkayamaBenedict L. AdamsMasanori MatsuiBenedict SheehyMasanori MurakamiBenedikt EggerMasao OtaBenedito C. PrezotoMasao YamaguchiBeni Gomez-ZunigaMasaru YamaguchiBeniamin Oskar GrabarekMasashi SekineBenítez Sillero Juan de DiosMasataka SuwaBenito León del BarcoMasatsugu OruiBenito Soto-BlancoMasayoshi MatsuiBenjamin AbbottMasayoshi TanishitaBenjamin AnsaMasayuki OkudaBenjamin BeckerMasayuki OzakiBenjamin C. BlountMasayuki SakakibaraBenjamin ChrisingerMasayuki TakadaBenjamin CullMasayuki TakigawaBenjamin Dowd-ArrowMasfer AlkahtaniBenjamin E. GarfieldMasfueh RazaliBenjamin E. Nilsson-PayantMasindi MphaphathiBenjamin George JacobMaslin MasromBenjamin HolfelderMasoud AmiriBenjamin L. SmarrMasoud BijaniBenjamin MakepeaceMasoud DaraBenjamin O. LaddMasoud KhosravipourBenjamin PutoisMasoud Nazarian-SamaniBenjamin SilverbergMasoud RahmatiBenjamin SpringgateMasoud YazdanpanahBenjamin Valentin BeckerMasoumeh Kourosh AramiBenjamin WebbMass Hareeza AliBenjamin WoolfMassimiliano AmmirabileBennett AmaechiMassimiliano BarattucciBenno KrachlerMassimiliano CapezzaliBenoît BernardMassimiliano ConsonBenoit JobinMassimiliano DonatiBenoît MissetMassimiliano EspositoBenoit MorelMassimiliano MangoneBentayeb NaïmaMassimiliano Matteo PellegriniBente KnollMassimiliano PanellaBente Sparboe-NilsenMassimo AndrettaBeomyoung ChoMassimo BonacchiBerber NemanjaMassimo BracciBerceste GülerMassimo ChelloBergita GanseMassimo CorradiBeril AltunMassimo CrescimbeneBerislav BolfekMassimo De MartinisBerislav ŽmukMassimo Del FabbroBerkeley KaiteMassimo Di GraziaBerna Haktanirlar UlutasMassimo IngrassiaBerna RahiMassimo LanzaBernadeta ChyrchelMassimo LazzariBernadette FloodMassimo MaccagnoBernadette JonesMassimo MarielloBernadette P. MarriottMassimo MartinelliBernard BarasaMassimo MontisciBernard LacazeMassimo MorettiBernard NaughtonMassimo MusacchioBernard Sinclair-DésgagnéMassimo ReconditiBernard SpeculandMassimo TusconiBernard SurialMassomeh HajileeBernardas VaznonisMassoomeh Hedayati MarzbaliBernardo GozziniMassoud MoslehpourBernardo IdeMassy MutumbaBernardo Martins RochaMasud RabbaniBernardo Nugroho YahyaMasudul ImtiazBernardo StutzMat Amin AmizaBernardo VegaMat DalgleishBernd W. SiguschMáté ZöldyBernd WallnerMatea BelanBernhard BogertsMatea Zajc PetranovićBernhard LangerMateescu CarmenBernhard RauchMatej FikeBernhard StürmerMatej ParBernhard UehlekeMatej ŠtuhecBerta CsabaMatej TomasBerta Garcia-FernandezMatej VajdaBerta Paz LouridoMateja LorberBerta SchnettlerMateja OžanićBerta Vall CastellóMateo López-MoralBertina KreshpajMateus Coelho SilvaBertram KuchMateus Dias AntunesBert-Ram SahMateus F. CarneiroBertrand DautzenbergMateus R. AmorimBertrand LeboucheMateusz BabickiBertrand PorroMateusz CiskiBertrand TchanaMateusz GrajekBerwin Singh Swami VethaMateusz HämmerlingBessem MkaouerMateusz IlbaBeth A. LanningMateusz JakubiakBeth BrodskyMateusz JankiewiczBeth G. McManisMateusz JankowskiBeth JonesMateusz KuklaBeth Mozolic-StauntonMateusz ŁucBeth ParksMateusz NowakBeth ResnickMateusz PaligaBeth Rose MiddletonMateusz RozmiarekBethany D. WilliamsMateusz SpiewakBethany FoxMateusz TomanekBethany SimmondsMateusz WoźniakBethany SussmanMateusz ZarebaBethesda O’ConnellMatevz PogacnikBetsy JosephMatheus B. SoaresBettina BlaumeiserMatheus BertanhaBetty BurstonMatheus KoengkanBetty C. TonuiMathew Albert Wei Ting LimBetty DoreMathew J. GregoskiBetty ExintarisMathew ParackalBetty McGuireMathew Thomas GilBeverley HaleMathias LudererBeverly FitzpatrickMathias TrempBeverly HoganMathieu DeflemBeverly MayMathieu Di MiceliBeverly RoskosMathieu NedelecBeytullah KaradayiMathijs LucassenBeyza Ünalan DeğirmenciMathilde Body-MalapelBhagavathi Sundaram SivamaruthiMathilde MunierBhagwat RimalMathilde SengoelgeBhajan SinghMati PääsukeBhakti BasuMatías González HernándezBhaskar NarjaryMatias Monsalves-AlvarezBhaven ModhaMatias MorinBhavuk GargMatias NollBhawna DevMatias TagleBhekumuzi GumbiMatija LandekićBheshem RamlalMatilda Anna FrickBhikhari TharuMatilde Díaz HernándezBhim Sen ThapaMatina GhasemiBhishma TyagiMatiss PalsBhoktear Mahbub KhanMatjaz PercBhola PradhanMatjaž ŠramlBhoomika MathurMatluba KhanBhoomika PatelMatonickjn Kepcija RenataBhubaneswor DhakalMats ErikssonBhuvanesh Sukhlal KalalMats JongBiagio RaponeMats KüssnerBiagio SantellaMats SjöströmBiagio SolarinoMatt AsareBianca EdisonMatt Coward-GibbsBianca HanganuMatt De LisiBianca Ioana TodorMatt MarlowBianca L. GarnerMatt PetersBianca Manuela SanduMatt SpickBianca Maria Serena InguscioMatteo AiminiBianca MiarkaMatteo Alessio ChiappediBianca TescasiuMatteo BolcatoBianca WerneckeMatteo Bruno LodiBiao LiMatteo CarpiBiao YuMatteo ClementeBiaoan ShanMatteo Cotta RamusinoBibhuti B. DasMatteo CrippaBibhuti K. SarMatteo Della PortaBibian Van Der VoornMatteo FabrisBibudha ParasarMatteo GarauBice AvalloneMatteo InnocentiBidhan C. BandyopadhyayMatteo LandoniBidisha RoyMatteo LucchiniBidur DevkotaMatteo Luigi Giuseppe LeoniBijan AbadiMatteo MalvezziBiju SomanMatteo MartiBikarma SinghMatteo Monzio CompagnoniBilal Ahamad ParayMatteo MoroniBilal AkbarMatteo NardinBilal KhalidMatteo NioiBiljana CvetkovićMatteo PaciniBiljana KurtovićMatteo PonzoniBiljana PopeskaMatteo RattiBilly BrocatoMatteo RenzulliBilly T. W. YuMatteo RiccòBilsen BilgiliMatteo RipaBimal K. PaulMatteo SaccoBimal Kanti PaulMatteo ScopettiBimala PantheeMatteo SeranoBin CaoMattero RuggeriBin ChenMatthäus S. SiebenhoferBin HuangMatthes HaraldBin LianMatthew AllsopBin LiuMatthew ArduinoBin XueMatthew ArmstrongBinaya SapkotaMatthew BaconBinbin LuMatthew BarnesBindu JosephMatthew BeerseBing HeMatthew BourkeBing KuangMatthew ChrismanBing WangMatthew CollischonBing XiaMatthew CookBing ZhangMatthew D. HickeyBingbing ZhouMatthew DonaduBingchun ZhaoMatthew EastinBingjie Liu-LastresMatthew FarrowBingjun WanMatthew ForemanBinglu WuMatthew GreeneBingwen Eugene FanMatthew H. ZimmermanBingxian XieMatthew HarrisonBingxin ZhaoMatthew HirschtrittBingxuan LinMatthew J. DelmonicoBinod AcharyaMatthew J. RegulskiBinu Kandathilparambil SasiMatthew LeeBinyan WangMatthew LeschBipin Kumar AcharyaMatthew Matsaganis MatsaganisBiplab DattaMatthew McGrailBiraj Kanti MondalMatthew NelsonBiresaw AberaMatthew R. SloggyBirgit BabitschMatthew R. X. DentithBirgit HabedankMatthew SmithBirgit ThomsonMatthew SparkeBirgitta Dresp-LangleyMatthew SpringhamBirgitta KerstisMatthew StevensBirgitta WiitavaaraMatthew TaylorBirgitte LerbækMatthew Ting Chi LiuBirol AkbaşMatthew WichrowskiBishnupriya SahooMatthew WildcatBiswajit MaharathiMatthew WilfongBiswajit SarkarMatthias An Der HeidenBiswajita PradhanMatthias BeckBiswanath MahantyMatthias BelauBjoern SeitzMatthias BethgeBjørn E. HolsteinMatthias GriederBjörn KlingeMatthias KapischkeBjörn SlaugMatthias KolibabkaBlanca De-la-Cruz-TorresMatthias KrapfBlanca Estela Bernal EscotoMatthias LukasczikBlanca Estela Pelcastre-VillafuerteMatthias NiedrigBlanca Lumbreras LacarraMatthias RaspeBlanca Prieto-CallejeroMatthias RippBlanca R. LópezMatthieu GuemannBlanca Rosa García RiveraMatthieu LenoirBlanca TejeroMattia BrancaBlanca TorresMattia CappellettiBlanka KlímováMattia FrascioBlaž LikozarMattia MiottoBłażej CieślikMattia TerzaghiBłażej KudłakMattias PaulssonBlažo T. LalevićMatúš MišíkBlecharz JanMaulik C. KotechaBlessing J. Akombi-InyangMaulik VyasBlesson Mathew VargheseMaura A. E. PilottiBo BurströmMaura CallieraBo ChenMaura GallettaBo Hyon YunMaura M. E. PilottiBo JiangMaura MacPheeBo LiuMaureen BernerBo Pieter Johannes AndreeMaureen LongBo WangMaureen MacNamaraBo WengMaureen MakamaBo XiaMaurice J. LyverBo XuMaurice MarsBo YuMaurice YollesBo YuanMaurício Lamano FerreiraBo ZhaiMaurício Schüler NinBo ZhangMauricio Zapata-CuartasBob FriedmanMaurizio AmbrosiniBob GunierMaurizio BossuBob HodgeMaurizio EliaBob IvesMaurizio Giuseppe AbrignaniBob MulderMaurizio MarchesiniBoban M. ErovicMaurizio MauriBoban MelovćMaurizio NordioBochao ZhaoMaurizio PascadopoliBochiuan SuMaurizio PiergiovanniBogdan AntonescuMaurizio SabbatiniBogdan BatkoMaurizio SimmacoBogdan C. FloreaMaurizio TiepoloBogdan IancuMauro BelluardoBogdan IliescuMauro CancianBogdan MocanMauro CeccantiBogdan NadoluMauro Cesar Palmeira VilarBogdan SamojedenMauro F. PereiraBogdan Severus GasparMauro FilippiniBogdan SkwarzecMauro FinicelliBogdan SlencuMauro GiudiciBogdan TiganoaiaMauro GiuffrèBoggarapu Praphulla ChandraMauro Henrique Nogueira Guimaraes de AbreuBogna Grygiel-GorniakMauro LizotBogna LudwigMauro ManiscalcoBogusław TwarógMauro MartinelliBogusława LachowskaMauro MarzoratiBogusława UrbaniakMauro MigliavaccaBogusz Aksak-WąsMauro RaposoBohdan RożnowskiMauro RomanelliBohuš LeitnerMavromatis TheodorosBojan MasanovicMax Carlos Ramírez-SotoBojan ObrenovicMax MaurinBojan PajicMax O. StephensonBojana BajićMax SarmetBojana KneževićMaxim FilimonovBokai ZhuMaxime JeanjeanBolem Sai ChandanaMaximilian HeumannBolin YuMáximo Bernabeu-WittelBong-Ju ParkMaxine MoweBonnie FreemanMaxwell DalabaBonnie J. FurzerMaxwell Fordjour Antwi-AfariBonnie YimMay EbaidBoowook KimMay Ee PngBoran SekerogluMay Kristine Jonson CarlonBoris Apolo-MasacheMay Lei MeiBoris De RuyterMay Portuguez CastroBoris Filipović-GrčićMaya CormanBoris Gabriel JohnsonMaya KaganBoris GolmanMaya KylénBoris KozlovMaya MascarenhasBoris UlitinMaya RubtsovaBorja Herrero De La ParteMaya TsfatiBorja Matías PompaMayank ChoubeyBor-Jiunn WenMayank PatelBoro ŠtrumbeljMaynara Fernanda Carvalho BarretoBorut PoharMayra B. C. MaymoneBosena Tebeje GashawMayra Urrea-SolanoBoshra ArnoutMaysaa NemerBoštjan ŠumakMayssam NehmeBota-Avram CristinaMaytee ZambranoBotir KhaitovMayumi NishiBoubié GuelMayur B. KaleBowei ZhuMayur DokeBoyan GrigorovMayuresh Sunil PardeshiBoyen HuangMayya UspenskayaBo-Young HongMazapuspavina Md-YasinBo-Young JungMazen OmerBožana Lončar-BrzakMazhar Hussain MalikBożena BabiarzMazhar Javed AwanBožena Ćurko-CofekMaziar ShabestariBożena Czarkowska-PączekMaziar YazdaniBożena GullaMazin MohammedBożena KukfiszMazyar Ghadiri NejadBożena MroczekMd Abdul AwalBozena PeraMd Abdul HannanBożena Targońska-StępniakMd Abdul WazedBrach PostonMd Abdus SoburBrad JokischMd Aminul IslamBrad SionMd Anwarul Azim MajumderBradford K. BergesMd Ashraful AlamBradley JorgensenMd Ashraful IslamBradley P. AnderMd Asrul Nasid MasromBradley TatarMd Azharul IslamBrady S. De CoutoMd Azijul IslamBrahim BrahmiMd Hafiz IqbalBrahim SelmaouiMd Hakimul HaqueBrahmaputra MarjadiMd Irteja IslamBrajesh SinghMd Lamiur RaihanBralind KiriMd Mahfujur RahmanBran Barral BucetaMd Mahidur Rahman SarkerBranden BornMd MohiuddinBrando Ortiz-SaavedraMd Monirul IslamBrandon EgglestonMd Mostafizur RahmanBrandon J. BeddingfieldMd Moyazzem HossainBrandon KohMd Muzammel HossainBrandy-Joe MillironMd NadiruzzamanBranislav GlišićMd Nazim UzzamanBranislav ŠarkanMd NekmahmudBranislava Srđenović-ČonićMd Nuralam HossainBranko AnđićMd Rabiul IslamBranko LukićMd Rezaul IslamBraulio Henrique Magnani BrancoMd Saiful BariBrenda BogaertMd Sanower HossainBrenda Cabrera-MendozaMd ShafiquzzmaanBrenda Chaves Coelho LeiteMd Shahjahan KabirBrenda CustodioMd Shakhawat HossainBrenda GroenMd SharkerBrenda Sarahi Cervantes-LunaMd Shiblur RahamanBrenda SealsMd Siddikur RahmanBrendan HydeMd Sofiqul IslamBrendan MajorMd Tanjin AminBrenden HeidrichMd Tanvir RahmanBrennan BeanMdutshekelwa C. NdlovuBrent Dean RobbinsMeda-Lavinia NegrutiuBrent PetersonMeden F. Isaac-LamBrent S. JacksonMeei Tyng ChaiBrent W. WeyersMeenakshi AhluwaliaBrett BlighMeenakshi ThakurBrett ToelleMeer M. ChisthiBrian BellMeerambika MishraBrian BossakMegan A. QuinnBrian D. PooleMegan Christine Hetherington-RauthBrian FildesMegan DiazBrian GodmanMegan M. WennerBrian GreeleyMegan M. ZeringueBrian HoweMegan McCoyBrian J. PiperMegan NelsonBrian JonesMegan ParkerBrian KristensenMegan QuinnBrian Kwok Hong LawMegan RiddinBrian LawtonMegha SatpathyBrian LaytheMeghan E. MartzBrian M. HendricksMeghana DesaiBrian MillarMeghashyam BhatBrian SchumacherMeghit Boumedienne KhaledBrian SchwartzMeghna GuptaBrian SlobodaMehdi AsghariBrian SwannMehdi AzaouziBrian T. PavilonisMehdi BahramiBrian WeirMehdi JahangiriBrian WitrickMehdi NateghpourBriana BombanaMehdi TorkmahallehBrianna LarsenMehmet A. OrhanBrianna Le BusqueMehmet Bahri SaydamBridget HutchensMehmet Ergün HatirBridget K. BiggsMehmet ErmanBridget MeltonMehmet FıratBridgitte SwalesMehmet KocaogluBridie S. ThompsonMehmet M. AltintasBright Isaac IkhenaodeMehmet SarierBrigita MiežienėMehmet YildizBrigitte BootheMehmet YilmazBrisa N. SánchezMehrab NodehiBritni L. AyersMehrdad RafiepourgatabiBritni R. BelcherMehrez El-NagarBritt HallingbergMehri Karimi-DehkordiBritt SkaathunMehrnaz ShoushtarianBritt WrayMehrnoosh GhadimiBritta A. LarsenMei Lan FangBrittan ScalesMei Ling LimBrittanie Atteberry-AshMei Shan LiewBrittany BloodhartMei TruebaBrittany E. BlanchardMei-Chen LinBrittany HauptMei-Chi HsuBrittany K. TaylorMeifang LiBrittany MurphyMei-Hua YuanBritteny HowellMeiji Soe AungBritt-isabelle BergMeike RombachBritton BrewerMeike StrekerBroder BrecklingMeilinda MaghfirohBronisława Skrzep-PoloczekMeimei LaiBronwyn ThompsonMeine Van NoordwijkBrook BelayMeir LotanBrooke HansenMeixue DongBruce B. WallaceMeiyi MaBruce Baer ArnoldMekalathur Roja RamanBruce E. WinstonMeksianis NdiiBruce JansonMelania BalasBruce Jeffrey RogersMelania BoitorBruce LanphearMelania MaglioBruce M. WilsonMelanie Anne KneenBruce SpittleMelanie BoeckmannBruce SunderlandMelanie BoltzmannBruce W. CraigMelanie CraneBruce WinstonMelanie HawkinsBruna Coelho LopesMelanie Joslyn WoodfieldBruna DenizMelanie KorbeliusBruna EibelMelanie SchubertBruna SinjariMelanie SpeckBruno BalbiMelika MohammadkhahBruno Barbosa SousaMelinda CsimaBruno BonnechèreMelinda KolcsárBruno Bueno-SilvaMelinda KrebszBruno De Freitas CamiloMelinda LaituriBruno DonatucciMelinda MetaxasBruno FabianoMelinda PénzesBruno FerreiraMelinda S. Butsch KovacicBruno Ferreira CostaMelinda SzekelyBruno FranceschettiMeline KevorkianBruno G. LoosMeline Rossetto Kron-RodriguesBruno GonçalvesMeLisa CreamerBruno José Nievas SorianoMelissa Agsalda-GarciaBruno LanfrancoMelissa CareyBruno M. FonsecaMelissa D. StockbridgeBruno MagalhãesMelissa De ReggeBruno MarquesMelissa GladstoneBruno MezêncioMelissa GrahamBruno Morgado FerreiraMelissa MacKayBruno P. GuidioMelissa MarianaBruno RossaroMelissa MeadeBruno VendittoMelissa MeloughBruno ZoricMelissa MerryBruria AdiniMelissa O’DonnellBryan D. HujsakMelissa PartykaBryan NelsonMelissa PetrakisBryan R. BrownMelissa RittenhouseBryan WongMelissa S. JonesBryane MichaelMelissa S. W. YamauchiBryanne BellovaryMelissa SchumacherBrygida KlemensMelissa SingletaryBrygida KnyszMelissa SuterBud CooperMelitta McNarryBuddhi PushpawelaMelkie Getnet TadesseBudi AzhariMellisa A. HallBuhari DoğanMelody AlmrothBukola G. OlutolaMelody E. Morton NinomiyaBukola UsidameMeltem Ezgi DurgunBulat RamazanovMenachem NahirBulat SoktoevMenderes KabadayıBulbul SiddiqiMeng CaiBülent AkkayaMeng ChenBülent KilitMengbing DuBülent Okan MiçooğullarıMeng-Che TsaiBum-Jin ParkMeng-Cong ZhengBumjoon BaeMengge DuBuong-O ChunMenghau SungBurak BİROLMenglong QiuBurak BuldurMengmeng JiaBuratin KhampiratMengru ShihBurcu Akan EllisMengxi ShenBurghele Bety-DenissaMengxiao WangBurkhard WeisserMengxuan ZhangBuse Ozcan KahramanMengyao HanBushrah Binti BasironMengye ChenByamukama BenedictoMengyi XuByoung Joon KimMengyu LiByoung-eun YangMengyuan QiuByoung-Hee LeeMenkel-Meadow CarrieByoungwook AhnMercè Balasch-BernatByron IoannouMercede ErfanianByung Chang KwagMercedes AyusoByung Jun ChoMercedes BotijaByung Yong JeongMercedes Díaz-RodriguezByung-Hoon KimMercedes FernandezC. Cody BarteetMercedes Gaitán-AnguloC. M. Sabbir AhmedMercedes LovrecicCafer SakaMercedes Matás CastilloCagatay CebeciMercedes Salazar HernándezCagdas DedeogluMercedesz OrsosÇağla GürMeredith GartinÇağrı ErginMeredith PerryCahyo BudimanMerete Kolberg TennfjordCaijun YangMerlin Gountié DedzoCaio Eduardo DominguesMerryn ColeCaio SantosMert BaştaşCaio SarmentoMert GuneyCaiqing QinMerten NefsCaitlin ClarkeMervyn Márquez GómezCaitlin SynovecMesbahuddin ChowdhuryCaitlin VayroMeshal AlmoshaogehCaitlyn PlacekMetehan ImamogluCalastrini FrancescaMette L. BaranCaleb EstebanMevhibe B. HocaogluCaleb LeducMeysam BayatCaleb RasconMeysam KeshavarzCalero Morales SantiagoMeysam SalarijaziCălin CăinapMeysam VadiatiCalin CorciovaMi Kyoung SongCalin GhermanMi XiangCălin I. ProdanMi ZhouCalin VaidaMia FolkeCalla R. BrownMia StokmansCalli Tzani-PepelasiMian Mobeen ShaukatCallie LittleMian YangCallie Watkins LiuMiao Ling WangCalogero Lo DestroMiao YuCalvin ChengMiao-Chiu HungCalvin Lloyd ColeMiao-Ju HsuCalzada Mendoza Calzada-MendozaMiaw-Chwen LeeCamelia BotezanMicael DahlenCamelia Delia VoicuMichael A. FlynnCamelia Lucia DrăghiciMichael A. LongCamelia OroianMichael A. TaliasCamelia SoponaruMichael AlkanCamelia TruțaMichael AppellCameron DavidsonMichael AshCameron FarrowMichael BaigentCameron G. EstrichMichael BaiocchiCameron GordonMichael BenderCameron MuraMichael BöcherCameron NewtonMichael BoudillonCameron W. MacDonaldMichael CampbellCamila Aparecida BorgesMichael ChaitonCamila Borelli ZellerMichael ChapwanyaCamila Corrêa Matias PereiraMichael Christopher MelnychukCamilla CattaneoMichael ConnollyCamilla HansenMichael CopeCamilla KienastMichael CurranCamilla Kin Ming LoMichael D. AndersonCamilla MateraMichael De VitaCamilla PredaMichael DearyCamilla S. GrahamMichael DeschenesCamilla Wikström-GrotellMichael Di StefanoCamille A. ClareMichael DietrichCamille EsneauMichael DooleyCamilo J. Bastidas PachecoMichael DoursonCamlyn MasudaMichael DudleyCan DongMichael E. MillerCan GungorenMichael E. VaydaCandace E. GriffithMichael EnwereCandace R. FoxMichael F. ShaughnessyCandace RecenoMichael FleischerCândida MalçaMichael FratkinCândida SilvaMichael FroschCándido InglésMichael G. MaukCandy BrownMichael GerlichCandy Lim ChiuMichael HastCanel EkeMichael HermanussenCaner ÇakiMichael HieteCano-Ramírez HugoMichael HollifieldCao YangMichael InskipCaoimhe ClerkinMichael J. CarperCapai LisandruMichael J. LandramCara SymanzikMichael J. ParksCarel JansenMichael John DoughertyCaren J. FrostMichael John NortonCari BogulskiMichael Keith RussellCarin Staland-NymanMichael KizitoCarina BalcoşMichael KnitschkeCarina BerteröMichael KralCarina De Fátima RodriguesMichael KundiCarina Ferreira PinheiroMichael LazarCarina Lundh HagelinMichael LiebmanCarina Pinheiro-AraujoMichael MachiorlattiCarinne MagnagoMichael MathuraCarissa WengroviusMichael McVeyCarita HakanssonMichael N. KammerCarl BeckerMichael NaorCarl BorchgrevinkMichael NorbergCarl F. ReinhardtMichael OdellCarl FriskMichael OlsonCarl J. BassiMichael OlumekorCarl WeemsMichael P. CravenCarla Alexandra Martins FonteMichael PalaciosCarla BagnaMichael PentellaCarla BarrosMichael PickupCarla BergMichael PoteserCarla CandeiasMichael R. ChladekCarla CannizzaroMichael RigbyCarla CarvalhoMichael RovitoCarla CavalloMichael SachsCarla FontanaMichael SchirmerCarla Freijomil-VázquezMichael SchmidtCarla GamelasMichael SchrammCarla LimaMichael Silva-PeñaherreraCarla López-NuñezMichael T. BaglivioCarla MarelloMichael Timothy LaneCarla Maria Batista Ferreira PiresMichael TozeCarla MasalaMichael V. JoachimCarla Matos SilvaMichael W. BrandCarla MeurkMichael W. MehaffyCarla Oliveira HenriquesMichael WallerCarla Rolo AntunesMichael WangCarla Sílvia FernandesMichael WeinbergCarla Sofia FarinhaMichael WellsCarla Tarazona-MezaMichael WestCarla VidoniMichael WiblishauserCarla ZappullaMichael YoungCarles BarriocanalMichael ZhukovskyCarles BorregoMichaela GoodwinCarles Saus-OrtegaMichaela LiuccioCarli ZegersMichaela PagelCarlina BlackMichaela PettieCarlo Alberto ArtusiMichaela TirpákováCarlo Alberto MaroneseMichail KalogiannakisCarlo BieńkowskiMichail TheodosiadisCarlo BizMichail TonkonogiCarlo BravoMichail Zografakis SfakianakisCarlo BrognaMichał BaranCarlo CampanaMichał BembenekCarlo CernettiMichał BilczakCarlo CinqueMichal BonczykCarlo D’AscenziMichał BoreckiCarlo DindorfMichał BulcCarlo Federico Dall’OmoMichał CaputaCarlo Giacomo LeoMichal CehlarCarlo LaiMichał CupiałCarlo LajoloMichał CzaplaCarlo LibertoMichal DolezelCarlo M. BiancardiMichał DziadkiewiczCarlo MeloniMichał FiedlerCarlo NavarraMichal FranekCarlo PerisanoMichal GalambošCarlo Vittorio CitterioMichał GąsiorekCarlo ZivelonghiMichał GazdeckiCarlos A. M. CarvalhoMichał GinsztCarlos AbreuMichał GondekCarlos Alberto Martínez-HuitleMichał GontarzCarlos Alberto P. De OliveiraMichal HolubcikCarlos Alexandre Borges GarciaMichal JurášekCarlos Alfonso Tovilla-ZarateMichał Kazimierz ChojnickiCarlos AmenabarMichal KelemenCarlos ArdilaMichal KlichowskiCarlos Augusto Carvalho VasconcelosMichal KrawczykCarlos Augusto Kalva-FilhoMichal KudláčekCarlos AyánMichał KwiatkowskiCarlos Barbosa-TorresMichał LatalskiCarlos Berlanga MacíasMichal LavidorCarlos Bernal-UtreraMichal LewandowskiCarlos C. DuarteMichał ŁobaczCarlos CalderonMichał MarczakCarlos CarvalhaisMichal NapieralaCarlos CelisMichał NapierałaCarlos Cuenca-BarralesMichał NowakowskiCarlos CulquichiconMichał NowickiCarlos CunhaMichal OrdakCarlos Curvelo SantanaMichał PietkiewiczCarlos de las Heras-PedrosaMichal PinkCarlos Díaz-CaroMichal PiotrowskiCarlos DosilMichal PuškárCarlos E. VelascoMichał RabijewskiCarlos Eduardo Fonseca-AlvesMichał S. NowakCarlos Eduardo FontanaMichał SarulCarlos Eduardo Keutenedjian MadyMichał Seweryn KarbownikCarlos Eduardo RaymundoMichał SoliwodaCarlos Eduardo SiqueiraMichał SpiesznyCarlos Enrique Cuevas SuárezMichal StarczewskiCarlos Ferrás SextoMichał StopelCarlos FigueiredoMichał SzeremetaCarlos Fontes RibeiroMichał T. TomczakCarlos Frederico Barboza De SouzaMichal VágnerCarlos GarcíaMichal ValčoCarlos GravatoMichal VostrýCarlos Guedes SoaresMichał WendtCarlos Hernando GómezMichal WielechowskiCarlos Hervás-GómezMichał WiśniewskiCarlos Iván Delgado-NieblasMichał WoszczykCarlos Javier Esparza LópezMichal ZarobkiewiczCarlos Jesus Toro HuamanchumoMichał ŻemłaCarlos LaranjeiraMichał ŻorniakCarlos LiconMichał-Goran StanišićCarlos Llanes-ÁlvarezMichalina BłażkiewiczCarlos López-de-CelisMichalina IlskaCarlos López-GutiérrezMichel BrondaniCarlos M. ChangMichel JancloesCarlos M. ScavuzzoMichel MarinaCarlos MadeiraMichel P. GuilleminCarlos Manuel Torres AlmeidaMichel PérusseCarlos MarcuelloMichel WhiteCarlos Méndez-MartínezMichela BossaCarlos Mestanza-RamónMichela FranchiniCarlos Miguel MartoMichela MeregagliaCarlos Montero-CarreteroMichela MitchellCarlos MuñizMichela PerroneCarlos NoronhaMichela SalamoneCarlos O’Connor-ReinaMichelangelo LucianiCarlos OliveiraMichele BellingeriCarlos Osorio-SandovalMichele BertocciCarlos Peñarrubia-LozanoMichele BraggioCarlos Peña-SalazarMichele CarboneCarlos Pérez PlasenciaMichele CovelliCarlos Pérez-MonterMichele CovielloCarlos PfeifferMichele De MariaCarlos Pinto-GomesMichele Di CosolaCarlos Portugal-NunesMichele Di StefanoCarlos Rivera-GómezMichele FioreCarlos Roberto Hall BarbosaMichele FontefrancescoCarlos RodríguezMichele GiulianiCarlos RonceroMichele GobbiCarlos RosadoMichele GolinoCarlos Ruiz-NuñezMichele GuidaCarlos SainzMichele HannaCarlos Salavera BordásMichele L. MoohrCarlos Salgado-PascualMichele LanzaCarlos SampaioMichele MaglioneCarlos SequeiraMichele MarchioniCarlos Shu Kei LamMichele NavarraCarlos SoaresMichele PaolantonioCarlos Soubervielle-MontalvoMichele PergadiaCarlos TeixeiraMichele PietragallaCarlos TrenadoMichèle PreydeCarlos VasconcelosMichele RivaCarlos Vladimir Zambrano RodríguezMichele RoccaCarlos-Augusto Gonzalez-CorreaMichele SilvestroCarlotta CocchettiMichele StaianoCarlotta FossataroMichele TepedinoCarlotta PenoneMichel-Edwar MickaelCarly DaleyMichelle BanfieldCarly FletcherMichelle ButlerCarme GarciaMichelle Colder CarrasCarme Hervada SalaMichelle CullCarme SaurinaMichelle GarciaCarmel Houston-PriceMichelle JeongCarmela BravaccioMichelle L. MillerCarmela ComitoMichelle Lee D’AbundoCarmela Di LucianoMichelle M. C. TanCarmela Martinez-VispoMichelle MagidCarmela MentoMichelle MoosbruggerCarmela MustoMichelle MottolaCarmela RinaldiMichelle PrasuhnCarmelina CosmiMichelle Renée Umstattd MeyerCarmelo A. Juárez-CastellóMichelle RogersCarmelo CalìMichelle SahrCarmelo Ortega RodríguezMichelle SimonCarmelo SaranitiMichelle YoungCarmen BalanMichikazu NakaiCarmen BerenguerMichiko FurutaCarmen BlythMichio HongoCarmen Burgos VidelaMickael L. D. DerocheCarmen CantuariasMickey KeenanCarmen CasanovasMickey SperlichCarmen ChanMidori MatsushimaCarmen Clemente-JulMieczysław AdamowiczCarmen ConcertoMieke GoetschalckxCarmen Corujo-VélezMieszko WięckiewiczCarmen Daniela Quero CaleroMigle BacevicieneCarmen De KockMiguel A. FernándezCarmen Del Pilar Gallardo MontesMiguel A. García-MartínezCarmen Delgado ViñasMiguel A. SogorbCarmen Delgado-AlvarezMiguel AlcarazCarmen Dueñas ZambranaMiguel Alves PereiraCarmen DuseMiguel AmadoCarmen F. Navarro-PérezMiguel Ángel Albalá GenolCarmen Fernández EcheverríaMiguel Angel Alcántara-OrtigozaCarmen FigueroaMiguel Angel Álvarez-VázquezCarmen Gloria Aguayo-ArriagadaMiguel Ángel CarboneroCarmen Guzmán GarcíaMiguel Ángel CastellanosCarmen HerranzMiguel Ángel Castro VillamorCarmen Jiménez-EspinozaMiguel Ángel Fernández-BarreraCarmen L. A. M. Vleggeert-LankampMiguel Angel Fernandez-GraneroCarmen LizarragaMiguel Ángel Gallard-VigilCarmen LlenaMiguel Angel HerreraCarmen M. LeonMiguel Ángel López-SáezCarmen M. TylerMiguel Ángel Montero-AlonsoCarmen Maximiliana DobreaMiguel Angel Negrín-HernándezCarmen Mayolas-PiMiguel Ángel Pérez-SousaCarmen MínguezMiguel Angel Prieto LageCarmen Moret-TatayMiguel Ángel Rojo TiradoCarmen Nadia CiocoiuMiguel Angel SánchezCarmen P. Álvarez NietoMiguel Angel Serrano RosaCarmen Palmero-CamaraMiguel Ángel Solano-SánchezCarmen Patino-AlonsoMiguel Arjona RamírezCarmen Paula AlvarezMiguel CardosoCarmen Pérez-RodrigoMiguel Castelo-BrancoCarmen QuinteroMiguel CastroCarmen RochMiguel ClementeCarmen Rodriguez-BlazquezMiguel Equivias PadillaCarmen RubioMiguel FilipeCarmen SavinMiguel HuertaCarmen Suárez-SerranoMiguel LópezCarmen TaberneroMiguel MadrugaCarmen ToderaşcuMiguel Motas GuzmánCarmen Vizoso-GómezMiguel Pérez FernándezCarmen Zabala BañosMiguel Ramón Pecci-LloretCarmen ZahariaMiguel RebeloCarmen-Gabriela SirbuMiguel RicouCarmina Castellano-TejedorMiguel Santibáñez-AndradeCarminda MoraisMiguel Soares-OliveiraCarmine BuonocoreMiguel TerrosoCarmine FinelliMiguel Toribio-MateasCarmine IzzoMiha FošnaričCarmine MerolaMihael Emilov Tsalta-MladenovCarol BurnsMihaela CretuCarol C. McDonoughMihaela Denisa ComanCarol D. RyffMihaela GhermanCarol Freedman-DoanMihaela GhitaCarol LinMihaela HedeșiuCarol McKinstryMihaela IonicăCarol NashMihaela KardosCarol VlassoffMihaela KavranCarol Yen Chin LinMihaela Laura BratuCarole Christofk UpshurMihaela LunguCarole PélissierMihaela MocanCarolin KilianMihaela OnofreiCarolin MüllerMihaela PopaCarolin PhilippMihaela SimionescuCarolina Archundia HerreraMihai CraiuCarolina Blanco FontaoMihai Cristian OrzanCarolina Bringas MolledaMihai DinuCarolina FredesMIhai DumitruCarolina G. OjedaMihai FedorcaCarolina Guzman HolstMihai MarianCarolina Lagares-FrancoMihai MusteataCarolina LunettiMihai OlanescuCarolina MachadoMihai PopescuCarolina N. KeimMihai ZahariaCarolina P. RamôaMihail MitovCarolina Perez FerrerMihai-Teodor GeorgescuCarolina PimentelMihajlo JakovljevicCarolina Piña RamirezMihalj BakatorCarolina VieiraMihály KökényCarolina Vila-ChãMi-Heon RyuCaroline BarakatMihkel KaljurandCaroline E. BrettMihnea-Alexandru GămanCaroline HarveyMihyeon SeongCaroline LenetteMiihir Narayan MohantyCaroline RhéaumeMiika KujanpääCaroline ShorterMi-jung KwonCaroline SubraMika GisslerCaroll HermannMika Kondo KuniedaCarolyn AugustaMika OgawaCarolyn E. LandisMikael ForsmanCarolyn Gentle-GenittyMikaela DufurCarolyn M. CunninghamMikaela OwenCarolyn Melissa PayusMikalai FilonchykCarson PattersonMike DaubeCarsten TjellMike ThelwellCarsten WerginMikel Pérez GutiérrezCary PresantMikhail F. BorisenkovCaryn Van VredenMikhail KolevCasanova GeorgiaMikhail KozhevnikovCasey QuinnMikhail SeminCasper Glissmann NimMikhail Yegorovich SinelnikovCassandra ChaneyMikio IshiwatariCassandra E. HendersonMikko HärmäCassandra EngemanMikko NurminenCassandra JohnsonMiklós BánhidiCassandra MayaMiklós FagyasCassandra S. DiepMikołaj DąbrowskiCassiano RechMiku SodhiCassio Arthur WollmannMi-Kyoung ChoCassio WollmannMi-Kyoung JunCat KutayMikyoung LeeCataixa LópezMila A. EmeraldCătălin BerescuMila de Moura Behar Pontremoli SalcedoCatalin LazarMila KingsburyCatalin PopescuMila V. VlaskovskaCatalina González-FortezaMila VeselinovićCătălina Iulia SăveanuMilada KrejciCatalina López-MartínezMilagros Benito HernándezCatalina LucaMilagros Sánchez MayorCatalina MotofeiMilagrosa Borrallo-JimenezCatalina TudoseMilan AndrejićCatarina Duarte SantosMilan B. RadovanovićCatarina GodinhoMilan GnjatovićCatarina RuaMilan HusarCaterina BarioglioMilan KragovićCaterina BensiMilan KřápekCaterina CavicchiMilan KubiatkoCaterina CavigliaMilan KubinaCaterina Erika La CasciaMilan TešićCaterina FedeMilan TomaCaterina Francesca GuidiMilanka Gardašević-FilipovićCaterina GrassiMilda ALksneCaterina GuiotMilda PečiulienėCaterina SagnelliMilda PetrulevicieneCath JacksonMildred MaisonetCatharine Ward ThompsonMile BošnjakCatherine A. EganMilen DimitrovCatherine A. LabrenzMilena D. LakićevićCatherine AudrinMilena LopreiteCatherine ChanMilena P. IlićCatherine ChristieMilena PanićCatherine CooperMilena Santric MilicevicCatherine EllisMilena ŚciskalskaCatherine IpsenMilena TomanicCatherine JaureguiMilena VasicCatherine L. ZemanMilena VuckovicCatherine LabbéMilene Tavares FontesCatherine Makison BoothMiles SowdenCatherine Marquis-FavreMili DasCatherine N. KibirigeMilica JankovicCatherine PerzMilica Kašanin-GrubinCatherine Plüss-SuardMilica Pejovic-MilovancevicCatherine SimonMilica ZekovicCatherine SmithMilivoj J. DopsajCatherine TebaldiMiljan BigovićCatherine WallMiljana JovandaricCathleen L. DohertyMilka Bubalo-ŽivkovićCathrine ReineholmMilka DonchinCathryn SparksMilla ImmonenCathy BreenMilon IslamCathy L. ThomasMilos B. RadenkovicCathy O’MullanMiloš BožovićCathy UreMiloš HitkaCátia MagalhãesMiloš PoliakCátia MartinsMiloš RadosavljevićCatia NetoMilos VucicevicCátia SilvaMilosh HuberCatinca SecuianuMiloslava CernaCatriona BradleyMiłosław KozakCayetano Medina-MolinaMiłosz BabeckiCécile BétryMiłosz LewandowskiCecile JauzeinMilton Vieira JuniorCecile LepageMilva PepiCecilia Alegado JimenoMimi Tatlow-GoldenCecilia AriciMin ChenCecilia ChanMin Cheol ChangCecilia FridénMin Heui YooCecilia RogmarkMin J. KwakCecilia Simón RuedaMin Jae LeeCecília SzigetiMin YangCecilia VarjuMin Young KimCecy Martins SilvaMin ZhengCédric CarascoMina AzimiradCédrick T. BonnetMina Di MarinoCelestino Garcia-GomezMina JoCelestino SarduMina SamukawaCélia FreitasMinal PatelCelia Garcia MaloMinan Al-EzziCelia HardingMinbo ZhangCelia Redondo RodríguezMincheol HanCélia VenturaMine BekarCelian RomanMine SeçkinCelina-Silvia StafieMinenori IshidoCéline DondeynazMinerva Codruta BadescuCéline KosterMinerva CruzCéline TiffonMinerva MartínezCélio Geraldo Freire-de-LimaMinerva Vanegas FarfanoCelso Raul Romero RamosMing ChenCelson Pantoja LimaMing Chieh LeeCelstine IwendiMing GaoCemil ColakMing Hsien ChanCen WuMing LiČeněk ŠašinkaMing LiuCenk ÇalışkanMing Lun ChuangCenk Ulvi OezpekerMing Tatt LeeCeri PhillipsMing XiaCésar BerzosaMing Yu Claudia WongCesar BordehoreMingaleva ZhannaCésar Calvo-LoboMingbao FengCesar IsazaMingchao HuangCesar Ivan Aviles GonzalezMing-Cheng ShihCésar Johan Pereira-VictorioMing-Chih ChiuCésar Leal-CostaMing-Fong TsaiCésar LeãoMing-Fu WangCésar Leyva-PorrasMing-guang ZhangCesar M. Flores-OrtizMinghan HuCésar Merino-SotoMing-Hung HsuCésar Octavio Ramos-GarcíaMing-Ju TsaiCesar San Martin SalasMing-Jui HungCésar Torres-MartínMingjun WuCésar VeigaMingliang ZhangCesare CerriMingming TongCeu MateusMing-Ming WangCeyda ÖzgenMing-Quan GuoCezar MorarMing-Shu ChenCezary BekerMing-Ta YangCh GangadharMing-wei HsuChaang-Iuan HoMingxi DuChadwick Co Sy SuMingxia ZhangChaeseong LimMingxiao LiChafia KaraMingxiao SuiChahnez Charfi TrikiMingxin ZhangChai Hong RimMingxing SunChai Lee GoiMingyang ChengChairat NeruntaratMingzhi ZhangChaisak ChansriniyomMingzhu FangChaitanya GadepalliMinh Hieu NguyenChaitanya P. PuranikMinh Hoang NguyenChaiyavat ChaiyasutMinh PhamChalil George JoshyMinh Thac NguyenChamara RajapaksheMinhhuyen T. NguyenChamberlain I. ObialoMin-Ho HanChamila GunathilakeMin-Huan WuChamindu DeepagodaMinhui GuanChampa WijekoonMinjeong KimChan Hee ParkMinjung KimChan MunMinjung ParkChan Young KwonMinna SorsaChanaka NavarathnaMinna TorppaChanapa KongmarkMinqi YangChanchal Deep KaurMin-Seong HaChanchal MandalMinseong KimChanchal Sur ChowdhuryMin-So PaekChandan Swaroop MeenaMinsu KimChandima P. KarunanayakeMinsu ParkChandnee RamkissoonMinuk KangChandra Shekhar PantMinwir Al-ShammariChandrakanta MahantyMiodrag LovricChandramohan DhasarathanMiomir MijićChang GuoMir Aftab Hussain TalpurChang Gyu WooMir Faeq Ali QuadriChang HuangMir Hojjat SeyediChang Ji EunMir MatinChang Kyun ChoiMiran LeeChang Seop RheeMiranda DallyChang ShuMiranda HillChang Won LeeMiranda Miranda Muhvic-UrekChang XuMiranda NovakChang-Bae KimMiranda OcchioneroChangbo ZhangMircea Andrei ScridonChangchuan JiangMircea BoșcoianuChang-Eun KimMircea Constantin ScheauChangfeng DingMircea HuleaChang-Gu LeeMircea Ioan PopaChang-Han ChenMircea RivișChang-Hoon ChoiMircea ScheauChang-Hyung LeeMircea TampaChangli ZhangMircea Viorel DrăgoiChang-Min LeeMircea-Catalin FortofoiuChangqing SunMireia FausChangsheng GuoMireia LlauradoChangsup ShimMirela Cleopatra TomescuChangwon YooMirela ImreChang-Yi WuMirela J. Wolf-BacaChang-Yong JangMirela LozićChangyoon JeongMirela PanainteChangyou LiMirela PanaitChang-Yu HongMirela Pavicic IveljaChangzhen WangMirela PlaninićChangzhou YanMirela StanciuChanin YoopetchMirela TiglisChanjuan SunMirela TomašChantal AngueyraMiren AltunaChantal StheneurMireya Vilar-CompteChantel SloanMiri Tal-SabanChan-Yen KuoMiriam Aracely Anaya-LoyolaChao FengMiriam Araujo-HernándezChao GuMiriam BazzicalupoChao HuMiriam Beatríz VirgoliniChao MaMiriam BragaChao WuMiriam CalkinsChaobin YangMiriam HaaksmaChaofan XianMiriam HeymanChaopu TiMiriam Poza-MéndezChaoqi ChenMiriam SaridChaowei ZhouMiriam SchiffChaoyang DuMiriam ZacchiaChaoyi ChenMirian Graciela Da Silva Stiebbe SalvadoriChao-Yu GuoMirja AhmmedChapman RackawayMirjana KovačićCharalambos CharalambousMirjana Pejić BachCharalampos KrommidasMirjana RadenkovicCharalampos SiristatidisMirjana RadomirovicCharalampos ThomasMirko Di RosaCharat ThongprayoonMirko DuradoniCharee ThompsonMirko ManchiaChariklia K. DeliMirko MarrasChariklia Tziraki-SegalMirko ProsenCharise Dallazem BertolMirko WinklerCharisse B. PickronMirna Habuda-StanicCharitha DiasMirna MihelčićCharles AllenMirna VelkiCharles AppersonMirna ŽulecCharles C. CoddingtonMirnajaf MousaviCharles D. H. ParryMirona Palczewska-KomsaCharles E. MillerMiroslav BartakCharles Frederick AlfordMiroslav D. VujičićCharles HuangMiroslav HercegCharles IkerionwuMiroslav JůzlCharles J. GerardoMiroslav SližikCharles J. StuhlMiroslav SpacekCharles MarksMiroslava A. NedyalkovaCharles ReichhardtMiroslava KnapkováCharles Roberto TellesMiroslava TokovskaCharles ScottMirosław BiczkowskiCharles William YoxallMirosław GiurgCharles-Henri Di MariaMiroslaw JanikCharlotte BeaudartMiroslaw JanowskiCharlotte De GeyterMirosław KobierskiCharlotte JeavonsMirosław KucharskiCharlotte Kelley-JonesMirosław MaziejukCharlotte KentenMirosław RurekCharlotte MienieMiroslaw ZagajaCharlotte RoyeenMiroslaw ZimnochCharlotte Wendelboe-NelsonMirta CrovettoCharmaine Van EedenMirta MilićCharoula K. NikolaouMiruna NemeczCharusluk ViphavakitMirza AlamChasity BrimeyerMirza PojskicChatchai EkpanyaskulMirza R. BaigChatchaporn UraipongMisa NakamuraChathurangi PathiravasanMisagh FaezipourChaturaka RodrigoMisagh ParhizkarChau Nguyen DinhMisari OeChau-Ren JungMisbahuddin M RafeeqCheang KhooMishall Al-ZubaidieChee Keng MOKMisi SiChee Kong YapMislav MikušChee Meng Benjamin HoMislav PuljevićCheerawit RattanapanMister Gidion MaruChee-Seng TanMita BanerjeeCheil MoonMita MehtaChelsea CarpenterMitat KozChelsea PelletierMitch ClarkChelsee ShorttMitch RauhChelsey KirklandMitchel A. RosenChen CaiMitchell MackinemChen Chin-LingMitchell SmithChen FengMitsuaki HiraiChen HuangMitsugi ShimodaChen LiMitsuhiko HataChen PengfeiMitsuru ShimizuChen Seong WongMitsuya YamakitaChen ShenMittal RochakChen XuMiya BarnettChen YangMiya L. BarnettChen ZhangMiya YoshinoChen-Chi TsaiMiyazato NaokiCheng LiMi-Young ChoiCheng YanMiza MoreauCheng-Chen ChenMizue SuzukiChengcheng XiaMladen AmovićCheng-Chia YangMladen AndjićCheng-Fang LiMladen KrstićCheng-Fang YenMladen MaradinChenghao ChenMM KhanChenghao WangMmm MowjoodCheng-Hsiung HuangMo ChenCheng-Hsuan LuMoacir MarocoloCheng-Jen ChangMobashir MohammadChengjiang LiModesti Martina NicoleChengjun HuangMohamad Fairus RabuniCheng-Liang WangMohamad HeriawanChengpeng LuMohamad MaghnieChengqi XiaMohamad MotevalliCheng-Shun LuMohamad Nabil AlmunawarChenguang DuMohamad Rizal BaharumChengwei HuangMohamad Rodi IsaCheng-Wen LeeMohamadi SarkarChengxu ZhouMohamamd A. Al-MamunCheng-Yang HuangMohamed A. ElsadekCheng-Yih HongMohamed A. HabilaChengyu GaoMohamed A. TahoonChengyu LinMohamed AbdallahChen-Hua FuMohamed AbdelgiedChenlin HuMohamed Abuelseoud Abdelzaher AliChen-Long WuMohamed AlkafafyChenlu GaoMohamed BarakaChenming JiangMohamed ElnaemChenxi LiMohamed El-RaiyChenxi LiuMohamed El-SherbinyChen-Yi SunMohamed EmranChenyu SunMohamed Esmail KararCheol-Hwan YouMohamed EssaCherie D. MaestasMohamed F. M IbrahimCher-Rin ChongMohamed Faisal ChevidikunnanCheryl BeselerMohamed FawzyCheryl CroppMohamed FodaCheryl Fairfield EstillMohamed GadCheryl JonesMohamed K. MostafaCheryl LinMohamed MahdiCheryl McQuireMohamed MeshrefCheryl RoumenMohamed MohanyCheryle G. LevittMohamed Rafik N. QureshiCheuk Lun LiuMohamed S. EliwaChe-Yu HsuMohamed Salah El-Din HassounaChe-Yuan KuoMohamed Salem NashwanChhanda BoseMohamed SalimChhaya PatelMohamed Yaseen JabarullaChi Kin KwanMohamed-Bassem Ali AshourChi LiMohammad (Behdad) JamshidiChi Shing WongMohammad A. AlkhamisChi Tung ChoyMohammad A. SaadChia Huei LinMohammad AatifChiachi WangMohammad Abdul Momin SiddiqueChia-Chi YangMohammad Abdus SalamChia-Hong YengMohammad Abu Zafer SiddikChia-Hsiang LaiMohammad AhsanChia-Hung ChouMohammad AkbariChia-Lun LoMohammad Al MamunChia-Min LinMohammad AlanafeaChia-Ming ChangMohammad AliChian Ju JongMohammad Ali TareqChian-Wen KaoMohammad Al-WardatChiao Xin LimMohammad AsghariChiara Alessandra CapogrossoMohammad AzarafzaChiara BaldacchiniMohammad BasyuniChiara BodiniMohammad Belal HossainChiara BurattiniMohammad DarabsehChiara CadedduMohammad El KhatibChiara CappelliMohammad EtoomChiara CorvinoMohammad Fahad UllahChiara D’AngeloMohammad Farhan KhanChiara de WaureMohammad Farooq UmerChiara DonfrancescoMohammad Hasan KhoshgoftarChiara GallioneMohammad Hashem AskariyehChiara GrassoMohammad Hashemi-TabatabaeiChiara ImperatoMohammad HassanChiara La TorreMohammad HomayounpourChiara MartinelliMohammad Hossein KhosraviChiara MussiMohammad Hossein MaghamiChiara OppiMohammad Javad Maghsoodi TilakiChiara PagliariMohammad Javad MohammadiChiara PeciniMohammad Junaid KhanChiara PolichettiMohammad KakooeiChiara ScanagattaMohammad Khursheed AlamChiara SorberaMohammad Mahbubur Rahman Khan MamunChiara VanniMohammad Mahmoodi HashemiChiara VillaMohammad MasukujjamanChiara VisentinMohammad Mehdi AlemiChia-Ting SuMohammad MehdizadeChia-Tsung YehMohammad Mohiminul IslamChia-Wei LeeMohammad MohsenzadehChia-Yu ChenMohammad Mostafa Ansari-RamandiChibuzor UdokwuMohammad NabipourChi-Chien KuoMohammad NajafzadehChich-Ping HuMohammad NajjarChie Tamamoto-MochizukiMohammad Nazari-SharabianChieh Yu LinMohammad Nisar KhattakChien Chung HuangMohammad RababaChien-Cheng JungMohammad RabbiChienhsiu HuangMohammad Radwanur TalukderChien-Huei HsuMohammad Rafe Bin HatshanChien-Hung LaiMohammad RahmanChien-Hung LeeMohammad Reza MahmoudiChien-I ChenMohammad Reza RahdariChien-Li ChenMohammad Rezaur RahmanChien-Liang LinMohammad S. ImtiazChien-Ming ChaoMohammad Sadegh Keikhosravi KianyChien-Shu TsaiMohammad Sadra RajabiChien‑Wen ChenMohammad SafariChien-Yi ChangMohammad Shahnawaz GhaziChifa ChiangMohammad Shakhawat HossainChih Pei HuMohammad SharifChih-Cheng ChangMohammad ShboolChih-Chi ChenMohammad Sultan AlakhaliChih-Chin LiangMohammad Tahir SiddiquiChih-Chin YangMohammad Tarequl IslamChih-Ching LiuMohammad ThaljiChih-Hao YangMohammad YaminChih-Hsin HsuMohammad Yaseen AbbasiChihhsiong ShihMohammad ZamaniChih-Hsiung HsuMohammad ZulkifliChih-Hsuan YenMohammadamin EmamiChih-Jen LuMohammadreza FayazChih-ling LiouMohammadreza HeydartaemehChih-Long LinMohammadreza MohebbiChih-Ming LinMohammadreza RezaeigolestaniChi-Horng LiaoMohammadreza ShalbafanChih-Ping YangMohammadvaghef GhazvinianChih-Wei PaiMohammed A. Al-SharafiChih-Wen WuMohammed A. DahabChih-Yuan FuMohammed A. FayadChika HondaMohammed A. KarimChikako HondaMohammed AbbasChikashi SatoMohammed AklChi-kin LawMohammed Al ZobbiChin Siang KueMohammed AlaswadChin Wen CongMohammed AlghamdiChin Yen HanMohammed Al-HusbanChina NaganoMohammed AlmukayniziChin-An WangMohammed Arshad KhanChinatsu NishidaMohammed AsadChin-Chen ChuMohammed Bait Ali SulaimanChing Sin SiauMohammed Ben AliChing-Cheng ChangMohammed BindakhilChing-Cheng ShenMohammed Dauda GoniChing-Chung HsiaoMohammed DiykhChing-Yeh TsaiMohammed EmranChing-Yi ChangMohammed F. H. BalahaChing-Yi ChenMohammed Ghazi SghaireenChin-Hsien HsuMohammed GrawishChin-Hsuan LiuMohammed HabesChin-Huang HuangMohammed HajjajChinmay ChakrabortyMohammed HssaisouneChinmoy SarkarMohammed IsamChin-Ping KungMohammed J. K. BashirChin-Ting LiuMohammed Khaled Al-HanawiChin-Wei WangMohammed LoudikiChin-Yen Alice LiuMohammed M. BeyariChin-Yu LinMohammed NadarChitiphon ChuaichamMohammed NassarChiung Yao ChenMohammed OmerChiung-Tzu Lucetta TsaiMohammed RasheedChiu-Yue LinMohammed RohaimChi-Wei LinMohammed Saad KamelChi-Wei SuMohammed SabbahChi-Wen ChienMohammed Saleh Ali MuthannaChi-Wen LungMohammed Shamim KaiserChloe BedardMohammed Yacin SikkandarChloe FletcherMohammed ZawiahChloe JiangMohan TanniruChloe KrakauerMohd Adli B. Md AliChloë WilliamsonMohd AdnanChng Saun FongMohd Bijarimi Mat PiahCho Nilar PhyoMohd Dzulkhairi Mohd RaniChoirul AminMohd Fadhli KhamisChokri KooliMohd Hafiidz JaafarChona HannahMohd Hafiz ArzmiChong QiMohd HanafiChong WeiMohd Hasni JaafarChongjing CaoMohd Helmy Bin MokhtarChongming WangMohd Idzat IdrisChongqing WangMohd Lokman IbrahimChongyang LiMohd Noor NorhayatiChongyu DangMohd SaifuzzamanChoo Yee YuMohd Shafie RosliChoon Fu GohMohd Shahezwan Abd WahabChoon Weng LeeMohd Yawar Ali KhanChoosak NithikethkulMohit ChawlaChris BlackmoreMohit JoshiChris BusbyMohit TyagiChris BuseMohsen AminizadehChris CurtisMohsen AnnabestaniChris DakinMohsen BakouriChris DouvrisMohsen MaghrebiChris Fife-SchawMohsen NasseriChris FradkinMohsen OmidvarChris LabakiMohsen SalimiChris MontenMohsen ZareChris PauluMohsin Usman QureshiChris PenlingtonMohtaram RabbaniChris WoodMoïra MikolajczakChrissie ButtsMoises Armides Franco-MolinaChrista J. Van DortMoises BetancortChristalla PitharaMoisés Grimaldi-PuyanaChristelle Bou-MitriMoises Leon-JuarezChristian A. Maino VieytesMoisés MarquinaChristian AuerMojgan AlaeddiniChristian BeltzerMojtaba EhsanifarChristian BrinkmannMojtaba FarjamChristian Campos-JaraMojtaba KavianiChristian CarulliMojtaba KeikhaChristian De GuzmanMojtaba VaismoradiChristian Di FlorioMojtaba YariChristian FrancoMokarram HossainChristian François RoquesMokoena Patronella MaepaChristian FreudlspergerMoktar ChtaraChristian HeumannMolly C. NicodemusChristian HulenMolly Duman-ScheelChristian ImbodenMolly NicodemusChristian J. Cabello-AlvaradoMomcilo JankovicChristian JanousekMoming LiChristian Juhl TerkelsenMominul AhsanChristian KaczmarekMominur RahmanChristian KanzianMomotazur RahmanChristian KetelsMona IonașChristian KrägelohMona JabbariChristian KuklaMona Mohamed Amin Abdel-FatahChristian LückMona VintilaChristian Luiz Da SilvaMoni RoyChristian M. RingleMonia LenziChristian Maurer-GrubingerMonia VagniChristian MertensMonica AchimChristian MesengeMonica BalzarottiChristian MoritzMonica CardarilliChristian N. MaduMónica CostaChristian NapoliMonica DutcasChristian OltraMonica GiancottiChristian PozziMonica Gruezmacher RosasChristian RitzMonica GuglielmettiChristian SchachMonica Ioana Burcă-VoicuChristian SchindlerMonica L. RobinsonChristian SiatkaMonica LöfvanderChristian SpreaficoMonica Lopes FerreiraChristian WernerMonica MaltaChristian WrightMonica MoneaChristian WunderMónica Morais De BritoChristiane GerardMónica Muñoz-ÚbedaChristiane SchefflerMonica NordbergChristiane StockMonica PalmaChristin HeidemannMónica Ríos-SilvaChristina AggarMónica SantosChristina Anna StratopoulouMonica Solana-TramuntChristina C. BartenschlagerMónica Suárez-ReyesChristina CarlanderMonica TarceaChristina CoughlanMónica Taveira PiresChristina Dieli-ConwrightMónica Teresa González-RamírezChristina E. LarderMonica Unsgaard-TøndelChristina EmmanouilMónica Viñarás AbadChristina GyörkösMônica Volino-SouzaChristina HeemskerkMonidipa DasguptaChristina HensonMónika AndokChristina HerisMonika BaumanováChristina LaspaMonika BlišťanováChristina LundbergMonika Boguszewicz-KreftChristina MalatzkyMonika BronkowskaChristina Maresch BernardesMonika Dmitrzak-WęglarzChristina Marie ZalaruMonika E. RacChristina MarneyMonika Foltyn-ZarychtaChristina MoulogianniMonika FranczukChristina Nikitopoulos SklibosiosMonika GałczykChristina Oetzmann Von SochaczewskiMónika Garai-FodorChristina PaxmanMonika GebskaChristina Pettan-BrewerMonika HalánováChristina RoussiMonika JakubusChristina S. BolloMonika KleinChristina TortorelliMonika KniotekChristina VanderwelMonika KoziełChristina ZorbasMonika KrukowskaChristine A. JamesMonika KrzyżanowskaChristine AblazaMonika LesickaChristine BarulMonika Łopuszańska-DawidChristine CansMónika MeiczingerChristine E. MerrileesMonika MichaelisChristine E. RittenourMonika Michałowska-SawczynChristine G. ParksMonika MladenovićChristine HackmanMónika MolnárChristine Lorre-JohnstonMonika PapieżChristine M. GagnonMonika ParchomiukChristine M. MermierMonika PiatkowskaChristine Manlai KwanMonika PrzeorChristine Maria SchwarzMonika PrzestrzelskaChristine PreiserMonika ReichertChristine RogersMonika RucinskaChristine San GiovanniMonika Šabić RunjavecChristine TaylorMonika SnowdonChristine Tessele NodariMonika SporekChristine UnsonMonika StomaChristofer BesterMonika Szymańska-CzerwińskaChristoffer BomanMonika TrojanowskaChristoph BorzikowskyMonika TrząskowskaChristoph BuchtaMonika WawrzkiewiczChristoph BurgerMonika Zawadka-KunikowskaChristoph ClephasMonika ZiemskaChristoph FaschingerMonique MokhaChristoph FriedliMonique Y. C. AraújoChristoph KurzMonir Moradzadeh KhiaviChristoph LechnerMonireh Sadat SeyyedsalehiChristoph NedopilMonserrat Termes-RiféChristoph NovakMontree TungjaiChristoph R. MeierMontserrat Boronat NavarroChristoph SchieferMontserrat EsteveChristoph SpangMontserrat ZamoranoChristoph StachMontserrat Zamorano ToroChristoph StallmannMoon Fai ChanChristoph StrumannMoon HyoungeunChristophe BarreaMoonisa Aslam DervashChristophe BlaisonMoraitou DespinaChristophe DomingosMoran BodasChristophe PlantampMoran PollackChristopher A. BeaudoinMoreno BaruffiniChristopher A. SimonMoreno D’AmicoChristopher Adair-ToteffMorgan BroggiChristopher Adam-BagleyMoria GolanChristopher ArnuschMorin LangChristopher BarnesMoritz Blanck-LubarschChristopher BoehlkeMoritz MarkelChristopher BryantMorten GjerdeChristopher E. AndersonMorten HesseChristopher E. SiorisMorteza Arab-ZozaniChristopher EllisonMorteza Fallah KarkanChristopher FahsMorwenna KirwanChristopher FergusonMosab TabashChristopher G. BeehnerMoshood OnifadeChristopher G. KempMoshtagh R. FarokhiChristopher G. WilsonMoslem SavariChristopher Holzmann-LittigMossab Y. SaeedChristopher J. WomackMostafa A. AbooufChristopher J. YoungMostafa Abo ElsoudChristopher John LundstromMostafa Amani MachianiChristopher Keith HaddockMostafa Amini RaraniChristopher KirkMostafa DianatinasabChristopher L. AtkinsonMostafa E. ShahenChristopher LoMostafa F. N. AbushahbaChristopher LovinsMostafa GoudaChristopher MansourMostafa Hajiaghaei-KeshteliChristopher McLeodMostafa M. El-SheekhChristopher McManusMostafa Mohammed Hasan Tawfeek MohammedChristopher OwensMostafa WalyChristopher PayanMotahareh SaadatpourChristopher R. GustafsonMotasem AlazaizaChristopher RobbinsMotaz HamedChristopher RogersMotofumi SuzukiChristopher SeitzMotoharu TakaoChristopher SprayMotoyoshi MorishitaChristopher SwiftMoudatsou MariaChristopher T. OwensMoulay Abdelmonaim El HidanChristopher V. HughesMoumita GhoshChristopher VineMounia TahriChristopher WilcoxMounir MesbahChristopher WilkinsonMourouzis PetrosChristos IoakimidisMoustafa NasrallaChristos K. YiannakopoulosMoustafa T. MabroukChristos KontogiorgisMousumi DebnathChristos KourekMo-Yeol KangChristos MarkosMpho MoleteChristos PapakostasMriganka SinghChristos PouliarisMrill IngramChristos RoumposMrudula NaiduChristos TsabarisMrutyunjay JenaChristos TsagkarisMst Ilme FaridatulChristy A. GreenleafMu NaushadChristy D. Moran CraftMuafi MuafiChristy Ling HomMuawuz IjazChrysa VoyiatzakiMubarak AltwaijiChrysan CroninMubarak S. KarsanyChrysanthi LeonidouMubashir SiddiquiChrysoula BetsouMubbasher MunirChuan-Fa ChangMudasir DarChuang-Yuan ChiuMudassar Iqbal ArainChuanqi TanMuddasarul HodaChuansi GaoMuddassar SarfrazChuanyun FuMudit TyagiChueh-Lung HwangMugurel Constantin RusuChukiat ChaiboonsriMuhamad Azahar AbasChulin LikasiriMuhammad A. RahmanChuloh JungMuhammad Abdul KamalChu-Lun HsiehMuhammad Adeel AhmedChulwon KimMuhammad AfzaalChun ChenMuhammad AkramChun Hung ChuMuhammad Ali ImranChun XieMuhammad AlkasabyChunbo HaoMuhammad Altaf KhanChun-chang LeeMuhammad AnsChun-Chieh KaoMuhammad AnshariChung ChoeMuhammad ArifChung-Dann KanMuhammad ArshadChung-Fu HuangMuhammad Ashraf FauziChung-Han HoMuhammad Ashraf JavidChungho LauMuhammad AsimChung-Hui LinMuhammad Asim NawazChung-Jen HuangMuhammad AslamChung-Jen WangMuhammad AzamChung-Ming ChenMuhammad Azeen AshrafChung-Shu LeeMuhammad BilalChung-Tung J. LinMuhammad ChutiyamiChun-Gu ChengMuhammad Dan Asabe AbdulrahmanChung-Ying LinMuhammad Faisal ShahzadChun-hong LiuMuhammad Faraz MubarakChunhong ZhaoMuhammad FarooqChun-Hou WangMuhammad FarrukhChun-Hsiung HuangMuhammad Hammad ButtChun-Hung ChangMuhammad Hassan KhanChunhung LinMuhammad HumayunChunil KimMuhammad IbrahimChunjie WangMuhammad Ibrahim AbdullahChunki FongMuhammad Iftikhar Ul HusnainChunlei HanMuhammad IhtishamChun-Liang ChenMuhammad IjazChun-Liang LinMuhammad IkramChunmei GengMuhammad Ikram UllahChunmiao YuanMuhammad ImranChun-Por WongMuhammad IqhrammullahChun-Qing ZhangMuhammad IrfanChun-Ru ChienMuhammad Irfan SiyalChunshan ZhouMuhammad Irfan SohialChunsheng FangMuhammad Irwan Padli NasutionChunsheng WuMuhammad Junaid FarrukhChunshui LinMuhammad Junaid MunirChuntian LuMuhammad Kaleem KhanChunui LeeMuhammad Kashif KhanChun-wang WeiMuhammad Kashif ShahidChun-Yueh LinMuhammad Khalid BashirChunzhi YiMuhammad Khalis Abdul KarimChutima JalayondejaMuhammad KhurramChutisant KerdvibulvechMuhammad MohiuddinChu-Yu HuangMuhammad MohsinChyan-Long JanMuhammad Moinuddin Qazi AbroChyi-Feng JanMuhammad Mubashar IdreesChyi-In WuMuhammad Mushahid AnwarCiavoi GabrielaMuhammad N. MahmoodCid André Fidelis De Paula GomesMuhammad N. ZahidCid Marcos Gonçalves AndradeMuhammad NadeemCidalia OliveiraMuhammad PervaizCiera E. SchoonoverMuhammad Qaiser ShahbazCilia Mejia-LancherosMuhammad Qamer ShahidCindy BlairMuhammad QaswarCindy CrawfordMuhammad RiazCindy TraboulsiMuhammad RizwanCinta Barba BriosoMuhammad Rokhis KhomarudinCinta ZapaterMuhammad Saad ShaikhCintia Chamorro-PetronacciMuhammad SalmanCíntia FrançaMuhammad SardarazCintia Maria RodriguezMuhammad ShafiqueCinzia CorrealeMuhammad ShahidCinzia La RoccaMuhammad Shoaid ArifCinzia MarinaroMuhammad Suleman RanaCinzia RienzoMuhammad TahirCiprian ManzuMuhammad Tahmidul HaqCiprian RoiMuhammad TariqCiprian SimutMuhammad TufailCiro BritoMuhammad Usman KhanCiro De LucaMuhammad Usman KhurramCiro EspositoMuhammad Wahab AmjadCiro GaleoneMuhammad ZeeshanCiro José Jardim De FigueiredoMuhammad Zia AslamCiro Rosario IlardiMuhammad ZubairCitlali TruetaMuhammad Zudhy IrawanClair MerrimanMuhammed SaeedClaire BadenhorstMuhammed Sedat SakatClaire BrolanMuhammed Yunus BektayClaire BrownMuhammed Ziya PaközClaire De BisschopMuhammet Fatih AslanClaire E. AdamMuhammet KarakavukClaire FlemmerMuhittin Hakan DemirClaire O’DonnellMuhlis CanClaire SmithMuhsin KilicClaire StockerMujahid FaridClaire TournyMujtaba HassanClara CerratoMujtahid KaavessinaClara IversonMukesh Anand KumarClara Martinez-PerezMukhtar A. KassemClara Pons-DuranMukhtar AhmedClara TalensMukhum HammadClara Vidal CarullaMukti SubediClare HanmerMulazim Hussain AsimClare M. P. RoscoeMunawir MunawirClare MckeaveneyMunehisa ShinozakiClare RaynerMuneto TatsumotoClarence E. GrimMunikumar ManneClarice Alves BonowMünir AktaşClarice TangMuqing DuClarissa Marcelle NaidooMurad HossainClarita Rodríguez SotoMurad RassamClark H. DennyMurali DamaClas-Håkan NygårdMuralikrishna LellaClaudette PooleMurat AkserClaudia AndradeMurat AycanClaudia CalvanoMurat BaşarClaudia CameddaMurat EkiciClaudia CampanaleMurat EyubogluClaudia CortiMurat EyvazClaudia CovucciMurat KirişciClaudia CrociniMurat TezerClaudia DamianiMuriel AbbaciClaudia Daniele De SouzaMuriel Ramírez-SantanaClaudia DellaviaMurod IsmailovClaudia DobreMurtaza SayedClaudia DuranMurthy MittintyClaudia Estela Saldaña DuránMurugan PattusamyClaudia F. ParvantaMurugesh ArunachalamCláudia FariaMusa MangaClaudia FerrarisMusfique AhmedClaudia FoersterMusheer AljaberiClaudia GallertMustafa AkkayaClaudia GiacomozziMustafa Al-MukhtarClaudia HansonMustafa CemaliClaudia HiltonMustafa CesurClaudia Iveth Astudillo-GarcíaMustafa FawzyCláudia Jacques LagranhaMustafa ÖzerClaudia LabiancaMustafa ÖztürkClaudia LevenigMustafa Said YıldızClaudia Luévano-ContrerasMustapha HidouriCláudia Margarida de Sousa E. SilvaMutahira LoneClaudia Maria BalzarettiMuthu ThiruvengadamCláudia Maria Ferreira PereiraMuthunarayanan VasanthyClaudia Maria MessiasMuttin FrédéricClaudia Maria Simões MartinezMuyesaier TudiClaudia MarottaMuyiwa OladosunClaudia MassarottiMuzaffer Can IbanClaudia MehedintuMya Myat Ngwe TunClaudia NiessnerMyeounggon LeeClaudia PetersMykhailo SavenetsClaudia PisanuMykola MakhortykhCláudia Regina Furquim De AndradeMykolas Simas PoškusCláudia Regina Pereira QuaresmaMyo Nyein AungCláudia Ribeiro De AlmeidaMyongsoon SungClaudia Roberta De Castro MorenoMyoung Soo KimClaudia RöhlMyoung-Ho HyunClaudia RusMyoungjin KwonClaudia SarduMyra LeungCláudia Sirlene OliveiraMyriam Alvariñas-VillaverdeClaudia StöllbergerMyriam De La IglesiaClaudia Tejada-GallardoMyriam Medina-VeraClaudia TodaroMyriam Van WinckelClaudia TomozeiMyron TsikandilakisClaudia TraunmüllerMyrto KasioumiClaudia TucleaMythily SubramaniamClaudia ViegasMyung Chae JungClaudia VogelMyung Goo LeeClaudia ZaniniMyung Ja KimClaudimar Pereira Da VeigaMyung-Bok WieClaudina NogueiraMyung-chul KimClaudine AugerMyung-Gwan KimClaudine BégheinMyung-soon KwonClaudinei De Souza GuimarãesN. L. PanwarClaudio Alberto Dávila-CervantesN. P. Dammika PadmakanthiClaudio ColomboN. Rich NguyenClaudio De LazzariNa ChenClaudio de LuciaNa GaoClaudio LucchiariNa SongClaudio MichelettoNa Young AhnClaudio SpinelliNaba AmgainClaudio TirelliNabaz R. KhwarahmClaudio UcciferriNabeel AlmotairyClaudiu C. PopescuNabi ShariatifarClaudiu George BoceanNabil A. HasonaClaudiu KiforNabil GmadaClaudiu MereutaNabil HossineyClaudiu MorgovanNabila BrihmatClaus E. AndersenNaďa AntošováClayton SmithNađa BeretićCleber Nunes KrausNada El OstaClemens BauerNada MarićClément BarsNada MzidClément LahayeNada SmigicClemon GeorgeNadav Perez-VaisvidovskyClifton AddisonNadeem IqbalClifton HolmesNadeem KhanClive D’SouzaNadezhda AngelovaClive FriedmanNadezhda LvovaClive PalmerNadezhda N. PokrovskaiaClive RichardsonNadezhda ShmelevaClotilde B. AngelucciNadezhda Yu StepanovaClyde F. MartinNadia AleemCodruta Adina BaltescuNadia AyubCody E. MorrisNadia CattaneCoeliac Annalisa SchiepattiNadia DepetrisCohen EmmanuelNadia El DibColette Balice-BourgoisNádia EusébioColin Anthony JonesNadia IntanColin CombsNadia NajafiColin DrummondNadia PreghenellaColin G. PenningtonNadia SelimColin HamiltonNadim AlmoshmoshColin Michael HallNadine TramowskyColin MulliganNadinne RomanColleen G. HergottNadir MamilovColleen M. GallagherNadire CavusCollin A. WebsterNadja FreundCollin JeromeNadja SchröderCollins K. TanuiNaeem Akhtar QaisraniCollynn WoellerNaeem HayatCôme ThieulentNaeem JanConceição Ilda Da Silva GomesNaeem KhanConceição SantiagoNa-Eun ChoConcepcion Carratala-MunueraNaga Babu ChinnamConcepción De Hita CantalejoNaga SamjiConcepción Pérez-LamelaNagavendra KommineniConcepción Soto-VidalNagesh PeddadaConcetta De PasqualeNagisa SugayaConcetta Di NoraNagy HenriettaConcetta PirontiNahal HabibiConcetta PirroneNahoko HaradaConcettina FengaNahrizul Adib KadriConcha AntónNa-hyeon (Hannah) KoConghua WenNaiara DemnitzCongsheng FuNaiara Ozamiz-EtxebarriaConn RotherNai-Jen ChangConnie OxfordNaila SharmeenConnie TompkinsNailya DelellisConnor Richard MillerNaiping GaoConrad MurendoNaira DelgadoConsolación Gómez-ÍñiguezNajafi Moghaddam Gilani VahidConsolato M. SergiNaji AlqahtaniConstantin NechitaNaji KharoufConstantinos DavosNajith AmarasenaConstantinos KoutsojannisNajmeh JafariConstantinos NicolaouNakata YoshioConstantinos ZervidesNalini ChintalapudiConstanza P. SilvaNallusamy SivakumarConstanze OlmsNalok DuttaConsuelo ChacarteguNam HuynhConxita MestresNameCoralie BarbeNamhee KimCordelia Estevez-CasellasNamhyun KimCorentin J. GoslingNamil UmCorey StiverNamita ShresthaCorina Anca SimionNamki ChoCorina Cristiana NastacăNan JiangCorina GüthlinNan LinCorina IoanășNan WangCorina Mihaela GruescuNana Aisha GarbaCorina PaulNana Amoah-RameyCorina SporeaNana HyldigCorina ZugravuNana Kwadwo BiritwumCorinna CullerNancai PeiCorinna Lesley SeidelNanching YehCorinna NoelNancy A. SkoppCorinna PeiferNancy DoetzelCorinne McDaniels-DavidsonNancy HaskellCorinthias P. M. SianiparNancy HelouCornelia AmalineiNancy JaoCornelia ConradieNancy L. BlackCornelia FiesslerNancy Long SieberCornelia GottschickNancy MendozaCornelia GyorodiNancy R. MudrickCornelia WiechersNancy S. BolousCorneliu BolboceanNancy S. YounisCorneliu CojocaruNancy Van LoeyCornelius EwuosoNandan ShettyCorrado BattistiNandkishor M. DhawaleCorrado ColapricoNan-Jay SuCorrado lo StortoNanon LabrieCorrado MagnaniNan-Ping YangCorrado RindoneNanxi YanCorrado ZoppiNaoharu YagiCorrine Warren RuktanonchaiNaoki FukutaCortés-Castell ErnestoNaoki KanoCorwin E. SmidtNaoki KunugitaCory M. SmithNaoki MaruyamaCory SnyderNaoki SudoCosimo GallettiNaoki ToyamaCosimo PalagianoNaoko HorikoshiCosmas Ifeanyi NwakanmaNaomi GefenCosmas NathanailidesNaomi MatsumotoCosme Franklim BuzzacheraNaomi SteenhofCosmin CituNaoro TanakaCosmin Mihai VesaNaoru KoizumiCosmin O. PopaNaoto YoshidaCosta DamienNaoufal RaissouniCosta SolangeNaoya HasegawaCostantino BalestraNarashans Alok SagarCostanza Scaffidi AbbateNarasimha Rao PillalamarriCostas S. ConstantinouNarcis MasollerCostas VarotsosNardine NakhlaCourage MlamboNardos AbebeCourtney AndrewsNaregundi KarunakaraCourtney DowNarendar DudhipalaCourtney ThompsonNaresh DamukaCraig DepkenNaresh Krishna VissaCraig DonnachieNaresh KumarCraig DownsNareswari CinintaCraig McGartyNariane BernardoCraig PearlNarimasa KumagaiCraig SlatinNarkeesh ArumugamCraig TalmageNark-Kyoung RhoCraig WilkieNarongchai AutsavaprompornCrenguța Cristina AlbuNarsingh KumarCrétien M. C. Van CampenNaseer AhmedCricket MeehanNaser AlsharairiCrina CotocNaser KabashiCrina Julieta SinescuNaser ValizadehCringu Antoniu IonescuNasheen NurCristi SpulbarNasim Ahmad YasinCristian ÁlvarezNasim MontazeriCristian BaicusNasima AkhterCristian BersezioNasir IqbalCristian BonviciniNasir MahmoodCristian Calvo BarentinNasir Muwfaq YounisCristian Davalos-SaucedoNasser LaoualiCristian DelceaNasser ShararehCristian DigestoNasser TuqanCristian FerreiroNasser YimenCristian FurauNassib B. BuenoCristian LieneckNastaran AhmadiCristian MălinașNastaran Mohammadian RadCristián OyanadelNasya BahfenCristian PetcuNatacha CoelhoCristian PolizianiNatacha Jesus-SilvaCristian RotariuNatale Salvatore BonfiglioCristian ScheauNatalia A. BezdenezhnykhCristian Silviu BanacuNatalia A. Kozak-MuiznieksCristian TrambitasNatalia AversanoCristiana Furtado FirminoNatalia BelkovaCristiana GambelungheNatalia BelosludtsevaCristiana OliveiraNatalia Burgos AlonsoCristiane Aguiar Da CostaNatalia BursiewiczCristiane Helena GallaschNatalia Calvo AyusoCristiane Salomé Ribeiro CostaNatalia Cezon-SerranoCristiane Yumi Koga-ItoNatalia CheshenkoCristiano PesaresiNatalia Deeb-SossaCristiano ScandurraNatalia DistefanoCristian-Valeriu PatricheNatalia DowgiałłoCristina AdillónNatalia E. Fares-OteroCristina Alexandrina StefanescuNatalia GrzebiszCristina Anna Maria Lo IaconoNatalia Hernández-SeguraCristina B. NevesNatalia HudzCristina BaglivoNatalia Ivanovna AgalakovaCristina Bianca PocolNatalia KascakovaCristina BicaNatalia KasicaCristina CanovaNatalia Kaźmierczak-WojtaśCristina CapatinaNatalia KizilovaCristina Ciudad-BlancoNatalia KorczCristina CiuluvicaNatalia Lajara-CamilleriCristina CortisNatalia LauferCristina CosteiraNatalia MadetkoCristina CoutoNatalia Martínez-LeónCristina Di TeccoNatalia MazanowskaCristina DrumeaNatalia Morgulec-AdamowiczCristina Esmeralda Di FrancescoNatalia NadrowskaCristina Espinosa-SempereNatalia NehrebeckaCristina FaludiNatalia RamosCristina FloreaNatalia Romero-FrancoCristina Floriana PanăNatalia RovellaCristina GagliardiNatalia SabelnikovaCristina GalvanNatalia Salas-GuzmánCristina García FernándezNatalia ShnayderCristina Garcia-MunozNatalia SobolevaCristina GenoveseNatalia Soriano-SarabiaCristina Georgiana ZeldeaNatália StofelCristina GhineaNatalia SuárezCristina GiannattasioNatalia SzejkoCristina Jorge-SotoNatalia Tomczewska-PopowyczCristina Lavareda BaixinhoNatalia TomskaCristina Liébana-PresaNatalia Ukleja-SokołowskaCristina Lin-LianNatalia V. KuzmenkovaCristina Lirio RomeroNatalia V. YakovenkoCristina Lojo-SeoaneNatalia Vasilievna EfimovaCristina López CadenasNatalia Viktorovna NizyaevaCristina LullNatália VraňakováCristina Manuela DrăgoiNatalie A. ProwCristina MaroiuNatalie BrownCristina Mendoza-HolgadoNatalie PearsonCristina MenescardiNataliia TrushkinaCristina MennittiNatalija Ursulin-TrstenjakCristina MesquitaNatalio García-HonduvillaCristina Mihaela LacatusuNataliya PodgorodnichenkoCristina MocanuNataliya Valentinovna YaglovaCristina MonteiroNatalja DyorinaCristina Moreno-MuletNatallia B. HanssenCristina MurphyNatallia V. DubashynskayaCristina NoriegaNatalya GlushkovaCristina NunesNatarajan AyithanCristina Onesta MossoNataša B. SarapCristina PetrucciNatasa Bogavac-StanojevicCristina Pulido RodríguezNataša Danilović HristićCristina Raluca PopescuNataša Ivančić JokićCristina Rey-ReñonesNataša KovacicCristina Rivera-PicónNataša LjubičićCristina Roldán-JiménezNataša MatulayováCristina Romero-BlancoNataša RančićCristina SalvioniNatasa Slak ValekCristina Sánchez-MartínezNataša StojićCristina SecosanNatasa TodorovicCristina SernaNataša VardaCristina SkrypnykNatasa Vujica HerzogCristina SoaresNataša Zenić-SekulićCristina Sotomayor-CastilloNatascia BrondinoCristina Souza Freire-NordiNatascia RinaldoCristina TeixeiraNatasha P. Sobers-GrannumCristina Torrelles-NadalNatasha ParentCristina TrentiniNatasha ReidCristina TruzziNathalia PadillaCristine Vieira Do BonfimNathalia PizatoCristo M. Marrero-GonzálezNathalia Tejedor-FloresCristóbal Macías VillalobosNathalie AndréCristoforo ComiNathalie ColasantiCristoforo PomaraNathalie Hill-KapturczakCristopher SegoviaNathalie VermeulenCrystal R. ArcherNathan D. DicksCsaba BojtorNathan K. EvansonCsaba CenteriNathan Kumasenu MensahCsaba KorenNathan M. D’CunhaCsaba LantosNathan PitchfordCsaba PatkósNathan W. HillCsaba VeresNathanael OjongCuijuan HanNathaniel A. ShanokCuilan LiNathaniel Aviv CohenCuong Huu NguyenNathaniel T. BerryCurelaru MihaiNatheer KhasawnehCurtis FennellNato DarchiaCurtis HedmanNatsuki HasegawaCurtis LehmannNatthachet TangdamrongsubCurtis SmithNava ZisapelCynthia BaurNavaneeth Krishna Rajeeva PandianCynthia CorbettNavarro Gómez NoeliaCynthia E. MuñozNavdeep GognaCynthia IsleyNaveed AhmadCynthia Jan CoffmanNaveed Ullah KhanCynthia Kay ChandlerNaveen Chandra JoshiCynthia L. BodkinNaveen MekalaCynthia SchairerNaveen PuttaswamyCynthia T. ThompsonNavid Jubaer AyonCynthia WhissellNavin KaushalCynthia YoonNavin Kumar DevarajCyprian Mcwayizeni MostertNavin Kumar OjhaCyprian OlchowyNavneet KumarCyril FersingNavneet MatharuCyro AlbuquerqueNawab KhanCyrus MotamedNawaf Al-MaharikCzesław AdamiakNawaz HackCzesław MarciszNay Min HtunD. KalaivananNayeli Torres-RamírezD. M. Reddy PrasadNazam AliD. S. PrabakaranNazanin MojtabaviDa GaoNazif ElaldiDa Kysha P. MooreNazim NassarDa-An HuhNazli FarajzadehDabkienė VidaNazli KoseogluDa̧browska-Galas MagdalenaNdawula Junior CharlesDac MaiNdidzulafhi Selina RaliphaswaDadi ZhangNeal DoranDaejin KimNeamat Hassan AbubakrDafeng ZhengNeamtu BogdanDafna HalperinNebojša D. ZdravkovićDafna S. Rubin-KahanaNebojša JasnićDafni PetkouNebojša PavlovićDagang LiNebojša PetrovićDagmar KutsarNecmettin Fırat ÖzkanDagmar PavlůNeda VdovićDagmara KociubaNedelcut NelidaDagmara StangierskaNedim DurmusDagmara Tchorz-TrzeciakiewiczNedim TutkunDagoberto Torres-FlórezNeelam PunjaniDahai ZhaoNeelima SatyamDahye ParkNeeraj BhandariDai D. NghiemNeeraj JainDai PuNeeraj PuroDai Whan AnNeeraj Singh ThakurDaiana A. Quintiliano ScarpelliNeeru GuptaDaiani Modernel XavierNeetu TalrejaDaichi SugawaraNeftali NuñezDaichi SumiNefyn WilliamsDaichi YamashitaNegin A. RiaziDaiji EndohNeha A. ThakreDaiji NagayamaNeha MehtaDaisuke AkibaNeha ShroffDaisuke KatoNeil D. ClarkeDaisuke MachidaNejc HorvatDaisuke MatsushitaNejc KozarDaisuke MurakamiNejc PikoDaisuke NishiokaNejc UmekDaisuke SatoNejka PotočnikDaisuke SonNektaria PalaiologouDaisy FanNektarios KalyvasDaisy VolmerNeli Kinga OlahDaiva E. NielsenNelia P. AmadoDaiva JuknelienėNélio VeigaDaiwei YaoNelli LyyraDaizhong TangNellie TsipouraDaizy R. BatishNelly Molina-FrecheroDajana Kučić GrgićNelly Schulz-WeidnerDakota Aaron McCartyNelson Andrade-GonzálezDalbert Matos MascarenhasNelson Andrew PetersonDale A. DickinsonNelson DuarteDale J. AllenNelson M. AnayaDale T. SnauwaertNelson SassDale TerasakiNemanja TomićDalea M. BukharyNemany HanafyDalia AlmaghaslahNemesio Villa-RuanoDalia AntinienėNena VukelićDalia El KhaledNenad KoropanovskiDália LiberatoNenad KorugaDalia MedhatNengmin WangDalia PerkumienėNenita BukaloDalia StreimikieneNerea Gutiérrez-FernándezDalila BurinNermeen YosriDalila CerejoNermin HasanspahićDalila Laoudj-ChenivesseNesrein HashemDalila ScaturroNessr Abu RachedDalton Pessôa FilhoNestor AsiamahDamao XuNéstor Montalván-BurbanoDami MoonNeven PapicDamián CastañoNeven RicijašDamian CzarneckiNeven S. FučkarDamian FrancisNeven VoćaDamian HudziakNevena KulicDamian LiszkaNevenka MarasDamian MorganNevenka Popović ŠevićDamian NakoniecznyNeves-Amado JoãoDamian PawlikNevzat ÖzgürDamián Pereira-PayoNezha MejjadDamian PiotrowskiNg Chiew HongDamian ZasinaNgan Yin ChanDamiana ScuteriNgar Sze Elsa LauDamiano MeninNgee Sing ChongDamien Burlet-VienneyNgianga-bakwin KandalaDamien CassellsNgoc Dung BuiDamien PeyronnetNgoc Hai VuDamien VitielloNguyen Quang TrungDamien YoungNguyen Vinh KhuongDamir KapetanovićNguyen-Nhu-Y HoDamir PekasNguyet A. NguyenDamir ZubacNhut VoDamjan VlajNi Wayan MasriDamodaran ThenmolyNi ZhangDan Bahadur PalNi’matuzahroh Ni’matuzahrohDan ChenNiall M. H. McLeodDan Cristian RaduNiamh GillDan Iulian AlexeNiamh Ní BhroinDan JonesNiamh O’BrienDan LiNiccolò NirinoDan Mihai RohozneanuNiccolò PersianiDan MillerNichol M. L. WongDan PăunNicholas BelloDan RomerNicholas CaporussoDan SongNicholas D. LawsonDan VoiculescuNicholas DeebelDan WangNicholas DopkinsDan YanNicholas F. BoerDana BadauNicholas FelicioneDana GingaşuNicholas GrubicDana H. Z. WilliamsonNicholas J. ThomasDana HanesovaNicholas JohnsonDana L. AultNicholas K. SchiltzDana LawrenceNicholas OllberdingDana M. PrinceNicholas Peter LawsonDana PerniuNicholas R. JaegersDana PopNicholas ReedDana SitányiováNicholas RolnickDana Teodora Anton-PaduraruNicholas ShenkerDana ZahaNicholas ThompsonDan-Alexandru SzaboNicholas TsounisDan-Cristian DabijaNicholas UttingDane KrtinicNicholas YeagerDane PfluegerNichole NageotteDane WolfNick ChandlerDang-Trang NguyenNick GarrettDanguole SatkunskieneNick M. A. SchubertDanh C. VuNick RuktanonchaiDani MorenoNick SlagelDanica BakoticNicklas NeumanDanica FazekašováNickolas ZallerDaniel A. BoullosaNicky StanleyDaniel A. DarkoNicla NotarangeloDaniel A. GriffithNico ArcillaDaniel AkinyeleNico DraganoDaniel Andrei IordanNico NitzscheDaniel Antonio NarzettiNico UeberschaarDaniel B. YaroshNicola Alberto ValenteDaniel BellNicola BartolomeoDaniel BoegerNicola CantasanoDaniel BoscaljonNicola CapolupoDaniel C. AmaralNicola ChristieDaniel C. HughesNicola DöringDaniel C. MorrisNicola FabrisDaniel Carnieto TozadoreNicola FerraraDaniel Cerqueda-GarcíaNicola FortuneDaniel CookNicola LambertiDaniel CruzNicola LopomoDaniel Dalla TorreNicola LovecchioDaniel Das Virgens ChagasNicola Luigi BragazziDaniel Demétrio Faustino-SilvaNicola MadugnoDaniel DiazNicola MagnavitaDaniel DohNicola MarottaDaniel Dorta AfonsoNicola MondanelliDaniel DuneaNicola MucciDaniel Durán SandovalNicola OrzalesiDaniel DziobNicola PalenaDaniel Espejel-BlancoNicola RainisioDaniel EvansNicola RelphDaniel Ferreira Moreira LobatoNicola SambucoDaniel Filipe AlbuquerqueNicola SheeranDaniel Florin LighezanNicola SponsielloDaniel FuentesNicola TheisDaniel García IglesiasNicola TroisiDaniel GensorowskyNicola ValèDaniel Gónzalez-BandalaNicola ValenteDaniel GurínNicola WatersDaniel HolzingerNicolae BacalbașaDaniel HomocianuNicolae Dan TesloianuDaniel Horna MuñozNicolae GicaDaniel I. ŚliżNicolae GogaDaniel J. G. ThirionNicolae MironDaniel J. ReidenbergNicolae Popescu-BodorinDaniel José BarbosaNicolae-Florin CofaruDaniel KalúsNicolai CraciunDaniel KaszubowskiNicolas Aguilar-FariasDaniel KönigNicolas BabaultDaniel L. PiskorzNicolas BergerDaniel LandauNicolas BourdillonDaniel Lara-CobosNicolas BraultDaniel LichteNicolas BruderDaniel LindsayNicolas GhilainDaniel M. ConnollyNicolas GromikDaniel M. González-PérezNicolas HoertelDaniel M. MasekameniNicolás LibuyDaniel M. SchneiderNicolas LoriDaniel MagnoneNicolas MarineDaniel MaposaNicolas MerveilleDaniel María Lubián LópezNicolas PinsaultDaniel MayNicolas R. DaleziosDaniel MendozaNicolas ScellesDaniel Mensah AnangNicolas SöhlingDaniel MontNicolas StefaniakDaniel N. WarshawskyNicole BarraDaniel NedelescuNicole BobbetteDaniel NovakovicNicole Cieri-HutchersonDaniel O’ShielNicole De VilleDaniel OgundijoNicole KrasDaniel OuyangNicole LevitzDaniel P. JohnsonNicole MeierDaniel P. LongmanNicole SommerDaniel PaplaNicole SugdenDaniel Patiño-GarcíaNicole TraboldDaniel Perez ZarateNicoleta Adriana GeamănăDaniel Perez-MarcosNicoleta AntonDaniel PinillosNicoleta Dorina Racolța-PainaDaniel PotaczekNicoleta DospinescuDaniel Puente FernándezNicoleta Mihaela FloreaDaniel RaffertyNicoleta VrînceanuDaniel RayneNicoletta CeraDániel Róbert SzabóNicoletta De SantisDaniel RoethNicoletta FormentiDaniel Sanz-MartínNicoletta GuerrieriDaniel SaundersNicoletta ZermanDaniel SchulzeNicolette Teufel-ShoneDaniel ScottNicolina CapoluongoDaniel ŚlęzakNicolòmaria BuffiDaniel ŚliżNicos MaglaverasDaniel StoecklinNida AslamDaniel SullivanNidal JaradatDaniel T. L. ShekNidhi ManaktalaDaniel TorassaNidhi SharmaDaniel Torres-LagaresNidhi ShuklaDaniel VainstubNidhi SinghDaniel ValleroNidia M. León-SicairosDániel VéghNiedzielska IwonaDaniel VentusNiels-Peter Brøchner NygaardDaniel W. NixonNien Mu ChiuDaniel WendtNienke H. Van DokkumDaniel WilsonNien-Ming HongDaniel Xin ZhangNieves López-EstébanezDaniel YoukeeNigel D. RobbDaniel ŻmudzińskiNigel GilchristDaniela AnistoroaeiNighat NoureenDaniela ArsenovicNihan Öksüz NarinçDaniela BosiaNii Korley KorteiDaniela BraconiNik Norliati Fitri Md NorDaniela Caetano GonçalvesNika PavlovićDaniela Carmen AbabeiNikhil JaganDaniela CialfiNikhil KhannaDaniela CiobanuNikhil R. GandasiDaniela CraveiroNikhil SatchidanandDaniela DamianiNikita NikitaDaniela De BartoloNikki DunneDaniela DeboneNiklas CederströmDaniela DeufertNiklas WagnerDaniela DoegeNiko MännikköDaniela DumitruNikola KomlenacDaniela FighirNikola MijatovDaniela Guzzon SanagiottoNikola NaumovDaniela HanganuNikola SakačDaniela HartmannNikola StefanovićDaniela HorvatNikola StojanovicDaniela HozborNikola VučićDaniela IsolaNikola ŽajaDaniela Joyce Trujillo SilvaNikola ZaricDaniela KollerNikolai BobylevDaniela LopesNikolai PaceDaniela LuciniNikolaos A. ChrysanthakopoulosDaniela MaranNikolaos AndreatosDaniela MariNikolaos AntonakopoulosDaniela MartiniNikolaos E. TzanakisDaniela MarzioniNikolaos GogonasDaniela MateiNikolaos HasanagasDaniela MedasNikolaos KantartzisDaniela MirandaNikolaos KatzilakisDaniela MiricescuNikolaos PanagiotouDaniela Muntele HendreşNikolaos PellasDaniela Opris-BelinskiNikolaos ProutsosDaniela PajardiNikolaos RemmasDaniela PopescuNikolaos SkandalisDaniela RodriguesNikolaos StalikasDaniela RojatzNikolaos StathopoulosDaniela RossinNikolaos StilianakisDaniela S. Gutiérrez-TorresNikolaos TrihasDaniela Simina StefanNikolaos TsetsosDaniela ŠincekNikolaos VarotsisDaniela Soldić FrletaNikolaos VidakisDaniela Sonja NoschNikolaos VlachadisDaniela TestoniNikolaos ZarasDaniela VarricaNikolaus BeckDaniela ZanettiNikolaus HaasDaniele BorzelliNikolay KanevDaniele CespiNikolay MakishaDaniele ContiniNikoleta G. PrintzaDaniele DetanicoNikolett SzeleiDaniele FattoriniNikolina Jukić PeladićDaniele GarcovichNikos D. FakotakisDaniele GiansantiNikos GkraniasDaniele GrechiNikos SarrisDaniele NucciNikša AlfirevićDaniele PastoriNikša DulčićDaniele PergoliniNiladri BasuDaniele PiscitelliNilakshi T. WaidyatillakeDaniele RonsivalleNilesh WashnikDaniele SilvestriNilgün Ayhan GüneroǧluDaniele UrsoNilima Rajpal KundnaniDanielius SerapinasNilminie RathnayakeDaniella Campelo Batalha Cox MooreNilo RamosDanielle Ferreira Sobral-SouzaNiloofar MohammadzadehDanielle FribergNils HaneklausDanielle FurgesonNilufer OzdemirDanielle GardnerNima Afshar-MohajerDanielle GreenbergNima DadashzadehDanielle HodsonNima KhakzadDanielle L. GelardiNima SanadgolDanielle N. LambertNimi VashiDanielle NunneryNimish GuptaDanielle R. EugeneNimit AgarwalDanielle R. StevensNimsiri AbhayasingheDanielle Regina Da Silva GuerraNina A. BiskoDanielli MelloNina BergerDaniel-Rareș ObadăNina Brkić-JovanovićDaniil N. BratashovNina KrügerDaniil ShmatkovNina Lansbury HallDanijela DomljanNina MendezDanijela GračaninNina NovozhilovaDanijela PetrovicNina S. YanchevaDanijela Puric-MladenovicNina SzczygielDanijela Ristić-MedićNina TumosaDanijela SpasicNina TurmukhametovaDanijela Štimac GrbićNina V. TikunovaDanila de VitoNina Van DykeDanila MerinoNing DingDanilo CoimbraNing LiDanilo Oliveira CarvalhoNing MaDanlin YuNing WuDan-Ning HuNingli WangDanny ColombaraNingning WangDanny HoNipun SainiDanny KesslerNir UzielDanny LumNiraj Prakash JoshiDanping YanNirajan ShresthaDanqi LiNirakar SahooDantong ZhuNirit Tagger-GreenDănuț JemnaNirit Yuviler-GavishDanuta DykNirmal Kumar MohakudDanuta GajewskaNirmala Thomas MyersDanuta Kołożyn-KrajewskaNiroj Kumar SahooDanuta Kosik-BogackaNirupama RamadasDanuta Lietz-KijakNiruwan TurnbullDanuta MiedzińskaNisha BaoDanuta Szkutnik-FiedlerNisha ChoudharyDanuta SzpilkoNishad Thamban ChandrikaDanuta UmiastowskaNishant Raj KapoorDanuta ZarzyckaNishel Mohan ShahDanutė GailienėNishikawa YuichiDany Perwita SariNishimura OsamuDanyelle GreeneNishu GuptaDaowei SunNitasha PhatakDaozong XiaNitesh TewariDaphna Birenbaum-CarmeliNitika KumariDaphne E. PedersenNitin AmdareDaphne KaklamanouNitin KhandelwalDaphne Sze Ki CheungNitzan Peri-RotemDaqian LiuNiva DolevDara LeungNivedita K. GuptaDara TwomeyNives GalićDaragh BradshawNives VidakDaranee JareemitNiyousha HosseinichimehDareen A. OmariNizam Uddin AhamedDaria BiechowskaNizamettin GüzelDaria Elżbieta JaremenNizar B. AlsharifDaria Gorczyca-SiudakNizar TliliDaria MottarealeNkiruka C. AtuegwuDaria Schneider-MatykaNnanake-Abasi OffiongDarie CristeaNnedinma UmeokaforDariga AkashevaNnennaya U. OparaDarija Vukić LušićNoa B. Martín-CófrecesDarijo MiškovićNoah J. GoodallDarina DuplákováNobuaki MoriyamaDarina PetrovskyNobuhiko TanakaDarinka FakinNobuhiro IshidaDario BabićNobuhiro OobaDario BacchiniNobumitsu TsunematsuDario BruzzeseNobuo YoshinariDario CalafioreNobutaka HirookaDario Di GiuseppeNobutaka KuriharaDario FajNobuto NakanishiDario FrisioNoe B. MercadoDario GenoveseNoe Baruch TorresDario LasićNoel CarrollDario LipariNoel Marcen-CincaDario ManaraNoel VellaDario MaradinNoelia Amador-FernándezDario MirabelliNoelia BelandoDario MonzaniNoelia Durán-Gómez.Dario MusolinoNoelia María Martín EspinosaDarío Salguero GarcíaNoelia S. Bedoya-PeralesDario SiniscalcoNoelia Santamaría-CárdabaDariusz BazalińskiNoemi GiannettaDariusz BorońNoemí Moreno-SeguraDariusz DrążkowskiNolan C. TolosaDariusz GraczykNoleen ChikoworeDariusz KałkaNoman IqbalDariusz KotlȩgaNoor JahanDariusz KrokNoor KazimDariusz KuszNoor Ullah KhanDariusz ŁuszkiewiczNoor ZamanDariusz MoslerNooshin SohaniDariusz NowakNopadon KronprasertDariusz PyzaNopakoon NantsupawatDariusz StasiakNoppol ArunratDariusz TomkiewiczNor Aini JamilDariusz TworzydłoNor Azlida Mohd NorDariusz WłodarekNor Norazlin KamalDariusz Wojciech MazurkiewiczNor Rohaizah JamilDarja KubečkováNora EstgfaellerDarja Maslić SeršićNora J. BirdDarja ŠemrovNora M. ChapmanDarja TopolšekNora M. LaskowskiDarko BožanićNora MunguiaDarko MekterovicNora RepoDarko ModunNora Suleiman-MartosDarlingtina EsiakaNora SydoDarna L. DufourNóra SzigetiDarrah SleethNorazian Mohamed NoorDarrell O. RickeNorazwina ZainolDarrell P. WheelerNorbert EbertDarren HaywoodNorbert GrafDarren RancoNorbert SiposDarren RussellNorbert TripoltDarryl PlecasNorbert ZolekDartagnan Pinto GuedesNoreen GleesonDaryl JonesNorfaridatul Akmaliah OthmanDaryl MahonNorfazilah AhmadDaša StupicaNorhasnida ZawawiDastan BamwesigyeNorhayati RamliDat Tien DoanNoriaki MaedaDat TranNoriel CalaguasDave GilmoreNorihiko NaritaDavid A. HolcombNorihiro SatoDavid A. WeisblatNorintan Ab-MuratDavid A. WinterNorio SogawaDavid Alarcón-RubioNorio YamamotoDavid Alberto Rodríguez MedinaNoritaka KatataniDavid AlmenarNoriyoshi UsuiDavid Almorza-GomarNoriyuki SuzukiDavid Alonso GonzálezNoriyuki YamagiwaDavid AnderssonNorizzati AmsahDavid AparisiNorm ArcherDavid ArditiNorma Angélica Moy-LópezDavid AshleyNorma F. KanarekDavid Baeza MoyanoNorma Latif FitriyaniDavid BarkerNorma OviedoDavid BarneyNorman M. SpivakDavid BaronNorman R. WilliamsDavid BerbrayerNormarie Torres BlascoDavid BerriganNorrie PearceDavid BeversdorfNorshah Afizi ShuaibDavid Bienvenido-HuertasNorzalita AzizDavid BoansiNoto Susanto GultomDavid BotequimNoula GibsonDavid BroomNounagnon AgbanglaDavid Bruce ConnNour DoumaniDavid BuitenwegNour KhlaifatDavid BurghesNoureddine BouaïchaDavid C. MarchantNoureddine LakouariDavid C. MohrNowakowska ElżbietaDavid Cabello-ManriqueNqoba TsabedzeDavid CampbellNtsieni Stella MashauDavid CarpenterNúbio Gomes FilhoDavid CatelaNuerla AilijiangDavid ChelazziNuggehalli M. RavindraDavid Chien Liang KuoNuha A. El-SharifDavid CingranelliNunes Farinha Carlos ManuelDavid ClabornNuno Alexandre Ribeiro CostaDavid CokerNuno AmaroDavid Colomer-PovedaNuno CoutoDavid ConversiNuno CrespoDavid CooperNuno Domingos GarridoDavid CórdovaNuno DurãesDavid CovellNuno GarridoDavid CromptonNuno HermannDavid De FranzaNuno JorgeDavid Delgado-GómezNuno LavadoDavid E. HoganNuno Manuel Da CostaDavid E. JacobsNuno MartinsDavid E. MandelbaumNuno Miguel Barateiro Gonçalves SilvaDavid EisenmanNuno Miguel Castanheira AlmeidaDavid EllwoodNuno MoraisDavid EscuderoNuno S. OsórioDavid Falcón MiguelNuno V. BritoDavid FanfaniNunzia CapobiancoDavid G. SmithardNunzia IaccarinoDavid Galan MadrugaNunzia LinzaloneDavid GallagherNunzia NappoDavid Gallar HernándezNunzio CirulliDavid GeldmacherNunzio Di NunnoDavid GolightlyNur BelkayalıDavid González-BarrioNurdan TozunDavid GrynspanNurettin Dorukhan SerginDavid HalaNurhayati Abdul MalekDavid HarrisonNuria ArísDavid HenryNuria Castro-LemusDavid HerbertNuria De PedroDavid Hernández HerreroNúria FabrellasDavid Hung-Tsang YenNuria FiolDavid HunterNuria Martin RomeroDavid InglebyNuria Padrós-FloresDavid J. BentleyNuria Rodríguez-LópezDavid J. ChesireNuria Sánchez-LabracaDavid J. KukulkaNursanti AnggrianiDavid J. O’ConnorNursyazwani Mohd FuziDavid JolleyNurten AkgünDavid KranjacNuruddin UnchwaniwalaDavid KrantzNurudeen Olalekan OlosoDavid KriebelNurul Azita Bt SallehDavid KumpNurul Hawani Binti IdrisDavid Kyle LatinisNurul Mohammad ZayedDavid LabatNuruol MohdDavid LarsenNuttanan WichitaksornDavid LimNuttavikhom PhanthuwongpakdeeDávid LíškaNuvia M. SaucedoDavid Llaneza-SuarezNyamongo OnkobaDavid Lopez-CarrNyoman SutarsaDavid Lozano-PaniaguaNyong Princely AwaziDavid LucasNyuk Ling MaDavid M. PoppersO. Roger AndersonDavid MalloyO’neil W. GuthrieDavid ManninoOak-Hee ParkDavid Manzano SánchezOana BengaDavid Marín-GarcíaOana Cella AndreiDavid MartinOana Cristina PopoviciDavid MartinezOana DanilaDavid Martínez-IñigoOana IonDavid McDaidOana LucaDavid McDowallOana SandulescuDavid MhlangaOana SăndulescuDavid MoleroOana-Ramona LobonțDavid Moreno MolinaOana-Ramona SocoliucDavid MorrisObdulia Monteserín-AbellaDavid NatcherObianuju E. Okeke-UzodikeDavid NelsonOcean ThakaliDavid O. IrvingOctav Marius RussuDavid O’SullivanOctav Sorin CandelDavid OcónOctavian AndronicDavid OharaOctavian DospinescuDavid OrmandyOctavian EnciuDavid Ortiz de ZárateOctavian G. DuliuDavid Pascoal RosadoOctavian MoldovanDavid Pastor-EscuredoOctavian Tudorel OlaruDavid PearsonOctavio Dublan-GarcíaDavid Perez BarbosaOctavio Vázquez-AguadoDavid Pérez-JorgeOdeya CohenDavid PerpetuiniOdon SanchezDavid Pina LopezOfer BaranovitchDavid PinedaOfir MenasheDavid Q. AndrewsOgnjen BrborovićDavid R. S. LeanOgueri NwaiwuDavid Ramiro-CortijoOhsawa SuguruDavid RhodesOihana AristondoDavid Rhys AxonOimahmad RahmonovDavid Rodriguez-AriasØivind BerghDavid RosenbaumOkan GurbuzDavid S. FavreOkechukwu ChukwudehDavid S. McKinseyÖkördi RékaDavid S. WeberOksana Alexandrovna KlimanovaDavid SalmanOksana PolinkevychDavid Sanchez-MolinaOksana ZininaDavid Sanz-RivasOlaf Chresten JensenDavid SchuermannOlaf GefellerDavid SchultzOlaf KrauseDavid SchwebelOlajide O. FadareDavid ShawOlalla García-TaiboDavid ShonkOlalla Sáiz-VazquezDavid SkvarcOlamide Timothy TawoseDávid SzakosOlawole FawehinmiDavid T. MarshallOlchowik GrażynaDavid Taylor HendrixsonOleg KaplińskiDavid ThorpeOleg SilyukovDavid TurbowOleg YakhinDavid V. McQueenOleg ZhdaneevDavid Varillas-DelgadoOleksii SkorokhodDavid VaronOlena BilousDavid VérezOlena ChygrynDavid VernezOlena Shelest-SzumilasDavid WatsonOlena VovkDavid WaynforthOlesya NazarenkoDavid Xavier MárquezOlga A. AzarovaDavide AsnicarOlga Alexandra Chinita PirrolasDavide BerardiOlga BelykhDavide BizzocaOlga Bruna-RabassaDavide CostaOlga Czerwińska-LedwigDavide De CiccoOlga De Cos GuerraDavide DonnerOlga DergachevaDavide FerorelliOlga DolgovaDavide GoriOlga E. IvanovaDavide MarinoOlga FajardaDavide MauriOlga Hecmarie Meléndez-FernándezDavide MoiOlga IakimenkoDavide MoroniOlga JensenDavide OrsiniOlga KolotouchkinaDavide PapurelloOlga L. VoroninaDavide Settembre BlundoOlga ŁawińskaDavide Shingo UsamiOlga López GuarnidoDavide SistiOlga López TorresDavide SoglianiOlga M. BazanovaDavide StaedlerOlga MaganoDavide VurroOlga MitinaDavit MarikyanOlga Navarro MartínezDavit PipoyanOlga PalusciDavood ZareOlga PanfilovaDavor CaricOlga PetrakovskaDavor ManceOlga PilipczukDawid BedlaOlga RadulovićDawid KoźleniaOlga RedinaDawid MadejOlga RibeiroDawid MarciniakOlga Roldan ReoyoDawid PrzystupskiOlga RousouDawid StormanOlga ScudieroDawid SzpakOlga ShevchenkoDawid SzwedowskiOlga StrizhitskayaDawn A. Goddard-EckrichOlga SysoevaDawn HoogeveenOlga TararovaDawn M. FallacaraOlga TsivilevaDawn PickeringOlga ValentimDawnyéa D. JacksonOlga Warszawik-HendzelDaxin LiangOlga YakovlevaDaya GuptaOlgica NedicDayane AlvaresOlha PopeloDa-Yuan ChenOlihe OkoroDe Anne TurnerOlimat HmoudDe DasguptaOlimpia LaiDe ZhaoOlimpia PinoDean CooleyOliva Maria Dourado MartinsDeana B. DavalosOliver Díaz LopezDebajit DeyOliver F. ShyrDebajyoti BoseOliver FaustDebaleena MajumdarOliver KalpakDebanjan DasguptaOliver Mendoza-CanoDebashis DuttaOliver Ramos ÁlvarezDebashish SenguptaOliver StefaniDebasis BandyopadhyayÓliver Valencia De PabloDebasree Das GuptaOliver VogelDebatosh DasOliver WeigeltDebayan DeyOliver WilsonDebbie FaulknerOliver Winston LaytonDebbie HagerOlivera DolićDebbie JansonOlivera Mitrović AjtićDebbie LingOlivia CurzioDebbie OlsonOlivia H. TousignantDebbie S. FetterOlivia McDermottDebdeep MitraOlivia RealdonDebmalya BarhOlivia RemesDebo DongOlivia ZinDebojyoti MoulickOlivier AromatarioDebora ColangeloOlivier BoninDebora Farias Batista LeiteOlivier EscaffreDebora PorriOlivier GarraudDébora Regina Schneider LocatelliOlivier Piedfort-MarinDebora RosaOlivier PierreficheDeborah BackusOlivier PourretDeborah CottonOlivier Rémy-NérisDeborah GentileOlivier ReynardDeborah GlasoferOlivier RolinDeborah GrayOliwia Gawlik-KotelnickaDeborah H. L. NgOllie Yiru YuDeborah IresonOlli-Pekka HilmolaDeborah J. GoodOlof ÖstergrenDeborah J. KeszenmanOlu AwosogaDeborah JumpOlubukola OduntanDeborah M. WinnOlugbemi ArokeDeborah Oyine AluhOlúgbémiga T. EkúndayòDeborah PanepintoOlumuyiwa James PeterDéborah Sanabrias MorenoOlusegun Gabriel FawoleDeborah ScharfOlusola AyeleruDeborah StanistreetOlutosin TaiwoDebra JacksonOluwafemi OluwoleDebra JosephOluwagbemiga Dade MatthewsDebra MacKenzieOluwarotimi Williams SamuelDebra RickwoodOluyomi OsobajoDebra StroineyOlwenn MartinDebra Wetcher-HendricksOm KurmiDębska MałgorzataOm Prakash ChoudharyDe-Chih LeeOmanjana GoswamiDechristian BarbieriOmar AlibrahimDecky AspandiOmar AlzubiDedy AntonyOmar DurrahDeena SnokeOmar El HibaDeepa AcharyaOmar Farookh PinjariDeepa AlexOmar GeloDeepak BalamuraliOmar HahadDeepak BalramOmar Hussein SalmanDeepak KotiyaOmar JanehDeepak KumarOmar KujanDeepak Kumar KhajuriaOmar M. ShamiehDeepanjan PaulOmar S. AsfourDeepika AroraOmayma A. EldahshanDeepika KoundalOmbretta ParaDeepika YadavOmer AgaDeepti BehlOmer Hosni Mohamed IbrahimDeepti ChopraOmer UnsalDeepti SharmaOmid AkhavanDeidre HurseOmid MirmosayyebDejan M. VasovićOmkara Swami MuddinetiDejan MiljenovićOmneya AttallahDejan MirakovskiOmni CassidyDejan NikolićOmori YasutakaDejana JakovljevićOmotuyole AmbaliDejian LaiOnay Burak DoganDejian YuOndrej HanušovskýDelfín Ortega-SánchezOndrej JiricekDelia BlayaOndrej StopkaDelia Mirela TitOndrej TichyDelia ȘtefenelOng Ardvin Kester SDelia VîrgăOng SayhowDelicia Shu Qin OoiOng SongDeling YuanOnn Chiu ChuenDelky Meza-ValderramaOnno BouwmeesterDelphine GirardOnofrei PavelDelva ShamleyOnur ErgenDemba SarrOnur GuvenDemet Kaya AktaşOoi Guat SeeDemetra AntimisiarisOpeyemi Oladapo-ShittuDemetrio Antonio ZemaOpeyemi R. SalauDemetrio LozanoOPhir NaveDemétrio MatosOren LapidDemetrios A. ArvanitisOrestis SchinasDemetrios J. HalazonetisOrges LenaDemitrios VyniosOrhan HacihasanogluDemóstenes Zegarra RodríguezOrhan KoçakDenis BaranovskiiOrhan ÖzdemirDenis Bernardeau-MoreauOriana MottaDenis BourgeoisOriane SarrasinDenis ChênevertOrietta SeguraDenis LazarenkoOriol Abellan-AynesDenis MuhangiOrish E. OrisakweDenise ConroyOrjena ŽajaDenise CorridoreOrkideh GharehgozliDenise DillonOrlanda CruzDenise EvansOrlando CiminoDenise M. ClaiborneOrlando DuránDenise MacMillanOrly SaridDenise Paschoal SoaresOrna Baron-EpelDenise PergolizziOrna Braun-LewensohnDenise TaylorOrna RegesDenise Vieira TravassosOrnella TarolaDenise VociOrnwipa ThamsuwanDenisse ParraOrsola GambiniDennis GörlichOrsolya KissDennis GrevensteinOrsolya NémethDennis GrunwaldOrsolya Zsirka-FónagyDennis LevinsonOsama Anwer SaeedDennis MurphyOsama D. SweidanDennis NiebelOsama E. MansourDennis RosenbergOsama SohaibDeodato AssanelliOsamu GotohDeogratius LuyimaOscar BergensDeokjong LeeÓscar CervillaDeok-Oh WooOscar CrisafulliDepeint FloreOscar De La Torre-TorresDependra BhattaOscar F. AranedaDeQuincy A. LezineOscar Fraile-MartinezDerek B. OienÓscar Gavín-ChocanoDerek G. ShendellOscar Hengxuan ChiDerek LarkinOscar Herrera-CalderonDerek P. EvansOscar Martinez De QuelDerek SmolenskiOscar ModestoDerk BrouwerOscar NavarroDerrick R. StowellOscar Núñez EnríquezDerrick SsewanyanaOscar Salomó-CollDer-Shin KeOskan TasinovDerya AklemanOskar A. PalaciosDerya BirantOskar JonssonDeryk G. JonesOskars KalejsDesale HabtzghiOsman AktaşDesirée Mena-TudelaOsman ImamogluDesirée Valera-GranOsman M. KaratepeDesmond AgboadaOsmar Antonio Jaramillo-MoralesDespina CochliouOsmel La-Llave-LeonDespina KoualiOsmindo R. Pires JúniorDespina Maria BordeanOsnat BashkinDespoina IatridouOssi PesämaaDespoina PapadopoulouOsslan Osiris Vergara VillegasDespoina-Simoni AlexiouOsvalda De GiglioDesu ChenOsvaldo F. A. LoquihaDeulle MinOswaldo Sinoe Medina-GómezDev RoychowdhuryOswaldo Villena CarpioDevan M. HawkinsOthman A.M. OmarDevender Kumar SainiOthman AlmashaqbehDevendra KumarOthman Bin MohdDevidas MenonOtilia ClipaDevin M. McCauleyOtilia MantaDevinder Mohan ThappaOtilia TudorelDevis BenfaremoOtmane AzeroualDevlina Das PramanikOtsuyama KensukeDevojit Kumar SarmaOttavia FerraroDevon McaslanOttmar HerchenröderDevrim Can SaraçOtto LamDewi HerawatyOtto RuokolainenDexing ZhangOurlad Alzeus TantengcoDeya XuOussama BaaloudjDezhong KongOussama SaidiDezhong YangOuti Ilona KansteDhafer MezghaniOvergaauw PaulDhananjay MankarOvidiu BordeanDhananjay YadavOvidiu Popa-VeleaDhara SharmaOvidiu TitaDharmalingam MalaOwen J. KellyDharmendra RajputOyelola AdegboyeDharmendra SinghÖzcan ÖzekeDhaval PauÖzgü İnalDhavamani SugasiniÖzgül Yılmaz KaramanDhaya KanthavelÖzgür Arslan-AyaydinDhirgham Alobaydiözgür BostancıDhruba PathakOzgur EkenDhruv DesaiOzgur KasapcopurDhuha WazqarÖzgür KuzukıranDi HuÖzkan UǧurluDi JinOznur BilacDi WangOzren PolašekDi’an FangÖzüm Yuksel TunçyürekDian HandayaniP. P. KrishnapriyaDian ShengP.M. Durai Raj VincentDian YuPablo A. Cantero GarlitoDiana Borges DuartePablo A. HenríquezDiana CelebańskaPablo A. LizanaDiana CenariuPablo Caballero-PérezDiana Cunha-ReisPablo Cabrera-BaronaDiana E. ValeroPablo Camacho LazarragaDiana Elena DumitrașPablo Campo-PrietoDiana EmangPablo Cervera-GarviDiana GagliardiPablo EscandónDiana Gil-GonzálezPablo Ezequiel Flores KanterDiana Lea BaranovichPablo FaríasDiana Luminita ManolescuPablo G. Del RíoDiana LungeanuPablo Galvez-RuizDiana LupuPablo Garrido-PradaDiana M. DiNittoPablo González FrutosDiana MacKayPablo Henrique Dos Santos PicapedraDiana Maria VranceanuPablo Hernandez-LucasDiana Mariana CocârțăPablo J. AlonsoDiana Marin SuelvesPablo Jorge Marcos-PardoDiana MartinsPablo MansillaDiana MiconiPablo Manuel Millán-MillanDiana MindrilaPablo Martinez-CamblorDiana MoangaPablo MirallesDiana NicaPablo Monteagudo ChinerDiana Pacheco BarzalloPablo Ruisoto PalomeraDiana PoliPablo Sánchez LéonDiana Popa-AndreiPablo Thomas-DupontDiana RabadjievaPablo Tomas-CarusDiana RichterPablo Usán SupervíaDiana Rodríguez-HurtadoPablo Valdés-BadillaDiana RusuPablo VergaraDiana SoeiroPablo Vidal GonzálezDiana TsoyPablo Villalobos DintransDiana UrbanoPa-Chun WangDiana-Ionela StegarusPadulo JohnnyDianchen ZhuPage D. DobbsDiane DepkenPaige Padgett WermuthDiane JarvisPaiman AhmadDiane Lillo-MartinPaitoon PimdeeDiane Lynn SmithPäivi JokelaDiane M. DaubertPak Hin Hinson CheungDiane MagranePakieli H. KaufusiDiane MahoneyPal MartonDiane McDaniel RhodesPalak PatelDiane RyanPalanikumar BalasundaramDiane-Gabrielle TremblayPalanisamy NallasamyDian-Jeng LiPalanisamy RavichandiranDiannan LuPalina PrysmakovaDianne GardnerPaloma Alonso-StuyckDianne SheppardPaloma De Almeida RodriguesDianne WästerlundPaloma Echevarría-PérezDibya PandaPaloma Malagon LopezDidar UcuncuogluPaloma MassóDidhiti MukherjeePam Dachung LukaDidi SurianPam Factor-LitvakDidier G. LeiboviciPam JarvisDiego Alexander Escobar GarciaPamayla DarbyshireDiego Alexandre Alonso-AubinPamela A. Harris-HamanDiego Alonso-FernandezPamela BartloDiego BarcellosPamela D. SwanDiego De Souza SardinhaPamela Di GiovanniDiego Dos Santos FerreiraPamela S. TsangDiego Fernández-NóvoaPamela TozzoDiego FettermannPan WangDiego Gavilán-MartínPan ZhangDiego Giulliano Destro ChristofaroPanagiota G. PietriDiego Gomez-BayaPanagiota GaletsiDiego Guimarães Florencio PujoniPanagiotis F. LiaparinosDiego ManzoniPanagiotis G. HalvatsiotisDiego Muñoz MarínPanagiotis GkriliasDiego NogueiraPanagiotis N. TsikourasDiego Peruchi TrevisanPanagiotis PapazotosDiego QuirogaPanagiotis PeitsidisDiego RaimondoPanagiotis PentarisDiego RomeroPanagiotis PetrakisDiego Sales-leridaPanagiotis TheofilisDiego Soto GarcíaPanagiotis TsimeasDiego Tlapa-MendozaPanagiotis ZikosDiego VasconcellosPanaoura AretiDien WuPanayiotis DimitriadisDierdra BycuraPanda SantoshDiesch TamaraPandji Wibawa DhewantaraDieter FinkPang-Yun ChouDieter GertenPaniz JasbiDieter HohenwarterPankaj BhattDietrich K. HofmannPankaj DadheechDietrich Schmidt-VogtPankaj KumarDigiulio LaurenPankowski DanielDih-Ling LuhPanmela SoaresDihogo De MatosPanpan ZhouDilaram AcharyaPanrawee RungskunrochDilaver TengilimogluPantaleo GrecoDiletta Francesca SquarzantiPantea IzadiDiletta GazzaroliPanteha FarmaneshDilfuza EgamberdievaPantelis KourosDilip K. NagPantelis ZempekakisDilipkumar R. PatelPaola AngeliniDillon ChrimesPaola BerchiallaDillon MintoffPaola BianchiDimas AdiputraPaola BianucciDimiter Georgiev VelevPaola BorellaDimitra KoumakiPaola CozzaDimitra Rafailia BakaloudiPaola DamianiDimitri PoddighePaola Di MascioDimitri SchuurmanPaola FerriDimitrina MitevaPaola FossaDimitrios G. KonstantinidesPaola GandiniDimitrios KantasPaola ManducaDimitrios KasselimisPaola ManfrediDimitrios KoilakosPaola MasottiDimitrios KotsirisPaola PalestiniDimitrios LampakisPaola ParenteDimitrios MichelogiannakisPaola PassafaroDimitrios NikolopoulosPaola PetrosinoDimitrios PanagopoulosPaola QuaresimaDimitrios PapadopoulosPaola ScarcellaDimitrios PapagiannisPaola ZanchiDimitrios PoulimeneasPaolina CroccoDimitrios SioutopoulosPaolo AbondioDimitrios StamovlasisPaolo BoldriniDimitrios TsarapatsanisPaolo BorattoDimitrios VlachodimitropoulosPaolo BozzatoDimitrios VlachopoulosPaolo BrustioDimitrios ZikosPaolo CandioDimitris BeliasPaolo CapparèDimitris EvangelopoulosPaolo CarosiDimitris G. MandalidisPaolo CasadioDimitris KarampatzakisPaolo CastelliniDimitris KaskaoutisPaolo CastigliaDimitris KikidisPaolo CeciDimitris LambrouPaolo ContieroDimitris PapageorgiouPaolo D’AquilaDimitris TatsisPaolo De BlasiisDimitris TsoukalasPaolo Emilio SantoroDimitris VoudourisPaolo FagoneDimity CrispPaolo Francesco ManiconeDimosthenis ChochlakisPaolo GagliardiDimosthenis KotsopoulosPaolo GargiuloDina AbdellatifPaolo IovinoDina Di GiacomoPaolo LauriolaDina HuangPaolo MaranzanoDina IzenstarkPaolo Maria CongedoDina PetrashovaPaolo MartellettiDinand EkkelPaolo MeneguzzoDinesh Kumar VermaPaolo MontuoriDinesh RokayaPaolo OrsariaDing WangPaolo PapaDingde XuPaolo PascoloDingding XiangPaolo PastorinoDing-Han WangPaolo PeregoDing-Hau HuangPaolo PesceDingmei ZhangPaolo PudduDing-Quan NgPaolo RavioloDinh Dung NguyenPaolo Tranquilli LealiDinil PushpalalPaolo TrerotoliDinithi PeirisPaolo ValerioDiogo Alexandre Martins CoutinhoPapp-Zipernovszky OrsolyaDiogo Gomes Da SilvaParag MaruDiogo Guedes VidalParaskevi TrivilouDiogo Luís MarquesParastoo TarighiDiogo MoraisParavee ManeejukDiomo MotubaParbeen SinghDionysia PanagouliaParco M. SiuDionysios C. VorosParés-Matos ElsieDionysios TafiadisPargin BangotraDiosey Ramon Lugo-MorinParham PahlavaniDiotima ChattorajParichoy Pal ChoudhuryDiptonil BanerjeeParisa GazeraniDirga LamichhaneParisa Moll-KhosrawiDirk AerenhoutsParivallal ArumugamDirk H. R. SpennemannPark DaejunDirk Hueske-KrausParomita SarbadhikaryDirk MöllerParsa ArbabDirk W. LachenmeierPartha Pratim RayDivanizia Do Nascimento SouzaParthasakha DasDivya GopinathParthasarathi SubramanianDivya NairParthasarathy A. MadurantakamDivya PrakashParthiban SubramanianDivya SahuParveen RewriDivya SinhaParvin GhafarianDivyapriya GovindarajPascal BauerDjanilson Barbosa Dos SantosPascal IzzicupoDjihed BerkoukPascal LambertDjordje G. JakovljevicPascale Besse-HogganDmitri ShchekochikhinPascual Herrera-GrimaldiDmitri SobolevPasi FräntiDmitrii ShekPasqua Anna QuitadamoDmitriy IvashchenkoPasqua Irena SciancaleporeDmitry A. RubanPasquale AuricchioDmitry FrankPasquale CuccoDmitry S. KosyakovPasquale Fabio ProvenzanoDmitry S. PanfilovPasquale GiungatoDmitry TretiakowPasquale MoneDmitry VlasovPasquale MussoDmitry VorontsovPasquale NapoletanoDmitry Yu. IvkinPasquale StefanizziDmitry ZinoveevPasqualina BuonoDmytro OsiichukPasqualino Maietta LatessaDmytro ZherlitsynPasuk M. MahakkanukrauhDobrea Dan-MariusPatchiya PhanthongDobrin CosminPatel PalakDobromir PressyanovPatrice X. PetitDobromira ShopovaPatricia AnafiDobrotă GabrielaPatrícia BatistaDodiek Ika CandraPatricia BrownellDoǧa KavazPatricia C. García-SuárezDohern KymPatricia CaseyDohoon KooPatricia ChiminDohyung KeePatricia Correa-GhisaysDoina AzoicaiPatricia E. FastDoina TodeaPatricia Faraldo CabanaDolaji HeninPatricia Fernandes TrevizanDolores D. GuestPatricia González-DíazDolores Galindo-RiañoPatricia Grace-FarfagliaDolores Lucía Sutil-MartínPatricia GuarnieriDolores Rando-CuetoPatricia López-GarcíaDolores VillalobosPatrícia Manarte-MonteiroDolors CañabatePatrícia MarquesDomagoj DodigPatricia Martínez García de LeanizDomaszewska KatarzynaPatricia MartinkovaDomen KandutiPatricia Masterson-AlgarDomen VerberPatricia MatsumotoDomen VozelPatricia McKayDomenic MarbaniangPatrícia Moura SáDomenico AielloPatricia Núñez-GómezDomenico AmusoPatricia Otero OteroDomenico CiavarellaPatricia PavónDomenico D’UvaPatricia Pia WadowskiDomenico DavolosPatrícia PiresDomenico De BerardisPatricia QuesadoDomenico IannelliPatrícia Raquel Silva FernandesDomenico MorronePatricia SagaspeDomenico SurianoPatricia Sampedro-PiqueroDomicián MátéPatrícia SanchoDomingo RasillaPatricia Segura MedinaDominic Selorm Yao AmuzuPatrícia SilveiraDominic VilleneuvePatricia SlattumDominik BorawskiPatrícia SoaresDominik DłuskiPatricia SolísDominik ŁagowskiPatricia TerryDominik OlejniczakPatricia VazquezDominik RadzkiPatricia VermeerschDominik SaulPatricia VieiraDominik SikorskiPatricio E. Ramirez-CorreaDominik WawrzutaPatricio Iturriaga-VasquezDominika CichońskaPatricio SánchezDominika DąbrowskaPatrick A. PalmieriDominika DzwonkowskaPatrick Allen RoseDominika GłąbskaPatrick AnglardDominika GuzekPatrick DunnDominika HadroPatrick FlanaganDominika KalankovaPatrick GeraghtyDominika M. PindusPatrick GoodmanDominika MazurkiewiczPatrick GuinnessDominika SalamonPatrick H. CarpentierDominika SkolmowskaPatrick HarnarayanDominika WilczyńskaPatrick J. SheehanDominika ZającPatrick JachyraDominique BicoutPatrick M. PerrigueDominique D. GagnonPatrick Marius KogaDominique J. BicoutPatrick MillerDominique LeporePatrick N. O’MahenDominique MassottePatrick ObermeierDominique PotvinPatrick OrmeDomitilla MagniPatrick PageatDomitille CallonPatrick PalmieriDomnina-Maria RazusPatrick PangDomonkos WettsteinPatrick PoucheretDon DiefenbachPatrick SeylerDon E. AlbrechtPatrick TylerDon Hee LeePatrick YorioDon Thiwanka WijeratnePatrik DridDonaji Jiménez-IslasPatrik ŠimjákDonald A. JurivichPatrizia DanesiDonald B. GiddonPatrizia DorangricchiaDonald DeAngelisPatrizia GazzolaDonald LucasPatrizia HreliaDonald MolonyPatrizia LaurentiDonald N. RobersonPatrizia Lo PrestiDonald OkekePatrizia ProiaDonald R. RoyallPatrizia StefanelliDonald RakowPatrizia TodiscoDonatas AustysPatrizia TortellaDonatella CialdeaPatrizia ZaramellaDonatella Di CorradoPatrizio PetroneDonatella PriviteraPatrizio TratziDonatella ValentePatrizio VanellaDonato MoreaPatrycja Bałdowska WitosDone StojanovPatrycja Grosman-DziewiszekDong AnPatrycja KapczukDong Ho RiePatrycja KleczkowskaDong Hyuk ChunPatrycja KlinkDong Hyun YoonPatrycja LipińskaDong LiuPatrycja OkońskaDong WangPatrycja Romaniszyn-KaniaDong Wook ShinPatrycja ŻegleńDong Woon HanPatrycja ZurzyckaDong ZhongPatryk RzońcaDong-Chan KohPatsie FrawleyDongdong CaoPattanasin AreeudomwongDongdong FengPattanee WinichagoonDongdong WangPatti ParkerDongfang LiPatti-Jean NaylorDongfeng GuPau Loke ShowDong-Hee LeePau Redón LurbeDong-Hee RyuPau Soldevila-MatíasDonghwan LeePaul A. SalamhDonghwan ParkPaul A. SandiferDonghyun KimPaul A. SimonDongjie NiuPaul ArmstrongDongjuan XuPaul ArnaboldiDongkyu KimPaul BălănescuDongliang YangPaul BatchelorDongmei LiPaul BlaschkeDongni YePaul CliftDongwon ChoiPaul CookDongxu ChenPaul CrockerDong-Yeop KimPaul DelfabbroDongying LiPaul DesanDongyoup KimPaul DonahueDonley StudlarPaul E. RappDonn L. MarrinPaul EnglishDonny GerkePaul EnlowDonté T. BoydPaul EnthovenDonya KamravamaneshPaul Eze EmeDora Dodig HundricPaul GeoergDora Isabel Fialho PereiraPaul GrossfeldDora Luz OjedaPaul HennebergerDóra ParóczaiPaul HérouxDora Tomić ReljićPaul J. AllisonDorel BadeaPaul JacobsDorian SkrobekPaul JoyceDorian VerdelPaul KindDorien M. KimenaiPaul MackieDorin GheorghePaul McFlynnDorin IonescuPaul McJarrowDorin TibulcaPaul MetaxatosDorin-Nicolae GheorghePaul OakleyDoris BergenPaul OsmondDoris F. OberlePaul S. AmieuxDoris M. KakuruPaul S. LinksDoris OkePaul SilviaDoris RušićPaul SilvianDoris Y. P. LeungPaul SullinsDorit Hadar-ShovalPaul T. J. ScheepersDorit NitzanPaul TanchioDorja VočanecPaul ValleDorota BabilasPaul Van DonkelaarDorota CianciaraPaul WestermeyerDorota Cygan-SzczegielniakPaula A. OliveiraDorota CzyżowskaPaula AcevedoDorota DiakowskaPaula Alexandra PostuDorota DukarskaPaula AranazDorota GroffikPaula BajdorDorota JegorowPaula Bastida-MolinaDorota KleszczewskaPaula BeneveneDorota KopciuchPaula BlasiDorota KrupaPaula C. NevesDorota Kwiatkowska-CiotuchaPaula ChavesDorota LasotaPaula Clara Ribeiro SantosDorota Maria KilańskaPaula Costa-UrrutiaDorota OrtenburgerPaula Cristina da Silva AlbuquerqueDorota PapciakPaula Cristina Marques MartinsDorota RaczkiewiczPaula Esteban-GarcíaDorota RozmusPaula Felippe MartinezDorota Tichaczek-GoskaPaula FernándezDorota WójcikPaula Fernández-PiresDorota WojniczPaula FerreiraDorota WójtowiczPaula GeigleDorota Wołyńczyk-GmajPaula Ioana MoraruDorota ZyśkoPaula L. GrubbDorothea KesztyüsPaula LamoDorothee AlfermannPaula LorgellyDorothy GrahamPaula MadejónDorothy LucksPaula Manuela Mendes Moleirinho-AlvesDorottya FrankPaula Marie HelmDorret I. BoomsmaPaula Marinho ReisDorte Toudal ViftrupPaula Mello De LucaDoru-Nicolae StanescuPaula MosińskaDoug VogelPaula NuñezDougho ParkPaula Peral GómezDouglas AdamoskiPaula QuinteiroDouglas D. KanePaula RibeiroDouglas J. SpielesPaula Rodríguez-FernándezDouglas KalmanPaula Rodríguez-ModroñoDouglas MyersPaula RoviraDouglas Vieira ThomazPaula Ruiz-HincapieDouglas WalkinshawPaula SheppardDouglas WarnerPaula SilvaDouglas WrenPaula Simplício Da SilvaDounia ElfadilPaula StehrDovrat HarelPaula VagosDragan BjelicaPaula VazDragan BurićPaula WilliamsDragan CockaloPauliina HusuDragan KomljenovicPaulina AdamskaDragan MarinkovićPaulina FariasDragan MaticPaulina HebiszDragan MilicevicPaulina MertowskaDragan PamučarPaulina MetelskaDragan RanđelovićPaulina MisztakDragan WeronikaPaulina Mularczyk-TomczewskaDragana BjelicPaulina PająkDragana DogančićPaulina PobleteDragana DrakulovićPaulina PolkoDragana KomnenovPaulina RuteckaDragana MiličićPaulina SztanderaDragana NikolicPaulina TrebskaDragana VidovicPaulina VanDragana Ž. KrstićPauline A. ThomasDrago JelovacPauline Catherine GillanDrago PupavacPauline JonesDragos ArotărițeiPauline ThomasDragos Catalin JianuPauline TurnbullDragos DarabaneanuPaulius SkruibisDragos Florin LismanPaulius ŠūmakarisDragoș Ioan TohăneanPaulo Alexandre S. Armada Da SilvaDragos SerbanPaulo BritoDragoslav NikezicPaulo CarneiroDrahomíra SpringerPaulo CarvalhoDraško PavlovićPaulo Cesar CastroDrazen CularPaulo de MatosDrazenko GlavicPaulo FavasDrew ScolesPaulo Francisco De Almeida-NetoDrew SmithPaulo FrazãoDries Van GassePaulo GentilDriscoll De VaulPaulo Henrique De Araújo GuerraDrona P. RasaliPaulo InfanteDrozdstoi StoyanovPaulo Jorge NogueiraDrucilla G. RobertsPaulo Jorge PalmaDu ViviennePaulo L. HenriquesDuane C. McBridePaulo MafraDuanyang LiuPaulo Magno Martins DouradoDuarte Henriques-NetoPaulo MartinsDubravka ČerbaPaulo MascarenhasDubravka SladićPaulo MeloDubravko HabekPaulo PereiraDuc Huy NguyenPaulo Reis MourãoDujrudee ChinwongPaulo RochaDulce EstevesPaulo SantosDulce Maria Cairós BarretoPaulo Silva Belmonte-de-AbreuDulce Maria MinayaPaulo Veloso GomesDulce MinayaPaulo VitóriaDumitru Constantin-TeodosiuPaulo ZainiDuncan HillPaunita BoancaDung-Jang TsaiPavan Kumar DhanyamrajuDunja AnđićPavani RangachariDunja NiccaPavel A. RyazantsevDuo Wai-Chi WongPavel DivisDuong Van TuyenPavel H. Lugo-FabresDuraisamy SathyaPavel KepezhinskasDurga Rao GijjapuPavel KrpálekDurmuş KoçPavel RozsívalDursun KırbaşPavel SolopovDušan DimićPavel StefanovičDušan HrabecPavel UkrainskiyDušan JandačkaPavel ZhabyeyevDušan KatunskýPavla VachováDusan MiticPavle BanovićDušan MladenovićPavle RandjelovicDusan SchreiberPavle SpasojevicDusan StuparPavlo LykhovydDušan VopálkaPavlos ArvanitisDusanka BoskovicPavol PechoDushad RamPavol SuloDushyant DamaniaPawan Sirwan FarisDuška GlavašPaweł A. PiepioraDustin SlivkaPaweł BłoniarzDuygu AğagündüzPaweł BorkowskiDuygu GurleyikPaweł BrzustewiczDweepna GargPawel BurandtDyah GandasariPaweł DębskiDyah Wulan AnggrahiniPaweł DobrakiwskiDylan M. JohnsonPawel DobrzanskiDylan MoliniéPaweł DolibogDylan P. CliffPaweł DziekańskiE. Dinand EkkelPaweł GaćE. Wesley F. PetersonPawel GajdzisEarl DuncanPawel GawlowskiEbba OssiannilssonPaweł GrygielEbba SundinPaweł HachajEbenezer BonyahPaweł HydzikEberhard UhlPawel JamrozEbrahim Abbasi OshaghiPaweł KalinowskiEbrahim GhaderpourPaweł KawaEbrahim KouhsariPawel KazibudzkiEbrahim MattarPaweł KrukowEbrahim NorouziPaweł LarionowEbru Ersoy TonyaloğluPawel LinekEbru GencerPaweł LonkwicEbru YıldırımPaweł ŁukowskiEbtesam Al-SuhaimiPawel MacekEckardt JohanningPawel MiotlaEckhard KahlePaweł MiśkowiecEddery LamPaweł MundałaEddie BradleyPawel OlczykEddieson Pasay-AnPaweł P. WłodarczykEdel KeaveneyPaweł PakoszEdelberto Franco SilvaPawel Pawel MiotlaEdeltraut KrögerPaweł PlichtaEdgar CambazaPaweł PrędkiewiczEdgar DalePaweł R. LataczEdgar EA BurnsPaweł RadzikowskiEdgar GutiérrezPaweł RussekEdgar SimulunduPaweł SiudemEdgar Torres-MaravillaPaweł ŚwitEdgar Vazquez-NunezPaweł TylekEdgardo BuscagliaPawel TysiacEdgardo EtchezaharPaweł WańkowiczEdgars EdelmersPawel WegrzynEdina MolnárPaweł WięchEdinson Dante Meregildo RodriguezPaweł WolnyEdit GorliczayPawin PadungtodEdit HermeszPayal GangulyEdit XhajankaPayal ShahEdita FinoPayam BehzadiEdita Rosana WidasariPayam Safavi-NaeiniEdite Teixeira-LemosPayam ShojaeiEdith ClarosPaz Fernández-OrtegaEdith J. AranyPearl ChenEdith Valdez-MartinezPearl Chun ChangEdith Van DyckPearl Pei LiuEdmund Ilimoan YambaPearl SubbanEdmund J. ZolnikPedro Aceituno-AceitunoEdmund TomaszewskiPedro Antonio Martín CervantesEdmundo Bonilla GonzálezPedro Baena-LunaEdmundo Rosales-MayorPedro CantistaEdmunds ReineksPedro CisternaEdna AcostaPedro Cuesta-ValiñoEdna NavaPedro Delgado-FloodyEdna PatatanianPedro Díaz-SimalEdnah MaduPedro Fernandes AnunciaçãoEdnardo RochaPedro FerreiraEdoardo BerettaPedro FonsecaEdoardo BucchignaniPedro GóisEdoardo Gioele SpinelliPedro Gonzalez-MenendezEdoardo PappaianniPedro GullónEdoardo PasquiPedro Henrique Lopes SilvaEdoardo PireraPedro Henrique Triguis SchimitEdoardo RellaPedro Hidalgo LopezosaEdorta Carrascal LekunberriPedro J. BerzosaEdouard CoeugnietPedro Jiménez EstevezEdouard LeaunePedro Jorge CaridadeEdsaul Emilio Perez-GuerreroPedro José AlcoleaEdson Baltazar Estrada ArriagaPedro Juan Tárraga-LópezEdson BustosPedro Juárez RodríguezEdson KieuPedro L. CastroEdson Roberto da SilvaPedro LaxEdson Roberto SantanaPedro LucasEdson Santos Ferreira-FilhoPedro M. CostaEdson TalaminiPedro M. HernandezEduard EdelhauserPedro M. S. M. RodriguesEduard Inglés YubaPedro MagalhãesEduard IsenmannPedro Manonelles MarquetaEduard Moreno-GabrielPedro Manuel Rodríguez-MuñozEduarda Marquesda CostaPedro MarijuánEduardo AlgrantiPedro Marín CarrilloEduardo BernabéPedro MeloEduardo CañetePedro MendesEduardo CarballeiraPedro Miguel Alves Ribeiro CorreiaEduardo CejuelaPedro Miguel Ortega-CabezasEduardo CostaPedro Miguel RodriguesEduardo Cyrino Oliveira-FilhoPedro Miguel SardoEduardo DovalPedro Moreno-BernalEduardo Ferro Dos SantosPedro MucharreiraEduardo Garcia-RicoPedro O. RosselEduardo GomesPedro O. S. Vaz de MeloEduardo Gutiérrez-AbejónPedro ParreiraEduardo Hernández-PadillaPedro Paulo Menezes ScariotEduardo João Ribeiro SantosPedro PellicerEduardo José Fernández RodríguezPedro Pezarat-CorreiaEduardo José Lopes-TorresPedro Plans-RubióEduardo José Veras LourençoPedro RochaEduardo LeitePedro Román-GravánEduardo Lopez-DominguezPedro Santana Sales LauriaEduardo López-MartínezPedro SantosEduardo Moraes SarmentoPedro SerralheiroEduardo MoralesPedro SimõesEduardo NotivolPedro SoaresEduardo OsunaPedro TaulerEduardo PiedrafitaPedro Valdivia-MoralEduardo PimentaPedro Veiga RodriguesEduardo Sánchez-SánchezPeer Van Der HelmEduardo Sandoval-ObandoPeera WongupparajEduardo SanguinetPeerayuth CharoensukmongkolEduardo TolosanaPeggy Pik Kei CHOWEduardo Yoshio NakanoPeggy PolicastroEdurne Elgorriaga-AstondoaPei LiuEdurne Úbeda DocasarPeichao DaiEdvard EhlerPeichao GaoEdvard JohanssonPei-Chi ShaoEdward AsisPei-Fung WuEdward C. WrightPeiheng YuEdward ChiyakaPeihua FuEdward FitzgeraldPei-Ing WuEdward G. NagatoPeikuan Cor LinEdward G. SpilgPei-Lin YangEdward HenselPeilong LuEdward HigsonPeiman GhasemiEdward J. PavlikPei-Rung YangEdward J. TrapidoPei-Shan HoEdward JamesPei-Shu HoEdward LebakaPeitao WangEdward Mark CummingsPeivand BastaniEdward MillsPei-yao ChengEdward R. MaguirePeizhi LiEdward R. NewtonPek Kei ImEdward SutantoPekka TarkkilaEdward TolhurstPelin GülEdward TowerPenelope KalogeropoulosEdwin Andres Villagran MunarPenelope WatsonEdwin Barrios-VillaPeng CuiEdyta BarnaśPeng GuoEdyta CharzyńskaPeng HouEdyta Ewa SutkowskaPeng LiEdyta Janeba-BartoszewiczPeng XuEdyta KardasPeng YangEdyta Krzych-FałtaPeng ZhangEdyta ŁaskawiecPeng ZhaoEdyta MądryPeng ZhuEdyta PasickaPengbiao XuEdyta Roslon-SzerynskaPengbo ChuEdyta Sidorczuk-PietraszkoPenge RobertaEdyta SkwirczyńskaPengfei SunEdyta SpychałPenghong GuoEdyta Wrzesińska-JędrusiakPengwei RenEdyta ZbrochPengyu ChenEeswaran RasuPenny AbbottEetu WalliusPer F. L. HallEfaq Ali NomanPer Magnus EhdeEffi Helmy AriffinPer Medbøe ThorsbyEfkleidis KeramopoulosPer Moestrup JensenEfrat Monsonego-OrnanPer NordinEfrat NeterPer-Anders FranssonEfrat RoginskyPere GodoyEfrat ShustPere Lavega-BurguésEfrén Murillo-ZamoraPere Torán-MonserratEfstathios ChristodoulidesPereira Sueli YoshinagaEfstratios PapazoglouPerica MustafićEfstratios-Stylianos PyrgelisPeriyasami GnanaprakasamEfthimios ZervasPerla Jannet Jurado-GarcíaEfthymios MotakisPerman GochyyevEftim ZdravevskiPerminder GulaniEftychia G. KoukkouPernilla GarmyEggi ArguniPernille HolmagerEgidio CelentanoPerola VasconcellosEglantina Lopez-EcharteaPer-Olof ÖstergrenEgoitz AstigarragaPerpetua ModjadjiEgor ZadereevPerri DruenEhsan AghayaniPerri EasonEhsan AsadollahiPerry ZurnEhsan FarsangiPersephone SextouEhsan KhanPershina OlgaEhsan MohammadiPertti HakkinenEhsan SoleimanianPertti PasanenEhsanul Hoque ApuPertti VäisänenEiichi MomotaniPerumal VivekanandhanEija K. LaakkonenPerwira RediEiji ArakawaPeta MurrayEike QuillingPetar AntovEileen M. WankePetar MitićEileen PetersPete DriezenEileen WillisPete KinesEimo MartensPete LampardEinar ThorsteinssonPeter A. BosEinat S. MetzlPeter A. KumbleEinat YehenePeter A. ShapiroEirini OrovouPéter ÁcsEirini SkylogianniPeter AllebeckEitoyo KokubuPeter AmpimEjemai EboreimePeter AntesEkaterina BaranovaPeter BarrEkaterina ChernovaPeter BeckettEkaterina ChernyaevaPeter BerlitEkaterina EzhovaPeter BottenbergEkaterina Fedorovna KolesanovaPeter ChoateEkaterina GlebovaPeter ClarksonEkaterina OrlovaPéter CsécseiEkaterina V. ZubarevaPeter DavisonEkaterini Simões GoudourisPeter DomonkosEko HanudinPeter EdwardsEkram W. Abd El–WahabPeter EibichEl Khalil CherifPeter FiegerEla ErtuncPeter FranklinElad LaxPeter GlavičElahe HosseiniPeter GlynnElaine LumPeter GoodwinElaine Maria Sgavioli MassucatoPeter GrayElaine WethingtonPeter HalajElamara Marama De Araújo VieiraPeter HauserElanagan NagarajanPeter J. AspinallElanor Lucy WebbPeter JankovicElazar Ben-LuluPeter JarcuskaElcio OliveiraPeter Johann SturmEldon R. RenePeter Kenneth HuggardElectra PaskettPeter KerrEleftheria EgelPeter KnightEleftherios GkioulekasPeter KondrlaEleftherios ParaskevopoulosPeter KrizanEleftherios Terry FarmakisPeter LarsonElena A. ProkopyevaPeter LemonElena Adelina TomaPeter LenkElena Alonso-ApertePeter LundqvistElena AmaricaiPeter MachnikElena AscariPeter MccarthyElena BencurovaPeter McGillElena BerezinaPeter McGranaghanElena BernadPeter MeiksinsElena BotezatPeter MeiselElena Bueno-GraciaPeter Michael WardElena BurtsevaPeter Modupi MphekgwanaElena CandigliotaPeter MurrayElena CardilloPeter MwangiElena Carlotta OlivettiPeter NovoszathElena Carrillo-AlvarezPeter NynasElena CarriónPeter Omondi-OchiengElena Cecilia RoscaPeter PaalElena ChianesePeter PikoElena Chover-SierraPeter PutzElena CiguPéter RagályiElena CommodariPeter ReinhardtElena ConstantinouPeter SchmidtElena CyrusPeter Seah Keng TokElena De-La-Barrera-ArandaPeter SmithElena DilonardoPéter TamásElena Dimcheva DimovaPeter UkpokoduElena DruicaPeter V. PaulElena EfremenkoPeter VeranicElena Escolano PérezPeter Von FragsteinElena Fernández-GarcíaPeter W. PiperElena FicoPeter WakholiElena FloritPeter WallnerElena GiacomelloPeter WankeElena GrakovaPeter WattElena GrazianoPeter ZweifelElena GrimacciaPēteris TretjakovsElena HadjimbeiPeter-John MeynellElena IsakovaPeter-Michael SackElena Jiménez-PérezPether JildenstalElena KonovalchikovaPetr BahenskýElena KropinovaPetr KudrnaElena Labajo GonzálezPetr NovákElena Mainer-PardosPetr O. IlyinskiiElena MalushkoPetr RyapolovElena Marbán-CastroPetr SedlakElena Martinez-SanzPetra DurmanElena MazzaPetra LenártováElena MenshikovaPetra Lindholm-LehtoElena MunariniPetra MarkováElena Muñoz-GómezPetra NetterElena N. IkkonenPetra PalátováElena Natera-VillalbaPetra PotměšilováElena NavarroPetra Staufer-SteinnocherElena NikitinaPetra ȘurlinElena OlivettiPetre BretcanElena Perez VazquezPetre BrezeanuElena Piera MontacchiniPetre IonitaElena Pinero-PintoPetri BöckermanElena PopovaPetrick Ramesh A. L. K. PeriyasamyElena RadaPetrina CoventryElena RezusPetrona LeeElena RizaPetronila OlivaElena RoccaPetros BotonisElena RogantePetros MourouzisElena RolandiPey Yee LeeElena SchmidtPeyman Rahimi BorujerdiElena SezennaPezhman MahmoodiElena SidorovaPezhummoottil Vasudevan Nair AsharaniElena Sinkiewicz-DarolPhaneendra K. DuddempudiElena SomovaPhanindra GoyariElena TarcaPhatthranit PhattharapornjaroenElena TenconiPhil AyersElena TombaPhil GendallElena V. EmelyanovaPhil HandcockElena V. GerasimovaPhil RauschnabelElena V. IndukaevaPhilip D. ChilibeckElena V. ProskurninaPhilip GallagherElena VallinoPhilip GreinerElena VasilievaPhilip HarberElena VasilyevaPhilip HazellElena VerticchioPhilip HodgsonElena-Daniela GrigorescuPhilip LewisElena-Laura AntohiPhilip LiElena-Laura UrsuPhilip MayElena-Loreni BaciuPhilip N. CohenElena-Suzana Biriș-DorhoiPhilip SamEleni AndreouPhilip SarajlicEleni G. KatsogiannouPhilip SinnaduraiEleni GeorgakopoulouPhilip TsengEleni Georgios KatsogiannouPhilip WertzEleni KalantidouPhilipp E. SischkaEleni KalliaPhilipp LechnerEleni KortianouPhilipp ÖhlmannEleni KotsiomitiPhilipp Paul LobmaierEleni MakriPhilippe BrouquiEleni PalliPhilippe GerberEleni VousouraPhilippe GourbesvilleEleonora Bielawska-BatorowiczPhilippe GuillaumeEleonora BinattiPhilippe GyanEleonora DesnicaPhilippe J. GiabbanelliEleonora GenovesePhilippe R KoninckxEleonora MarzilliPhilippe RobertEleonora NicolaiPhilippe T. GilchristEleonora NuceraPhillip A. BonneyEleonora PintoPhillip J. MurphyEleonora PorcuPhillip L. WilliamsEleonora RicciPhillis Soek Po TengEleonora SantosPhoebe GriffinEleonora TobaldiniPhoebe Py LamEleonora TopinoPhong ThaiEleonora VirgilioPhrashiah GithinjiEleuterio A. Sanchez RomeroPhu DuongElham AzarpazhoohPhuong The NguyenElham HedayatiPhyllis AnastasioElham HosseiniPhyllis GasparElham KheradmandPhyllis J. KankiEli FeiringPia AnderssonEliã Pinheiro BotelhoPia HenfridssonEliana TassiPia SolinEliane Aparecida CastroPia VidalEliane CastroPia Z. Pace-AsciakElias AliPic MiguelElias DiarbakerliPier MannucciElias M. EliasPiera Anna MartinoElias MpofuPierfrancesco De PaolaElica ValkovaPierfrancesco FrancoElida Nora FerriPierfrancesco MorgantiElide Di-ClementePierfranco LattanziEliete BouskelaPiergiorgio FranciaEliete CaldeiraPierluca PiselliElif Damla ArisanPierluigi CongedoElif Inan-ErogluPierluigi CordellieriElif Melike BildiriciPierluigi DiotaiutiElina SaekiPierluigi PolitiElina WrightPierluigi SaccoElio BorgonoviPierluigi ZoccolottiElio TrusianiPiero MaestrelliElio VenturiniPiero MannaElisa AndrenelliPiero PaladiniElisa BenedettiPiero PavoneElisa BisagnoPiero Vincenzo LippolisElisa Bizetti PelaiPierre DevolderElisa BogossianPierre FaillerElisa CabanaPierre GanderElisa CairraoPierre-Yves Collart-DutilleulElisa CavicchioloPierre-Yves de MüllenheimElisa DelvecchioPierre-Yves RobillardElisa GalofaroPierrick BruneauElisa Hergueta CovachoPierrick LebainElisa Lopez-DoladoPieter Hermanus MyburghElisa MartinelliPieter J. J. SauerElisa MazzaPieter M. E. van GorpElisa MazzottaPietro AsproniElisa PaneroPietro BartocciElisa PratiPietro CastellinoElisa RussoPietro FeltriElisa TomassoniPietro FerraraElisabet ForsumPietro GareriElisabet Huertas-HoyasPietro GrussuElisabet Navarro-TapiaPietro ManiscalcoElisabeta AntonescuPietro MaranoElisabeta BotezPietro MonforteElisabeta Ilona MolnárPietro MontemezziElisabete BorgesPietro MorroneElisabete NevesPietro ParisiElisabete P. SilvaPietro SarassoElisabeth A. StelsonPietro ScicchitanoElisabeth AndriePilaiwanwadee HutamekalinElisabeth AndritschPilar Alfageme-GarcíaElisabeth BalintPilar Andrés-OliveraElisabeth DarjPilar Aparicio-ChuecaElisabeth E. DuursmaPilar Aparicio-MartinezElisabeth Malonda-VidalPilar De Lucas-RamosElisabeth NöhammerPilar LacasaElisabeth PinartPilar Marqués-SánchezElisabeth QuendlerPilar Martin HernandezElisabeth RiegelPilar Martín-EscuderoElisabeth SolanaPilar MatudElisabeth Sornay-RenduPilar Munuera GómezElisabetta BeneventoPilar Nieto-GilElisabetta BertelliniPilar Pérez-RosElisabetta D’AgattaPilar Sánchez-LópezElisabetta FerraraPillai NikhilElisabetta GenovesePimbucha RusmevichientongElisabetta LombardiPin ZhouElisabetta Maria VencoPinelopi VergouElisabetta MariniPing GuoElisabetta PetrucciPing HuangElisabetta PolizziPing LiElisabetta SagonePing RenElisaldo Mendes CordeiroPing SongElisardo Becoña IglesiasPing WangElisavet GrouziPing XiangElisavet StavropoulouPing ZengElisaveta ApostolovaPing ZouElise Arsenault KnudsenPing-Chung LeungElise M.A. SlobPing-Hung HsiehElise RiveraPingjun SunElise VerotPingping LiuEliseo Iglesias-SolerPing-Shun ChenEliseo SciarrettaPing-Tao TsengElisete DiogoPin-Kuei FuElisete MourãoPinto MonicaElismauro Francisco MendonçaPinto-Almazán RodolfoElissa JelalianPinyang YehElissavet Gina GeorgiadouPiotr AschenbrennerElitsa DeliverskaPiotr BąskaElivelton Da Silva FonsecaPiotr BiałasEliza BlicharskaPiotr BilskiEliza ChircanPiotr BolibokEliza NichiforPiotr BórawskiEliza WasilewskaPiotr BorkowskiElizabeth A. GoblePiotr BugajskiElizabeth A. WahlerPiotr BułaElizabeth A. WhalenPiotr ChmielarzElizabeth BarteltPiotr ChomczyńskiElizabeth BrondoloPiotr CzarneckiElizabeth C. PenickPiotr DługoszElizabeth D. NobmannPiotr F. CzempikElizabeth DartnallPiotr FoltynskiElizabeth De GaspariPiotr FormanowiczElizabeth DicesarePiotr GałeckiElizabeth DodgePiotr GawdaElizabeth E. HibberdPiotr GibasElizabeth G. HunterPiotr GolasaElizabeth H. BradleyPiotr GorczycaElizabeth H. LeePiotr GronekElizabeth HatchPiotr GuniaElizabeth HicksPiotr HerbutElizabeth HortonPiotr Holnicki-SzulcElizabeth I. JohnsonPiotr JachimowiczElizabeth J. KingPiotr JaskowskiElizabeth L. SeamanPiotr K. KrajewskiElizabeth McDermottPiotr KaczkaElizabeth Morales-MorenoPiotr KafelElizabeth O’BrienPiotr KarniejElizabeth OcholaPiotr KasprzakElizabeth Opiyo OnyangoPiotr KnyziakElizabeth PierothPiotr KornetaElizabeth R. StevensPiotr KryczkaElizabeth R. WaltersPiotr KułykElizabeth RixPiotr KwiatkowskiElizabeth ShottonPiotr LeszczyńskiElizabeth Solis-PérezPiotr LorensElizabeth StegemollerPiotr MalaraElizabeth SwadenerPiotr MazurkiewiczElizabeth ThomasPiotr MichalakElizabeth TilleyPiotr Mirosław MaŁkowskiElizabeth UnniPiotr MiśkiewiczElizabeth VieiraPiotr MitkowskiElizabeth Ya-Hui ChangPiotr MorasiewiczElizabeth-Barbara TatsiPiotr OdyaElka RadevaPiotr Oskar CzechowskiEl-Kazafy Abdou TahaPiotr PachuraElke KniselPiotr PerlińskiElke MurdockPiotr PetrykowskiElke NaumannPiotr PietrzakElkhan Richard Sadik-ZadaPiotr PoznańskiElkhan Richard ZadaPiotr PróchniakElkin LuisPiotr PrusElla Cohn-SchwartzPiotr RatajczakEllen Amanda StruijkPiotr RaźniakEllen CarlPiotr RegulskiEllen F. MoslethPiotr RomaniukEllen HertzmarkPiotr RoszakEllen J. HahnPiotr SauerEllen KemlerPiotr StaszkiewiczEllen L. IdlerPiotr SulewskiEllen MceachenPiotr SzymaniecEllen SelkiePiotr T. NowakowskiEllen W. McGinnisPiotr TutkaEllie CarmodyPiotr WałdykowskiElliot BenjaminPiotr WlaźElliot LassonPiotr ZapalaElliot M. LevinePippin AndersonEllyn R. MulcahyPitoyo HartonoElmedin BajricPittaluga PaolaElnaz ZeynalooPiya TemviriyanukulEloi SerranoPiyameth DilokthornsakulEloisa Dezen KempterPiyawat KatewongsaEloísa Fernández-OrdóñezPiyush PandeyEloisa Guerrero BaronaPiyush RanjanEloise BiharPiyush VyasEloy GirelaPlamen PanayotovEloy López MenesesPloenthip PuthongkingElpidio Maria GarzilloPoay Sian Sabrina LeeElpidoforos SoteriadesPo-Cheng HsuElpiniki SkoufogianniPo-Chin YangElrasheed Ismail Mohommoud ZayidPo-Ching WangElsa CardosoPodlasińska JoannaElsa López-PintorPo-Fu LeeElsa PegadoPoh Foong LeeElsa RibeiroPoh Heng ChongElsa T. RodriguesPo-Jung HuangElsa VitalePoliana MendesElspeth McInnesPo-Lin PanElton J. R. VasconcelosPolina PopovaElvan ÖçmenPolly Siu Ling ChanElvina ViennetPolly Wai Chi LiÉlvio GouveiaPomi ShahbazElvira BratilăPongrác ÁcsElvira MaranesiPongsakorn SuppakittpaisarnElvira NicaPonnuraj Nagendra PrabhuElwira Gross GołackaPonthip LimlahapunElwira Zajusz-ZubekPooja KamatElyce GreenPoonam VinayamohanElzbieta AntczakPoongavanam Ganesh KumarElzbieta BroniewiczPopescu Aura LoredanaElżbieta BukalskaPoppy WatsonElżbieta CieślaPosen LeeElżbieta CiporaPo-Sheng KoElżbieta Hać-SzymańczukPostan-Aizik DassiElżbieta Jarosz-KrzemińskaPoulamee ChakrabortyElżbieta Krajewska-KułakPoulami DattaElżbieta MacioszekPouya HassandarvishElżbieta NawrockaPrabha RanasingheElżbieta PatkowskaPrabhakar SharmaElzbieta PawłowskaPrabodha PradhanElżbieta PetriczkoPrabuddh MishraElżbieta Poniedziałek-CzajkowskaPrachi ChavanElżbieta RolkaPradeep KumarElżbieta RosiakPradeep Kumar BollaElżbieta Roszko-WojtowiczPradeep RaiElżbieta RyńskaPradeep SinghElżbieta SkorbiłowiczPradeep V. KadambiElzbieta SkorupskaPrado Silván-FerreroElżbieta SobczakPrajakta MasurkarElżbieta Sochacka-TataraPrajish IyerElzbieta SzulcPrajna TripathiElżbieta Wójcik-GrontPrakash BhuyarElżbieta ŻbikowskaPrakash BoominathanElzbieta Zdankiewicz-ŚcigalaPrakit SaingamElżbieta ZyskPramod SubediElzė RudienėPranav BhounsuleElzette KorkiePrasanth GovindanEm BouldPrasenjit MondalEmad KazemzadehPrashant Kumar ShuklaEmad NoaimePrashant SakharkarEmad S. GodaPrashant SharmaEmad SidhomPrashanth RavishankarEman A. MwafyPrashanth Thevkar NageshEman Abu-GharbiehPrasun KumarEman KhalifaPrathip PhantumvanitEman M. OthmanPrathyusha BagamEman MehannaPratima LabrooEman TadrosPraval KhanalEmanuela BianciardiPraveen Kumar NeeliEmanuela CamilliPrawej AnsariEmanuela Gualdi-RussoPreethy S. SamuelEmanuele AmodioPreeti ChauhanEmanuele CannizzaroPreeti DubeyEmanuele CardilloPreetish Kadur Lakshminarasimha MurthyEmanuele CaroppoPrem BhandariEmanuele CassioliPrem Chandra PandeyEmanuele CerrutoPrem Haridas MenonEmanuele FanelliPremachandra WattageEmanuele LeporelliPremanand TiwariEmanuele MicaglioPremnadh Madhava KurupEmanuele RinninellaPrenilla NaiduEmanuele Rocco VillaniPrerna JoshiEmeka E. ObiohaPricila H. MullacheryEmel Karakaya AyalpPricila MullacheryEmel ÖzPrimrose FreestoneEmeline LaurentPrince AtorkeyEmem AnwanaPrince DonkorEmerald HeilandPrince Kwaku AkowuahEmerson GalvaniPrince Michael AmegborEmese Beáta BereiPriscila K. LangeEmi DikaPriscila MarconcinEmiko KamitaniPriscila Nasser De Carvalho KrollEmil DrápelaPriscilla SandersonEmil IsraelPritam BoseEmil Marian ArbănașiPritam Kumar DikshitEmil N. SobolPrithwi GhoshEmilia AlvesPriti GuptaEmilia BiffiPritpal KaurEmília Bigotte De AlmeidaPriya RajagopalanEmilia CirilloPriyadarshini KachrooEmilia GrzegorzewskaPriyam PatelEmilia HermanPriyan DiasEmilia JaneczkoPriyanka AgrawalEmilia KlimaszewskaPriyanka SandalEmilia MadudovaProdip ChouhanEmilia NeagProlay MondalEmilia OgodescuProsanto K. ChowdhuryEmilia P. CollarProsenjit ChatterjeeEmilia SerraPrzemysław BrzyskiEmilia SkupieńPrzemysław CharzyńskiEmilia SzumskaPrzemysław DorożyńskiEmilian ZadarkoPrzemysław DudaEmiliana BomfimPrzemysław Falkowski-GilskiEmiliana GiacomelloPrzemysław KołodziejEmiliano CePrzemyslaw KowalEmiliano DíezPrzemysław LisińskiEmiliano Rodríguez-SánchezPrzemyslaw M. WaszakEmiliano TesoroPrzemysław Mateusz DomagałaEmilie CameronPrzemyslaw WolakEmilie DionnePrzemysław ŻuratyńskiEmilie ValléePrzemysława Jarosz-ChobotEmilien JeannotPsyche LouiEmilija D. JensenPtach WiesławEmilio AndreozziPu HuangEmilio ChiodoPugazhenthan ThangarajuEmilio Crisol MoyaPuhan HeEmilio GarciaPui Fong KanEmilio García-CabreraPui Ho YunEmilio GrecoPui-hang WongEmilio López-ParraPuja KumariEmilio Lozano-AguileraPuneetpal SinghEmilio MartínPuppo FrancescoEmilio Paolo VisintinPura Marín SanleandroEmilio PiccionePuria ParviniEmily BianPurnachandra SahaEmily BryanPushpa Raj JoshiEmily C. RadlowskiPushpendra KumarEmily CaponePuspa Raj PantEmily CoxPuspendra P. SinghEmily E. GrammerPuthearath ChanEmily EmmottPuting DongEmily HaasPuya Dehgani MobarakiEmily J. SauersPyziak-Skupień AleksandraEmily KroskaQaisar MahmoodEmily LauerQaiser RiazEmily LowthianQamaruddin MaitloEmily MaileyQi CaiEmily PadhiQi FanEmily ReadQi FangEmily RobertsQi HuEmily S. BaileyQi JinEmily ScheinfeldQi WangEmily ShoesmithQi YanEmily W. DuffyQi ZhangEmily ZimmermanQian DiEmin BayraktarQian Forrest ZhangEmina HerovicQian WangEmir GanićQian WuEmir OzerenQian YeEmma Adriana OzonQian ZhouEmma BorrelliQian ZhuEmma C. CacciolaQiang ChenEmma CarlinQiang FuEmma CowleyQiang GaoEmma HakalaQiang LiEmma HockQiang LiuEmma Kurnat-ThomaQiang WangEmma L. GillinghamQiang ZhuEmma LidingtonQianling JiangEmma MarczyloQianyi FuEmma MaynardQiao LiangEmma MontellaQiao ZhuEmma MortonQifa ZhouEmma MosleyQifan NieEmma PietrafesaQihang QiuEmmanouela SdonaQihong ZhuEmmanouil KapetanakisQihui ChenEmmanouil KokkinakisQiliang DengEmmanouil MagiorkinisQimin HaiEmmanouil PikoulisQi-Ming HeEmmanouil RizosQin SuEmmanouil ZouliasQin Xiang NgEmmanuel AboagyeQinaat HussainEmmanuel BerthierQing SuEmmanuel D. AdamidesQing SunEmmanuel FavaloroQing YeEmmanuel Fortunato GulinoQing ZhangEmmanuel FrimpongQingchuan LiEmmanuel Karlo NyarkoQingchun GuoEmmanuel MavhuraQinghua NianEmmanuel MulinQingjun PanEmmanuel N. KontomanolisQinglan DingEmmanuel Navarro-FloresQingli DongEmmanuel NjogaQingmu SuEmmanuel Obeng-GyasiQingsong HeEmmanuel RineauQingyang WeiEmmanuel SalimQingyu ZhaoEmmanuel Senyo FianuQinjian JinEmmanuel SerranoQinli ZhangEmmanuel ZúñigaQinyun LinEmmanuele A. JanniniQiong WangEmmanuella MagriplisQiongxian YanEmmanuelle AmorosQiqi ChenEmmanuelle CugyQitao TanEmoke PallQiting ZuoEmőke-Szidónia FederQiu LimingEmre AksoyQiuju GuoEmre DemirkayaQiuliyang YuEmyr BenbowQiuping LiEmyr Reisha IsauraQiusheng YuanEnas A. Abdel-HadyQiyang LiuEncarnación Martínez-GarcíaQize ZhangEndale TadesseQoot AlkhubaiziEnder KazazogluQu ChengEndre ZimaQuamrul HudaEndrin KoniQuan ZhouEndrit ShahiniQuang Trung NguyenEnéas A. Fontes-JúniorQuan-Hoang VuongEnedina SánchezQuanjun LyuEng Tat AngQuanle ZouEng. Haim CikurelQuanpeng YangEngin BaysenQuanyi ZhaoEngin YaralıQuanyin TanEng-kiong YeohQuenette L. WaltonEngy ElekhnawyQuin MorrowEnid J. SchatzQun WangEnita NakasQuy Van KhucEnkeleint A. MechiliR. Constance WienerEnno NowossadeckR. David HaywardEnno Van der VeldeR. Henry OlaisenEnoch A. MensahRa’ed Masa’dehEnoch AninagyeiRaad Z. HomodEnoch Odame AntoRabiu Muazu MusaEnoch ParkRabiu MusaEnquan XuRachael BaiducEnri NakayamaRachael L. MosesEnrica CiucciRacheet MataiEnrica De FalcoRachel CriswellEnrico BentivegnaRachel DaleEnrico BergamaschiRachel E. DavisEnrico BocciolesiRachel E. SteinEnrico BrugneraRachel GoldsteinEnrico BrunettiRachel HopmanEnrico ContiRachel J. SkowEnrico DestefanisRachel JenkinsEnrico FerreroRachel KafriEnrico G. CaianiRachel L. WashburnEnrico IvaldiRachel LoganEnrico MancinelliRachel M. RanneyEnrico MorettoRachel Mamlok-NaamanEnrico OddoneRachel MilesEnrico SellaRachel Nissanholtz-GannotEnrico SicignanoRachel R. OuelletteEnrico StaderiniRachel SharabyEnrique ArranzRachel SoperEnrique ArribasRachel TamblingEnrique AubáRachel TanEnrique ContrerasRachel TaylorEnrique García OrdóñezRachel YerburyEnrique Gea-IzquierdoRachele MarianiEnrique GermanyRachid Ait AddiEnrique MattosRachid MarzougEnrique Méndez BolainaRachid OuaretEnrique MontonRachida DalouhEnrique Morales-VillegasRachna KapoorEnrique Ramón ArbuésRachna ValvaniEnrique Rico-GarcíaRaci KarayigitEnrique Romero-VelardeRada KazakovaEnrique S. PumarRadenko MaticEnrique Sáez-AlvarezRadhi Al-MabukEnrique Urrea-MendozaRadhika De SilvaEnsar AbazovicRadhya SahalEnshirah Da’naRadìm ŠrǎmEnwu LiuRadim TolaszEnza SperanzaRadivoj ProdanovićEnzo Maria VingoloRadka PrichystalovaEphraim Kwashie ThompsonRadmila SparicEphrem EngidaworkRadoje VujadinovićEraldo OcchettaRadoslav BeňušErdem GökerRadoslaw B. WalczakErdogan MalatyaliRadosław ChaberEren OnerRadosław GacaEren ŞahinerRadosław GocołEren YildizRadosław IdzikowskiErfu DaiRadosław KempińskiErhan OkuyanRadosław MącikEri MatsubaraRadosław MaksymEric A. EppingRadoslaw Marek KiedrowiczEric BerksonRadosław MirskiEric ConcholaRadosław RybarskiEric Danso-BoatengRadosław ZajdelEric DukuRadostina A. AngelovaEric ForcaelRadovan ŠomplákEric Francelino AndradeRadu Bogdan TomaEric GatoRadu BotezatuÉric GouletRadu BotgrosEric HodgsonRadu C. RacovitaEric J. CooksRadu ChiceaEric Kam-pui LeeRadu CiorapEric KloppRadu D. StanciuEric KyereRadu LupuEric Larry WisotzkyRadu NeamtuEric LutzRadu Paul MihailEric M. LambergRadu PrejbeanuEric MartinRadu SageataEric MosierRadu Silaghi-DumitrescuEric OlsonRadu-Ioan PopaEric OpokuRadzisław MierzyńskiEric R. BravermanRae Yule KimEric RobinsonRaeann G. LeBlancEric RobitailleRaees CalafatoEric RytkinRafael A. CasusoEric SchusslerRafael AcevedoEric SilvaRafael Almendra-PeguerosEric SobolewskiRafael BernardesEric StokanRafael Brandao VarellaEric Van GorpRafael BurgueñoEric VukicevichRafael Castro DelgadoEric W. PetersonRafael Del Rio-SalasEric YoshidaRafael Escamilla-NunezErica CostantiniRafael García-QuesadaErica CruvinelRafael Giovani MisseErica GobbiRafael GómezErica L. EliasonRafael Gómez-GalánErica L. MajumderRafael Gutiérrez-LópezErica LauRafael HarunErica MenegattiRafael KonsErica NeriRafael LlobetErica Timko OlsonRafael López-CorderoErica TongRafael ManzaneraErica VareseRafael Martinez-GallegoErica VoltaRafael Moreno-Gómez-ToledanoErich KastenRafael OliveiraErich SorantinRafael Ortí-LucasErick Andres Perez AldayRafael Ramiro De AssisErick Martinez-HerreraRafael Rávina-RipollErick Sierra-CamposRafael Salido-VallejoErico RenteriaRafael ToledoErik B. LarsonRafael Vila-CandelErik BerglundRafaela AndolheErik De SoirRafaela Gutierrez CaceresErik KarlssonRafaela Maito Velasco SofiaErik PerssonRafaela RosárioErik R. SwensonRafaela VasiliadouErik SauleauRafaella Pessoa MoreiraErika Estrada CamarenaRafal BalinaErika FaberRafał Białynicki-BirulaErika JoosRafał BlazyErika MarekRafał ChmaraErika OrbanRafał FrańskiErika QuendlerRafał GerymskiErika ScottRafał J. DoniecErika T. EbbsRafał K. WyczółkowskiErika WolfRafal KaminskiErika ZemkováRafał MichalskiErin A. VogelRafał MłyńskiErin BrennandRafał ObuchowiczErin ByrdRafał PrusakErin ClarkeRafał RójErin CrawRafał Stanisław JureckiErin De Fries BouldinRafał StaszkiewiczErin E. SullivanRafał StemplewskiErin HobinRafał SzafraniecErin KellyRafal WolnyErin KeltyRafat GhanamahErin Lavender-StottRafea Al SuhiliErin S. RogersRaffaele BaioErjia GeRaffaele CioffiErlandson Ferreira SaraivaRaffaele La RussaErman ÇakıtRaffaele MarroneErna KaralijaRaffaele MauroErnest TyburskiRaffaele PerniceErnesto A. Lagarda-LeyvaRaffaele SerraErnesto RosarioRaffaella CalatiErnestyna Szpakowska-LorancRaffaella CoccoErnoiz AntriyandartiRaffaella ZerbettoErnst OberlechnerRaffaello PellegrinoErnst StadloberRafi YoungmannErol YildirimRaghav KaliaEros Di GiorgioRaghu GandhiErsan ArslanRaghunadharao DigumartiErsilia LucenteforteRagna StalsbergErtuğrul ŞahinRahbel RahmanErum RehmanRaheema Abdul RaheemEryk KosinskiRahim AbdurEsau JoaoRahimi A. RahmanEshani HettiarachchiRahman TafahomiEshwar RavishankarRahul ChaudhariEslam Mohammed AbdelkaderRahul JainEsma ÖzkanRahul MukherjeeEsmaeel SoleimaniRahul SehgalEsmaeil MehraeenRaid Al-TahirEsmail DoustkhahRaid NajiEsme HacıeminoğluRaif CergibozanEsmé Jansen Van VurenRail M. ShamionovEsmeralda DelgadoRail ShamionovEsmeralda Santacruz-SalasRaimundo Jimenez-BallestaEspen OlsenRaimundo MateosEsperanza Begoña García NavarroRaimundo Otero-EnriquezEsperanza Clares-ClaresRain LiuEsposito LucaRainer LeonhartEsra AǧelRaj K. GcEsra KulRaj KumarEssam Al-MoraissiRaj RengalakshmiEssam SolimanRaja DandamudiEsteban Agulló-TomásRaja RehmanEsteban Obrero-GaitánRaja VeerapandianEsteban Ortiz-PradoRajaguru VasukiEsteban Sánchez MorenoRajai JureidiniEstefanía Acién GonzálezRajalekshmi C. PillaiEstefanía Bautista-ValarezoRajamohanan K. PillaiEstela BlancoRajan JakhuEstela CalatayudRajani RaiEstela Cuevas-RomeroRajaretnam Kingsy GraceEstela Marine-RoigRajarshi DasguptaEstela Ruiz-BacaRajat Emanuel SinghEstela SalagreRajeev DwivediEster AyllónRajeev K. SinglaEster Garcia-CelaRajendra KurapatiEster NavarroRajendra NathEster OlmedaRajesh AngireddyEster TriguerosRajesh GuptaEster Vidaña-VilaRajesh KasamEstera Twardowska-StaszekRajesh Kumar DuttaEsther Christiane RindRajesh Kumar SinghalEsther CuadradoRajesh N. V. P. S. KandalaEsther Garcia-RioRajesh P. ShastryEsther K. ElliotRajesh PandeyEsther LázaroRajib Lochan DasEsther LubzensRajib ShawEsther MaasRajiv JanardhananEsther Medrano-SánchezRajiv PeriakaruppanEsther Moraleda SepúlvedaRajiv Ranjan SrivastavaEsther O. ErdeiRajnish S. DaveEsther SanfeliuRajveer SinghEsther VillajosRakan Al YamaniEsther WillingRakesh ChaudhariEsther YoudRakesh NagarajuEstibaliz JarautaRakhi IssraniEstrella Armada-CortésRaktim RoyEstrella RausellRalf AdamEstrella TrincadoRalf BuckleyEszter Eniko MarschalkoRalf ErberEszter SzabadosRalf SchwarzerEthan WalkerRalf ZieglerEtsuro ItoRalph BaergenEu Gene ChinRalph FingerhutEuclides Lourenço ChumaRalph L. PiedmontEugen AvramRalph LuthardtEugene AndersonRalph MeulenbroeksEugene SilowRalph SonenshineEugenia GallardoRaluca Ioana Horea-ȘerbanEugenia M. W. NgRaluca IsacEugenia PapadakiRaluca LadaruEugenia VlachouRaluca MijaicaEugenio CavalloRaluca RăcăşanEugenio De GregorioRaluca SassuEugenio OropalloRam C. SharmaEugenio RagazziRam K. MazumderEugenio Velasco-OrtegaRam Kinker MishraEugeniusz KodaRam Krishna UpadhyayEugeniusz MoczukRam Prasad AcharyaEugeniusz MokrzyckiRam SharmaEugeniusz ProninRam Vinay PandeyEui Seob JeongRama PulicharlaEulalia Arias-PujolRamaa BalkaranEulalia SkawińskaRamachandran PrakasamEun A. L. KimRamachandran SrinivasanEun Hye KwonRamakrishnan RamanEun Jeong HwangRamalingam GopalEun Jeong ParkRamazan YirciEun Joo ParkRambabu PothinaEun Nam LeeRamcés Falfán-ValenciaEun Woo NamRamendra Pati PandeyEun Young ParkRamesh ChandrababuEuna HanRamesh KumarEunchan MunRamesh Kumar GargEung Ju KimRamesh NagarajappaEun-Hi ChoiRamesh S. YadavaEun-Hi KongRameswari ChilamakuriEunhoe GooRamey MooreEunice CarrilhoRamganesh SelvarajanEunice MacedoRami DiabEunjeong ChaRamjee GhimireEun-Jeong KimRamokone LebeloEunjoo YangRamokone Lisbeth LebeloEunjung HyunRamón Bouzas-LorenzoEun-jung LeeRamón Castañer BotellaEunkyu LeeRamon Cladellas ProsEunsil ParkRamon FlechaEunyoung HanRamón FuentesEun-Young ParkRamón García-PeralesEusebio ChiefariRamon GonzalezEustaquio AbayRamón Llopis-GoigEva Aguaded-RamírezRamona BabosovaEva AusóRamona BongelliEva Ausó MonrealRamona BranÉva Bácsné-BábaRamona HenterEva BagyinszkyRamona Işfănescu-IvanÉva BíróRamona KuhnEva Češ̈kováRamona McNealEva CunhaRamona Năstuță HuzumEva GerslovaRamona Silvana De AmicisEva HajnalRamona SuharoschiEva HasslerRamona ZgavarogeaEva HenjeRamune JacobsenEva HolsingerRamune ZurauskieneÉva KállayRamya BillurÉva KeresztyRamya ShenoyEva L. BergstenRan AnEva León ZarceñoRana K. ZabadEva LitavcováRana Muhammad Adeel-FarooqÉva LovraRana Muhammad AdnanEva María Martínez-JiménezRanda El BedawyEva Maria Navarrete-MunozRanda EshaqEva Mihalyko-OrbanRandall MaplesEva PetizRandhir KumarEva PodovšovnikRandi K. JohnsonEva Raudonytė-SvirbutavičienėRandi VitaEva RothermundRanganath VallabhajosyulaEva Sánchez-HernándezRania ChristoforouEva Segura-OrtíRania M. GhoniemEva SemančíkováRania MekaryEva SventekovaRania W. SemaanEva T. López SanjuánRanieri PoliEva TékusRanjeev Chrysanth PulleEva Torres-SangiaoRanjit Kumar PaulEva TsaousidouRanjith KamityEva TvrdáRanka GodecEvaldo FaviRanko BiondićEva-Maria RisoRannakoe J. LehloenyaEvan A. NadhimRao BalusuEvan AntzoulatosRaouf Senhadji-NavarroEvan D. PeetRaoul NuijtenEvan J. DouglasRaphael Martins De AbreuEvan RossRaphael PetegrossoEvan SenreichRaquel Agnelli Mesquita FerrariEvando AraújoRaquel Aparicio UgarrizaEvandro Eduardo BrodayRaquel Braz Assunção BotelhoEvandro WatanabeRaquel CanteroEvangelia AntoniouRaquel CostaEvangelia ChrysikouRaquel EchavarriaEvangelia GoliaRaquel Fábrega-CuadrosEvangelia KerezoudiRaquel Faria de DeusEvangelia KouidiRaquel FaubelEvangelia KoukakiRaquel Fernández GarcíaEvangelia NenaRaquel FreitasEvangelia TzanetouRaquel García RevillaEvangelos I. KritsotakisRaquel Gilar-CorbiEvangelos K. KaimakamisRaquel Gomez LopezEvans AsensoRaquel HueteEvanthia AsimakopoulouRaquel Lama LópezEvanthia SakellariRaquel Lara MorenoEvaristo Barrera AlgarínRaquel Lazaro GutierrezEvdokia KaravasRaquel Leirós-RodríguezEve BohnettRaquel Lozano BlascoEve GriffinRaquel Ortega LapiedraEve NamisangoRaquel RamosEve S. PufferRaquel Rodríguez-BlanqueEvelia Franco ÁlvarezRaquel Sanabria-De-La-TorreEveline J. M. WoutersRaquel Sánchez-RecioEvelio Teijón-López-ZuazoRaquel Sánchez-RodríguezEvelyn LeeRaquel SilvaEvelyn McElhinneyRaquel Simões De AlmeidaEvens EmmanuelRaquel Tardin-CoelhoEverton Falcão De OliveiraRaquel Vaquero-CristobalEvgenia GkintoniRaquel VázquezEvgenia OstroumovaRares Halbac-Cotoara-ZamfirEvgenii GusevRareş Mircea BîrluţiuEvgenii KuzinRasa BarkauskienėEvgeniy CherepovRasa BartkuteEvgeniy MakarchenkoRasa JankauskieneEvgeny D. KasyanovRasa MladenovicEvgeny R. BojkoRasa Pilkauskaitė ValickienėEvita StraumiteRasa PranskūnienėEvlyn NovoRasa SmaliukienėEvonne MillerRasa TamelienėEvridiki KabaRashad AlfkeyEwa BielskaRashed NoorEwa BrzdękRashedul HasanEwa ChlebuśRashelle HoffmanEwa ChodakowskaRashid AmeerEwa Chomać-PierzeckaRashid LatiefEwa CichowiczRashmi JoglekarEwa Czarniecka-SkubinaRashmi YadavEwa DacewiczRasmus KleppeEwa DudaRatana SapbamrerEwa FalkowskaRathinamoorthy RamasamyEwa FlorekRatmawati MalakaEwa GajewskaRatnakar DeoleEwa Gibuła-TarłowskaRattana JariyaboonEwa GieysztorRaul Alberto Carrilho CordeiroEwa GniazdowskaRaul Alegria-MoranEwa GrudzińskaRaul AntunesEwa HąciaRaúl Arango-MirandaEwa HalickaRaúl BañosEwa HumeniukRaúl Capote-PuenteEwa JaroszRaúl Cuauhtémoc Baptista RosasEwa JaskaRaúl Díaz-MolinaEwa Joanna RodakowskaRaul Domínguez HerreraEwa KnapikRaúl EspertEwa KocotRaul Felipe Palma-AlvarezEwa KołoszyczRaúl Ferrer PeñaEwa KorzeniewskaRaúl Frutos LlanesEwa KruszyńskaRaul Josue Nájera-LongoriaEwa KusidełRaul Lopez-GruesoEwa LangeRaúl López-IzquierdoEwa Miller-KasprzakRaul MatsushitaEwa MisterskaRaul NavarroEwa NiewiadomskaRaul OrtegaEwa NowakRaúl Pérez CaballeroEwa OkoniewskaRaúl PezoaEwa Olchowik-GrabarekRaúl Quevedo-BlascoEwa Otto-BuczkowskaRaúl Soto-CámaraEwa PolakRaul VilarinhoEwa Puszczalowska-LizisRauno PääkkönenEwa RaczkowskaRavesh SinghEwa RopelewskaRavi GudavalliEwa RzońcaRavi Kumar CheedaralaEwa SewerynRavi Philip RajkumarEwa SicinskaRavi Shankar SathyamurthyEwa SkarżyńskaRavi YadavEwa ŚliwickaRavinder KumarEwa SobolewskaRavindra A. PrabhuEwa StachowskaRavindra Kumar JenaEwa SzusterRaviteja VangaraEwa TomaszewskaRawan AlHereshEwa WedrowskaRawiwan SiriratEwa Wender-OzegowskaRay CorrellEwa WieloszRay MerrillEwa WilkRay NorburyEwa Wszendybył-SkulskaRay W. BasrowiEwa Zabłocka-GodlewskaRay W. SquiresEwa ZasadzkaRay ZhongEwan McDonaldRaymond CespuglioEwan ThomasRaymond D. AdamsEwan WilkinsonRaymond M. QuockEwelina ChawłowskaRaymundo Buenrostro-MariscalEwelina CzubackaRayonil CarneiroEwelina DziurkowskaRaysa RochaEwelina GaszyńskaRayssa Ferreira ZanattaEwelina GrywalskaRazia AdamEwelina HallmannRaziye PourdarbaniEwelina KrólRăzvan Adrian IonescuEwelina MaculewiczRazvan BocuEwelina NojszewskaRazvan DinaEwelina PogorzelskaRǎzvan PetcaEwen C. D. ToddRazvan SerbuEzio TodiniRazvan SocolovEzzaldeen EdwanRăzvan-Ionuț PopescuEzzat KhanRebbecca LilleyFabián CasasRebeca Boltes CecattoFabian FischerRebeca Gómez-IbáñezFabian KerwagenRebeca María Elena Guzmán-SaldañaFabian Martin TroschelRebeca Monroy TorresFabian TomschiRebeca RamisFabiana BolelaRebeca Robles-GarcíaFabiana Di DucaRebeca Suárez-ÁlvarezFabiana Gil MelgaçoRebecca A. G. ChristensenFabiana NicitaRebecca A. StatesFabiana TezzaRebecca AlleeFabiano Azevedo DorçaRebecca AmatiFabien A. BassetRebecca AppletonFabio A. MadauRebecca BabcockFabio AngeolettoRebecca ErschensFabio BarboneRebecca EvansFabio Batista MotaRebecca FrederickFábio BittencourtRebecca H. ChungFabio BorghettiRebecca HubbardFabio CarboneRebecca J. WilliamsFabio CianferoniRebecca J. WrightFabio Del DucaRebecca McClayFabio EboliRebecca MeadFábio FernandesRebecca NantandaFábio Juner LanferdiniRebecca PrattFabio PinheiroRebecca RaesideFabio PisaniRebecca RosenFabio SantacaterinaRebecca SargissonFabio SasaharaRebecca TsaiFabio ScoppaRebecca TsuiFabio SereniRebecca UpsherFabio SerpilliRebecca WongFabio TimeusRebecka LundgrenFabio ZainaRebekah L. WilsonFabio ZickerRebekka LeeFabiola BizziRebekka MummFabiola Miranda-SanchezRebekka WeidmannFabiola SpolaorRebekkah MiddletonFabrício Daniel Dos Santos SilvaReda ElkacmiFabricio Eduardo RossiRedha TaiarFabrício PereiraRedi RahmaniFabrizia D’ApuzzoRedmond R. ShamshiriFabrizia FestanteReem AlkilanyFabrizio BattistiReeta KankaanpääFabrizio BraccoReeta LamminpääFabrizio CedroneRegiane Maria Tironi De MenezesFabrizio ChiricoRegina C. CasperFabrizio ConsortiRegina C. SerpaFabrizio DesideriRegina Ferreira AlvesFabrizio FasanoRegina LeiteFabrizio OlivitoRegina Maura De MirandaFabrizio SantoboniRegina N. ParnellFabrizio Terenzio GizziRegina Pana-CryanFady MansourRegina WierzejskaFadzidah AbdullahReginald AlvaFahad AlasmariReginaldo FidelisFahad AsmiRegragui TakiFahad Bin AbdullahRégulo López-CallejasFahad HannaReham ShalabyFahad M. AldakheelReidar J. MykletunFahad QuhalReiko HojoFaham KhamesipourReima SuomiFahd QadirReinhard DallingerFaheem Ahmed MalikReinhard StrametzFahiel CasillasReinier TimmanFahimeh GolbabaeiRejina ManandharFahimeh RanjbarRekha BalakrishnanFahriye AltınayRekha WaganiFahriye Hilal HaliciogluRemco S. MannakFáima GameiroRemedios Lopez-LiriaFaisal AlghayadhRemedios MeleroFaisal AzizRemigijus BubnysFaisal F. HakeemRemigijus LeipusFaisal K. ShaikhRemigiusz BąchorFaiz Ullah KhanRemigiusz GałęckiFaizah Safina BakrinRemington L. NevinFakhradin GhasemiRemo MombargFakhredin SabaRemus CretanFakhri JeddiRemy HurdielFakhri ShahidiRemy NicolasFakir M. Al GharaibehRena KostiFaliang LuoRenan Dal-FabbroFaming WangRenata BalnytėFan WuRenata Barczyńska-FelusiakFan YangRenata ChalasFan ZhangRenata De Castro MartinsFang TongRenata Eloahde Lucena Ferretti-RebustiniFang WangRenata GasparinoFang YangRenata Gruca-RokoszFang ZhengRenata GržićFangcong DongRenata GrzywaczFangfang LiuRenata Jadresin MilicFangqing WeiRenata KędziorFangwu WeiRenáta KopenaFangxin YiRenata KorsakienėFangyi LiuRenáta MachováFang-Ying ChiuRenata Pacholczak-MadejFangzhu ZhaoRenata PerlikowskaFanhao NieRenata Pietrzak-FiećkoFani GkrozouRenata PiotrkowskaFani LadomenouRenata Puppin ZandonadiFanlei KongRenata RetkuteFanny BoulaireRenata Różycka-CzasFanyu HuangRenata RutkauskaiteFan-Yun PaiRenata SiemienskaFaozi A. AlmaqtariRenata SoliminiFarah AmmousRenata SoutoFarah AzizRenata Tobiasz-SalachFarah HamandiRenata Toczylowska-MaminskaFarah IslamRenata Voltolini VelhoFarah QureshiRenata Walczak-JędrzejowskaFarahnaz AminiRenata Wolfshaut-WolakFarai NyabadzaRenata WoźniackaFaraz FarhidiRenata ŻyłłaFareeda Abo-RassRenate KlaassenFares Abu-AbedRenate WeissFarhad GaravandRenato CostaFariba GoodarzianRenato De Oliveira MoraesFariba MostajeranRenato Janine RibeiroFarid MenaaRenato Sobral Monteiro-JuniorFarid OrudzhevRencai DongFarid Saberi-MovahedRene A. de WijkFarida BegumRene CorteseFarida NurhasanahRené HandschuFarida SaleemRene RietraFarina KingRené Ulloa-EspíndolaFaris F. BrkicRenee RobinsonFaris KhanRenée S. MacPheeFarman Ali KhanRen-Fang ChaoFarnaz Ghazi-NezamiRenfeng MaFarnoosh Abbas-AghababazadehRengin GüzelFarooq AhmedRenin TomsFarrukee RubaiyeaRen-In YouFarrukh ChaudhryRenisson Neponuceno Araújo FilhoFarrukh ChishtieRen-Jay SheiFarshad SohbatzadehRenjith V. RaviFarshid MirzaalianRennan Yanlin DuFarzad AmirabdollahianRens MeerhoffFarzad SalehpourRenuka JayatissaFarzad Taghizadeh-HesaryRenuka RamanFarzaneh MoayediRenzo ZanottiFatema ShaikhReparata Rosa Di PrinzioFatemah AlsalehResi PucciFatemeh DabbaghReuben Olugbenga AyelekeFatemeh Mohammadi-NasrabadiReuben TamakloeFatemeh Sanie-JahromiReuven Maskil-LeitanFatemeh YousefianRevan Birke Koca-ÜnsalFathima WakeelRey David Vargas-SánchezFatih DurmuşoǧluReyna María Guadalupe Fonseca-Montes De OcaFatih KarayürekReza AfhamiFatih ÖzReza ArezoomandanFatih OzbugdayReza BaratiFátima AmaroReza DerakhshaniFátima Chacón-BorregoReza KafipourFátima FradeReza MazaheriFátima García-VillénReza PiriFatima Lambarraa-LehnhardtReza RanjbarFatima Leon LariosReza ZamaniFatima MucceeRhett MartinFatima O. MartinsRhiannon V. McNeillFatima RizviRhianon ReynoldsFátima RoqueRhona WinningtonFatimah Saeed AlhamlanRhonda GaradFatma Nese SahinRhonda GriffithsFatma Turna DemirRhonda L. ShermanFatma YıldırımRiaan MulderFatme AlAnoutiRicarda MicallefFatmir DragidellaRicardo Aguasca-ColomoFau RosatiRicardo AlmendraFausta LuiRicardo AraujoFaustin Armel Etindele SossoRicardo Bernardez-VilaboaFausto AssandriRicardo Castro AlvesFausto ZampariniRicardo Chalmeta RosaleñFauzia JabeenRicardo Costa ClimentFayaz Rahman KhanRicardo Dinis-OliveiraFaye McCallumRicardo Escobar-JiménezFayuan WangRicardo FernandesFederica AltieriRicardo GomesFederica AmatiRicardo Hernández-RojasFederica BevilacquaRicardo IbañezFederica BuccinoRicardo Iglesias-PascualFederica CanzanRicardo LoureiroFederica CappelliRicardo MalheiroFederica CavazzoniRicardo Moreno-RodriguezFederica ColliniRicardo Pérez-LucoFederica D’AlòRicardo RojasFederica DemariaRicardo Sánchez De MadariagaFederica Di SpiritoRicardo Soares-Dos-ReisFederica EpifaniRicardo TesorieroFederica FilippiRicardo VardascaFederica FogacciRicardo Ventura BaútoFederica GalimbertiRicardo Vivas-ReyesFederica LaguzziRiccardo AigottiFederica MagagnoliRiccardo AiutoFederica MurmuraRiccardo Antonio RicciutiFederica PolverariRiccardo BeltramiFederica RomanoRiccardo BientinesiFederica ValerianiRiccardo CorradoFederica VeroneseRiccardo IzzoFederico A. León-ZerpaRiccardo MastroianniFederico Abate DagaRiccardo Matteo CioniFederico ChmetRiccardo OrusaFederico D’AntoniRiccardo PistelliFederico FerrelliRiccardo TartagliaFederico FloreaniRiccardo ZuccaFederico GerminiRich GormanFederico M. StefaniniRichard A. BowenFederico Maria RubinoRichard B. KruegerFederico MastroleoRichard B. StuartFederico MiglioreRichard ButlerFederico MorettiRichard CharlewoodFederico PapaRichard ChristianaFederico PennestrìRichard D. FoustFederico RicciRichard DarveauFederico RoggioRichard E HeymanFederico ZainaRichard E. FryeFederico ZannoniRichard EbsteinFedor TatarinovRichard F. IttenbachFedor VybornovRichard F. PotthoffFei ChenRichard F. SchmidFei LuoRichard FedorkoFei MenRichard GravesFei PeiRichard H. MartinFei PengRichard H. RobertsFei WangRichard IsralowitzFei XuRichard J. CebulaFei ZhangRichard J. StarkFeixiang ZanRichard John Bruce FrancisFeiyan ChenRichard JohnsonFeiyu ChenRichard K. FlemingFelice LorussoRichard KahnFelice SiricoRichard KdolskyFelice SorrentinoRichard L. WarmsFelicia ConstantinRichard L. WolfelFelícia FonsecaRichard M. RyanFelicia Ramona BirauRichard McLeanFeliciano H. VeigaRichard MolesFelipa De Mello SampayoRichard MorehouseFelipe B. G. SilvaRichard N. MerchantFelipe Contreras-BriceñoRichard Neil GreeneFelipe CostaRichard ParrishFelipe Da CunhaRichard PaulFelipe J. AidarRichard PearsonFelipe León MorillasRichard PeterFelipe Muñoz La RiveraRichard PriceFelipe Ojeda PérezRichard R. SuminskiFelipe RidolfiRichard ScepuraFelisa LatorreRichard ScottFélix A. UrraRichard SegallFelix Ballesteros LeivaRichard SmardonFelix CarrielloRichard ViskupFelix Haifeng LiaoRichard W. BohannonFelix Javier Jiménez JiménezRichard W. GriffinFelix Jimenez-RondanRichard W. StoffleFelix M. OnyijeRichard WallsgroveFélix Marcos TejedorRichard WrightFelix ReschkeRichards SunnyFelix SchmittRick LaguerreFelix SternbergRickelle RichardsFelix StrolloRicky Finzi-DotanFelix ZhouRidha GhayoulaFeng JiangRie MiyazakiFeng LiRifat AkhterFeng RenRifat Ullah KhanFeng WangRiham FliefelFeng XuRiitta SauniFeng YuanRiitta TurjamaaFengchao LangRijuta GaneshFeng-Chyi DuhRika Indri AstutiFenghua SunRike BeckerFeng-Hua YangRiku KlénFeng-Jen TsaiRiku NikanderFenglin ZhangRim BourgiFengping LiRima E. RuddFengqing ChaoRima Jamal IsaifanFengshu LiRima KregždytėFengzhi LiRimadani PratiwiFerdinand GaroffRimma KalinaFerdinando AttanasioRina DasFerdinando Di MartinoRina Suryani OktariFerdinando FebbraioRini RachmawatiFerdows AfghahRinki KumarFerenc BognárRipu KunwarFerenc IhaszRiquelme Marín AntonioFerenc JankóRisako MiuraFerenc MeszarosRishabh ChoudharyFerenc OroszRishabh U. ShahFerhat KaracaRishi Man ChughFerman KonukmanRishika SakariaFermín VillarroyaRishikesh BajagainFern WebbRishil KathawalaFernanda Guerra Lima Medeiros BorsagliRisto H. MarttinenFernanda Isabel Oduber PérezRita BaraldiFernanda SperottoRita BergerFernanda TonelliRita BritoFernando A. WilsonRita CannasFernando AlacidRita Cássia Coelho De Almeida AkutsuFernando AntoñanzasRita Catalina Aquino CaregnatoFernando Austria-CorralesRita ChiaramonteFernando BarragánRita De Cássia AquinoFernando BoinasRita De Cássia Pontello RampazzoFernando CardonaRita KabraFernando CarvalhoRita KissFernando Cepero-MejíasRita ManoFernando De C. JacinaviciusRita Maria Vittoria NobiliFernando De MoraRita OliveiraFernando Díez RuizRita PadoanFernando FerreiraRita PavasiniFernando FerreroRita PeresFernando FonsecaRita PolitoFernando Gimeno-AriasRita RonconeFernando GõmezRita S. StrakovskyFernando Gonzalez-AlonsoRita Santos-RochaFernando González-MohínoRita SeeboeckFernando J. C. Magalhães FilhoRita SousaFernando J. PereiraRita Tavares De SousaFernando Javier García-CastroRita VolochayevFernando José HerkrathRita Yi Man LiFernando L. MeloRita ZarboFernando Lanas ZanettiRitesh ChimoriyaFernando Leiva-CepasRitesh KumarFernando Lopitz-OtsoaRitesh MenezesFernando M. S. F. MarquesRito BaringFernando Manuel Lourenço MartinsRitthideach YorsaengFernando Martín-RiveraRittika ShamsuddinFernando MendesRitu ChauhanFernando MonroyRizauddin Bin RamliFernando MoreiraRizwan JouharFernando Muñoz-HernándezRizwan QureshiFernando Navarro MateuRizwan ShahidFernando OlivaresRizwan WahabFernando Pérez-RodríguezRob A. GrutersFernando Poyatos-JiménezRob DonovanFernando Ribas FeijóRob FarrowFernando RibeiroRob Kim MarjerisonFernando RiegelRob KinnersleyFernando RochaRob SiebersFernando Rodríguez RodríguezRobbert HuijsmanFernando Rodríguez-ArtalejoRobert A. HorowitzFernando Rubén García HernándezRobert Alan SloanFernando SantosRobert AlexanderFernando Suparregui DiasRobert BembenikFernando Toledo FerrazRobert BłaszczykFernando ZaroneRobert BosFerreira-Gonzalez IgnacioRobert BoydFerruccio ConteRobert BrackbillFerucio Laurentiu TipleaRobert BraggFesto KayimaRobert BransfieldFestus Victor BekunRobert Brawura-Biskupski-SamahaFevzi YılmazRobert C. GiblerFiammetta CosciRobert CarleerFidel Hita-ContrerasRobert ĆelićFidelis AjibadeRobert Cristian PurdoiuFides A. del CastilloRobert D. BixlerFigen BaloRobert D. MorrisFigueroa Montaño ArturoRobert DempseyFikret UstaoğluRobert DonovanFilbert TotsinganRobert E. PittsFiliberto Toledano-ToledanoRobert E. PykeFilip JovanovicRobert EgermanFilip KukićRobert Emmett KellyFilip MilanovicRobert F. BentleyFilip RaciborskiRobert F. HerrickFilipa CardosoRobert FlisiakFilipa M. RodriguesRobert GajdaFilipa MandimRobert GirlingFilipa Maria Santos FerreiraRobert GniadeckiFilipa SeabraRobert GrozaFilipa VenturaRobert GuthrieFilipa VicenteRobert HoerrFilipe FidalgoRobert HomeFilipe Manuel ClementeRobert J. MoriarityFilipe Paiva-SantosRobert K. StallmanFilipe PrazeresRobert KissFilipe RodriguesRobert Konrad SzymczakFilippe Falcao-TebasRobert KowalikFilippo AntoniacciRobert KrzysztofikFilippo BanchiniRobert KunstFilippo BarberaRobert L. MeiselFilippo ConfalonieriRobert M. BadeauFilippo Della RoccaRobert M. CariniFilippo DiaraRobert M. NowakFilippo FratiniRobert M. SiegelFilippo MacalusoRobert MachowskiFilippo ManelliRobert McMasterFilippo MantiRobert N. AndersonFilippo Maria NimbiRobert N. MontgomeryFilippo MarianoRobert NewellFilippo MaselliRobert NowackiFilippo PaganelliRobert OhFilippo PieriniRobert OlszewskiFilippo RapisardaRobert OporaFilippo RebessiRobert PawlusińskiFiliz Bektas BalcikRobert Philipp KosilekFilomena FezzaRobert PopekFilomena MaurielloRobert PriceFilomena MazzeoRobert PrillFilzah Md IsaRobert RijavecFiniki NearchouRobert RollinsFinn DiderichsenRobert S. OlickFinn Edler Von EybenRobert Steven EsworthyFiona Caulfield-EcarnotRobert StrongFiona EcarnotRobert SusłoFiona FrenchRóbert SzilágyiFiona Joy NewtonRobert SzmytkieFiona K. P. SiuRobert TaylorFiona MajorinRobert TomanekFiona OatesRobert TragerFiona SaundersRobert TwardoszFiorella SalvatoreRobert W. HensarlingFiorenzo MoscatelliRobert Walter MannFirat AkcaRobert WarnerFirda RahmadaniRobert WeidnerFirdoos Ahmad GogryRobert WellsFirdos Alam KhanRobert Yung-Liang WangFirman MenneRobert ZupkoFiroozeh SamimRoberta AdorniFirzan NainuRoberta BevilacquaFitri Fareez RamliRoberta Di GiacomoFlaminia FerriRoberta FoligniFlaminia FranchiniRoberta PalmourFlávia Aparecida GraçaRoberta RenatiFlavia FranconiRoberta RussoFlavia GhellerRoberta SalaFlavia M. SartiRoberto Abeldaño ZuñigaFlávia Noronha Dutra RibeiroRoberto Alonso González LezcanoFlavia ZacconiRoberto ApponiFlávio Antôniode Souza CastroRoberto BadaróFlavio De Alcantara CamejoRoberto Barcala-FurelosFlavio De Castro MagalhaesRoberto BaroneFlávio De Freitas MattosRoberto BarreraFlávio Gomes Borges TiagoRoberto BenoniFlávio HojaijRoberto CalistiFlávio LaraRoberto CannataroFlavio MilanaRoberto CastiglioneFlavio RodriguesRoberto CerchioneFlávio Sanson FogliattoRoberto ChiarelliFlavius RovinaruRoberto ChimenzFleischer C. N. KoteyRoberto CodellaFletcher NjororaiRoberto ColluFlora BacopoulouRoberto DemontisFlora N. BalievaRoberto Di CapuaFlorenc DemroziRoberto E. RojanoFlorence LebertRoberto FeltreroFlorence Mei Fung WongRoberto Fernandes CostaFlorence NeymotinRoberto FornaraFlorence Samkange-ZeebRoberto GarcíaFlorence VorspanRoberto GeladoFlorent SebbaneRoberto Giovanni Ramírez-ChavarríaFlorența-Diana TănaseRoberto H. ParadaFlorentina-Cristina MerciuRoberto Lo GiudiceFlorian BodescuRoberto LucchiniFlorian EmmerichRoberto MandrioliFlorian FiebelkornRoberto Martínez BeamonteFlorian FischerRoberto MartinsFlorian FollertRoberto MenèFlorian G. HartmannRoberto Merino-MartinezFlorian GambuśRoberto MerlettiFlorian GesslerRoberto MiceraFlorian HeilmannRoberto PaianoFlorian HeineRoberto PasiniFlorian K. EbnerRoberto PradoFlorian NutaRoberto S. FrancoFlorian PochsteinRoberto S. NettoFlorian ReckerRoberto San JoseFlorian SchmitzRoberto Sánchez-CabreroFlorian SchneiderRoberto Sanchis-SanchisFlorian StegerRoberto Sanz-PonceFlorian TheilmannRoberto ScendoniFloriana ZucaroRoberto StellaFlorica OrtanRoberto SussmanFlorigio Romano ListaRoberto VagnettiFlorin AonofrieseiRoberto ZefferinoFlorin GirbaciaRobin A. WilsonFlorin LeuciucRobin BakerFlorin MihaiRobin FearsFlorin MituRobin HackettFlorin MuselinRobin HaliouaFlorin NechitaRobin HankinFlorin OanceaRobin HealyFlorin OnisorRobin J. TyackeFlorin RADURobin KumarFlorin ZăinescuRobin L. CooperFlorina M. BojinRobin MazumderFlorina TeodorescuRobin MilhausenFlorin-Dumitru PetrariuRobin PlaFloris SchoetersRobin PurshouseFolajinmi OluwasinaRobin QuiggFolorunso FasinaRobin SamuelssonFong-Jia WangRobin Singh BhadoriaFoquan GuRobyn Braun-TrocchioForoud ShahbaziRobyn BruntonForrest BakerRobyn GershonFortunato BattagliaRobyn L. PrueittFortunato IacovelliRoca JudithFosco M. VeselyRocco Carmine ServidioFotini KehagiaRocco FrancoFotini VenetsanouRocco FrondiziFotios ChatzitheodoridisRocco HaaseFouad AbdullahRocco MeaFouad SakrRocco PapaliaFowzia AkhterRocco ServidioFran C. BlumbergRochelle SturtevantFran Hardin-FanningRocío A. BaqueroFrance WalletRocío Cruz-DíazFrances Mary JohnsonRocío De Andrés CalleFrances Meier-GibbonsRocío De Diego CorderoFrances PressRocío Juliá-SanchisFrances StillmanRocío Lago-UrbanoFrancesc Fusté-FornéRocío Llamas-RamosFrancesc VallsRocío López-LópezFrancesca AbastanteRocío Montoya-PérezFrancesca AmadoriRocío Ortiz AmoFrancesca ArezzoRocio Palomo-CarriónFrancesca BassiRocio RodriguezFrancesca BonaRocío Romero-CastilloFrancesca BorghiRocío Yñiguez OvandoFrancesca BorzìRock Keey LiewFrancesca BuonomoRocky J. DwyerFrancesca CattoniRocsana Bucea-Manea-TonisFrancesca CiampaRoddy HiramFrancesca CucinottaRodgers MakwinjaFrancesca D’ELIARodica Elena IonescuFrancesca D’ErricoRodica-Mihaela DinicǎFrancesca De FalcoRodman E. TurpinFrancesca DemichelisRodney DuffettFrancesca Di GiallonardoRodney P. JonesFrancesca Di PilloRodolfo AllendesFrancesca Di VirgilioRodolfo Andre DellagranaFrancesca DiolaiutiRodolfo BuselliFrancesca DiomedeRodolfo FagliaFrancesca ErvasRodolfo Iván Martínez-LemosFrancesca FedericoRodolfo MauceriFrancesca FerrarisRodolfo Morrison JaraFrancesca FerroniRodolfo RedaFrancesca FilonRodrigo A. Guzmán-VenegasFrancesca GallèRodrigo Alejandro Hidalgo DattwylerFrancesca GastaldiRodrigo Antunes LimaFrancesca GiglioRodrigo Arturo Zárate-TorresFrancesca LatinoRodrigo F. O. PenaFrancesca LionettoRodrigo Fernando Dos Santos SalazarFrancesca MagliRodrigo Filev MaiaFrancesca MeliniRodrigo Gomes Da RosaFrancesca MiselliRodrigo HohlFrancesca MorettiRodrigo Martín-San AgustínFrancesca NegroRodrigo NovaFrancesca NoccaRodrigo PardoFrancesca PennucciRodrigo Poblete UmanzorFrancesca PisanoRodrigo Proença De OliveiraFrancesca Romana LugeriRodrigo RamalhoFrancesca RossiRodrigo ResendeFrancesca SilvagnoRodrigo Rodrigues De FreitasFrancesca TessitoreRodrigo RosaFrancesca VannucchiRodrigo Torres-CastroFrancesca VariaRodrigo ValenzuelaFrancesca VichiRodrigo Vargas VitoriaFrancesca ZaraRodrigue PignelFrancesco AgostiniRodríguez-Fernández Carmen AntíaFrancesco AlettaRoel Van OvermeireFrancesco AsciRoemi FernandezFrancesco BalestriRogelio De Jesus Portillo-VelezFrancesco BarberoRogelio Flores-RamírezFrancesco BarrecaRogelio González-GonzálezFrancesco BiancoRoger FigueroaFrancesco BimboRoger HoltenFrancesco BochicchioRoger JensenFrancesco BoscoRoger JonesFrancesco CadarioRoger K. GreenFrancesco CampaRoger PenlingtonFrancesco CarboneRoger Pielke Sr.Francesco CaridiRoger RamsbottomFrancesco CarocciaRoger RochatFrancesco CastagnettiRoger Saint-FortFrancesco CauteruccioRoger SeilerFrancesco CeresiaRogério César FerminoFrancesco ChelliRohail HassanFrancesco ClapsRohan JadhavFrancesco ClementiRohan KapoorFrancesco ComacchioRohan NagareFrancesco CoreaRohanaFrancesco CraigRohil BhatnagarFrancesco D. D’OvidioRohit JainFrancesco De PascaleRohit P. DugarFrancesco DemariaRohit TanwarFrancesco Di BelloRohitashw KumarFrancesco Di GennaroRok FinkFrancesco Di LorenzoRok GaspersicFrancesco Di NataleRoksana MuzykaFrancesco FasanoRokshana ParvinFrancesco FelicettiRoland BalFrancesco FerriniRoland DielFrancesco FischettiRoland GärtnerFrancesco ForastiereRoland Kueh Jui HengFrancesco Francini PesentiRoland MooreFrancesco GallettiRolando Rubilar-TorrealbaFrancesco GastaldiRolf HolleFrancesco GherardiniRolf LarssonFrancesco GrimaldiRolf-Detlef TreedeFrancesco InchingoloRomain NoëlFrancesco La RussaRoman AndrzejakFrancesco LicciardoRoman GablFrancesco MaffìaRoman HornungFrancesco MantaRoman KrálikFrancesco MarcattoRoman LeischikFrancesco Maria ChelliRoman MoskalenkoFrancesco Maria FuscoRoman PavliukFrancesco Maria GalassiRoman PiotrowskiFrancesco MartinoRoman RolbieckiFrancesco MaseduRoman S. NagovitsynFrancesco MassariRoman SmolarczykFrancesco MassoniRoman VýletaFrancesco MeneguzzoRomana Koberová IvančakováFrancesco MongelliRomana Korez VideFrancesco MoraRomana MoutelíkováFrancesco MusacchiaRomana PerkovićFrancesco NapolitanoRomana StehlikFrancesco NicoliRomea Manojlovic TomanFrancesco OrziRomeo DobrinFrancesco PaccheraRomil ParikhFrancesco PaceRomina FucàFrancesco PallottiRomina KrausFrancesco PisanuRomina Marina SimaFrancesco PistelliRomina NabieeFrancesco ProcaccioRomina PaceFrancesco RomanoRomina VivaldiFrancesco S. ViolanteRomuald ZabielskiFrancesco SalzanoRomuald ZalewskiFrancesco SanmarchiRomualdas MalinauskasFrancesco Saverio LudovichettiRon KedemFrancesco ScibelliRon PritzkuleitFrancesco SegalaRon RapeeFrancesco SessaRon SaulnierFrancesco SgròRon van LammerenFrancesco SpinelliRonak ReshamwalaFrancesco SullaRonald B. BrownFrancesco TaddeoRonald B. MooreFrancesco TessaroloRonald Cheong Kin ChanFrancesco TommasiRonald EvansFrancesco VenneriRonald FallerFranchek DrobnicRonald HinchFrancilene Gracieli Kunradi VieiraRonald Huarachi OliveraFrancina FonsecaRonald J. SigalFrancina Lozada NurRonald RoopnarineFrancine Amaral PiubeliRonald SnijderFrancine CournosRonaldo CozzaFrancine M. OvercashRonaldo Vagner Thomatieli-SantosFrancis Enejo IdachabaRonan LordanFrancis F. PavloudakisRónán O’CaoimhFrancis MitrouRondy YuFrancis MuchaambaRonei De AlmeidaFrancis NkrumahRongfang LyuFrancis OlooRong-Fu ChenFrancis RaynsRongrong LiuFrancis RiesRonit KorenFrancis Yue-lok CheungRonnie J. Araneda-CabreraFrancisca Alves CardosoRoohollah NooriFrancisca Angélica Monroy GarcíaRoohollah RostamiFrancisca OmorodionRoozbeh Abedini NassabFrancisca Pereira De MoraesRoozbeh Haghnazar KoochaksaraeiFrancisca S. RodriguezRoozbeh MoazenzadehFrancisco A. Burgos-JuliánRory D. WattsFrancisco AlegreRos KaneFrancisco Alejandro Lagunas-RangelRos SambellFrancisco AlenRosa AisaFrancisco Alexandrino JúniorRosa Ana Alonso Alonso RuizFrancisco AlonsoRosa Angela FabioFrancisco AmadoRosa ArroyoFrancisco ArmijoRosa Cândida Carvalho Pereira De MeloFrancisco ArnalichRosa ColuzziFrancisco BrocalRosa Devesa-ReyFrancisco BuitragoRosa M. Cárdaba-GarcíaFrancisco Caamaño IsornaRosa Magallón-BotayaFrancisco Campello Amaral MelloRosa María Giráldez-PérezFrancisco CarameloRosa Maria N. MarcussoFrancisco Cartujano-BarreraRosa María Oliart-RosFrancisco CesárioRosa Maria Perez CanaverasFrancisco CordobaRosa Maria Tapia-HaroFrancisco D. RodriguezRosa Martha Meda LaraFrancisco Del Cerro VelázquezRosa Martínez-PiédrolaFrancisco Entrena-DuranRosa Posada-BaqueroFrancisco EpeldeRosa RedolatFrancisco Eugenio Calvo-IglesiasRosa Roig BerenguerFrancisco FernándezRosa Rosario-RosadoFrancisco Flor-MontalvoRosa ScardignoFrancisco García-Muro San JoséRosa VallettaFrancisco Gonzalez FernandezRosa Wanda Diez-GarciaFrancisco Gonzalez-SalaRosa WongFrancisco Guede-RojasRosa-Eva Valle-FlórezFrancisco Guillen-GrimaRosales-Hernández Martha C.Francisco Guzman PrunedaRosalia Contreras-BulnesFrancisco Haces-FernandezRosalia CrupiFrancisco IniestoRosália SáFrancisco J. Martínez-OlmosRosalie CallwayFrancisco J. MedinaRosalie GreenbergFrancisco Javier Cano-GarcíaRosalie PowerFrancisco Javier CristòfolRosalie ThackrahFrancisco Javier De La BallinaRosalina Pisco CostaFrancisco Javier del Río OlveraRosalina TameleFrancisco Javier Fernández CarrascoRosamaria Kostic-CisnerosFrancisco Javier Gago ValienteRosa-María Rodríguez-JiménezFrancisco Javier Herrero GutierrezRosana ShuhamaFrancisco Javier Jiménez RíosRosane Härter GriepFrancisco Javier Martín-AlmenaRosanne A. CouttsFrancisco Javier Orquín-CastrillónRosapia Lauro-GrottoFrancisco Javier Paniagua-RojanoRosaria CalifanoFrancisco Javier Peralta-SánchezRosaria GesuitaFrancisco Javier Pérez-RivasRosaria MeccarielloFrancisco Javier Pilar-OriveRosaria ScudieroFrancisco Javier Povedano-MonteroRosario J. MarreroFrancisco Javier Robles MoralRosario MegnaFrancisco Jesús Gálvez-SánchezRosario PastorFrancisco Jesús García-NavarroRosario SommellaFrancisco Jose Berraldela RosaRosario UndurragaFrancisco José del Campo-GomisRosaura LeisFrancisco José Eiroa-OrosaRosaura Mayén-EstradaFrancisco José Fernández-GómezRose CalixteFrancisco José García-MoroRose GilroyFrancisco Jose García-SanchezRose Marie O. MendozaFrancisco José Hernández-PérezRoseli Rodrigues De MelloFrancisco José HidalgoRosemarie Felder-PuigFrancisco José Medina AlbaladejoRosemary CaronFrancisco José Nunes AntunesRosemary KellyFrancisco José SantonjaRosemary SevaFrancisco Jose Sarabia SanchezRosendo BerengüíFrancisco José Tarazona SantabalbinaRoser GraneroFrancisco López-CantosRoseriet BeijersFrancisco LozanoRosetta LombardoFrancisco Manuel BaenaRoshan KumarFrancisco Manuel Morales RodríguezRoshan Lal KoulFrancisco Manuel Moreno-PinoRosibel Rodríguez-BolañosFrancisco MendezRosie GibsonFrancisco MesaRosie HardingFrancisco Miguel Escandell RicoRosilawati ZainolFrancisco MonteiroRosimeire Nunes De OliveiraFrancisco Moreno-SanchezRosita BorlimiFrancisco Navarro ValverdeRoslan IsmailFrancisco Nieto-EscamezRoslina IsmailFrancisco PerezRoslyn W. LivingstoneFrancisco Reyes-SantiasRosmah Mat IsaFrancisco Rodríguez-CifuentesRoss GarnerFrancisco Rosa NetoRoss HillFrancisco RuizRoss LarueFrancisco SaavedraRoss PruittFrancisco SalamancaRoss ThomsonFrancisco Sánchez-AlberolaRossana AlloniFrancisco Teixeira Da SilvaRossana C. ZepedaFrancisco Triguero-RuizRossana IzzettiFrancisco TustumiRossana MorabitoFrancisco Villegas LirolaRossana RanieriFrancisco-Ignacio Revuelta-DomínguezRossana ScutariFrancisco-Javier Cantero-SánchezRossella LianiFranciszek GrabowskiRossella MarmoFranco BagnoliRossella MoscarelliFranco BassettoRossella SgarzaniFranco GemignaniRossettini GiacomoFranco GiraudoRoswith RothFranco RabbiaRo-Ting LinFranco Sancho-EsperRoto RotoFranco ZaniniRoumen VesselinovFrançois Borel-HänniRowalt AlibudbudFrançois LalondeRowena IversFrançois PhilipponRowida MohamedFrancoise SailhanRoxana Acosta GutiérrezFrancois-Pierre CounilRoxana Adriana StoicaFrane PaicRoxana Liana LucaciuFranjieh El KhouryRoxana MareFranjo MijatovićRoxana Maria BădîrceaFrank AndrasikRoxanne BavarianFrank ChenRoxanne BerubeFrank E. NelsonRoy AloniFrank M. MarchakRoy Alvin MalenabFrank Mayta-TovalinoRoy BeranFrank R. ChenRoy McConkeyFrank R. HalfwerkRoy NagleFrank RomanelliRoy RobertsonFrank TrovatoRoy SetiawanFrank WeberRozalia VanovaFrank WernitzRozeta SokouFranklin Delano Soares ForteRoziah Mohd RasdiFranklin W. N. ChowRozina KhattakFrank-Peter TillmannRuben A. MarsFrantišek BabičRubén Abraham Domínguez-PérezFrantisek BuzekRubén Arroyo FernándezFrantisek ChmelikRuben FossionFrantišek MilichovskýRúben FranciscoFrantišek PetrovičRuben FukkinkFrantišek PollákRubén López-BuenoFrantišek ZahálkaRubén Navarro PatónFranz ZehentmayrRubén RaedoFranziska KrebsRubén Rivas-de-RocaFranziska KühneRubén Vázquez MedinaFranziska PapenfußRuben VrijhoefFranziska PerelsRubí Rodríguez-DíazFranziska WeberRubie JacksonFranziska WelzelRubinia Celeste BonfantiFraser CarsonRuchira GhoshFred BidandiRüdiger PryssFred Gustavo Marique-AbrilRudolf KuklaFred LurmannRudolf PsottaFred MasonRudra P. AryalFred Van RaaijRuei-Nian LiFreda McCormickRufaro GaridziraiFreddy BrownRuggero Andrisano RuggieriFreddy Marín-GonzálezRugivan SabaratnamFrédéric ClercRui BertuziFrederic De MarizRui C. MartinsFrederic DenisRui DangFrédéric DumurRui DingFrédéric GrecoRui JiangFrederic HarbRui Jorge Rodrigues da SilvaFrederic JungbauerRui LançaFrédéric OuimetRui LiFrederic SchoenbergRui LinFrédéric VanderhaegenRui M. FilipeFrédéric VellaRui MatosFrederic Woodward HopfRui Miguel DantasFrederick A. Clive WrightRui NevesFrederick Jan MednickRui Pedro BritoFrederick PhilippeRui Pedro JuliãoFrederico VieiraRui PoínhosFrederik PetersRui TorresFrederike T. FellendorfRui YanFrédérique ServinRui ZhangFredric GovedichRuibin ZhangFredric SchifferRuifeng HuFredrik MolinRuifeng YuFredrik NorströmRuijie WangFredrik SjödinRuilong ShengFredrik VelanderRuimin FengFreek LapréRuipeng TongFreya TyrerRuipu MuFrida BettoRuiqiu ZhangFrieder KrafftRuixin YangFriederike BarthelsRuixue HouFrikkie J. W. CalitzRu-Jeng TengFrits Van Der EerdenRukman Awang HamatFrits Van MerodeRukmani PandeyFritz KahlRukshan MehtaFrontistis ZachariasRu-Lan HsiehFuad AmeenRulan JiangFuad Hamdi A. AbuadasRully PrahmanaFu-Gong LinRumi TanoFu-Hsuan ChenRumin LuoFulden Ulucan KarnakRun Zhou YeFulgencio Sánchez VeraRundk HwaizFulvio AdorniRungruang JantaFulvio LauretaniRungtai LinFulvio PlesciaRun-Ping CheFulvio SignoreRunpu ChenFulya OzerRuntang MengFumi MasudaRunzeng LiuFumiaki FujibeRuobing QianFumihiro OgawaRuojun WangFushan TangRuoxing WangFushing HsiehRuoyan SunFüsun ÜlenginRupert HindsFuu Ming KaiRupert W. StraussFuyi LiRusan CatarFuyuan WangRu-Si ChenFuyuan XuRuslan KravetsGábor KardosRuslans SmiginsGábor MocsárRussell D’SouzaGábor Nagy-GróczRussell EisenmanGábor Szabó-SzentgrótiRussell KabirGabriel Alomar-GarauRussell PittmanGabriel Brito CostaRustem IlyasovGabriel C. TenderRustem MustafaogluGabriel Dias RodriguesRusty BarrettGabriel Dorantes-ArgandarRūta UstinavicieneGabriel G. Dela TorreRute SantosGabriel González-ValeroRute Sofia Monteiro SampaioGabriel GulisRūtenis PaulauskasGabriel KolvekRuth Adisetu PobeeGabriel L. SchwartzRuth BreezeGabriel López-MartínezRuth C. CronkiteGabriel MustateaRuth D. LipmanGabriel Núñez-NogueiraRuth E. McKenzieGabriel Rodriguez-RomoRuth E. Taylor-PiliaeGabriel RojasRuth Esther Villanueva-EstradaGabriel RubinRuth IrwinGabriel SadiRuth L. SteinerGabriel StefanRuth Maldonado-RengelGabriel YesufRuth PlackettGabriel Zorello LaportaRuth S. HwuGabriela AzocarRuth SnyderGabriela Breaban IulianaRuth SpeidelGabriela BuemaRuth TappenGabriela ChiciudeanRuth WaitzbergGabriela Cristina KüppersRuth WillsGabriela De Morais Gouvêa LimaRuthmarie Hernández-TorresGabriela DimaRuwei LinGabriela DrocRuxandra BejinaruGabriela DrojRuxandra Diana SinescuGabriela E. LeghiRuxandra FolostinaGabriela Elena DumitranRuxandra Malina Petrescu-MagGabriela GabajováRuxandra Sava-RosianuGabriela GarcíaRuxandra StefanescuGabriela IonescuRužena KrálíkováGabriela KiryakovaRuziana MasiranGabriela KołodyńskaRwei-Ling YuGabriela KowalskaRwik SenGabriela López AymesRyad BouzouidjaGabriela Loredana PopaRyan CausbyGabriela M. WilsonRyan Cory MavesGabriela Mihaela MuresanRyan GibsonGabriela MorosanuRyan GordonGabriela MuñozRyan H. L. IpGabriela O. ChiciudeanRyan McGrathGabriela PetcuRyan Mead-HunterGabriela Quintana VigiolaRyan T. ShieldsGabriela ShirkeyRyan WornGabriela TopaRyland MorgansGabriela Ventura A. SilvaRylee DionigiGabriele CervinoRyo MaekawaGabriele CiciurkaiteRyo MomosakiGabriele ColaRyo NakamaruGabriele D’OrsoRyoichi AsakaGabriele Di FrancescoRyoko AsanoGabriele FincoRyoma AsahiGabriele GhettiRyota InokuchiGabriele GrunigRyota SakamotoGabriele LombardiRyota ShizuGabriele MascheriniRyouichi SatouGabriele MelegariRyszard BlazejewskiGabriele MeroniRyszard GrendaGabriele MorucciRyszard MalinowskiGabriele MulliriRyszard PukałaGabriele NibbioRyszard ŚwietlikGabriele SacconeRyszard ZwierzchowskiGabriele TodiscoRyun-Seok OhGabriella BottiniS. Camille PeresGabriella CasalinoS. M. Lutful KabirGabriella D’AmoreS. M. Sohel MahmudGabriella DvorakS. M. Sohel RanaGabriella Esposito De VitaS. M. Yasir ArafatGabriella GraziusoS. PriyanGabriella MarféS. RamkumarGabriella MartinoS. SasikalaGabriella OlmoS. Zaung NauGabriella PiscopoSa LiuGabriella PunzianoSaad SalemGabriella SantangeloSaaima HasninGabriella TognolaSaba Ahmed BeighGabriella VerucchiSabahudin HadžialićGad SegalSabba MehmoodGadaf RexhepiSabeeh Lafta FarhanGaëlle DanieleSaber SaedmocheshiGaetan BlandinSabina AngreckaGaetano Maria FaraSabina Antonela AntoniuGaetano PaoloneSabina Jakóbczyk-KarpierzGaetano PolizziSabina KrupaGaetano PriviteraSabina La GruttaGaetano RaiolaSabina LicenGaetano RiemmaSabina MandicGaetano SabatoSabina N. ValenteGagan Deep SharmaSabina RanjitGagandeep KaurSabina StanescuGaia BarisioneSabina ValenteGaia GherardiSabina VatterGail ReesSabine HahnGainel UssatayevaSabine J. RozaGajendra JogdandSabine Panzer-KrauseGalateja JordakievaSabine Sayegh-JodehlGalder KortaberriaSabine Van Der AsdonkGalina S. ZhamsuevaSabino PachecoGalina ShinkarevaSabinus Oscar Onyebuchi EzeGalya Georgieva-TsanevaSabreen Ezzat FadlGamal AtallahSabri KanzariGamal MohamedSabrina BonichiniGamaliel BléSabrina DemarieGamze OzelSabrina MartinezGamze VarankSabrina MolinaroGanapathy NatarajanSabrina RoguaiGanesh SableSabrina StranoGanesh SinhaSabrina ŠumanGang ChenSabuj Kanti MajumderGang FuSabyasachi ChatterjeeGang HuSabyasachi MoulikGang KouSachiko InoueGang PengSachiko TakeharaGangadhar AndaluriSachio TakenoGaofeng LiuSachio TsuchidaGareth JohnsonSachit AnandGarima DwivediSachiyo MoritaGarima KaushikSadaf JahanGarry KuanSadahisa KatoGarth KendallSadam HussainGary A. RoselleSaddam HussainGary BriersSaddam Q. WaheedGary ClaptonSaddam SaqibGary CraigSadeq Al-FayyadhGary D. RaysonSadia SaifGary FranklinSadia Samar AliGary GlaubermanSadia ZafarGary HooperSae OchiGary J. KennedySaeed AnwarGary Li-Kai HsiaoSaeed GhorbaniGary MillerSaeed MoridGary PotterSaeed MozaffariGary SchwartzSaeed Nazari KudahiGary Scott EarnestSaeed ShojaeiGary StoreySaeed YousefinejadGary WingenbachSaei Ghare Naz MarziehGaryfallia PeperaSaeid AminiGasim HayderSaeid HosseinzadehGašper KlančarSaeideh Valizadeh-HaghiGastón AresSae-Yeon WonGator YudokoSafwan OmranGaurav DhawanSagadevan SureshGaurav Kumar SharmaSagar MaitraGaurav RainaSagar PandeGaurav Raj DwivediSagit LevGaurav V. SarodeSagrario Gomez-CantarinoGautam PandeySagrario Martín-AragónGautam PareekSagrario Pérez-De La CruzGautham YepuriSahar DerakhshanGauthaman SukumarSahar HoushdaranGavendra PandeySahar SoltaniGavin LaiSahar ZavarehGavin MoirSaheli NathGavriil George ArsoniadisSahizer Samuk CarignaniGavril GrebenisanSai Pranathi Meda VenkataGavril StefanSai Reddy DodaGayan RubasinghegeSai Shilpa KommarajuGayathri Devi MekalaSaïd MekaryGayathri NatarajanSaif KhanGayo DialloSaifei LeiGazi Mahabubul AlamSai-fu FungGbadebo Clement AdeyinkaSaija-Liisa KankaanpääGbekeloluwa B. OguntimeinSaijun ZhangGbemi OluleyeSaikat BalaGea Oliveri ContiSaima SohniGeanina Cucu CiuhanSaionara Maria Aires CamaraGecheng YuanSairam BeheraGede SuantikaSait KaramanGeert DomSaja KosanovićGeert MayerSajad BorzoueisilehGeeta HitchSajana MaharjanGeidy E. SerranoSajid AliGema Aonso-DiegoSajid KhanGema FrühbeckSajid Khan TahirGema Pérez-RojoSajid SoofiGema Sanchis-SolerSajjad AhmadGema VichoSajjad HussainGemma Castano-VinyalsSajjad MalikGemma Caterina Maria RossiSajjad U. AhmadGemma HammertonSajjakaj JomnonkwaoGemma PratSaju Madavanakadu DevassyGemma RenartSakari B. SuominenGemma SkaczkowskiSaket A. KottewarGen LiSakiko KanbaraGen NakayamaSakine ShekoohiyanGenevieve HealySakineh HajebrahimiGeneviève MarchandSakineh VarmazyarGennady KolesnikovSakorn MekruksavanichGennaro RuggieroSakshi SardarGennaro SelvaggiSakthivel KumaravelGennaro VenosoSal ConsoliGenovaitė LiobikienėSalah A. MohamedGentaro KumagaiSalahaldin AlshatshatiGenuario BelmonteSalam DhouGeoff ArgusSalam Salloum-AsfarGeoff M. BoucherSaleh AminyavariGeoffrey D. KahnSalem S. SalemGeoffrey DavisonSalesa BarjaGeoffrey MadeiraSalil DeoGeonwoo KimSalim HeddamGeorg BolligSalim MorammaziGeorg SchillerSalima MeheraliGeorg SingerSalina JantarangGeorge A. MarcoulidesSallie Beth JohnsonGeorge A. TavartkiladzeSally DemirdjianGeorge A. TsalisSally Fowler-DavisGeorge A. ZangaroSally GuttmacherGeorge AbongSally NashGeorge AndersonSally ShermanGeorge AphamisSally ThorneGeorge BarakosSalma AbedelmalekGeorge ChingarandeSalma QasimGeorge CrooksSalman KimiagariGeorge Danut MocanuSalman ShooshtarianGeorge EvangelouSaloua BiyadaGeorge GellertSalvador BaenaGeorge ImatakaSalvador García-Ayllón VeintimillaGeorge JamilSalvador PérezGeorge JenningsSalvador RambaudGeorge JîtcăSalvador Romero-ArenasGeorge KatavoutasSalvador Sánchez-CarrilloGeorge Laurenţiu Şerban-OprescuSalvador Vergara-LópezGeorge LăzăroiuSalvatore BocchieriGeorge M. PamborisSalvatore CocuzzaGeorge ManisSalvatore G. De-SimoneGeorge MarisSalvatore Giovanni De SimoneGeorge MichaelidesSalvatore GiuffridaGeorge MichailSalvatore IaconoGeorge ObaidoSalvatore LeonardiGeorge P. SillupSalvatore MonacoGeorge R. KasyanSalvatore RomanoGeorge RachiotisSalvatore SarubbiGeorge RamosSalvatore ScaccoGeorge RaymentSalvatore ScolavinoGeorge S. KorresSalwa El TawilGeorge S. NyamatoSam JohnsonGeorge SoklisSam OxfordGeorge SorokaSam SalehianGeorge SoulisSam StrainGeorge SuciuSamander KaushikGeorge TheodossiouSamantha C. SodergrenGeorge UmemotoSamantha DaveyGeorge W. LiechtiSamantha HahnGeorgi PanovSamantha L. RedmanGeorgi ZhelezovSamantha MajicGeorgia GomatouSamantha MoraisGeorgia NikoloudakiSamantha MossGeorgia PanagiotakiSamantha Sze Wan ShanGeorgia PapantoniouSamantha Tamara Jimenez OyolaGeorgia PapoutsiSamar HelouGeorgia VourliSamar Kamal KassimGeorgina FauraSamar TharwatGeorgina Galicia-RosasSamara Urban De OlivaGeorgina Mayela Núñez-RochaSameh AttiaGeorgina Silva-SuárezSamer A. KharroubiGeorgios BamposSamera HamadGeorgios BartzasSamet ÖzdemirGeorgios D. PanosSami Abou FayssalGeorgios DimakopoulosSami AkbulutGeorgios GalaziosSamia AminGeorgios GiarmatzisSamia ElseginyGeorgios GrivasSamiha ChemliGeorgios IatrakisSamina AbidiGeorgios KanavakisSamina SalimGeorgios KatsarasSamineh MesbahGeorgios LaliotisSamir Kumar BandopadhyayGeorgios MarinosSamira Alfayumi-ZeadnaGeorgios PampalakisSamira SalihovicGeorgios PapagiannisSamira Samiee-ZafarghandyGeorgios PatsiaourasSamira TarashiGeorgios TsantopoulosSamo RauterGeorgios TsekouropoulosSamrad MehrabiGeorgios TsiavaliarisSamrita DograGeorgiy KorobeynikovSamson G. KhachatryanGeovani Lopez-OrtizSamson TanGera MeetaSamsul Ariffin Abdul KarimGerald AranoffSamsuri AbdullahGerald FerrettiSamuel A. IwarereGerald H. TomkinSamuel A. KareffGerald JeromeSamuel BaxterGerald M. MkojiSamuel CaitoGerald MboowaSamuel ChngGerald McKinleySamuel Durán-AgüeroGerald VoelbelSamuel E. TannerGerald ZernigSamuel Fernández CarneroGeraldes VítorSamuel Fernández-SalineroGeraldine GueronSamuel García-ArellanoGeraldine TorrisiSamuel José Fonseca MonteiroGeraldo A. Maranhao NetoSamuel Kofi ErskineGeraldo Cardosode Oliveira NetoSamuel López-CarrilGerard A. KennedySamuel MatthewsGerard DumancasSamuel McKayGerard LevySamuel NutileGerard M. MoloneySamuel OtterstromGerard Marshall RajSamuel OxfordGerard McMahonSamuel ShiGérard MerlinSamuel SilvestreGerardo Arturo López MuñozSamuel TomczykGerardo CarusoSamuele MarinelloGerardo CazzatoSamy Mahmoud H. SayedGerardo Cebrián-TorrejónSamy RimaGerardo FebresSana MahtabGerardo Fernández BarberoSanaa Najeh Al-Haj AliGerasimos RazisSanath KumarGerd LuppSanaul Haque MondalGerd Marie AleniusSanaz ChamanaraGerd U. AuffarthSanaz SalatiGerdt SundströmSanchia ArandaGereon ElbersSanda Cristina OanceaGergana PetrovaSanda TanGergely DarnaiSandeep JagtapGergely JakabSandeep KondakalaGergely RáthonyiSander HilberinkGergo TothSandesh PanthaGerhard NortjéSandhiran PatchayGerhard ReeseSandi Baressi ŠegotaGerman Dominguez VíasSandip RoyGerman Eduardo DevoraSandor BenyheGermán Hernández CruzSándor BeszédesGerman PerezSándor RózsaGermano RossiSandra A. HartasanchezGermina-Alina CosmaSandra AlhoyekGero BenckiserSandra AndrušaitytėGerson PechSandra BarbalhoGert WagnerSandra BrkanovićGertrudes Saúde GuerreiroSandra C. ButtigiegGeta MitreaSandra CastilloGetachew A. DagneSandra CortésGetahun Ersino LombamoSandra D. AdamsGeu Mendoza CatalánSandra DixonGeva ShenkmanSandra FigueiredoGhada RegaiegSandra FoltinGhaffar AliSandra GavinhaGhasem FarahmandSandra GrabowskaGhazanfar AnwarSandra HirschGhazi RacilSandra J. WinterGhefar FuraijatSandra Jiménez-Del-BarrioGheorghe DărăbuşSandra KusGheorghe H. PopescuSandra McFaddenGheorghe LazaroiuSandra Milena Colorado-YoharGheorghe N. PopescuSandra PetrauskieneGheorghe-Daniel VoineaSandra RebeloGheorghe-Gavrilă HognogiSandra RecueroGheorghita DincaSandra RibeiroGheorghița NistorSandra RodriguesGhislain HardySandra SánchezGhislaine L. LewisSandra Sanmartin FeijooGhislaine MayerSandra Santos-MartinezGholamreza GoudarziSandra SoaresGhorban TaghizadehSandra Sumalla-CanoGhulam AbidSandra TrzcińskaGhulam MurtazaSandra V. ConstantinGhulam Rasool MadniSandra VujkovGiacinto BarresiSandra Wajchman-ŚwitalskaGiacoma GalizziSandra ZampieriGiacomo AngeliniSandrah P. EckelGiacomo BaimaSandrina Berthault MoreiraGiacomo BalduzziSandrina TeixeiraGiacomo BianchiSandrine BayleGiacomo BrunoSandrine SelosseGiacomo DeianaSandrita SimonyteGiacomo FarìSandro Arrufat-MartínGiacomo FerrettiSandro Fernandes Da SilvaGiacomo MediciSandro Ferreira VeigaGiacomo NalliSandro GentileGiacomo PallanteSandro Luis SchlindweinGiacomo Pietro VigezziSang Bong LeeGiacomo PignataroSang Hyeon LeeGiada BenasiSang Kyu LeeGiada MinelliSang LeeGiada TripoliSang Ouk WeeGiampaolo NicolaisSangamanatha Ankmnal VeerannaGiampaolo SantiSangchun ChoiGiampiera BulfoneSang-Dol KimGiampiero TarantinoSang-Guk YumGiampietro AlbertiSanggyun NaGiampietro FarronatoSang-Hee ParkGian Franco CapraSang-Heng KokGian Franco ZannoniSang-Ho KimGian Luca ChiarelloSanghoon JangGian Luigi CanataSanghoon KangGian Luigi NicolosiSanghyuk BaeGian Marco ContessaSang-Jib KwonGian Marco DumaSang-Joon KimGian Maria GaleazziSanglim LeeGian Maria GrecoSangram SamalGian Mario MigliaccioSang-Seok NamGian Pietro SechiSangsoon LimGianandrea PasquinelliSang-Sup LeeGiancarlo CondelloSangwoo SungGiancarlo CuadraSania QureshiGiancarlo PecorariSanja FrancGiane G. LenziSanja KezicGianfrancesco FioriniSanja KovačićGianfranco FaviaSanja Lovric KojundzicGianfranco NataleSanja Music MilanovicGianfranco SantovitoSanjay KumarGiang PhamSanjay MohanGianluca CiardiSanjay RathodGianluca EspositoSanjay Singh RathoreGianluca LanzaSanjeeb MandalGianluca M. TartagliaSanjeet K. PandaGianluca MattarocciSanjid ShahriarGianluca NazzaroSanjoy SahaGianluca Raffaello DamianiSanne VeldmanGianluca RizzoSanthana Krishna Kumar AlagarsamyGianluca SerafiniSanthosh Kumar BalanGianluca TartagliaSanti MandalGianluigi CoppolaSantiago BallazGianluigi DorelliSantiago Criollo-CGianluigi SalvucciSantiago GascónGianmarco AbbadessaSantiago Giraldo LuqueGianmarco LugliSantiago Gomez-PaniaguaGianmaria D’AddazioSantiago HenaoGianmaria SalvioSantiago Martínez-IsasiGianna Fiori MarchioriSantiago MoraGianna MontiSantiago Navarro LedesmaGianni CappelliSantiago Pineda-MetzGianni Di GiorgioSantiago Tejedor CalvoGianni LoboscoSantiago UrrejolaGianni TestinoSantiago ZabaloyGianni VirgiliSantosh DhakalGiannicola IannellaSantosh K. YadavGiannino MelottiSantosh Subhash PalmateGianpaolo Di BonaSanwei HeGianpaolo VaccarellaSaqib BashirGianpaolo ZammarchiSaqib SaeedGibson Monica PrasadSara A. QuandtGibson PraçaSara A. WingesGideon KrusemanSara B. DonevantGiedre BulotieneSara BaldassanoGiedrė LapinskienėSara BernardiGigliola D’AngeloSara CabanillasGihoon HongSara ComaiGil Baptista FerreiraSara CorreiaGil FraquezaSara Cortés AmadorGilbert AhamerSara De BruynGilbert LimSara De CarolisGilbert Nchongboh ChofongSara DiogoGilbert RamirezSara Domínguez-LloriaGilbert W. FellinghamSara E. BenhamGilberto B. FischerSara E. LuckhauptGilberto Castañeda-HernándezSara FariaGilberto CorsoSara Fernández-AguayoGilberto GalindoSara FilipiakGilberto Gonzalez-ParraSara FulignatiGilberto Pérez LechugaSara García-BravoGilberto SammartinoSara GarfieldGilberto TadeuSara Gil RodríguezGill A. Ten HoorSara GuerraGill NelsonSara Huerta-GonzálezGill WatersSara JayousiGillian LawsonSara L. KinterGillie Pragai OlswangSara LappanGilmário Ricarte BatistaSara Larsson LönnGilson Brito Alves LimaSara LunaGina AbdalaSara MaioGina Delia Roque-TorresSara Martínez-CarreraGina HarperSara McNeillisGina HeathcoteSara Morón-LópezGina Ionela ButnaruSara MucherinoGina Rio DedelesSara NamaziGiner Alor-HernándezSara NunesGinny LaneSara Ojeda-BenitezGinny ZhanSara Palomo-DíezGino GrifoniSara PietracupaGintare KalinienėSara Raquel Boaventura RodriguesGintare VaznonieneSara RicciardiGintarė VaznonienėSara SaliniGioele CastelliSara SantiniGioele GavazziSara SilvaiehGionata FragomeniSara SkandraniGiorgia CasaloneSara SousaGiorgia CinelliSara SpadoniGiorgia Dalla SantaSara TargonskaGiorgia VaralloSara TodeschiniGiorgio AiroldiSara WawrzyniakGiorgio BasileSara ZagonariGiorgio BedogniSara ZapicoGiorgio CalloviniSara ZedakerGiorgio OrlandoSara ZellaGiorgio PolettiSarabjit MastanaGiorgio RappelliSarah AherfiGiorgio SantoroSarah BrowneGiorgio SmaldoneSarah BurkartGiorgos AlevizopoulosSarah Cirilo GimenesGiovana Goretti Feijó De AlmeidaSarah CommodoreGiovanna Barbarini LongatoSarah EisslerGiovanna BarzanòSarah GephineGiovanna Calixto AndradeSarah HancockGiovanna CampaniSarah Hian May ChanGiovanna CaparelloSarah J. Breese McCoyGiovanna CasiliSarah KellerGiovanna CentorrinoSarah KipermanGiovanna D’EliaSarah Kittel-SchneiderGiovanna Elisa CalabròSarah LutzGiovanna GhianiSarah M. RayGiovanna Maria DimitriSarah ManessGiovanna MurmuraSarah MooreGiovanna OrsiniSarah MortonGiovanna PerriconeSarah NaiyerGiovanna PerrottiSarah NutterGiovanna PiangiamoreSarah P. MaxwellGiovanna PisacaneSarah PriorGiovanna RicciSarah ReedmanGiovanna ZimatoreSarah RileyGiovanni Avila-FloresSarah RominskiGiovanni BarassiSarah ShuebGiovanni Battista ForleoSarah SpencerGiovanni Battista Levi SandriSarah Stewart-BrownGiovanni CangelosiSarah Susanne Lütke LanferGiovanni CannataSarah T. HenesGiovanni CenderelloSarah TaylorGiovanni ChiariniSarah VordenbergGiovanni Codacci-PisanelliSarah WheelerGiovanni DamianiSaral DesaiGiovanni FalsiniSaran LotfollahzadehGiovanni FanniSarassunta UcciGiovanni Freitas GomesSaraswathy NairGiovanni GabuttiSarat Kumar SahooGiovanni GerbinoSarath RajGiovanni GiganteSarath VijayakumarGiovanni GuggSarawut LapmaneeGiovanni IolasconSarbeswar PraharajGiovanni LaviolaSarbhan Singh Lakha SinghGiovanni MaccioniSardar S. ShareefGiovanni MarinelliSarhang HamaGiovanni MatareseSarika GuptaGiovanni MessinaSarina KajaniGiovanni N. RovielloSarit L. KaserzonGiovanni PeiraŠárka HubáčkováGiovanni PesŠárka KaňkováGiovanni PilloniSarkar M. Abe KawsarGiovanni PivaSarker AnkurGiovanni RalliSarminah SamadGiovanni RollaSarmiza Elena StancaGiovanni RostiSaroj AmarGiovanni SantiSaroj Kumar SahuGiovanni TesoriereSartaj Ahmad BhatGi-Ren LiuSarumathy SundararajanGiridhar BabuSarvath AliGirish BeedesseeSaša ĆukovićGirish UpretiSaša KostićĢirts BriģisSasa MissoniGisela Canedo-MarroquínSaša PrđunGisela ImmichSasa RajsicGisela Margarita Torres AcuñaSasan FaridiGisela Marta OliveiraSascha RuhleGisela Redondo-SamaSascha SchäubleGisela TeixeiraSasha M. ZeedykGisele CâmaraSasha ShepperdGisselle N. MedinaSashi DebnathGitima SharmaSashka KrumovaGiulia AbateSaška RoškarGiulia AmicucciSaskia WeberGiulia AtzoriSatarupa DasguptaGiulia BallardiniSatheesh NeelaGiulia BallarottoSathishkumar RamalingamGiulia BarbatiSathishkumar V EGiulia BivonaSatilmis BilginGiulia D’AurizioSatinder GillGiulia DonadelSatish VishwanathaiahGiulia FrissoSatomi KohyamaGiulia GiordanoSatoru KomatsuGiulia GuerriSatoru TsujiiGiulia IsettiSatoshi HayakawaGiulia LambertiSatoshi HirabayashiGiulia LausiSatoshi IreiGiulia MarroneSatoshi KawakamiGiulia MenculiniSatoshi MurakiGiulia PaganinSatoshi OsukaGiulia PaparellaSatoshi ShiraganeGiulia PaveseSatoshi UchidaGiulia PellegriSatoshi YamaguchiGiulia PozziSatoshi YuguchiGiulia PurpuraSättar EzzatiGiulia TorrominoSatu KalliolaGiulia TotiSatu LipiäinenGiulia VillaSatu NivalainenGiulia ZontaSatu PakarinenGiuliana DravaSaturnino Marco LupiGiuliana QuattroneSatwinder Kaur BainsGiuliana SolinasSatyanarayan RoutrayGiuliana VinciSatyapaul A. SinghGiuliana VitielloSatyavani KaliamurthiGiulio ArcangeliSaud AlrawailiGiulio ContemoriSaudade AndréGiulio CorazzaSaudade LopesGiulio Di MizioSaujanya KarkiGiulio Francesco RomitiSaúl Gómez-ManzoGiulio GabrieliSaúl Martín RodríguezGiulio SenesSaul Neves De JesusGiulio SeveriSaúl Oswaldo Lugo ReyesGiuseppe AcriSaulius ŠukysGiuseppe Alessandro ScardinaSaumik PanjaGiuseppe AmorusoSaumya GuptaGiuseppe AttanasiSaurabh PandeyGiuseppe BanfiSaurabh Suhas ThosarGiuseppe BarbatoSaurav DixitGiuseppe BarlettaSauren DasGiuseppe BattagliaSaverio CainiGiuseppe CaminitiSaverio MuscoliGiuseppe CoratellaSavina SchoenhoferGiuseppe CorradoSavvas LampridisGiuseppe CovielloSawako OkamotoGiuseppe De PalmaSawan JalnapurkarGiuseppe De PietroSawsan MohammedGiuseppe DefeudisSayed Ameenuddin IrfanGiuseppe DeleddaSayed Samed TalibiGiuseppe Di MartinoSayyed Ali SamadiGiuseppe FanettiSayyed Fawad Ali ShahGiuseppe GalzeranoSchmidt-Petri ChristophGiuseppe GulloSchroder SattarGiuseppe La TorreSchutte RudolphGiuseppe LanzaScot KimballGiuseppe LanzaroneScot RaabGiuseppe LiottaScott CoussensGiuseppe Lo GiudiceScott D. NashGiuseppe LoprencipeScott D. RozelleGiuseppe MaccagnanoScott DoigGiuseppe MancusoScott FlemingGiuseppe MarinoScott MazzettiGiuseppe MarraroScott McdonaldGiuseppe MasciaScott OgletreeGiuseppe MazzeoScott R. RichmondGiuseppe NassoScott SpreatGiuseppe NittiScott TalpeyGiuseppe PaceScott TeasdaleGiuseppe PelliccioniSean Chun Chang ChenGiuseppe PoleseSean ClarkGiuseppe PonziniSean D. TallmanGiuseppe PorroSean P. M. RiceGiuseppe RicciSean R. ScottGiuseppe RitellaSean SeamanGiuseppe RivaSean WilsonGiuseppe RussoSebastià Verger GelabertGiuseppe ScandurraSebastian BeckerGiuseppe ServilloSebastian BernatGiuseppe SignorielloSebastian BöttgerGiuseppe StabileSebastian Cǎlin VacGiuseppe SuariaSebastián Espoz-LazoGiuseppe UccelloSebastián Fierro-SueroGiuseppe VaroneSebastian FischerGiuseppe ZimmittiSebastian GłowińskiGiuseppina Lo MoroSebastián Gómez-LozanoGiuseppina SpanoSebastián Ignacio Espoz LazoGiusi Antonia TotoSebastian JäckleGiusi PerriSebastian KuhnGiusto TrevisanSebastian LüningGiusy Danila ValentiSebastian PrzybyłkoGiusy Rita CaponioSebastian RobledoGiusy TassoneSebastian RuinGizem AratSebastian SaniukGkikas GeorgiosSebastian SchadeGladys A. RamosSebastian SchloerGladys Barragan-JasonSebastian SchnaubeltGladys Merma-MolinaSebastian SkalskiGladys Onambele-PearsonSebastian SuggateGlauco ChisciSebastián Valverde-MartínezGleiston DiasSebastian WeirichGlen BatesSebastian ZartGlen CooperSebastian ZduńskiGlen NowakSebastiano CiccoGlen SegellSebastiano LavaGlenda Roberta Oliveira Naiff FerreiraSebastien BlanchetteGlenda TinneySébastien CambierGlenn E. WeisfeldSébastien CossinGlenn FinauSébastien LamyGlenn LedderSébastien UrbenGlenn Ole HellekjærSebastjan ŠkerličGlenn P. DorsamSebnem Ozturkoglu-BudakGlenn R. ValdezSecil AbayGlenn SheriffSeda YıldırımGlenn SinglemanSeffetullah KuldasGlenn YamakawaSegun OlatinwoGleyson Kleber Do Amaral-SilvaSehar IqbalGleyvis Coro-MontanetSehar Un Nisa HassanGloria Achempim-AnsongSehar ZulfiqarGloria GuidettiSeigo OhbaGloria Gutiérrez-VenegasSeiji ShibataGloria GutmanSejong BaeGloria I. Dávila-PulidoSelami Aykut TemizGloria Jiménez-MarínSelena FirminGloria LikupeSelena O’ConnellGloria Obuobi-DonkorSelene BaroneGloria PascualSelene MezzaliraGloria Pérez-RubioSelenia di FronsoGloria Reig-GarciaSelfa A. ChewGloria SantangeloSelim BozkurtGloria Santos-BeneitSelma Gouvêa De BarrosGlüge JulianeSelvakumar RadhakrishnanGniewko WięckiewiczSema K. AydedeGo AnanSema YurdakulGo InoueSemila Fenelly FernandesGo KanzakiSenatro Di LeoGo ShimadaSenem GünerGobbi PietroSenka Borovac ZekanGodfrey M. RwegereraŞenol TuranGodvin Sharmila VSenshuang ZhengGoichi HagiwaraSenthil AmudhanGokhan BacikSenthilnathan PalaniyandiGonca SoysalSenwei TsaiGonçalo C. RodriguesSeo Young KangGonçalo DiasSeok Shin TanGonçalo FernandesSeol A. KwonGonçalo MarquesSeonah LeeGonçalo SantinhaSeon-Cheol ParkGonzález Valencia Daniela GuadalupeSeong Hoon BaeGonzalo CarrascoSeong Man ParkGonzalo Díaz-MenesesSeong Nam HwangGonzalo Emiliano Aranda-AbreuSeongil LeeGonzalo García-RosSeongkwan ChoGonzalo Navarro-FernándezSeong-Kyu KangGonzalo R. Ríos-MuñozSeong-Nam NamGonzalo VarelaSeon-Rye KimGoo-Churl JeongSeoyoon HeoGoohyeon HongSepalika Swarnamali Baththana MudiyanselageGopal Nambi NambiSepideh HosseiniporghamGopal NathSepideh KhoshnevisGopal ShuklaSepideh OmidvariGopalakrishna AkurathiSerafí CambrayGoran AugustinSerafim M. OliveiraGoran ErfaniSe-Ran JunGoran GajskiSerdar SaritasGoran JaćimovićSerena CavalleroGoran KuvačićSerena DattolaGoran LivazovicSerena DonatiGoran TrbicSerena GrumiGorazd NovakSerena OliveriGorazd PojeSerena VeschiGordana PavlišaSerene TseGordana ŽauharSerge BrandGordon BarbaraSerge SchmitzGordon BinkhorstSergei M. ChernyákGordon HarperSergei SoldatenkoGorka Epelde UnanueSergej GričarGorka GallasteguiSergey DydykinGorka VuletićSergey KolesnikovGórnicki KrzysztofSergey MaksimovGottfried LemperleSergey MosolovGötz GelbrichSergey PirogovGour GoswamiSergey TarimaGoutam SinghSergey VasinGovind KrishnamoorthySergey ZhironkinGovind Sharma Shyam SunderSerghei CovantevGovinda R. PoudelSerghei SumanGowhar MerajSergi Garcia-RetortilloGowtham MunirajuSergii KrysenkoGoylette ChamiSergio A. Mota-RolimGözde Şengül AyçiçekSergio A. UsecheGrace Blest-HopleySérgio António LousadaGrace CullySérgio BrasilGrace E. ShearrerSergio Calonge-PascualGrace FarhatSergio ChicumbeGrace Felix GomezSergio Cinza-SanjurjoGrace IjomaSergio ContiGrace MaldarelliSergio Cuevas-CovarrubiasGrace SumSergio De SalvatoreGrace SzetoSergio Eduardo Contreras EspinozaGrace WongSergio FocardiGraciela Alatorre-CruzSergio FrumentoGraciela Caire-JuveraSergio Hernández SánchezGraciela Catalina Alatorre-CruzSergio Ibarra EspinosaGraciela Padilla-CastilloSergio Jiménez-RubioGrady RobertsSergio López-GarcíaGraeme NorvalSergio Luiz Stevan JuniorGraham A. MatthewsSergio MakrakisGrahame SmithSergio Martinez-VázquezGrant G. SchultzSérgio MatosGrant MurewanhemaSergio MazzoleniGrayson MorganSérgio MoroGrazia AntonacciSergio Ochoa JiménezGrazia CatalanoSergio SambataroGrazia D’OnofrioSergio Selles-PerezGrazia MaugeriSergio SilverioGrazia NapoliSergio Uribe-SierraGraziano MontaruliSergiu FendrihanGrażyna BączekSergiusz NawrockiGrażyna BartkowiakSergiusz PatelaGrażyna BudrynSerhat AsciGrażyna DykowskaSerhii NazarenkoGrażyna JanikowskaSerhiy NyankovskyyGrażyna Jarza̧bek-BieleckaSerik MeirmanovGrazyna LietzauSerkan PekçetinGrażyna Markiewicz-ŁoskotSerkan UsguGrazyna Nowak-StarzSerkan VarolGrażyna NowickaSerrano RitaGrażyna PutoSesan Abiodun AransiolaGrażyna RosaSe-Sergio BaldewGrażyna WejnerowskaSeth HoelscherGrażyna Wyszyńska-PawelecSeth JennyGreg BrowerSeth NorrholmGreg ElversSethupathi VelmuruganGreg HenrySetiawan PriatmokoGreger LindbergSetti IlariaGregorio Giménez EstebanSeul Ki LeeGregorio Martínez-SánchezSeulkee HeoGregorio VonoSeung Gu ShinGregory B. GoodrichSeung Joon BaekGregory BogdanisSeung Myung MoonGregory C. PrattSeung Uk LeeGregory GuthrieSeung-Geun LeeGregory HeathSeung-Gyu SimGregory L. BockSeung-ha LeeGregory M. RebchookSeungho JungGregory MoorlockSeunghoo LimGregory NeocleousSeunghwan LeeGregory S. ChingSeunghyun HwangGregory SimonsSeungman ChaGreta AdamczykSeung-min ParkGreta RiboliSeung-Nam KimGretchen MacySeungwon KwonGretha J. BoersmaSeung-Yoon RheeGrethe HyldigSevasti FilippidouGricelda Herrera-FrancoSevenpri CandraGrigorios G. DimasSeverin HornungGrigorios L. KyriakopoulosSevero Vázquez PrietoGrigoris AmoutziasSevgül DönmezGriselda LópezSeweryn LipińskiGrissell TiradoSeyed Abbas HosseiniGro Ellen MathisenSeyed Ahmad Naseri-AlaviGrzegorz BielecSeyed Ahmad Seyed AlinaghiGrzegorz BiloSeyed Alireza R. SalamiGrzegorz BlicharzSeyed Habibollah KaliliGrzegorz CieslarSeyed Meysam KhoshnavaGrzegorz DahlkeSeyed Mohammad MazloomiGrzegorz DrozdowskiSeyed Mohammad Nasir MousaviGrzegorz DziubanekSeyed Omid NabaviGrzegorz GałkoSeyed Vahid Razavi-TermehGrzegorz GrześkSeyed-Ali Sadegh-ZadehGrzegorz IgnatowskiSeyede Raheleh YousefiGrzegorz IlewiczSe-Youn JungGrzegorz JakielSezgin Çağlar AksezerGrzegorz JakubiakSha ZhuGrzegorz Józef NowickiShaaban Saad ElnesrGrzegorz KinelskiShabana BibiGrzegorz MaciejewskiShabana KhanGrzegorz MajewskiShabbir Syed-AbdulGrzegorz MentelShachar Timor-SchlevinGrzegorz MichalskiShadab ShahaliGrzegorz Nałęcz-JaweckiShadi BeshaiGrzegorz OleniaczShaghayegh BagheriGrzegorz OnikShagufta GaffarGrzegorz PaluszakShah FahadGrzegorz PituchaShah HussainGrzegorz PolokShah LimonGrzegorz Rza̧dkowskiShah RukhGrzegorz SchroederShahabaldin RezaniaGrzegorz SobotaShahabedin RahmatizadehGrzegorz StarobratShahar Lev-AriGrzegorz TrybekShahbaz KhanGrzegorz TrzcińskiShaher ZyoudGrzegorz WałowskiShahfahad ShahfahadGrzegorz Wilk-JakubowskiShahid Nawaz KhanGrzegorz WrzesińskiShahid UllahGrzegorz ZagułaShahira Abdel-RazekGrzegorz ZającShahista NisaGrzegorz ZielińskiShahla M. WunderlichGrzegorz ŻurekShahla WunderlichGu Eon KangShahriyar MukhtarovGuadalupe Hernández EscobedoShahrokh RahbariGuadalupe Molina TorresShahryar JafarinejadGuadalupe Rojas-SanchezShahzad AhmedGuadalupe Virginia Nevárez-MoorillónShahzad Zafar IqbalGuan LiShahzadah Nayyar JehanGuan Thye HueShaik Gouse PeeraGuang JiaShaik Vaseem AkramGuangda PengShaikh Abdullah Al RifatGuanghui JiangShaima AlothmanGuanglei HouShaimaa SelimGuangrong ShenShajahan FerosekhanGuangwei HuangShaji KachanathuGuangyang FangShakeel IjazGuangyang HouShalei SongGuanhua HouShalom StaubGuanqiu QiShambhabi ChatterjeeGuan-Ting PanShamik ChakrabortyGuenka PetrovaShamik ShahGugamsetty BalakrishnaiahShamik TiwariGuglielmo CampusShamika Ann MitchellGuglielmo DurantiShamir CassimGuglielmo ManentiShamshad KaratelaGuglielmo ManticaShamsudheen VellarikkalGuglielmo Niccolò PiozziShamsul Azahari Zainal BadariGuglielmo SaittoShamyla NawazishGuglielmo StabileShan JiaGui JinShan SuGui XuechenShancheng BaoGuido BelforteShandir RamlaganGuido BelliShane DixonGuido ErreygersShane DraperGuido Franco AmorettiShane EptingGuido MacchiarelliShane WarrenGuido MarsegliaShang SuGuido VanhamShang WuGuido VielShang-Jyh ChiouGuido WoesteShang-Pao YehGuilherme FurtadoShangyi GuGuilherme José Cunha GomesShani TiwariGuilherme Weiss FrecciaShaniko KaleciGuillaume GiraudShanina KnightonGuillaume Goyette-DesjardinsShankar BakkannavarGuillermina Guerrero MoraShankar Ganapathi ShanmugamGuillermo A. Cañadas-Dela FuenteShannon B. JuengstGuillermo Espinosa-ReyesShannon E. JarrottGuillermo Felipe López SánchezShannon Guillot-WrightGuillermo Gómez-DelgadoShannon GussGuillermo LaheraShannon M. RobsonGuillermo MoreirasShannon O’ConnorGuillermo Sanahuja-PerisShanquan ChenGuillermo Til-PerezShanshan CaiGuillermo Valencia-PalomoShan-Shin TonGui-ying CaoShanti SandaranGukdo ByunShantimoy KarGül DikeçShantkriti SrinivasanGulasekaran RajaguruShanton ChangGülçin ErmişShanyong WangGülendam KaradağShaoan WangGulistan Bahat-OzturkShaofei JinGulzar H. ShahShaofeng YanGulzhanat AimagambetovaShaohua ChenGun Hyung KimShaoni MukhopadhyayGundu RaoShaoning JiangGunilla BohlinShaonong DangGunn AasvangShaoquan LiuGunnar MauShaotao CaoGunnar ToftShaoxia PanGunnar W. SchadeShaoying MaGunter KreutzShaoyun PuGünther A. RezniczekShaozhuang MaGünther EiblSharad ChandGunther HempelSharada P. WastiGünther KoraimannSharafaldin Al-MusawiGunther SeckmeyerSharareh SharififarGuobin XiaSharifah Nurafidah Syed AnnuarGuocai TianShariq NajeebGuochun WuSharna MathieuGuodong ZhangSharon AndersonGuofeng ShenSharon BarakGuogui HuangSharon CoenGuohua HuangSharon E. BarrettGuojie MaSharon Elizabeth RossGuojun JiSharon H. MurffGuolei ZhouSharon L. LarsonGuoli DaiSharon MartinoGuoming LiuSharon O’HaraGuor-Cheng FangSharon StollGuosong ZhaoSharyl NassGuotian CaiShatha Ahmed AlduraywishGuowen ShangShauhuai FuGuoxiao SunShaun J. KiltyGuoya GanShaun PenningtonGuoyou QinShaun SabicoGupta SandeepShawky M. AboelhadidGupta VikasShawky MansourGurdal SahinShawn CarraherGürdal SahinShawn O’ConnorGurdeep SinghShawna L. McMillinGurjeet SinghShawnee WakemanGursharan K. SinghShay ShulerGursukhmandeep SidhuShayeb ShahariarGurvinder KaurShaza BishtiGustáv JablonskýShazia JamshedGustave MabiamaShazid Md. SharkerGustavious Paul WilliamsShazya KarmaliGustavo A. Ovando-MontejoSheeja Saji VargheseGustavo Adolfo MuñozSheela RaoGustavo AmorimSheena CarlisleGustavo Aviña CerecerSheena E. MarteniesGustavo CavagnariShehzad Abid KhanGustavo ChristofolettiSheikh Ahmad Izaddin Sheikh Mohd GhazaliGustavo De A. CoelhoSheila GahaganGustavo De Conti Teixeira CostaSheila GlennGustavo DesouzartSheila SeedGustavo Duarte PimentelSheila WeinmannGustavo Fabián MolinaSheilagh HodginsGustavo FernandesSheillah SimiyuGustavo FondevilaShekhar SeshadriGustavo MeschShelby PeelGustavo OlivaresSheldon MorrisGustavo PimentelShelia FleischhackerGustavo Rodríguez FuentesShelley GowerGustavo Vieira De OliveiraShelley L. HoldenGustavo ZuletaShellie AcocelloGuy HochmanShelly R. McFarlaneGuy HowardShelly RuartGuy RostokerShen YangGuyeol JeongSheng Dean LuoGuy-Lucien WhemboluaSheng FuGwénolé LoasSheng HoGyanesh Kumar TiwariSheng ZhangGyöngyvér SoósSheng ZhuGyorgy FeketeSheng-Cheng LinGyouhyung KyungShengfeng LuGytė DamulevičienėSheng-Hsiu HuangGyudo LeeShengjie LinGyula SzaboShengkun XieGyu-Un BaeSheng-Lih YehH. FritschSheng-Shun YangH. R. NagendraSheng-Wei WangHabil Pakai AnnamáriaShengwei ZhuHabyeong KangSheng-Wen TsengHadas Ben-EliSheng-Yu FanHadeel HalawehShenlong YangHadi FarhadiShenqi WangHadi KhoshabSheona MchaleHadi MoeinShepard HurwitzHadiar RahmanSheraz AliHadii M. MamuduSheree YorkHadith RastadSheri GersonHadiyanto HadiyantoSherif AbdelhamidHae Ran KimSherif SirajudeenHaeik ParkSherin SaheeraHaein LeeShernaaz CarelseHaeryun ChoSherouk FoudaHaeseong ParkSherri StastnyHaewon ByeonSherrie WallingtonHaeyoung LeeSherry EmeryHafeez RahmanSherry HambyHafiz Muhammad Uzair AyubSherry Yun WangHafiz Suliman MunawarShervin HashemiHafiz T.A. KhanSheryl HendriksHagai TapiroSheryl KopelHai Van PhamSherzod N. TashpulatovHaibo LiuSherzod TashpulatovHaibo YangSheung-Fat KoHaichuan WangSheyla M. Baptista SerraHaider I. AlyasariShi ChenHaider Mohammed ZwainShi MinHaider QasimShi ShenHaidong LuShiau Foon TeeHaifeng DengShiau-Chian JengHai-Hua ChuangShibili NuhmaniHaijian BingShiblu SarkerHaijie TongShichao KanHaijun WangShidrokh GoudarziHailang WangShigeharu SatoHailay GesesewShigehiro KarashimaHailing ZongShigeho TanakaHaimanti BiswasShigeki MatsubaraHaiming YanShigeo YamamuraHainan GongShigeru KimotoHaiqiang JiangShihai DengHairong MuShihai LiHairui LiuShih-An LiuHai-shan DangShi-Hao HuangHaishan ZengShih-Bin SuHaitao MaShih-Chieh ChenHaitao WangShih-Chih ChenHaitham JahramiShih-Chin LeeHaiwei JiangShih-Yi LinHaiwen LiShih-Yu LoHaixin PengShi-Jie CaoHaiyan JiaShijin WangHaiyan SunShijiu YinHaiyan WangShikha JainHaiyun ZengShikha SharmaHajime AndoShili GuoHajnalka FényesShiliang ShaoHak Jun SongShilong LiHakan MaydaShilpa BhandiHakim ManghwarShilpa TejpalHak-Seon KimShilpi AgrawalHala ShokrShilpi JainHale DemirtepeShima SaffarionpourHalil İbrahim BulguroğluShimin ZhuHalil İbrahim BurganShimrit Yaniv-SalemHalil İbrahim CeylanShin Jun ParkHalim İşseverShin Kue RyuHalina DoroszkiewiczShin MurakamiHalina Jędrzejowska-SzypułkaShinae AhnHalina KirylukShincheng YehHalina Mielicka-PawłowskaShing-Jye ChenHalina PodbielskaShingo HatakeyamaHalis SüleymanShingo YamamotoHallie ThomasShinichi ArakawaHalvard B. BönigShinichi EgawaHalyna KominkoShinichi SobueHamada ElwanShin-ichiro KarakiHamadjam AbboubakarShinji OtaniHamdi ChtourouShinji TeramotoHamdy AbdelkaderShinobu HasegawaHamdy ShaabanShinsuke MizutaniHamed GhiaieShintaro SatoHamed Haddad KashaniShintaro SengokuHamed MalekmohammadiShintaro SukegawaHamid BokharyShinwoo ChoiHamid MirzahosseinShinya IkedaHamid MoghaddasiShinya SuzukiHamid NoghanibehambariShirit Kamil-RosenbergHamideh BeigiShirley AlexanderHamidou KeitaShirley Ben ShlomoHamidreza Rabiei-DastjerdiShirley HillHamza El HadiShirley WyverHamzeh GhorbaniShirley Xin LiHan C. G. KemperShirli WernerHan LiShisong CaoHan Mou TsaiShiva MohajerniaHan Naung TunShi-wei GongHan ReichgeltShiyu LuoHan Shi Jocelyn ChewShizuo KatamotoHana BandouchovaShoaib AkhtarHana BrborovićShoaib FarooqHana Mahmutefendić LučinShobha Lata SinhaHana MorrisseyShobhika ParmarHana PadyšákováShofarul WustoniHanaa ShuwiekhShohreh GhasemiHanadi Y. HamadiShoji TakenakaHanbai ParkShokoofeh ShamsiHanbeom KimShooka MohammadiHande DemirShou-Chun ChiangHaneen B. AliShou-Jen LanHang ChengShoukui HeHang QuShova Thapa KarkiHang XingShravan GiradaHang YiShray SaxenaHang YuShreekar PantHang ZengShrilaxmi BagaliHanga GalfalvyShruthi SriramkumarHang-kin KongShruti KangaHani Amir AouissiShrutilipi BhattacharjeeHani MansourShu YangHani MorganShuai CaoHani Nasser AbdelhamidShuai LiHania Rahimi ArdabiliShuai MaoHanieh MohammadiShuai QianHanif QureshiShuaishuai LiHanifa BachouShuaiyi ShiHanisah KamilahShuang LiHanish JainShuanghong Jenny NiuHank WhiteShuanglin LiHankyung JunShuanning ZhengHanna BandarenkaShubha Ranjan DuttaHanna DudekShubham PathakHanna Górska-WarsewiczShuchi TripathiHanna JärveläinenShu-Ching MaHan-Na KimShu-Chu Sarrina LiHanna KustererShudong YuHanna LiberskaShuguang DengHanna Michniewicz-AnkiersztajnShuhei TsujiHanna NałęczShuhei YoshidaHanna Przepiera-BędzakShu-hen ChiangHanna SawickaShuhua MaHanna Tigerstrand GrevnertsShu-Hui WenHannah BadlandShu-Hui YehHannah CarverShu-I. WuHannah Cohen-ClineShujie YuanHannah H. CovertShu-Jui KuoHannah HeathShuko NojiriHannah Louise BradwellShula ShazmanHannah PazderkaShulamit GellerHannah TarverShulei ChengHanna-Kaarina JuppiShulei WangHannakaisa Niela-VilénShulin ShiHanne Christine BertramShu-lun MakHanno ErythropelShumin WangHanns de la Fuente-MellaShumit SahaHanns MoshammerShunfu HuHannu J. T. KorhonenShunichi HondaHannu LaurilaShunji YamadaHans BosmaShunli YuHans DietrichShunsuke KidaniHans DrexlerShunyao ZhuangHans HobbelenShuo LiHans RennekampffShuo YangHans SlabbekoornShuowei CaiHans TroppShuqiang WangHans Van EyghenShurong LuHans ZoellnerShutian ZhouHans-Christian SiebertShuva ChowdhuryHans-Georg ClassenShuyi XieHan-Shen ChenShuyuan HuangHans-Joachim Appell CoriolanoShweta GoreHans-Joachim LinckeShweta KukrejaHans-Ulrich BenderShweta YadavHanuman JatavShwu-Ru LiouHanuman Singh JatavShyam MoreHanvedes DaovisanShyam S. SamantaHanxiang LiShyi-Tien ChenHany Ezzat KhalilShyue-Cherng LiawHany H. ArabShyunji HayashiHany HassaninSibusiso MdletsheHany I. SakrSibylle ErmlerHany M. R. Abdel-LatifSichi LiHao ChenSiddhartha Bikram PandayHao ChengSiddhartha PatiHao DongSiddhita D. MhatreHao FangSidheswar RoutrayHao JiangSidor MonikaHao LiSie Long KekHao MaSiew Mooi ChingHao NieSigal Eilat-AdarHao PengSigal NaimHao ShenSigalit BlumerHao WangSigita LesinskienëHao WuSigmar SchnutenhausHao YuanSigne TomsoneHao ZhangSigrid Kusch-BrandtHaobijam BasantaSigrid LuhrHaobin FanSigrid TheunissenHaojun LiSigrid-Helene Kjørven HaugHaojun ZhangSigura MauriziaHaolan LiaoSigurður BrynjólfssonHaomiao LiSihyun KimHaomin LiSijia GuoHaoran ZhaoSijian LiHaorui WuSikandar I. MullaHaowen YanSikarwar Vineet SinghHaoyang LiSila KittiwachanaHaoyi WangSiliang YangHao-Yun KaoSilin HuangHaradhan KolyaSilin ZhangHaralabos Christos KarantonisSilivan Valentin MoldovanHarald BoehmSilke Anna Theresa WeberHarald JungnickelSilke GrabherrHarald KindermannSilke PawilsHarald KrentelSilva Thais GarciaHarald StummerSilvana Alves PereiraHarald VrankenSilvana Loana De Oliveira-SousaHarapan HarapanSilvana Mabel Nuñez-FaddaHardik AminSilvana Maria Russo WatsonHardy LimebackSilvana SecinaroHareef Ahmed KeerioSilvana StefaniHari KandelSilvano DragonieriHari Krishna NamballaSilvano Gabriele CellaHari Ponnamma RaniSilvano ZanusoHari Prasad DevkotaSilveri GiuliaHari Prasad SharmaSílvia A. SousaHariklia D. SkilodimouSílvia Almeida CardosoHarikumar RajaguruSilvia BaroniHaris AlibašićSilvia BecagliHaris MemisevicSilvia BonettaHarish ChanderSílvia CaldeiraHarkaitz ZubiriSilvia CanettoHarminder Pal SinghSilvia CapannaHarold EysterSilvia CarusoHarold HorellSilvia CasiniHarold I. ZeligerSilvia CataldiHarold J. FarberSilvia CerisolaHarold L. LazarSilvia ChersichHarold LauSilvia CirioHarri H. HakovirtaSilvia D’AgostinoHarriet BradleySilvia De SimoneHarriet ChurchillSilvia DíazHarriet OkronipaSilvia DragoniHarris Hyunsoo KimSilvia FerrariHarris Shah HamidSilvia FlorescuHarry CoccossisSilvia FornasaroHarry FriedmannSilvia Fraijo SingHarry MeyerSilvia García-BujalanceHarsh PriyaSilvia GiannattasioHarsha FowdarSilvia GonellaHarshad IngleSilvia Helenade Carvalho Sales PeresHarshit MahandraSílvia LopesHarshraj LeuvaSilvia M. Chavez-BarayHartmut MüllerSilvia Marcos-GarcíaHarto HakonenSilvia MascoloHaruka AbeSilvia MianoHaruka KatoSilvia MonacoHarukaze YatsugiSilvia MonteiroHaruki ShimazuSilvia Morales ChaineHarumasa YokotaSilvia Navarro-PradoHarun TanrıvermişSilvia NievaHarun UçakSilvia Ortiz BonninHaruo NakayamaSilvia Pellicer-OrtínHarvey BurnettSilvia PerzolliHarvey HouSilvia PiantoniHarvey L. SternsSilvia QuadroniHarvey S. JamesSilvia Regina Dias Medici SaldivaHarvey SarnatSilvia Reverté-VillarroyaHary RazafindralamboSilvia RiboHasan DarabiSílvia Rocha-RodriguesHasan ErogluSilvia SoléHasan HashemiSilvia SpivakovskyHasan MujtabaSilvia StaubliHasan ÖnderSilvia Ștefania MaicanHasan SardarSilvia SumedreaHasan SaygınSilvia SvegliatiHasan SymumSilvia ToricesHasanain Faisal GhaziSilvia Torres-PilesHasanul BannaSilvie RádlováHashem Salarzadeh JenatabadiSilvija ŠiljegHaslina TaibSilvio CristianoHasnan BaberSilvio De LucaHassan AlgadiSilvio IontaHassan ChoudrySilvio LorenzettiHassan El-RamadySilvio MaringhiniHassan Hossein-MohandSilvio NoceraHassan MajdoubiSilviu Daniel PredaHassan SaghiSilviu NateHassan TahirSilviu RogobeteHassan ZiadaSima HamadehHassanah HusinSima NaminHatice ÇıtakoğluŠime UkićHatice KumruSime VersicHaukur Freyr GylfasonSimeng LiHaya KornweitzSimeon KaitibieHayato SatoSimerjeet KaurHayden StewartSimge Taşar FarukHayder F. N. Al-ShukaSimin JiangHaywantee RamkissoonSimin PengHazel MarzettiŠimo ŠokčevićHazem S. KassemSimon AnnaheimHazim ElbakrySimon Bheki KhozaHazizi Abu SaadSimon ByrneHazrul Abdul HamidSimon CooksonHazuki TamadaSimon DublerHe LiSimon EvansHe LiuSimon HustonHe YinSimon John Christoph SoerensenHeather A. DavisSimon KilbaneHeather BrownSimon KizitoHeather BurksSimon P. PhilbinHeather D’AngeloSimon PorcherHeather E. DanyshSimon VrhovecHeather Gordish-DressmanSimon WeigmannHeather HowerSimona Andreea ApostuHeather J. A. FouldsSimona Cătălina ŞtefanHeather J. GothamSimona De StasioHeather J. WilliamsonSimona FerraroHeather KlemickSimona FortunatiHeather KrasnaSimona GavrilaşHeather KwokSimona GiordanoHeather L. BurrowsSimona GoiaHeather Leduc-PessahSimona KranjcHeather M. A. FentonSimona KutiHeather Nicole StoneSimona LattanziHeather StuartSimona ManciniHeather TilleweinSimona Mrakic-SpostaHeba A. EassaSimona PatarlageanuHebah M. S. Al UbeedSimona RaimoHebatalla NazmySimona SabbatiniHeber Arbildo-VegaSimona SagonaHéctor Beltrán-AlacreuSimona SpanuHéctor BurgosSimona TotafortiHector Eduardo RomanSimona VinereanHéctor García-LópezSimona ZaamiHéctor González De La TorreSimone BattagliaHéctor GutiérrezSimone BelliHéctor Hugo Pérez-VillarrealSimone BirocchiHector JorqueraSimone BoccalettiHéctor Pifarré ArolasSimone CammarasanaHéctor RalloSimone Charpentier MoraHector Riveros-RosasSimone CiacciHector TsangSimone CiaccioniHédi RomdhanaSimone CrouchHedva Vinarski-PeretzSimone De SioHedwin Kitdorlang DkharSimone Domenico AsprielloHee Il LeeSimone Garcia MacambiraHee Jin KimSimone GillHee Soon LeeSimone GrassiHee Sun KangSimone MorraHee Tae RohSimone MorselliHeeJin KimSimone PisanoHeejin LeeSimone RedaelliHeejung KimSimone SchenkmanHeena KumraSimone Valle De SouzaHeeran ChunSimone VarrasiHee-Sun KimSimonetta MorselliHeewon JungSimonetta VivianiHeeyoung LeeSimon-Pedro Izcara-PalaciosHefei ZhaoSina BorzooeiHege SletvoldSina DobaradaranHeidi HillmanSina HafiziHeidi Honegger RogersSina ShahabHeidi HungSinan DenizHeidi L. DempseySinan KardesHeidi OrtmeyerSing Kai LoHeidi ShukrallaSingara Singh KasanaHeidrun SturmSinghal RashiHeike ArgstatterSiniša BogdanHeike WieserSiniša MarkovHeikki RäisänenSiniša Mastelić IvićHeinrich WeberSiniša OpićHeinz BurtscherSiniša OzimecHeinz KleinöderSintu RongpipiHeitor Andrade JrSinziana SilișteanuHejun LiuSiobhan AaronHelber Enrique Balaguera LópezSiobhan CorcoranHelcio BlumSiobhan McAndrewHélder FerrazSiphiwe DlaminiHélder LopesSipho MdandaHélder Manuel Arsénio LopesSiqi CaiHelen AlmondSiqin WangHelen BanwellSiqing ChenHelen BranthwaiteSirajudheen AnwarHelen CollinsSiranjeevi NagarajHelen DickinsonSirish DharmapuriHelen FogartySiro CasoloHelen GriffithsSirpa KurppaHelen LittleSisira EdirippuligeHelen SchultzSisira Kumara Naradda GamageHelen WihongiSisitha JayasingheHelen YifterSitalakshmi VenkatramanHelena Akemi Wada WatanabeSithembile MabilaHelena BarrosoSiti Aisyah PanatikHelena Belchior RochaSiti Azrin Ab HamidHelena BiancuzziSiti BaidurahHelena C. MaltezouSi-Tong ChenHelena CatarinoSiushing ManHelena GapeyevaSiv StafsethHelena Hervius AsklingSiva PandaHelena JoséSivakumar ViswanathanHelena KisvetrováSivalingam NalliahHelena LoureiroSivalingam PeriyasamyHelena Maria Guerreiro JoséSivana Mirella AlibertiHelena MartynowiczSivarama Krishna LakkaboyanaHelena NavasSivasubramanian PalanisamyHelena NejezchlebováSiwarit PongsakornrungsilpHelena P. FelgueirasSiwatt PongpiachanHelena Reis Do ArcoSiwon JangHelena RibeiroSiwon LeeHelena Sandoval InsaustiSiyang WangHelena Sant’OvaiaSiyang YangHelena VilaçaSiyuan HeHelena Vila-SuárezSizulu MoyoHelene FachimSk Akhtar AhmadHélène KaneSk Mohiuddin ChoudhuryHélène Niculita-HirzelSkulska IrynaHelga WaapSky Koh Wei CheeHelge StalsbergSlađana MilardovićHeli LuSlaven JozićHeli SiltalaSlaven JurićHeli ToomanSlavica Blažeka KokorićHélia DiasSlavica KranticHélia JacintoSlavica TomićHelio Junji ShimozakoSlavka MroskovaHelio SalgadoSławomir Cezary ZmonarskiHellen GelbandSlawomir FranekHellen SantosSławomir GawrońskiHelmut FrohnhofenSlawomir JarekHelmut KüchenhoffSławomir KalinowskiHelmut MairSławomir KociraHelmut WenzelSławomir KoziełHelmuts AzacisSławomir KujawskiHéloïse LucaccioniSlawomir NeffeHelvi Heinonen-TanskiSławomir RębiszHem Dutt ShuklaSławomir S. NikielHemant KulkarniSławomir ŚlaskiHemant KumarSławomir SzymczykHemant Kumar SrivastavaSławomir TobisHend AbdelhakimSławomir ZapłataHendrik Gideon BrinkSławomir ZmonarskiHendrik Meyer-LueckelSławomira Drzymała-CzyżHenk Erik MeierSlobodan MarkovicHenk KoppelaarSlobodan RadusinovićHennie FisherSlobodanka Lemajic-KomazecHénock Blaise Nguendo-YongsiSmaranda BuduruHenri C. MollSmita MisraHenri TilgaSmita Nambiar-MannHenrieta HlisníkováSmriti NepalHenrieta HorníkováSneha RatnapriyaHenriette Löffler-StastkaSnehil DixitHenrik AspegrenSnehil GuptaHenrik GalboSnežana KaplanovićHenrik P. MinassiansSnežana KneževićHenrique CastroSnežana ŠtetićHenrique CerqueiraSnezana StrbacHenrique José CardosoSnežana Tomašević TodorovićHenrique NascimentoSnezhana Dineva SulovaHenrique NeivaSnezhanka OvcharovaHenrique PereiraSnježana Mardešić-BrakusHenry GoodfellowSnjezana PetrovicHenry InegbedionSo Jung MunHenry J. NussSo Young YooHenry McNabSoe MoeHenry MunyandukiSofia AugustoHenry SutantoSofia BarbosaHenryk KrawczykSofia Castanheira PaisHenryk MarszałekSofia Costa de OliveiraHenryk MizerekSofia Garcia-San JuánHen-yi JenSofia GomesHeping ShuSofia HadjileontiadouHera AntonopoulouSofia KoukouliHerbert E. AinamaniSofia LopesHerbert Júnior DiasSofia MariniHerbert LoellgenSofia MastrokoukouHerbert LöllgenSofia MeachamHerbert Ryan MariniSofia PugnaloniHeribert RamrothSofia RavaraHeriberto Antonio Pineda-EspejelSofia TaginiHerica Emilia Félix De CarvalhoSofia VeigaHêriş GolpîraSofia Villacis-NunezHeris HendrianaSofie SergeantHerlander Mata-LimaSofya YarusovaHerlin ChienSohail MumtazHerman A. Van WietmarschenSohail MushtaqHerman LutterodtSoham Al SnihHerman PeifferSoheil Abbaspour-RavasjaniHermona SoreqSoheil SalhaHernando ZuletaSohel M. JuloviHero P. WitSohsaku YamanouchiHeros Santos LoboSohtaro MimasakaHervé Laborde-CastérotSohye LeeHerve PlatelSohyun ParkHervé QuiquampoixSoibam Khogen SinghHervé ReychlerSoi-Hoi Michael LamHesham Ali El EnshasySok Kuan WongHesham HaffezSol García-GermánHetanshi NaikSola HanHeungsoon KimSolai Ramatchandirane PrabagaranHeung-youl YoumSolange MagalhaesHew MerrettSolange Parra-SotoHeying Jenny ZhanSolarski MaksymilianHezekiah FalolaSoledad BallesterosHid Felizardo Cordero-FrancoSoledad HerreraHidde BekhuisSoledad Romero RodríguezHideaki KawabataSolehuddin ShuibHideki HasunumaSoleiman AhmadyHideki KoizumiSoleiman BourrousHideki SekiyaSoleya DagnonHideo HashizumeSom DevHideo WadaSomasundaram SivachandiranHideoki FukuokaSomayeh FardindoostHideyuki KinoshitaSomya AggarwalHifzur SiddiqueSonal K. ThenganeHigashisaka KazumaSonam Sandeep DashHiginio González-GarcíaSonay AydınHikaru HoriSondavid NandanwarHilal Erkus OzturkSong JiangHilal OzcebeSong Soo LimHilario BecerrilSong YangHilary CauserSongli ZhuHilary CowieSong-Lih HuangHilary Izuchukwu OkagbueSongmiao FanHilary Piedrahita-ValdésSongül ÇakmakçıHillary K. KiprutoSongyan JiangHimanshu DeshwalSongyi KimHimender MakkerSongyi LeeHin-ngan Floria ChioSong-Young ParkHipolito NzwaloSonia BañuelosHiran ThabrewSonia Brito-CostaHiranya SritartSónia Cardoso PintassilgoHiro KishimotoSonia De Lucas SantosHiroaki FunahashiSónia Dias CoelhoHiroaki FurumaiSonia FantoneHiroaki KanouchiSonia García-SeguraHiroaki KawamuraSonia GondimHiroaki KijimaSonia GouveiaHiroaki KondoSonia Hernández CorderoHirokazu MadokoroSonia HornHirokazu ToyoshimaSonia J. Romero MartínezHiroki BabaSonia LippkeHiroki TamuraSonia Lozano-SepulvedaHiroko NakaokaSónia Maria Martins CaridadeHiroko OchiaiSónia Matilde Fonseca MateusHiroko OeSonia MorettiHiromasa TanakaSonia NathHiromichi YumotoSonia Regina PasianHironobu KatsuyamaSonia Rodríguez CanoHironobu TodaSónia SáHironori IshiguchiSonia Santander BallestínHironori YoshinoSonia ValHiroshi ChureiSonit BafnaHiroshi HoriuchiSonja Eriksson SteigenHiroshi IrisawaSonja HeintzHiroshi IshihataSonja JäckleHiroshi KinoshitaSönke LangnerHiroshi NonoguchiSonu M. M. BhaskarHiroshi YokomichiSonu SinghHiroshi YoshinoSonya MeyerHirotake MoriSoobin JangHiroto ItoSoohyun KimHiroya KuroyanagiSoo-Hyun SungHiroyuki HanadaSook Fan YapHiroyuki IshiyamaSook Jung KangHiroyuki NakamuraSookkyoung ParkHiroyuki WakiguchiSook-Ling ChuaHiroyuki YamadaSoon Singh BikarHiroyuki YamaguchiSoon Yew TangHisahide NishioSoon-Chan KwonHisao NakaiSoorej Jose PuthoopparambilHisashi MasudaSophia AnastasiouHisayo YokoyamaSophia CharitouHisayoshi OkamuraSophia HuyerHisham AlidrisiSophia LinHisham FansaSophia S. LeggettHitendra Singh SolankiSophia Tsong Huey ChewHitesh VijSophie AdamsHitoshi KondoSophie CarterHiu Chuen LokSophie Despina VasiliadisHlaváčková EvaSophie L. WardleHo Kee KoonSophie Lund RasmussenHo Man Homan LeungSophie YohaniHo Sun ShonSoraia F. NevesHo Yeon KimSoraj HongladaromHo Yin Eric LauSoram OhHoa VoSorana Maria BucurHoai TranSoraya Pacheco-da-CostaHoang-Long VoSoraya Paz MontelongoHocheol LeeSören MöllerHodur CeciliaSoren U. NielsenHojka Gregorič KumperščakSorin BurlacuHo-Jui TungSorin CaceHolger MuehlanSorin DanHollis KarolySorin Gabriel AntonHolly GaddSorina Grisaru-GranovskyHolly HollingsworthSorina Maria AurelianHon Lon TamSorinel CapusneanuHong BoSorout ShaliniHong Ching GohSoroya Julian McFarlaneHong FanSorush NiknamianHong LinSosei YamaguchiHong LingSossio Fabio GrazianoHong XuSotirios KakavasHong ZhaoSotirios TheofanisHong ZhengSotiris ApostolopoulosHongbin DengSotiroula LiasidouHongbin FangSou Hyun JangHongbing SunSoufiane BentoutHongbo LiSouha BougatefHongbo LiuSouhail HermassiHongbo ZhangSoukaina HilaliHongchang LiSouleymane DoucoureHongchang YangSoumi SuklaHongfang WangSoumitra DasHongfeng ZhangSoumyajyoti MajiHonghao ZhangSounak SahuHonghe LiuSoung-Yob RhiHong-In ChengSousan ValizadehHongjun FanSousana PapadopoulouHongli Sam GohSouvik PalHongliang ZhaoSowmiya A. MoorthieHongling SunSowmya PoosapatiHongmei YangSo-Yeon YoonHongmei ZhangSoyeong KimHongming ShanSo-Youn ParkHongming ZhuSpase ShumkaHongnan CaoŠpela KonjarHongping JinŠpela ŠalamonHongshik AhnŠpela SelakHongsun GuoSpela VerovsekHong-Tae KimSpencer BoyleHongwan LiSpencer JamesHongwei ChenSpencer SteinbergHongwei YangSperanza Claudia PanicoHongwu JingSphiwe Emmanuel MhlongoHongwu WangSpiros A. KostopoulosHongxiao LiuSpiros BezantakosHongyan ChaiSpórna TomaszHong-Yan LiuSpyridon KanellakisHongyan ZhangSpyridon PapageorgiouHongyi ZhouSpyros GalatsidasHongying PanSpyros KarakitsiosHong-Youl HaSpyros PapageorgiouHongyu RenSpyros TsitsigkosHonorata HowaniecSpyros VernardisHon-Yi ShiSravan PerlaHoon-Ki ParkSrđan D. KostićHor Yan Angel LaiSrdjan MarkovicHoracio Molina-SánchezSrdjan NikolovskiHorațiu CatalanoSrdjan SeremesicHorea George CrișanSrdjan VujicicHoria HaragusSrecko StopicHoria PăunescuSree Deepthi MuthukrishnanHortencia Silva-JímenezSree Lekshmi Sreekumaran NairHortense PetatSreekanth J. VarmaHortensia Moreno-MacíasSreekanth Kumar MallineniHosam A. SaadSreeram UdayanHosang AhnSreevatsan RaghavanHossain Mohammed Syedul HoqueSri Atmaja P. RosyidiHossein AfzalimehrSri Kumaran NadarajahHossein AzadiSri Ram PentakotaHossein ChavoshiSri TatminingsihHossein HamidifarSridhar PatraHossein Hossein-MohandSrijana KarkiHossein SadeghiSrijani BasuHossein SafariSrijithesh Rajendran SrijitheshHossein Shabanali FamiSrikanth UmakanthanHossein Tabatabaei-JafariSrinivas KolluruHossein TohidiSrinivas KoppuHossein ZareSrinivas NamuduriHossein ZarrinfarSrinivas NellimarlaHosung ShinSrinivas PasupuletiHou-Cheng YangSrinivas PittalaHouda El MustaphaSrinivasa ChandrasekarHourei OhSriram ValluriHoward ChenSrjit DasHoward F. PollickSsu-Han ChenHoward S. OsterStacey A. SantiHoward TakiffStacey Brown-AmilianHoward Thomas HurstStacey SloneHrayr AttarianStacy ClemesHridaya Raj DevkotaStacy M. PettigrewHridesh Kumar MishraStamatios GiannoukosHristo ChervenkovStamatios PapadakisHrvoje BudinčevićStamatoula TsikrikaHrvoje GrofelnikStanislas Thiriet-RupertHrvoje KarninčićStanislav FábryHrvoje MeaškiStanislav FerdovHrvoje TomićStanislav KlazarHsiangkuo YuanStanislav LazopuloHsiang-Yin ChenStanislav PekarskiyHsian-Yu WangStanislav YankovskyHsiao-Mei ChenStanislava IvanovaHsiao-Ping ChangStanislava StoyanovaHsiao-Ting TsengStanisław BaciorHsiao-Tung ChangStanisław GłazHsiao-Yu YangStanisław HorakHsien-Kuo ChangStanisław KaliszHsien-Wei TingStanisław KowalakHsien-Wen KuoStanislaw MaksymowiczHsi-Hsien YangStanisław ManulikHsin RauStanisław MlekoHsin-An ChangStanisław SzwedaHsin-Chi KoStanisław WanatHsin-Chieh WuStanisław WasilewskiHsin-Chih LaiStanislaw ZajaczkowskiHsingwei TaiStanislawa Bazan-SochaHsing-Yi ChangStanko RužičićHsing-Yuan LiuStankovska GordanaHsin-Ta HsuehStanley ChanHsin-Tang LinStanley Kam Ki LamHsin-Wei HsuStanley XuHsiu-Ching ChiuStanton A. GlantzHsuan-Chu ChenStarling EmeraldHsuanping ChangStasja DraismaHsuehhsing PanStav ShapiraHsueh-Ju LuStavros A. BelbasHsun-Yu ChanStavros CheristanidisHu LiuStavros KalafatisHu YuStavros KalogiannidisHua QinStavros PitoglouHua ZhangStavros PoulopoulosHuacheng XuStavros SindakisHuafeng TangStavros TryfonHuajun ChaiStavroula BarbounakiHuajun LiangStavroula I. BargiotaHuakang LiangStavroula IliaHuan HeStavroula TsitkanouHuan LiStefan Andrei NestianHuanbi YueStefan BushuvenHuan-Feng DuanStefan GebhardtHuan-Ming ChuangStefan HertlingHuanming WangStefan Herwig GödekeHuan-Wu ChenStefan KlebingatHuanzhou XuStefan Mandić-RajčevićHuapeng FengStefan MihaicutaHua-Tao WuStefan NaydenovHuaxian WanStefan NetschHuaxiong JiangStefan OlanderHubert DobrowolskiStefan S. IvanovicHubertus HimmerichStefan SchmidtHu-Chen LiuStefan ZirnHuei-Jane LeeStefania A. CilibertiHuei-Jhen WenStefania CantoreHuey-Hong HsiehStefania CerinoHuey-Jen SuStefania CeruttiHugh DevlinStefania CimbanassiHugh GashStefania CostiHugo Consciência SilvestreStefania Della VecchiaHugo CordeiroStefania FugazzaroHugo Heitor Moreira Enes FerreiraStefania MarsiliHugo José Nogueira Pedroza Dias MelloStefania MocciaHugo Leiria NevesStefania MoramarcoHugo López-RosasStefânia Ordovás De AlmeidaHugo LouroStefania OrrùHugo Machado SanchezStefania PaduanoHugo MurciaStefania PiazzaHugo OsórioStefania PilatiHugo SaldarriagaStefania ProiettiHugo SimkinStefania ScuriHugo Valadares SiqueiraStefania TamburriniHui MaoStefania ToselliHui Shan LeeStefania TroiseHui XiaoyanStefanie GrataleHui YueStefanie HarschHui ZhangStefanie HelmerHui ZhuStefanie KleyHuibin ZouStefanie L. RussellHui-Chi LiStefanie MaurerHuicong JiaStefanie P. GlaeserHui-Hsun ChiangStefanie PlageHui-Hua HsiaoStefanie SamietzHuijun ChihStefano AmatoriHuiming HuangStefano CanduraHui-Ming LiuStefano CasalottiHuiming ZongStefano CirilloHuiquan WuStefano CozziHui-Ting YangStefano DastoliHuitzilihuitl Saucedo-OrozcoStefano EramoHuixiang ZengStefano FerracutiHui-Ying LukStefano FerrettiHuiying LuoStefano FilacordaHuiying QiStefano FranceschiniHuiyu ZhouStefano Maria IacusHuizhen ZhangStefano MartinaHuizhou YangStefano MasieroHulisani MatakanyeStefano NecozioneHulya TorunStefano NobileHumaira SeemaStefano PaganoHumood NaserStefano PalermiHung King TiongStefano PassanisiHung Vo ThanhStefano PinardiHung-Chang LiaoStefano PorruHung-Lung LinStefano QuartaHung-Pin HuangStefano RamatHung-Pin TuStefano RestainoHung-Vu TranStefano RizzaHung-Wei WangStefano RossiHung-Yi ChuangStefano RuggieriHung-Yin LinStefano SapienzaHung-Yu HuangStefano SaranHunter BennettStefano ScaranoHunter J. BennettStefano SilvestriHunter WaldmanStefano TambuzziHuong Nguyen ThuStefano TardivoHusam Jasim MohammedStefano TasselliHusam QanashStefano TavanoHusarova DanielaStefano TribertiHüseyin Bariş TecimenStefano TuriHüseyin Onur TezcanStefano VarrellaHusin AlatasStefano Zauli-SajaniHusnul Azan TajarudinStefanos DailianisHussein BasmaStefanos PlexousakisHuthaifa ObeidatStefanos TsiarasHuy Duc DangStefanos TsigdinosHuynh Vuong Thu MinhSteffany ChamutHuza GheorgheSteffen FlessaHwai-Ting LinŠtefica MikšićHyam Nazmy Bader KhalafŠtefica MrveljHyang Yuol LeeStefinee PinnegarHyang-In Cho ChungStefka FidanovaHye Chang RhimStelian DimitrovHye Eun LeeSteliana RodinoHyegyeong ChaStelios XanthosHyejin JeonStelios ZimerasHyejin JungStella GiannakopoulouHyemin HanStella LopezHyeon-Cheol KimStella María Fuensalida NovoHyeongsu KimStella Maris MacínHyeonseok KimStella SekulićHyeran AnSten HellströmHye-Ryoung KimStepan FeduniwHyeryun KimŠtěpán KavanHyo Sun JungStephan AltmayerHyo Young LeeStephan BischofHyo-Geun ChoiStephan HeisingerHyo-Jeong HwangStephan HülsmannHyojong SongStephan J. ReshkinHyo-jung JeongStephan K. SchwanderHyokeun LeeStephan LauxmannHyong Nyun KimStephan PayrHyong-Jin HongStephan ScherneckHyo-Sun KwakStephan SchmidHyoung Chul ShinStephan StoebeHyoung Suk LeeStephan Van Der ZwaardHyuk-Jae RohStephan WalchHyuk-Yoon KwonStéphane P. AhernHyun ByunStéphane SanchezHyun Jin LeeStephania A. CormierHyun Ju ChongStephanie A. SmithHyun K. KimStephanie AtkinsonHyun Min OhStephanie C. FieldHyun Young KooStéphanie CaëtHyun-Duck KimStephanie DauthHyung Rok WooStephanie DevignotHyung-Il KimStephanie DuguezHyungoo KangStephanie FosterHyun-Hee HeoStephanie G. B. SullivanHyunjin ChaStéphanie G. GentileHyun-jin MinStephanie Glass ClarkHyunjoo KarlssonStephanie HoltHyunjung KimStephanie Howard WilsherHyun-Su YounStephanie HunterHyun-Woo LeeStephanie J. SteeleHyun-Young KimStéphanie Lefebvre‪I. Gusti Ngurah Edi PutraStephanie M. HareI. ImeldaStephanie MailletI. Ting ChuangStephanie MaloneIain A. BrownleeStephanie PyneIain J. CruickshankStephanie Rodríguez BesteiroIain RobertsonStephanie SteeleIain ScottStephanie VallianatosIan BoggeroStephanie Wing LeeIan CulpanStephanus Petrus du PreezIan CumminsStephany DudaIan David BullerStephen AmbroseIan G. DuncanStephen B. LevineIan HodgsonStephen BagwellIan JohnsonStephen BokIan JonesStephen CardenIan KuijtStephen CarterIan McLeanStephen ChristopherIan NewhouseStephen ClaytonIan RotherhamStephen CornishIana MarkevychStephen CrookeIanik PlanteStephen D. EdwardsIb JammerStephen DrinkwaterÍbis A. P. MoraesStephen E. ForemanIbolya FülöpStephen E. HamptonIbomoiye Domor MienyeStephen FavaIbraheem KarayeStephen HollandIbrahim AgwaStephen JacobsIbrahim AlpStephen JenningsIbrahim AlsohaimiStephen John HoughtonIbrahim ArpaciStephen JonesIbrahim ElbatalStephen M. PopkinIbrahim ElshaerStephen McKinneyIbrahim ElsohabyStephen NewmanIbrahim IssifuStephen P. RollIbrahim M. SayedStephen R. ShamblenIbrahim MoslyStephen RoyleIbrahima SallStephen Siu Yu LauIbraín Enrique Corrales-ReyesStephen Taiwo OnifadeIbukun OluwoyeStephen TaylorI-Chen TsaiStephen TsuiI-Chien WuStephen VogtIchiro YamadaStephen WiryaIcíar ArteagoitiaStephen YoolIciar Pablo-LerchundiStergios BoussiosIda AringerSteve BeermanIda Bagus Ilham MalikSteve GimbelIda CariatiSteve GlassIda G. MonfaredSteve GoodmanIda PastoreSteve HemingwayIda PetrovičováSteve JefferyIda Rosnita IsmailSteve JexIdayu Badilla IdrisSteve MannIdiano D’AdamoSteve Simpson-YapIeva StundienėSteve SussmanI-Fei ChenSteve T. RussellIffat ElbaraziSteven A. KolmesIffath SyedSteven BaumruckerIftikhar AhmedSteven BrowningIftikhar Ahmed ChannaSteven D. ShirkIgal ShohetSteven DarbyIgnacio BartoloméSteven ForrestIgnacio Díez LópezSteven G. KovenIgnacio Diez-VegaSteven JacobsIgnacio HernandezSteven KongIgnacio MacphersonSteven Kwang San TanIgnacio Martínez González-MoroSteven LohIgnacio MontorioSteven LucasIgnacio Novo-VeleiroSteven McMichaelIgnacio O. RomeroSteven PresleyIgnacio Ramos-VidalSteven PudneyIgnacio Refoyo RomanSteven PueppkeIgnasi CasanovaSteven R. ApplewhiteIgnasi Navarro-SoriaSteven Ross MurrayIgnatius Ryan AdriawanSteven SheldonIgnazio BlancoSteven SilversteinIgnazio CondelloSteven ThygersonIgnazio Gaspare VetranoStig HellebustIgnazio La MantiaStig MolstedIgor AgranovskiStina EngelheartIgor BetkierStine Eriksen HammerIgor Cavallini JohansenStöcker Phil. NicolaIgor CelikovicStormi Pulver WhiteIgor CostaStuart C. PainterIgor DiasStuart KimIgor FischerStuart LeskeIgor G. ZenkevichStuart LinleyIgor GallayStuart MarcovitchIgor GrabovacStuart McCluskeyIgor GruicStuart ReeceIgor JerkovićStuti KokkaleraIgor P. KaidashevStyliani GeronikolouIgor Piotr TurkiewiczStylianos E. TrevlakisIgor PušnikStylianos PapazisIgor RyabovSu Keng TanIgor S. LitvinchevSu ZhangIgor SmojverSuarez Quintanilla Juan AntonioIgor Y. IskusnykhSubhabrata MoitraIgor ZurbenkoSubham MukherjeeIhab H. SawalhaSubhas KhajanchiI-Hsuan LinSubhashis DasI-Hua ChenSubhasish DasIjung KimSubhradip KarmakarIkbal Agah InceSubin Thenkunnel SurendranIker MadinabeitiaSubodh Chandra PalIker SaezSu-Boon YongIk-Hwan HanSubrata DebIkhwan RinaldiSubuhi KaulIkkei TamadaSuchada KongkiatkamonIkpe Justice AkpanSucharu GhoshIkram AliSuchata KirdponpattaraIksoo HuhSudarshan BhattacharjeeIkuya MiyamotoSudeshna GhoshIlaha IsaliSudha BhavanamIlan KedanSudhakar VundavalliIlana HaliwaSudhanya BanerjeeIlaria BenedettiSudhir K. RajpootIlaria CataldoSudhir K. ThakurIlaria ChiccaSudhir KotnalaIlaria DoimoSudhir Kumar PaidesettyIlaria FantasiaSudhir Kumar PandeyIlaria FerrariSudip DuttaIlaria FuocoSudip ShethIlaria GuerrieroSudipta BiswasIlaria PinaSudipta RoyIlaria RiboldiSue BuckleyIlaria RodellaSue DevineIlaria Tocco TussardiSue HolttumIldikó JócsákSue JordanIldikó KovácsSue L. HallIleana C. FarcasanuSueli Coelho Da Silva CarneiroIleana PetrariuSueli M. GomesIleana RadulescuSugantha Priya ElayapillaiIlenia RuggiuSugato HajraIlia NadareishviliSugy ChoiIliana FerrerSuha JaradatIliana Medina-RamírezSuhail Sarwar SiddiquiIlias KavourasSuhaila Mahmoud SmailiIlias MahmudSuhaily Mohd HaironIlias MavroidisSuhas Sampat KharatIlias N. LymperopoulosSuh-Hee ChoiIlias TsiakasSu-hie TingIlias VlachosSuilan ZhengI-Lin WangSuin ParkIlir ShehuSuisui HaoIliya GutinSujan GautamIlke Coskun BenlidayiSujarwoto Sujarwotoİlker DereSujatha RamaniIlkka ArminenSu-Jin Jungİlknur KaleliSujin ShinIlma MufidahSujit DasIlmari MäättänenSujita Kumar KarIlona GórnaSujita NarayanIlona PokoraSu-Jung LiaoIlona Rubi-FessenSukanya SereenonchaiIlse BlignaultSukeun JeongIlse SchrittesserSukkyung YouI-Lun ChenŞükrü MereyIlya M. MukovozovSukru Serdar BalciIl-Youp KwakSukwon KimIlze BeitaneSulaiman LakohIlze ŠtrumfaSulaymon EshkabilovIm Hee ShinSuleman LazarusIm SunSüleyman EkenImad SalehSu-Lien LuIman RadSulimon SattariIman Salehi HikoueiSuling HuangIman TavassolySultan Ayaz AlkayaImelu G. MordenoSultan Ayesh Mohammed SaghirImlak ShaikhSultan Daud KhanImmacolata Di NapoliSultan KavImran AliSumaiya ThaseenImran AnwarSumali PandeyImti ChoonaraSuman SamantrayImtiaz Afzal KhanSuman WattalImtiaz HussainSumate ChaiprapatIn Seo LaSumeer SinghIna KayserSumei LiIna Manuela SchülerSu-Mei XiaoIñaki De La PeñaSumera NazIñaki ElíoSumesh ParatInaki IzquierdoSumio AkifusaInaki Pastor-PonsSumira MalikIndira Deepthi Gamage KitulwatteSumitaka KobayashiIndri Hapsari SusilowatiSumitha Rajendra RaoIndrikis KramsSun YanInês Carvalho RelvaSun-Ae KimInés Casado-VerdejoSuncana Simonic-KocijanInês Casquilho-MartinsSundararajan JayaramanInes DrenjančevićSunday OnagbiyeInês FranciscoSunday SimboInes HrdaloSunee BovonsunthonchaiInes KožuhSuneel KumarInés Llamas-RamosSung Ho HwangInês PaciênciaSung Jae KimInes TestoniSung ParkInes Veiga PereiraSungbae MoonInese StarsSungchul MunIneta KristovskaSunghoon ParkInga Griskova-BulanovaSunghoon ShinInga KonstantinaviciuteSunghwun KangInga MatulytėSunghyup Sean HyunInga V. SlesarenkoSungjo HongInga ZinicovscaiaSungjun JungIngalill HallbergSungkyu LeeInge WernerSung-Kyung KimIngeborg LundSung-Lang ChenInger KullSungok ChangIngi EinarssonSungsig BangIng-Jer HuangSung-Soo KimIngrid Del Valle García CarreñoSung-Ta LiuIngrid EngdahlSungwon LeeIngrid FalnogaSungwook KimIngrid HausserSung-Woon OnIngrid HellströmSungyong ChoiIngrid KalteneggerSunhee ParkIngrid MoonsSunil KarhadkarIngrid TonniSunil Kumar GuptaInguna GriškevicaSunil Kumar SansaniwalIngunn FjørtoftSunil NaikIngyu MoonSunil RaiIn-Hee LeeSunil S. KarhadkarInhong SongSunil SinghInigo Navarro-FernandezSunil SirohiInigo SoterasSunita GhoshInken HöllerSunith Babu MadduriInmaculada Aguiar-DíazSun-Kyug KangInmaculada C. Palomo-ToucedoSunkyung ChoiInmaculada Corral LiriaSunwoo LeeInmaculada García MartinezSunyong EomInmaculada LizasoainSun-Young KimInmaculada López-IzquierdoSun-young ParkInmaculada Martínez PérezSuparat PhuanukoonnonInmaculada MateoSuparerk JanjarasjittInmaculada Mateo-RodríguezSuphanut JamonnakInmaculada Rodriguez CunillSuprava ChakrabortyInmaculada YustresSupriya GuptaInna KhovrakSupriya JaggaInna P. SolyanikovaSupriyo RayInna PetrashevichSurachai WongchareeInna SekirovSurachet ImlimthanInna SimakovaSurajit BasakInna V. MelnykSurendiran BalasubramanianInnocent MusondaSurendra RajpurohitInon ZuckermanSuresh Chandra SubediInsung ParkSuresh NarayanasamyInta Dimante-DeimantovičaSurinder SinghIntan Hashimah Mohd HashimSururah A. BelloInyong KimSurya KantIoan AschileanSurya PaudelIoan HutuSurya Prakasarao KovvasuIoan I. ArdeleanSusan AstleyIoan MilosanSusan BuchananIoana Bianca ChițuSusan BuellIoana BoerasSusan Chang SuIoana DemetrescuSusan ChristoIoana DulamaSusan FlynnIoana Madalina MoldovanSusan HandyIoana MarinSusan J. JenkinsIoana MartuSusan JansonIoana MozosSusan K. BurkeIoana SilistraruSusan LetvakIoana StanculescuSusan McClementIoana StreațăSusan P. BrattonIoana SurSusan ParhamIoana-Crina Pop-CohuţSusan PattersonIoana-Laura ȚibulcăSusan RacetteIoana-Mihaiela CiucaSusan RamloIoanina ParlatescuSusan RazvanIoanna Anna PapachristouSusan RifkinIoanna KyriakouSusan RobertsonIoanna PalaiologouSusan ScottIoannis A. BarboutisSusan SissonIoannis A. KalogiannidisSusan Snow WadleyIoannis AnagnostopoulosSusan StraussIoannis G. AviziotisSusan Van HoorenIoannis GeorgakopoulosSusan WakenshawIoannis KampourisSusan WhitingIoannis KansizoglouSusan WitteIoannis KosmasSusan WoskieIoannis KostakisSusan YatesIoannis KostoulasSusan YeeIoannis LiampasSusan Yee ChowIoannis LogothetisSusana Adelina Moreira Carvalho BastosIoannis M. AslanidesSusana BorgesIoannis PaliokasSusana CoimbraIoannis PetrakisSusana CostaIoannis TsakiridisSusana G. Paíno QuesadaIoannis VardopoulosSusana HenriquesIoannis VentoulisSusana HormigosIoannis ZabetakisSusana I. Justo-HenriquesIolanda BorzìSusana LázaroIolanda De Fátima Lopes Calvo TibérioSusana MiyahiraIolanda PopaSusana MourãoIon PopaSusana Navalpotro PascualIon SanduSusana NetoIon SavaSusana PaixãoIonela ManiuSusana PastorIonela Ruxandra SfeatcuSusana PedrasIonescu Tiberiu-ConstantinSusana Pulgar MunozIonut BanuSusana SeidmannIonuţ Daniel GeoneaSusana SilvaIonut Laurentiu PetreSusana Sofia MiguelIonut LuchianSusanna ChuiIonuţ MineaSusanne FrennertIonuț NicaSusanne G. R. KlotzIonut SchiopuSusanne K. WocheIonut TudoranceaSusanne M. Van Der VeenIoseb KhelashviliSusanne MoebusIosif BotetzagiasSusanne RankIosif SifakakisSusanne SchmidtIosif Vasile NemoianuSusanne StickelIppolito NotarnicolaSushil DubeyIqbal AkhterSushil K. UpadhyayIqram HussainSushobhana BandyopadhyayIqrash ShafiqSusi Ari KristinaIra HelslootSusie YoonIra-Adeline SimionovSusmita BarmanIram FatimaSusmita GuptaIram MurtazaSusmita LahiriIrena Baranowska-BosiackaSusrutha Babu SukhavasiIrena BatesSusumu ItoIrena Brčić KaračonjiSusumu MitsuyamaIrena FigurskaSusumu YamadaIrena GlažarSutanuka BhattacharjyaIrena IlicSutham NanthamongkolchaiIrena KeserSuvi NenonenIrena M. HlaváčováSuvi RaviIrena MilaniakSuyanto SuyantoIrena Niedźwiecka-FilipiakSu-Ying TsaiIrena Pandža BajsSuzan CinarIrena PawłyszynSuzan HamidIrena Raguž KrištićSuzana BrownIrena Smaga-MaślankaSuzana DemyenIrena TušerSuzana GavrilovicIrena Zupanič PajničSuzana KonjevodaIrene AlbarránSuzanne Adele TraskIrene CampiSuzanne C. LiIrene Carrillo MurciaSuzanne FitzsimmonsIrene CasiqueSuzanne GilbeyIrene CecchiSuzanne GrossmanIrene Claudia ViscontiSuzanne HagenIrene Cortés-PérezSuzanne HeldIrene DaskalopoulouSuzanne Hoi Shan LoIrene Gomes Câmara CamachoSuzanne L. CrossIrene MargaritisSuzanne LeaIrene OliveiraSuzanne M. SimkovichIrene OttavianiSuzanne O’NealIrene P. CarvalhoSuzanne R. KunkelIrene Polo-BlancoSuzanne R. SteinbaumIrene RodriguesSuzanne WenzelIrene Sandoval HernándezSuzanne WilsonIrene ScalaSuzy WeemsIrene ScavelloSven BarthIrene SchiavettiSven DreyerIrene TagliaroSven F. GarbadeIrene TilikidouSven FlemmingIrene Torres-SanchezSven MeisterIreneusz GrubeckiSvenja Dannewitz ProssedaIreneusz NowogońskiSvetlana EdenIresh JayawardenaSvetlana K. PerovicIrfan A. RatherSvetlana KrasnovaIrfan KhawajaSvetlana NepocatychIrfan MohamadSvetlana SharonovaIrfan SaleemSvetlana SimićIrfan Ullah KhanSvetlana Sokolov MladenovicIrida TsevreniSvetlana UščaIride Francesca CeresaSvetlana V. GusakovaIrimia Mollinedo-CardaldaSvetlana VukotićIrina Adriana ChiurciuSvetozár DluholuckýIrina Albastroiu NastaseSviatlana MarozavaIrina AndrievskayaSvitlana BilanIrina B. AlievaSwaantje CasjensIrina BilanSwaminathan P. IyerIrina Eduardovna SokolovskayaSwanand KoliIrina FeyginaSwapna YellankiIrina G. BelyaevaSwapnil Ganesh SanmukhIrina GradinaruSwatantra DubeyIrina IelciuSwati JaiswalIrina Isakova-SivakSwati SachanIrina KarpovichSwee KuikIrina KaygorodovaSwen M. JohnIrina KlizienėSwetha RamadesikanIrina Magdalena DumitruSwinburne AugustineIrina MakarovaSyafrizal Helmi SitumorangIrina MateiSyazwani IdrusIrina NaletovaSyed Ali HussainIrina Nicoleta ZetuSyed Awais Ali Shah BokhariIrina NovikovaSyed Aziz Ur RehmanIrina OntelSyed Ehtisham-ul-HaqueIrina RabinovichSyed Hassan RazaIrina SeverinSyed Husain Mustafa RizviIrina V. KiselevaSyed Khairul BasharIrina YakhneevaSyed Mohammed BasheeruddinIrina-Iuliana CostacheSyed NabilIrineu De Brito JuniorSyed NurulainIris BergerSyed RazaIris FeinbergSyed Saad Azher AliIris Juárez LealSyed Sarosh MahdiIris MamierSyed Sayeed AhmadIris PlioniSyed Shamsul HassanIris UdasinSyeda ShehzadiIris Xiaoxue YinSylvain IcetaIris XuSylvain QuiédevilleIrma EloffSylvère StörmannIrma TchokhonelidzeSylvester OmoruyiIrmina Ćwieląg-PiaseckaSylvester Reuben OkekeIrmina MorawskaSylvia KirchengastIrmina RostekSylvia MignonIrshad AhmadSylvia MooreIrvan LuhungSylvia OforiIryna BashynskaSylvia TwerskyIsa Abdullahi BabaSylwester KłyszIsaac AmoahSylwester KozakIsaac EstevanSylwia Agata BęczkowskaIsaac GagnéSylwia AndrzejczukIsaac González-SantoyoSylwia BudzynskaIsaac Levi HendersonSylwia Dzięgielewska-GęsiakIsaac LifshitzSylwia GwoździewiczIsaac López-LavalSylwia HuszlaIsaac Machorro-CanoSylwia JaskulskaIsaac NooniSylwia Katarzyna KałuckaIsaac Oyeyemi OlayodeSylwia ŁaganIsaac P. DapkinsSylwia Łęgowik-ŚwiącikIsaac RampediSylwia Pieńkowska-KamienieckaIsaac Uzoma AsuzuSylwia PłaczkowskaIsabel Alvarado-CruzSylwia RzeszotekIsabel BalteiroSylwia WesołowskaIsabel Cabrita CondessaSylwia Wieder-HuszlaIsabel CansadoSylwia Żakowska-BiemansIsabel CarmoSyoichi TashiroIsabel Corrales-GutiérrezSyrine HizemIsabel del-Arco BravoSyuichi ItahashiIsabel Diéguez CastrillónSyu-Ruei JhangIsabel EscobarSzabolcs DulebaIsabel Escobio-PrietoSzabolcs FogarasiIsabel FernandesSzabolcs KériIsabel FernandezSzabolcs KertèszIsabel GomezSzabolcs SzatmariIsabel Iborra-MarmolejoSzczepan PiszczatowskiIsabel Lopez-CoboSzczepan WiechaIsabel LucasSze Yan LiuIsabel M. Herrera SánchezSzenderák JánosIsabel María Gómez-TriguerosSzergej VinogradovIsabel Martinho Da SilvaSzilvia FiatalIsabel Mercader-RubioSzilvia FodorIsabel Mourão-CarvalhalSzmodis MártaIsabel Pardo GarcíaSzu-chieh ChenIsabel Pérez HernándezSzymon BudrejkoIsabel PiñeiroSzymon CyfertIsabel ProençaSzymon PlewikIsabel ReisSzymon RozanskiIsabel Rodrigo MartínSzymon SkoczyńskiIsabel RodriguesSzymon SzemikIsabel Rodríguez CostaSzymon ZylinskiIsabel RubioT. G. Nilmini ChandrasenaIsabel SalvatT. J. HollingsworthIsabel Ten-DoménechTabitha M. BenneyIsabel TraperoTabito KinoIsabella AquilaTadas ŽvirblisIsabella BonacciTadashi ItoIsabella F. S. NgTadashi YagiIsabella NeriTadayuki IidaIsabella ZanellaTadeusz A. GrzeszczykIsabelle AlbertTadeusz AmbrozyIsabelle BonmarinTadeusz KufelIsabelle Brun-HeathTadeusz MalewskiIsabelle CabyTadeusz MoskalikIsabelle DemersTadeusz OlkuskiIsabelle DenisTadeusz PaszkoIsabelle Durand-ZaleskiTadeusz PrzybyłowskiIsabelle GermainTadeusz SiwiecIsabelle LoganTadgh McMahonIsabelle SkinnerTae Ha ChungIsabelle ThaonTae Im KimIsac Florin-LucianTae Jin ChoIsaías García RodríguezTae Keun YooIsaias Scalabrin BianchiTae-ahn KangIshak PacalTae-Hwan KimI-Shiang TzengTae-Ju ChoIshikawa NoboruTae-Jung KimIshita BhaktaTaekeun OhIsidora VujcicTae-lim YoonIsidoro FasolinoTaesu CheongIsidro A. PérezTaewoo NamIsidro DiegoTaeyoung KimIsidro Juan MironTafadzwa DzinamariraIsilda RibeiroTafireyi MarukutiraIskra CvetkovikjTaha A. El-SayedIslam Hamdy ElghonaimyTaha Koray SahinIslam HusainTaha MehanyIslam IbrahimTahereh MaghsoudiIslam MohamedTahir NooraniIsmael Cristofer BaierleTahissa Frota CavalcanteIsmael Fuente MerinoTahna PettmanIsmael S. Sánchez-HerreraTaichi TenkumoIsmael Sánchez-GomarTai-Jin SongIsmail AlbayrakTaiki MoriIsmail AlkhatibTai-long PanIsmail Ercument AyazliTaina LajunenIsmail LuharTainya ClarkeIsmail MondalTaira MiyasakaIsmail OdetokunTairan LiuIsmaila Rimi AbubakarTai-Sheng YehIsraa H. MahmoudTaishi KayanoIsraa M. ShatwanTaisuke TogariIsrael Casado-HernándezTaisuke TomonagaIsrael Fisseha FeyissaTaiwo AremuIsrael IkoyiTaiwo LasisiIsrael Miguel AndrésTakaaki SenbonmatsuIsrael Villarrasa-SapiñaTakaaki TomofujiIssa Sulaiman Al-AmriTakafumi AbeIssac SachmechiTakafumi NakayamaIstiak A. BhuyanTakafumi YanagisawaIstván A. HarmatiTakahide KagawaIstván AndóTakahiro IkedaIstván BerkesTakahiro KannoIstván BodnárTakahiro KitsukaIstván FüzesiTakahiro NemotoIstván KissTakahiro OnoIstvan RajtaTakahiro OtsudoIstván TarrósyTakahiro TabuchiIta HadžisejdićTakahiro TsugeÍtala MarxTakahiro WadaÍtalo Sousa De SenaTakahiro YamazakiItamar GonçalvesTakahisa YanoItandehui Castro QuezadaTakahito OhshiroItay BarneaTakako YasudaItziar Chapartegui-GonzálezTakalani Grace TshitanganoItziar IruarrizagaTakamasa MiyauchiItziar Sobrino-GarciaTakanobu HirosawaIulia D. ArionTakanobu OkamotoIulia MuresanTakanori YasuIulian A. BratuTakao KimuraIuliana CosteaTakashi AsakuraIuliana GheorgheTakashi AzumaIuliana HartescuTakashi HashimotoIuliana VijulieTakashi NaruseIuliia PavlovaTakashi NomizoIustina BoajăTakashi YanoIva BinicTakashi YazawaIva BlazevicTakayuki HaradaIva BužančićTakayuki KageyamaIva HolmerováTakayuki KoikeIva Juranović CindrićTakazawa YujiIva LakićTakehiro MichikawaIva MrakTakehisa HanawaIvan BorrelliTakeji Takamura-EnyaIván CarreraTakemi OtsukiIvan ChorbevTakemichi FukasawaIvan Chulvi-MedranoTakenobu YamamotoIvan Clavel San EmeterioTakenori HarunaIvan ČukTakeshi KannoIvan DimauroTakeshi KawaguchiIvan DimovTakeshi KezukaIván EcheverriaTakeshi MiyamaIván FernándezTakeshi NinchojiIvan Gustavo Masselli Dos ReisTakeshi YamaguchiIván Herrera-PecoTakshashila TripathiIvan JerkovićTaku SuzukiIvan LangeTakuichi SatoIvan LuzardoTakuma InagawaIvan Malagoli LanzoniTakumi YamaguchiIvan Miguel PiresTakuro TobinaIvan MiskulinTakuya NakaiIvan NalvarteTakuya TadaIvan Onone GialainTal Oron-GiladIvan PariseTalaghir Laurentiu-GabrielIvan Peres CostaTalapan DanielaIvan PerićTalat IslamIvan Pires De OliveiraTali Zitman-GalIván Prieto-LageTalia EsnardIvan RodriguezTalía SainzIván Sánchez-IglesiasTalin BabikianIván Santolalla-ArnedoTalya Miron-ShatzIvan ŠirićTamal SadhukhanIvan ŠklebarTamanna DalwaiIvan ŠošaTamantini ChristianIvan StoikovTamar BasishviliIván TartarugaTamar BermanIvan UherTamara B. IvetićIván Valdivia-GandurTamara Besednjak ValičIvan VasenevTamara BozovicIvana BilicTamara BrownIvana BlešićTamara Džamonja IgnjatovićIvana BulogTamara Fernández-ArévaloIvana DjuricicTamara FloricicIvana GrgicTamara GajićIvana GusarTamara GuénounIvana HromatkoTamara Hew-ButlerIvana KmetičTamara JovanovicIvana MihajlovićTamara Lazarević-PaštiIvana MilovanovićTamara MenichiniIvana Načinović BrajeTamara MohorićIvana OleckáTamara Nikolic TurnicIvana Pavlinac DodigTamara Pawlaczyk-KamienskaIvana Rumora SamarinTamara PlatteelIvana SamarzijaTamara SerdinšekIvana Savić PavičinTamara TaillieuIvana ŠkrlecTamara WrightIvana SutejTamás BerkiIvana TadićTamás HaideggerIvana TomacTamás KarchesIvana Tomas ŽikovićTamás KovalcsikIvana TošićTamas MolnarIvanka DimovaTamás OllmannIvano CaselliTamas SzaboIvano VassuraTameka Samuels-JonesIvar ZekkerTamer ElsakhawyIveta BernatovaTamerah HuntIveta VrabkovaTami Bar-ShalitaIvette Margarita Espinoza-DíazTamsin FordIvica MatićTamuka MagadzireIvica PelivanTan Fee YeanIvica StančerićTan Pui YeeIvo HornTanaya SparkleIvo HristovTancredo De SouzaIvo PonocnyTaneisha S. ScheuermannIvo RegliTangao HuIvona SkultetyovaTangela RobertsIvone MartinsTangtian HeIvone Vaz-MoreiraTangui BarréIvonne LozanoTanguy SouliéIvy Ka Yu ChengTania Ahalya ThimrajIvy ShiueTania Cecilia MihaiescuIwan August MeynaarTania ColasantiIwao UeharaTania ContardoIwona BąkTânia CorreiaIwona BatykTania Garfias MacedoIwona Beata PaśmionkaTania GasparIwona Bodys-CupakTânia Gonçalves AlbuquerqueIwona BonieckaTânia LucciIwona GołaśTania ManoIwona Gorczyca-GłowackaTania PearceIwona JazdewskaTania Pinto-EscalonaIwona Kantor-PietragaTania Romo-GonzálezIwona KowalczukTânia RosadoIwona Kuźniarska-BiernackaTania RusIwona MalickaTania SignalIwona Markiewicz-GórkaTanja BukovčanIwona MazurTanja CegnarIwona Olszewska-CzyzTanja Grubić KezeleIwona Piatkowska-ChmielTanja HüschIwona PomianekTanja SchmidtIwona SikorskaTanja VeljovicIwona StaniecTanja WirthIwona SzerTanjina RahmanIwona SzymusikTanneke HerklotsIwona WertelTanseer AhmedIzabel Galhardo DemarchiTansel DokerogluIzabela BartkowskaTansin BennIzabela BetlejTanu AllenIzabela BolesławskaTanuj WadhiIzabela ChmielewskaTanupriya ChoudhuryIzabela DembińskaTanvee ThakurIzabela DudaTanvir KhanIzabela KrupinskaTanya BenitezIzabela Kutschenreiter-PraszkiewiczTanya BozhkovaIzabela Luiza Ciesielska-WróbelTao ChenIzabela Maurício De RezendeTao HuangIzabela Michalska-DudekTao JingIzabela Młynarczuk-BiałyTao LanIzabela PawlakTao LiIzabela PiegdonTao LiuIzabela Sadowska-BartoszTao SongIzabela StalończykTao TongIzabela SztangretTao YangIzabela ZakrockaTao ZhangIzabela ZawiskaTaofeeq Ibn-MohammedIzabela ZóltowskaTaona HaderleinIzabella LeckaTapan ParikhIzabella MajTapan SenapatiIzhak SchnellTar Choon AwIzhar KhanTar IldikóIzidor MlakarTara Clinton-MchargIzonete Cristina GuiloskiTara McIsaacJ. Craig PhillipsTara Patricia HurstJ. D. RodriguezTaraprasad BhowmickJ. Daniel TwelkerTaras HutsolJ. Francis BorgioTarek Ben HassenJ. J. Sylvia IVTarek L. RashwanJ. JumadiTaren SandersJ. Masa-CallesTarik KivrakJ. Matt McCraryTariq Alwada’nJacalyn DuffinTariq MehmoodJacco DuelTariq ShahJacek AntonkiewiczTariq Usman SaeedJacek BarcikTarita BiverJacek BoguckiTarja SuominenJacek BucznyTaro KamigakiJacek CabanTaro TakeuchiJacek ChmielewskiTaro UraseJacek GancarczykTaru S. DuttJacek KawalecTarun JainJacek KlichTarun UpadhyayJacek ŁubińskiTaryn KlarnerJacek Łukasz Wilk-JakubowskiTasadduq ImamJacek MoskalewiczTassanee SilawanJacek OskarbskiTastaftiyan RisfandyJacek PaśTasuku OkuiJacek PolechońskiTatiana A. TrifonovaJacek RogalaTatiana B. FashchevskayaJacek RóżkowskiTatiana BegottiJacek SmerekaTatiana BegunJacek StańdoTatiana BerezinaJacek SzmaglińskiTatiana Casado De StaritzkyJacek SzymańskiTatiana ChristidesJacek TabarkiewiczTatiana ČorejováJacek WasikTatiana De Oliveira SatoJacek WąsowskiTatiana DelgadoJacek WilczyńskiTatiana KremlevaJacek WozniakTatiana MinayevaJack E. FinchamTatiana O. SatoJack HardwickeTatiana SilvaJack HolbrookTatiana TambouratzisJack L. SeymourTatiana Viktorovna KochetovaJack ThepsourinthoneTatiana Yu BraslavskayaJack YanovskiTatiana ZimenkovaJackie Martin-KerryTatiane AssoneJackie Van DaelTatjana FischerJackie WrightTatjana KramaJackie YangTatjana KrstićJackline Freitas Brilhante De Sao JoseTatjana PekmezovicJacky BowringTatjana PõlajevaJacky ChinTatjana PortnovaJacky MathonnatTatsiana PukhalskayaJaclyn C. KearnsTatsuhiko KuboJaclynn M. HawkinsTatsuma OkazakiJacob Arie JordaanTatsunori IkemotoJacob BethemTatsuya FujikawaJacob P. BylTatsuya KoyamaJacob S. ThomasTatsuya MorimotoJacob SkauvoldTatu RojalinJacobo A. Rubio-AriasTatyana KrupnovaJacobo Cano EscoriazaTaufan BramantoroJacobo Rodríguez-SanzTauseef AhmadJacobus Van Der VeldenTavan JanvilisriJacopo A. VitaleTayebeh AbediJacopo CiaffiTaylor J. ReifJacopo Junio Valerio BrancaTaylor LivingstonJacopo PruccoliTayo AdekiyaJacqueline E. ReznikTa-Yuan ChangJacqueline M. DaviesTea GalicJacqueline MarquesTe-Chun ShenJacqueline Rose StarrTecla BonciJacques CabaretTed McDonaldJacques de MouzonTed SaarikkoJacques MansouratiTed YeungJacques OosthuizenTeddy LazebnikJad Adrian WashifTeerawat CharoenratJade CartwrightTeet SeeneJade KettlewellTe-Feng YehJade Lai King WongTehila KalagyJadeera Phaik Geok CheongTehmina KhanJademilson C. SantosTejinder PalJadranka A. SpahijaTek DangiJadwiga KrólikowskaTek Yew LewJadwiga KubicaTekla SzépJadwiga MazurTella LanttaJadwiga SołoduchoTelma AlmeidaJae Bum ParkTelmo PereiraJae Jin SongTelmo Raul Aveiro-RóbaloJae Kyung LeeTelung PanJae Min KimTemidayo S. OmolaoyeJae Seok LeeTemple GrandinJae Yon WonTena NiseteoJae Young LeeTena VerslandJae-cheol LeeTengyao JiangJaehee HanTennley VikJaehwan KimTeodora Alexa-StratulatJaehyeon ParkTeodora DominteanuJaehyuck LeeTeodoro GeorgiadisJaehyun AhnTeoh Sian HoonJaehyun ParkTera L. FazzinoJae-Ik ShinTeralı KeremJaejong ParkTerence A. ImberyJaeseok LeeTerence LeahyJaesin SaTerence MoriartyJaeson CallaTerence OngJaesun WangTeresa BarrosJaewon JoungTeresa Bobes-BascaranJaeyoung KimTeresa De DiegoJae-Young LeeTeresa DieguezJafir ShiraziTeresa FaziaJagadeesh K. VenkatesanTeresa González GómezJagadeesh PuvvulaTeresa GriecoJagadheshwar BalanTeresa LeszczyńskaJagdeep KaurTeresa Letra MateusJagdeep RahulTeresa MadureiraJagdish KhubchandaniTeresa MagalhãesJagoda Kaszowska-MojsaTeresa Makowiec-DąbrowskaJagriti SainiTeresa Marat-MendesJahanpour AlipourTeresa Maria SgaramellaJaheer Mukthar K. P.Teresa MonteJahidur Rahman KhanTeresa PaivaJahirul MullickTeresa PaolucciJa-Hoon KooTeresa PerraJai P. RaiTeresa PinheiroJai PrakashTeresa Rubio-TomásJaideep MahendraTeresa RussoJaikirshan J. KhatriTeresa Sanclemente-HernándezJaime BarrientosTeresa Silveira LopesJaime BoschTeresa Sławińska-OchlaJaime de Pablo ValencianoTeresa SummavielleJaime DelgadoTeresa VelosaJaime DevineTeresa WagnerJaime Estevão ScandolaraTeresina MancusoJaime LopezTereza NovákováJaime Moll De AlbaTereza ZalmanovaJaime NavarreteTerézia PošivákováJaime Ramis SorianoTerézia RončákováJaime Vásquez-GómezTero TuovinenJaimie L. O’GaraTerrence J. RavineJairo L. VanegasTerri PikoraJairo León-QuismondoTerri-Ann BerryJairo Ortiz RevillaTerry C.F. YipJaishree RamanTerry ElseJaka SriyanaTerry FulmerJake CampTerry GordonJake KanerTerry J. FroggattJakkarin LimwongyutTerry V. ShawJakob PietschnigTerry WotherspoonJakub BekierTeruo OnishiJakub CiazelaTeruyoshi AmagaiJakub DuszczykTess DoeffingerJakub GlowackiTessa BakerJakub KołodziejczykTessa CarrauJakub KortasTessa R. EnglundJakub KosteckiTesseltje De LangeJakub KrejčíTessho MaruyamaJakub KubiczekTestusko KasaiJakub M. GacTetiana A. VakaliukJakub MokrzyckiTetsuro KobayashiJakub MorzeTetsuya IkedaJakub PolákTetsuya OgawaJakub StempienTetsuya OhiraJakub SuchánekTetsuya TakahashiJakub SwachaTetyana PimonenkoJakub WięckowskiTeuta MuratiJakub ŻurawskiTewodros R. GodeboJala RizeqThach N. DinhJalel EuchiThaís R. SilvaJam KhojastehThais Suzane Milessi EstevesJamal Ben MansourThaíse Gonçalves AraújoJamal KhatibThanapong ChaichanaJamal MabroukiThanapong ChampahomJamal MamkhezriThangasamy SaminathanJamal MartínThangavelu KokulnathanJamal NaderiThanh Nguyen PhongJamal ZainiThanh-Phong DaoJameel Rizwana HussaindeenThanhTin NguyenJames BednallThanh-Tuan NguyenJames BeirneThanigaivelan KanagasabaiJames BreretonThanos GoulasJames C. OlesonTharshanah ThayabaranathanJames C. SimeonThawatchai PhaechamudJames Cole GallowayThayne KowalskiJames ConeThayse Natacha GomesJames D. KeanTheam Foo NgJames DanckertTheano KokkinakiJames DonaldsonTheera TongsongJames DoorleyThelma SpindolaJames FellTheng Choon OoiJames FrideresTheo HatzipetrosJames Gavin BremnerTheo NiyonsengaJames H. StewartTheodor DollJames HallinanTheodora ManiouJames HokansonTheodora PapamitsouJames HunterTheodore KotsilierisJames J. CochranTheodoros EleftheriadisJames JohnstonTheodoros FouskasJames K. C. ChenTheodoros MavridisJames Kevin SummersTheodoros StamatopoulosJames KimballTheofani AntoniouJames LeighTheresa L. ScottJames Lloyd MichenerTheresa Roma JasineviciusJames LomasTheresa W. GillespieJames LongstaffeTherese AsplundJames MacLeanTherese HellmanJames MaguireTherese Van AmelsvoortJames MakiTheresia KriegerJames NichollsThereza Maria Magalhães MoreiraJames OdeiThersilla OberbarnscheidtJames Omondi OutaTheyazn H. H. AldhyaniJames PeachThi Thi NgeJames PethickThi Thuy Huong NgoJames R. BarthThiago Almeida VieiraJames R. CouchThiago Antonini AlvesJames R. MinerThiago LeonardiJames RileyThiago MaiaJames RussoThiago MurariJames S. MiserThiago Pavoni Gomes ChagasJames S. PowersThiago PoletoJames ScottThibauld MoulaertJames StameyThibault MesplèdeJames StiehlThi-Dieu-Hien VoJames V. CoeThierry Burger-HelmchenJames WalterThierry Oscar EdohJames WaplesThierry RibeiroJames WilliamsThiha Tin KyawJami JonesThi-Kim-Quyen VoJamie De WittThiloththama Hiranya Kumari NawarathnaJamie MarsdenThin Nyein Nyein AungJamie SmithThippa Reddy GadekalluJamie TamThitiya TheparodJamie TaylorThivanka MuthumalageJamil HussainThobela NkukwanaJamille G. DombrowskiTholang Alfred MokheleJamir Pitton RissardoThomas AkintayoJamison ConleyThomas B. EllisJamiu Adetola OdugbesanThomas BaerJammie Lo Kit MeiThomas BakupogJan A. WendtThomas BaranowskiJan Amos JelinekThomas BlaineJan BarwickiThomas BöhlerJan BocianowskiThomas BrandonJan BogackiThomas BrogginiJan BondarukThomas ChaillouJan CebulaThomas CraemerJan ChrastinaThomas DavidsonJan CudzikThomas De PreeJan D’haeseThomas DuffJan DvořáčekThomas EgerJan EhlersThomas F. HestonJan F. YbemaThomas FaitJan FazlagicThomas FischerJan Felix KerstenThomas FrederickJan GC Van AmsterdamThomas George CampbellJan H. RosenvingeThomas GreenfieldJan HaukeThomas HoferJan HenzelThomas J. DinzeoJan HoflandThomas J. GlynnJan HofmanThomas J. LubenJan Jens KoltermannThomas John KeeganJan JungerThomas Jürgen KlotzbierJan K. KazakThomas KantermannJan K. WachterThomas KleinsorgeJan KabátekThomas KocherJan KavanThomas KoutsosJán KnapíkThomas KüpperJan KoltermannThomas LeiJán KováčThomas LichtJan KucharskiThomas M. HinckleyJan KühnischThomas MetaxasJán LeštákThomas N. MaloneyJan MacháčekThomas PerreaultJan Magnus SjøgrenThomas PlocosteJan MarcussonThomas PlümperJan MejzlikThomas PolakJan MieszkowskiThomas Rhys EvansJan MuzikThomas SchaibleJan O. BackThomas ScheidstegerJán OndrušThomas SpoorenbergJan OwsinskiThomas T. H. WanJan P. RöerThomas TenkateJan PizońThomas ThiebaultJan PolcynThomas TschernigJan SaevelsThomas VolkenJan SchröderThomas WaldhörJan SkalkaThomas WanJan Stefan BihałowiczThomas WeberJan TuranThomas WenzelJan Van AalstThomas WolfJan Van AmsterdamThongkholal HaokipJan Van der BorgThórarinn SveinssonJan VychytilThorsten SchwarkJan Wilko SchrickelThowayeb HassanJan ZabrzyńskiThuane RozaJan ZawadkaThuli Godfrey MthembuJana AsherThunwadee SrithawiratJana BabjakováThuy LaJana ChovancováTia RichJana DjakowTiago AtalaiaJana GeroldTiago Augusto MoreiraJana HeczkovaTiago LimaJana HeldTiago NazarethJana KostalovaTian QiangJana MarečkováTian XieJana MarkovaTianan YangJana MašlankováTianhua NiuJana NačeradskáTianhui HuJana PócsováTianjie ShaoJana PoórováTianjun LuJana SemrauTianjun WangJana ŠiftováTianpeng GaoJana SmahelovaTiantian YangJana SuklanTianxiao ZhangJana VasickovaTianyao ZhangJane A. WeintraubTianye ZhaiJane AkisterTianyi LiJane CurrieTianying WuJane E. KrauseTianyu ZhaoJane EntwistleTianyu ZhengJane FletcherTianyuan WangJane HarrisTianyun ZhangJane J. Chung-DoTibebeselassie Seyoum KeflieJane Jie YuTiberius DicuJane KuepferTibor GuzsvineczJane Lilly LopezTibor HortobagyiJane M. GannonTibor SzkaliczkiJane R. OliverTibor ZsigmondJane RuseskiTie LiuJane SimsTiejian LuoJanet A. FooteTien Anh TranJanet BeznerTien Thi Hanh NguyenJanet C. KingTien Tsung LeeJanet Delgado-RodriguezTifeng JiaoJanet DzatorTiffani Josey HowellJanet KellyTiffany Champagne-LangabeerJanet Kit Yan ChanTiffany J. Ríos-FullerJanet LeeTiffany JonesJanet W. M. EllisTihomir DovramadjievJaneth SanabriaTihomir LukovicJanette RoseTihomir OpetukJanez MravljakTihomir OrehovačkiJanez RavnikTiija RintaJang Pyo BaeTiina IkäheimoJang-Kyung ParkTijana CrnčevićJangwon GimTijana IčinJangwoo LeeTijana ŠtajnerJan-Hendrik SchroederTikendra Nath VermaJani LaineTilahun HareguJani SilvaTilemachos KoliopoulosJanice Ann LakeTill BeutelJanice FletcherTill UteschJanice HegewaldTilman ReineltJanice ProbstTilman SteinertJanice R. HermannTim GomersallJanienke A. SturmTim Hideaki TanakaJanina GospodarekTim HulsenJanina Jędrzejczak-GasTim LysykJanine Anne CampbellTim M. SzewczykJanine CampbellTim MarjoribanksJanine JongbloedTim PohlemannJanine L. BarrettTim ReynoldsJanine WhiteTim TaylorJani-Petr I. LaamanenTim ZiemerJanis FiedlerTimea GaborJanitha H. BatagodaTímea JuhászJanja JazbarTimo AiraksinenJanja TrčekTimo FalkenbergJanko JankovićTimo H. VesikariJanna G. KoppeTimo SiikonenJanne LundénTimoteo MarchiniJannick NiortTimothy AdekoyaJános A. OsánTimothy B. DaviesJános Fiala-ButoraTimothy BarzykJanos G. PitterTimothy BungumJanos JuhaszTimothy BushnellJános OsánTimothy C. DurhamJanthima MethaneethornTimothy CiesielskiJanuka KhatiwadaTimothy EricksonJanusz GołaśTimothy EschleJanusz KarwotTimothy G. CampbellJanusz KasperczykTimothy G. FordJanusz KwiecieńTimothy HasenoehrlJanusz MaciaszekTimothy HsuJanusz MajewskiTimothy HunterJanusz MiskiewiczTimothy J. CraigJanusz MyszczyszynTimothy J. UssetJanusz OpolskiTimothy J. WadeJanusz RymaniakTimothy LeeJanusz Sytnik-CzetwertyńskiTimothy LutzJanvier GasanaTimothy M. EschleJapareng LalungTimothy MakubuyaJaqueline Castelo BrancoTimothy McCallJared D. TaylorTimothy N. C. WellsJared SmithTimothy O. AdekoyaJari IntraTimothy PohlmanJari KylmäTimothy R. KeifferJarmila HonzikovaTimothy RittmanJaroslav KacetlTimothy SlyJaroslav M. GutakTimothy StickleJaroslav MarekTimothy TseJaroslav ŠušolTimothy W. FarrellJaroslava ZimmermannTin ČohadžićJarosław ChwastowskiTin HorvatinovićJarosław DomaradzkiTin LukićJarosław HerbertTin PhanJarosław HorowskiTina BartelmeßJaroslaw JanusTina Cvahte Ojsteršek Cvahte OjsteršekJarosław Jaszczur-NowickiTina KostkaJaroslaw Meyer-SzaryTina KubrakJarosław PinkasTina LindhardJaroslaw S. KardasTina McKayJarosław SerafinTina PodlogarJarosław WaloryTina Poklepovic PericicJarosław WitkowskiTina SeverJarotwan KoiwanitTina TaylorJarred PrudencioTina Tičinović KurirJaruwan ChontanawatTina TomazicJar-Yuan PaiTina Unuk NahbergerJasemin TodriTina VrabecJasenka WagnerTinakon WongpakaranJasenko LjubicaTine VertommenJasmin AsbergerTing BaoJasmin RauseoTing HanJasmina IsakovićTing Kin NgJasmina Tomašić HumerTing NieJasminka AlijagićTing WangJasminka LaznjakTing XiaJasminka TalapkoTinggang LiJasna DjordjevićTingguang MaJasna KaračićTingting ReidJasna Šulentić BegićTingting ZhangJasneet GrewalTingyu ChangJason BarrTin-Tin Win-ShweJason BrumittTinyiko Enneth NkhwashuJason CohenTissiana BortolottoJason CooperTitkova TatianaJason D. FlattTito BrambulloJason FlatTito PizarroJason FoglerTiziana CappelloJason HungTiziana CentofantiJason James TurnerTiziana CiarambinoJason KilmerTiziana CrovellaJason MillsTiziana GreggiJason ParksTiziana GuzzoJason R. JaggersTiziana PietrangeloJason ReeceTiziana SuscaJason RotoliTiziano TestoriJason S. BuhrmanTjhin WigunaJason T. HarrisToan HaJason T. MagnusonToan Nguyen-SyJason W. MarionTobia LakesJason WilsonTobias BertschJasper KnightTobias DünnwaldJaspreet KaurTobias GoeserJaspreet SinghTobias KalischJassiel Vladimir Hernández-FontesTobias KünklerJasti SateeshTobias KutznerJatinder Kaur KatnoriaTobias Kvist StrippJauhar IqbalTobias SchmidtJaume Ferrer-SanchoTobias SchrippJaume Miquel March AmengualTobias Skuban-EiselerJaume Miranda-RiusTobias WeinmannJaume VivesTobias WittJavad AlizargarTobin SilverJavad Javan-NoughabiTodd AstorinoJavad Shafiei ShivaTodd BeerJaved HaneefTodd CunninghamJavid IqbalTodd M. JensenJavier Almela-BaezaTodd RogersJavier Bustos DíazTodd T. SchlegelJavier Caballero-VillarrasoTodor KundurzhievJavier Cabeza-RamírezTo-Hung TsuiJavier Campanini-SalinasToktam KhatibiJavier Carbajo-LozoyaTolessa DeksissaJavier CarniceroTolga OlmezJavier Cortes-RamirezTolga TezcanJavier Dóniz-PáezTolga ZamanJavier Eloy Martínez GuiraoTom AllenJavier F. BoyasTom ClarkJavier Gamez-PayaTom CoxJavier Gíl AresTom DanielsJavier Gil-GimenoTom DeningJavier HerruzoTom Elijah VolenzoJavier Jerez-RoigTom GarnerJavier Marco-LledóTom KovitwanichkanontJavier Marco-MartínezTom L. BroderickJavier Mendoza-JiménezTomas AlbrektssonJavier Merino-AndresTomáš BüchlerJavier Moreno-LázaroTomáš HavránekJavier Oliver-MuncharazTomas Herrera-ValenzuelaJavier Ortuño SierraTomas JungertJavier Parra DomínguezTomas KåbergerJavier PereiraTomas KralikJavier Reina-TosinaTomas KrulickyJavier Reyes-MartínezTomás Martínez-TrinidadJavier Rico-DíazTomas NovotnyJavier Rodriguez-FalcesTomáš PeráčekJavier Ruiz RamírezTomás RecioJavier Sanchez-SanchezTomáš SeemanJavier Sanz-ValeroTomas SoukupJavier TarangoTomas TamošuitisJawahitha SarabdeenTomáš VencúrikJaw-Yuan WangTomaso VairoJay PooleTomaso VecchiJaya BishwalTomasz BanasJaya JhaTomasz BoińskiJayakrishna KandasamyTomasz CholewaJayanarayanan KuttippurathTomasz CudejkoJayanta MondalTomasz DudaJayasmita JanaTomasz DzierżanowskiJayasree BasuTomasz GabryśJayden HunterTomasz GigolJayesh J. AhireTomasz GrodzickiJayeshkumar PatelTomasz GrzywaczJayme E. WaltersTomasz HalskiJaymie MelikerTomasz HermanowskiJayson StoffmanTomasz JakubowskiJazmín Mora-RíosTomasz JedynakJe Hyun SeoTomasz KalakJeadran Malagón-RojasTomasz KlekielJean Claude De MauroyTomasz KotowskiJean Croce HemphillTomasz KrukowiczJean D. Hallewell HaslwanterTomasz KubrakJean GoldingTomasz KuligowskiJean GrassmanTomasz ŁabuzJean Jacques MonsuezTomasz LesiowJean M. HughesTomasz ŁysakJean Michel CostesTomasz M. NapiórkowskiJean Michel MaixentTomasz MichalskiJean PerriotTomasz NiedobaJean Pierre KabueTomasz NiedzińskiJean RossTomasz OwczarekJean VanderpasTomasz PobożyJean VillerdTomasz PodgórskiJean WooTomasz RakJean Yves HeurtebiseTomasz RogozińskiJean Yves UwamunguTomasz RokickiJean-Baptist du PrelTomasz SekutowskiJean-Bernard MabireTomasz SiwulskiJean-Christophe GigerTomasz SłowikJean-Claude PaironTomasz SmalJean-Claude ThillTomasz SobierajskiJean-Denis ParisseTomasz SosulskiJeanette A. LawrenceTomasz StudzienieckiJeanette ErikssonTomasz SzablewskiJeanette M. AndradeTomasz TeleszewskiJean-Gabriel ContaminTomasz TokarekJeanine M. BuchanichTomasz UrbanowiczJeanine YoungTomasz WarzochaJean-Jacques MonsuezTomasz WolnyJean-Jacques TempradoTomasz ŻądłoJean-Marc RetrouveyTomasz ZielińskiJean-Marie AnnoniTomasz ZwęglińskiJeanne A. M. C. DirksTomaž ŠtupnikJeanne BertolliTomi ZlatarJeanne CartierTomina Gabriela SăveanuJeanne HolcombTomislav ĐurkovićJeanne Mager StellmanTomislav KokicJeanne SlatteryTomislav RukavinaJeanne WenosTomislav RupčićJeannette A. GoudzwaardTomislav TostiJeannie Vander LindeTomislav VladušićJean-Patrice BaillargeonTomiyo NakamuraJean-Philippe BerteauTommasina PianeseJeba Jesudoss ChelladuraiTommaso B. JanniniJeehoon SohnTommaso CaiJee-Hyun HwangTommaso ErcoliJee-In KimTommaso FilippiniJeeyoung LimTommaso GrecoJee-Young MoonTommaso ScquizzatoJeferson SantanaTommaso VitaleJeff AlbertTommi GainesJeff BattigelliTommy Cabeza De BacaJeff C. RabinTomoharu KitadaJeff McQueenTomohide KuboJeff VictoroffTomohiko AoeJefferson E. Contreras-RoperoTomohiko IsobeJeffrey A. BerinsteinTomohiro HigashiJeffrey AdlerTomohiro MatsuoJeffrey BakkenTomoki AbeJeffrey BaraTomoki MaekawaJeffrey BuchsbaumTomoki SekiguchiJeffrey ChanTomoko KodamaJeffrey GilbertTomoko OguriJeffrey Hugh GambleTomoko OmiyaJeffrey J. ParrTomoko SteenJeffrey L. MetznerTomoko UekitaJeffrey S. ForsseTomomi HigashiJeffrey S. HallamTomomichi OgataJeffrey S. MartinTomonori YasukawaJeffrey SallenTomotaka UgaiJeffrey T. HowardTomoya ItataniJeffrey VanWormerTomoyoshi KomiyamaJeffrey WhiteTomoyuki KanazawaJeffrey WickliffeTomoyuki KobayashiJegan IyyathuraiTomsa RalucaJehad A. KharrazTonća JukićJeih-Jang LiouTong GuoJekaterina SchneiderTong WangJelena AlićTong XiJelena DikićTongcui MaJelena DjokićTonghui DingJelena DorčićTonglin JiangJelena Jovičić-BataTongqing LiJelena KovacevicTongsai JamnongkanJelena MandičTongtong LuJelena Marković FilipovićTongwen LiJelena MileševićToni ModricJelena Nesovic OstojicToni P. MilesJelena RadonićToni Torres-McGeheeJelena Ristić TrajkovićTonia VassilakouJelena RoganovicTonino TrainiJelena ŠaracTonkin SarahJelena T. PetrovićTonking BastolaJelena Vapa-TankosicTonmoy ChoudhuryJelena VeličkovićTony CassidyJelena ViderTony KiszewskiJelena ŽivančevTony WallJelica Grujić-MilanovićTony WatersJelili Adegboyega AdebiyiTonya E. WallsJemma KönigTonya R. HammerJen MolloyTope OyeladeJenae M. NelsonTor Georg JakobsenJenalee HindsTorberg FalchJenna SciscoTorbjörn LedinJenni BlomgrenTorcicollo IsabellaJenni LehtimäkiTord Finne VedøyJenni ManuelTore BonsaksenJennie D. CoxTöres TheorellJennifer A. CrittendenTorfinn HynnekleivJennifer A. HallTorranis RuttanaphanJennifer A. JayTorsten PastorJennifer AndersonToru HorinouchiJennifer BossiToru TaguchiJennifer BradleyToru YoshikawaJennifer Bragg-GreshamToscanelli CeciliaJennifer BrayToshiaki TanakaJennifer BunnToshiaki YamadaJennifer ChippsToshiharu MitsuhashiJennifer ChopraToshihiro KawaeJennifer Cornacchione RossToshihisa DoiJennifer Coston-GuariniToshikazu ShinbaJennifer CreeseToshimi ChibaJennifer D. DeatonToshimitsu KatoJennifer D. McMillenToshimitsu OndukaJennifer D’SouzaToshinobu KazuiJennifer DanielsToshiyuki KawaiJennifer FieldsToshiyuki MatsuiJennifer GerlachTosin PopoolaJennifer GoffTotan GaraiJennifer GubitosaTracey CockertonJennifer HansonTracey GendronJennifer HartleTracey ThornleyJennifer HewsonTraci Rose RiderJennifer HoggTracy De WaldJennifer InnisTracy L. OliverJennifer J. BarbTracy ReibelJennifer KentTram CatJennifer L. ReevesTran Dang XuanJennifer L. WarnickTraugott RoserJennifer LehmannTravis Wilson WittJennifer LiraTreena OrchardJennifer Lynn MabryTrefor A. EvansJennifer Miner WeaverTrenton D. MizeJennifer MytychTrevan HatchJennifer PatnaikTrevor GersonJennifer PomeroyTri Retnaningsih SoeprobowatiJennifer R. SchroederTriantafyllos DidangelosJennifer Robertson-WilsonTricia J. JohnsonJennifer RoodTrim LajqiJennifer RuddTrimika L. BowdreJennifer RunkleTrine Strand BergmoJennifer SalinasTrinidad Luque-VaraJennifer SassTrinidad Sentandreu-MañóJennifer SdunzikTrinitat CambrasJennifer SneddonTrisha L. AmboreeJennifer Van WykTrishnee BhurosyJennifer Williams RobinetteTristan CaseyJennifer WooTroy A. LadineJennifer ZinkTroy PurdomJennifer ZuercherTrude HandalJenn-Ming YangTrudi CameronJenny FeredayTrudy NormanJenny LenkeitTrung NguyenJenny PangeTsang-Kai HungJenny RuduchaTsehay Admassu AssegieJenny Van Der SteenTse-Lun ChenJenny Vilchis-GilTse-Yang HuangJenny WilkinsonTshepo RasekabaJenny-Ann Brodin DanellTsige-Yohannes HabteJens BrauneckTsolanku MalieheJens HolstTsu-Ming YehJens JetzkowitzTsung Li LinJens KleinTsung-Cheng HsiehJens StrohaekerTsung-Hsien LeeJenshinn LinTsung-Jen WangJeong Hwan LeeTsung-Ju LeeJeong-eun LeeTsung-li LinJeong-hae ChoiTsung-Wen ChienJeongho JeongTsung-Xian LeeJeong-Hoon LeeTsuyoshi KawakamiJeong-Hun WonTsuyoshi OgataJeong-Hwa HoTsuyoshi TajikaJeongsoo YuTsvetelina VelikovaJeongwoo LeeTsz Ho KwanJeongyun ParkTualar SimarmataJeon-Young KangTuan T. NguyenJeoung Mi KimTuba Çiğdem OğuzoğluJeppe Zielinski Nguyen AjslevTudor CiumaraJera GregorcTugrul YakupogluJer-An LinTuire KuusiJere KoskelaTulip A. JhaveriJeremiás BaloghTunde PetoJeremie D. OliverTung-Hsun HsiehJeremie RichardTurgut KarakoseJeremy AkersTuri Reiten FinseråsJeremy D. MarksTuruna S. SeecharanJeremy ForemanTushar BambharoliyaJeremy HapetaTushar ModiJeremy ZwiegelaarTusneem KausarJer-Hao ChangTvrtko HudolinJeroen R. VermeulenTy Anna LovatoJeroen Van SchependomTyler D. HarveyJérôme AddaTyler E. GastonJerome NriaguTyler J. KybartasJerónimo Aragón-VelaTyler J. TitcombJerónimo González-BernalTymoteusz MillerJerónimo Vida ManzanoTyng Yuan JangJerraco Leontae JohnsonTyphanye Vielka DyerJerre Mae TamanalTyrone PretoriusJerred Junqi WangTyrone T. LinJerris HedgesTze-Huan LeiJerry Chin-Yi LinTze-Kiong ErJerry CourvisanosTzen Ying Jenny LingJerry KoloTzoutzas IoannisJerry T. MitchellTzu Hua WangJerylee Wilkes-AllemannTzu Tsun LukJerzy BeltowskiTzuan A. ChenJerzy BertrandtTzu-Chieh ChouJerzy ChudekTzu-Chueh WangJerzy GrabińskiTzu-Hsuen YuanJerzy JeznachTzu-Hua WuJerzy KaźmierczykTzu-Ling ChenJerzy KisilowskiTzung-De LinJerzy LisekTzuping ChiangJerzy NiemczykTzu-Tsung WongJerzy RomaszkoTzu–Yi PaiJerzy SowaTzu-Yu PengJerzy Wojciech MietelskiUan Carlos Briede-WestermeyerJerzy ZoladzUchendu Eugene ChigbuJesper Bo NielsenUday ChintapulaJesper KristiansenUdi LebelJesper T. N. KnijnenburgUdit SaxenaJess VergisUdo SchumacherJesse AcevedoUelinton PintoJesse C. KrakauerUgo CarraroJesse D. HinckleyUgo CovaniJesse ThornburgUgo FedeliJessica A. GriegerUgo IndraccoloJessica Allia WilliamsUgur Korkut PataJessica Ann DiehlUjjwal LayekJessica BriffaUlas AkkucukJessica BurraiUlderico FreoJessica CastagnaUlderico SantamariaJessica CastonguayUlf IsakssonJessica D. HoffmannUlf NestlerJessica GleasonUlf SchminkeJessica Ibarra MoraUliana AndrusivJessica J. TuanUlises De La Cruz-MossoJessica L. ElfUlla Hellstrand TangJessica L. KingUlla KampmannJessica L. LuceroUlrich GlitschJessica M. K. StreitUlrich JuliusJessica M. RayUlrich PecksJessica MassonniéUlrich SchörkenJessica P. AnandUlrich SinschJessica P. CerdeñaUlrich ZisslerJessica PistellaUlrike A. GischJessica R. DreistadtUlrike BinskerJessica Rial-VázquezUlrike FrischenJessica StubbingUlrike GelbmannJessica ThomsonUlrike Popp-BaierJessica VenturaUlrike WerbanJéssica WeilerUlubeyli SerdarJesús Adrián LópezUma Shankar SinghJesús Alberto Valero-MatasUma ShankerJesús Alejandro Prieto-AmparánUmair RiazJesus Barrena-MartínezUmakanta MishraJesús Chazarra-ZapataUmapathi ReddicherlaJesús Collado AgudoUmar DanoJesús Díaz-GarcíaUmar DrazJesús Francisco García-GavilánUmar FarooqJesús Funuyet-SalasUmar SuyantoJesus Gabriel Rangel-PerazaUmberto GaragiolaJesús García RubianoUmberto MoscatoJesús García-JiménezUmberto SolimeneJesus IbarluzeaUmit AkirmakJesús Jiménez-LópezUmut ÜzarJesús Jiménez-RuizUnai AzcarateJesús López BelmonteUnai Fernández GámizJesús María AlvaradoUnchalee SuwanmaneeJesús María López-ArrietaUnnur OttarsdottirJesús Marín-SáezUrbano LugrisJesús Martínez De La CalUriel Haile Hernandez-BelmonteJesús MateosUriel ZapataJesus Mauricio Flórez ParraUrooj KamranJesús MayaUroš KlanšekJesús Mejía-SaavedraUroš MarušičJesús Mena-ÁlvarezUrša VilharJesús MengualUrska DobersekJesús Miranda PáezUrška FricJesús Molina-MulaUrsula Gundert-RemyJesús Montero-MarínUrsula MartinezJesús Morón-LópezUrsula Mueller-WedanJesús Oliva-Pascual-VacaUrsula PanznerJesus S. JimenezUrsula TrummerJesús Sáez PadillaUrsula Von MandachJesus Saiz GaldosUrszula AmbroziakJesus Seco-CalvoUrszula FilipkowskaJesús TresguerresUrszula KotowskaJesús TrigoUrszula Krupa-KozakJesús VeraUrszula Lelek-BorkowskaJesús-Nicasio García-SánchezUrszula Markowska-PrzybyłaJette AmmentorpUrszula RyciukJetty Duffy-MatznerUrszula WydroJeu-Ming P. YuannUsama AhmedJeyasakthy SaniasiayaUsama Al-MulaliJey-R VenturaUsama ElewaJhon Castaño-OsorioUsman GhaniJi Hyun ParkUsman Khalid ChaudhryJi LiUsman SattarJi LiuUte GschwandtnerJi Wook JeongUtkarsh KohliJi Young ChoUtkarsh MitalJi ZhengUU YanagiJia ChenUwe HoffmannJia LeeUwe MüllerJia NingUwe Von FritschenJia ZhongUzma HaseenJiabin YuUzma ZaidiJiabo GengV. G. GirishJiacheng JiaoV. K. KumarJiafen HuV.L. MangeshJia-Feng ChangVaclav BuncJiahao PengVadim DukhaninJiahe LiVadim FetisovJiahua GuoVadim TseilikmanJiahua WeiVafaeinejad AlirezaJiajia ShanVahid ArabzadehJiajia SongVahid FarzanehJiake XuVahid Najafi Moghaddam GilaniJialei DuanVahid RakhshanJialiang LiuVaida SteponavičienėJiaman DaiVaidas MorkevičiusJiaming WangVaidyanathan SubramanianJiaming WuVaios PeritogiannisJian ChenVaiva LesauskaiteJian HangValackienė AstaJian HuVálber César Cavalcanti RozaJian LiValderi R. Q. LeithardtJian LinValene Garr BarryJian LiuValentin C. MihaiJian SangValentín Emilio Fernández-ElíasJian WangValentin M. VetterJian XuValentin Marian AntohiJian YangValentín Molina-MorenoJianan ZhaoValentin Nicolae VarlasJianbang DuValentin RomanovskiJianbo TongValentin SinitsynJiancang XieValentina BaccoliniJianfei CaoValentina BrombinJian-Feng JiaValentina Brzović RajicJiangang ChenValentina BucciarelliJiangbo QiaoValentina BudaJiangguo ZhangValentina BugelliJiangjin LiuValentina ButvinaJiangtao DuValentina CastelliJianguo ChenValentina CesariJiangyuan WangValentina ChkoniyaJianheng ZhaoValentina Diana RusuJian-Hong YeValentina E. BalasJianhua GongValentina FainardiJianhua ZhuValentina GinevičienėJianhui BaiValentina LaganàJianhui XuValentina LyubomirovaJianhui YuValentina MantulenkoJianing FuValentina MussiJianjun ChenValentina NdouJianlan ZhouValentina ObradovićJianlei GuValentina Ofelia RobescuJianmei GaoValentina Pavlovna ZverevaJianmin QiaoValentina PrestaJiannan LiValentina RahelićJianping CaoValentina RovelliJianping MaValentina RovielloJianrang WangValentina RusuJiansheng QuValentina TaluJiansong WuValentina TottiJianting ZhuValentina VasileJianwei HuangValentina-Mariana ManoiuJianwei ShiValentine Joseph OwanJianwen WeiValentines ChisuJianxiong ZhuValeria AlbanoJianye LiuValeria BacaroJianyou GuoValeria BachioccoJianzhong ZhangValeria Cesario TimesJianzhou HeValeria CostantinoJiao YuValeria FanghellaJiaoxia SunValéria Feijó MartinsJiaoyang LiValeria GaspariJiaqi LiValeria GiorgiJiawei XiangValeria Ochoa-HerreraJiawei ZhongValeria PasciuJiawei ZhuValeria SebriJia-Wen LinValeria StefanelliJiaxu ZengValeria TrivelloneJiayi JiValeria VendittiJiayu LiValeriana GuijoJiayuan WangValerie CainesJiayue GuoValerie de MarinisJiayue YanValerie Diane ValerianoJie ChangValerie GladwellJie ChenValerie KayJie DaiValeriia DemarevaJie GaoValeriia HaberlandJie GuoValerio BonavolontàJie MaValerio BrunettiJie WangValerio Dell’osteJie XueValerio Francesco AnneseJie YinValerio LeoniJie ZhangValério Monteiro-NetoJie ZhuangValerio PellegriniJieke JiangValerio PisaniJielu LinValeriy SnakinJiemin ZhuValesca AnschauJien Jiun ChenValliappan RamanJi-eun ShinValter M. Azevedo-SantosJifang WanVan Chien NguyenJifei ZhangVanaja Krishna NaikJigar ModiVan-An DuongJihad A. A. AwadVance G. NielsenJih-Fu TuVanda Maria AndradeJihoon JungVandana VarmaJih-Shun LiuVandilson Pinheiro RodriguesJihwan HaVanesa Cantón-HabasJi-Hye KimVanesa JordaJihye LeeVanesa Santás-MiguelJihyung HongVanessa AntunesJii Bum LeeVanessa Arán-FilippettiJijia WangVanessa Arizo LuqueJijian FanVanessa AssummaJikai WenVanessa C. SomohanoJill AndersonVanessa ChoJill E. LuotoVanessa ClarkJill JenkinsVanessa OddoJill JoyceVanessa PaulyJill M. CastekVanessa Zorrilla-MuñozJill M. MaplesVangelis SarlisJill M. SwirskyVanish KumarJill MurphyVanja KopilašJill ParnellVanja VasiljevJill R. SilvermanVarameth VichiensanJim BonhamVargas Nicole TheresaJim CampbellVarol Onur KayhanJim ChamberlainVarpu WiensJi-man KimVarsha JainJimi FrancisVarsha SrivastavaJimmy BordarieVarun BansalJimmy EfirdVarun GuptaJimmy Martin-DelgadoVarun Kumar SinghJin BaiVarun PaulJin ChenVasa LászlóJin E. Kim-MozeleskiVasanth KumarJin HeVasco VazJin Hyuck LeeVasile BaltacJin Hyuck ParkVasile DogaruJin JunVasile GherheșJin LiuVasile GramaJin Su KimVasile PotopJin WeiVasile SandruJin Youp KimVasilije StambolijaJina MahmoudiVasiliki D. AssimakopoulosJina OhVasiliki KaioglouJinbo HeVasiliki LeventakouJinbo XieVasiliki Maria IliadouJinchul KimVasiliki MollakiJinda QiVasiliki SyriopoulouJin-Ding LinVasiliki TsigkouJinfeng LiVasiliki VranaJinfeng XuVasiliki YotsidiJing GuVasiliki ZisiJing Han BehVasilios LiordosJing LinVasilis EvagelopoulosJing LiuVassileios FirfirisJing LuVassilios MakrakisJing ShenVassilios PanoutsakopoulosJing WangVassilis BarkoukisJing XuVassilis George AschonitisJing YiVasuki RajaguruJing YinVasyl LozynskyiJing ZhangVaughan W. ReesJingang LiangVedbar Singh KhadkaJingchen ZhaoVedran KrevhJinghu PanVeerachai TanpipatJingjing YinVeeraragavan SachithanandamJingli LiuVelga SudrabaJingling LiVeli YilanciJingping QiuVelia CassanoJingwei ShenVenceslas GoudiabyJingwen CaiVenera-Mihaela CojocariuJingxia LiuVenerina JohnstonJingyi ChiVenija CeroveckiJingyi ShiVenkadesh Sarkarai NadarJingyu WangVenkata Lakshmi Narayana KomanapalliJin-Ho YunVenkata Rajesh YellaJinhuang MaoVenkatakrishnan RengarajanJin-Hwa JungVenkatesh KatariJinjin LuVenkateshwari VaradharajanJinjin WangVenkateswarlu NalluriJinjun XiaVera AlmeidaJinkwan KimVera AmicarelliJinkyung ParkVera BrenčičJinling CaoVěra HubačíkováJinlong LiuVera LarinaJinlong ZhangVera MartinsJinlong ZhaoVera RarJinman PangVera Sa-IngJinming HanVera SimoesJinping LiuVera SnezkoJinpitcha MamomVera StaraJinsoo HwangVera Teixeira ValeJin-Tung LiangVera V. YurakJinwei DongVera Van ZoestJin-Woo JeongVered Kaufman-ShriquiJinxiu WuVerena AndreattaJin-Xu LinVerena BarbieriJinyan YangVerica PavlicJin-Yong LeeVerna B. McKennaJinyou ShenVernon M. ChinchilliJirapas JongjitwimolVernon Shane PankratzJiří ČeněkVeronica AranJiri HrdyVeronica BaioccatoJiří JandaVeronica CimolinJiří KantorVeronica CoramJiri MarsalekVeronica De SimoneJiří NeubauerVeronica L. TimbersJiri PlasekVerónica Marín DíazJiri PospisilVeronica MercuțJiří SchneiderVeronica Mugarab SamediJiří SuchýVeronica PapaJiří VacekVeronica Perez-CabezasJiro OkumuraVerónica Rocío Vásquez-GarzónJisu KimVeronica SpinelliJitendra KanshanaVerónica V. Márquez-HernándezJitendra KumarVeronica VelascoJitendra SinghVerónica Velasco-GonzálezJitka VackováVeronika BelousovaJiun-horng TsaiVeronika BocsiJiunn-Wang LiaoVeronika ShavlokhovaJiun-Yi WangVeronika StoffaJiuyuan HuoVeroniqa LundbäckJiwon LeeVeruscka LesoJi-won RyuVesa KuikkaJixuan MaVesna KosecJiyong DingVesna KrstićJi-Yong JungVesna Mijatovic-JovinJiyoung KimVesna Nikolic JokanovicJizheng PanVesna Tumbas ŠaponjacJo AldridgeVesna Vukašinovic-PešicJo Anna ShimekVesna ZupančičJo Anne OliverVessela KrastevaJo BjorgaardVesta SteiblieneJo Britt-RankinVetriselvan SubramaniyanJo Ellen M. SeftonVett LloydJo KatoViachaslau FilimonauJo ReyViacheslav OsadchyiJoachim BachnerVibeke GravenJoachim G. VossVibor MilunovićJoachim GreilbergerVicenç Hernández-GonzálezJoachim KowalskiVicent VillanuevaJoachim VossVicente Escamilla-RiveraJoachim WestenhöferVicente EstradaJoan Espaulella-PanicotVicente GabardaJoan Francesc Fondevila GascónVicente Gea-CaballeroJoan H. LeungVicente Giner-GalvanJoan HarveyVicente Guerola-NavarroJoan Ignasi Mestre-PintoVicente Miñana-SignesJoan Puig-BarberàVicente Morell-MengualJoan Ramon Rodríguez-AmatVicente Romo PérezJoan SantamariaVicki L. SimpsonJoan Sole-PlaVicki SquiresJoan Viciano BadalVicky Panduro-CorreaJoana Abou-RizkVictor AdekanmbiJoana Alegria QuintelaVictor Alejandro Suarez TorielloJoana CimaVíctor Arufe-GiráldezJoana Cristina Cardoso GuedesVictor CepoiJoana CruzVictor Díaz-Flores GarcíaJoana DuarteVictor Diogho Heuer De CarvalhoJoana Faria MarquesVictor Doménech GarcíaJoana FernandesVictor Fernandez NascimentoJoana FerreiraVíctor Gómez-MayordomoJoana FigueiredoVictor H. P. PattersonJoana LageVictor InfanteJoana NobreVíctor José Villanueva-BlascoJoana PrataVictor M. González-ChordáJoana Proença BeckerVictor M. Jimenez‪Joana RoloVíctor M. RiveraJoana Sofia Dias Pereira De SousaVictor Manuel Gonzalez-ChordaJoana SousaVictor Manuel Sanchez-CorderoJoan-Francesc Fondevila-GascónVictor MartinJoann SorraVíctor Mauricio HerreraJoanna BaginskaVictor MinichielloJoanna BąkVictor NorrefeldtJoanna BaranVíctor RincónJoanna Bartkowiak-WieczorekVictor Ruiz-GarcíaJoanna BasiagaVictor SierpinaJoanna BogusławskaVictor Talarico Leal VieiraJoanna BryśVictor TiberiusJoanna BurzyńskaVíctor Toro-RománJoanna Dec-PietrowskaVictor ValverdeJoanna DipnallVictor VianaJoanna Domagala-KulawikVictor W. PerezJoanna DudekVictor W. WeednJoanna FronczykVictor ZaiaJoanna GaitensVictor-Alexandru BriciuJoanna GarstangVictoria BelousovaJoanna Gmitrowicz-IwanVictoria BogdanJoanna GodlewskaVictoria Calvo-FuenteJoanna GorwaVictoria G. RontoyanniJoanna Guziałowska-TicVictoria GonzalezJoanna JabłońskaVictoria GrunbergJoanna JaroszewiczVictoria HouldenJoanna KamińskaVictoria L. BoggianoJoanna KapustaVictoria OsipovaJoanna KazimierowiczVictoria Ramírez GonzálezJoanna KizielewiczVictoria S. McKennaJoanna Kos-ŁabędowiczVictoria Soto-SanzJoanna KossewskaVictoria TeamJoanna Kowalczyk-AniołVictoria Valeriivna RodinkovaJoanna KowalskaVictoria WealeJoanna KwiecienVictoriya A. GeorgiyantsJoanna L. FiddlerVid VelikicJoanna LawrenceVidal Guerra-De La CruzJoanna Le Thanh-BlicharzVidanka VasilevskiJoanna ŁukasikVidas ŽuraulisJoanna M. StreckVidhisha VyasJoanna MazurVidit BhargavaJoanna MuszyńskaVidya AndersonJoanna Myszkowska-RyciakViera ŠvihrováJoanna Pawłowska-TyszkoVieri LastrucciJoanna PieczyńskaViet LeJoanna Pietrzak-ZawadkaViet TranJoanna Popiołek-KaliszViet-Khoa Tran-NguyenJoanna Rachtan-JanickaVijay IndukuriJoanna RatajczakVijay Kumar VermaJoanna RógVijay ReddyJoanna Rosak-SzyrockaVijay VictorJoanna RucińskaVijaya Saradhi MettuJoanna Sadłowska-WrzesińskaVijayabhaskar VeeravalliJoanna Smoluk-SikorskaVijayakumar SukumaranJoanna StawskaVijayashree Sathyanarayana MysoreJoanna StefanowiczVijendra Kumar PandeyJoanna StorieViji VijayanJoanna SuliburskaVikas KumarJoanna Szala-RycajVikas PalakondaJoanna Szczepańska-GierachaVikash KansalJoanna SzydłoVikki HouldenJoanna WieprowVikkie MustadJoanna Wiktoria Przeździecka-DołykVikram AdhikarlaJoanna WincenciakVikram JoshiJoanna WitkosVikram NiranjanJoanna Wnęk-GozdekVikrant RaiJoanna Wyczarska-KokotViktar LemiasheuskiJoanna WyszkowskaViktor BrygadyrenkoJoanna ZarębskaViktor FredrichJoanne BrookeViktor GoliasJoanne BryantViktor SchlosserJoanne K. HeymanViktor TrynchukJoanne LloydViktor V. NosovJoanne PikeViktoriia VovkJo-Anne PuddephattViktorija ČepukienėJoanne RoseViktoriya PantyleyJoanne ThandrayenViktoriya S. SidorenkoJoanne W. WilliamsVila Mª HelenaJoannes ChliaoutakisVilhelmova-Ilieva NeliJoão Antônio Leal De MirandaVili DragomirJoão Batista Ferreira-JuniorVilija Bite FominieneJoão BotelhoVille AlopaeusJoão BritoVille SivonenJoão CaramêsVilma KriaucionieneJoão Carlos Correia LeitãoVilma PinchiJoão Carlos MacedoVilma XhakollariJoão CruchoVimala RamooJoao Felipe CostaVinagre AlexandraJoão Flávio Da Silveira PetruciVinal MenonJoão Gama MarquesVinay KumarJoão Gaspar-MarquesVinay ShivannaJoão HenriquesVinayak JoshiJoão LimaVinayak KhodadeJoão Lucas Della SilvaVinayak VijayanJoão M. LopesVincent BerardiJoão MachadoVincent GroteJoão MaltaVincent MalletJoão Manuel Garcia Do Nascimento GravetoVincent NijmanJoão Manuel Graça FradeVincent ReyesJoão Manuel Mendes CaramesVincent TeeJoão Manuelda Silva CarvalhoVincent WildingJoão MartinsVincente Javier Clemente-SuarezJoão MattarVincenza CaponeJoão Miguel Marques dos SantosVincenza GianfrediJoão NevesVincenzo CannizzaroJoão P. MonteiroVincenzo Cristian FrancavillaJoão Paulo Silva Do Monte LimaVincenzo De CeglieJoão Paulo TribstVincenzo DeufemiaJoão Pedro DuarteVincenzo DovìJoão Pedro GregórioVincenzo GianturcoJoão PetricaVincenzo MarcotrigianoJoão PiedadeVincenzo Nunzio ScalcioneJoão PinheiroVincenzo NuzziJoão R. MesquitaVincenzo PatellaJoão Ramalho-SantosVincenzo PreteJoão ReisVincenzo QuagliarielloJoão Renato Rebello PinhoVincenzo Romano SpicaJoão SerraVincenzo SaliniJoão SerranoVincenzo VizziJoao SilveiraVincenzo ZanonJoão Silvestre Silva JuniorVineet Kumar KamalJoão TavaresVineet SinghJoão VasconcelosVinícius Coelho CarrardJoão Vicente De AssunçãoVinícius SilvaJoão Vitor GuerreroVinit GholapJoãodos Santos BaptistaVinit RajJoaquim CarrerasVinko PaulićJoaquim ChalerVinny NegiJoaquim FarguellVinod KumarJoaquim M. B. PinheiroVinod PatelJoaquín González-RódenasViola IntroiniJoaquín López PascualViola OldratiJoaquin Mora-MerchanViola SomogyiJoaquín NavajasViolante MartínezJoaquin TavernerVioleta AlarcãoJob Van ExelVioleta KeršulienėJoca ZurcVioleta Mihaela DincăJochanan VeerbeekVioleta MugicaJochen AlbrechtVioleta PopoviciJochen D. SchipkeVioleta RadulescuJo-David FineVioleta SimionJodeh ShehdehVioleta T. Pardío-SedasJodie AveryVioletta Sokoła-SzewiołaJody DushayVioletta ZającJody K. MaysViorel MihailaJody KreimanViorela-Ligia VaideanJodye SelcoViorica ChirilaJoe MillwardViorica PârvulescuJoe-Air JiangViorica PatruleaJoel Henrique EllwangerViraiyan TeeroovengadumJoel NitzkinViren SwamiJoëlle ProvasiVirendra YadavJoe-Ming LeeVirendrakumar DeonikarJoerg ZumbachVirgile Clergue-DuvalJoeri Jan PenVirgilijus SkulskisJoffrey DrignyVirginia Alcaraz-RodríguezJohan BorgVirginia Barba SánchezJohan CassirameVirginia Carbajosa RodríguezJohan De Vogel-Van Den BoschVirginia CorazziJohan Du PlessisVirginia MarshallJohan G. J. Vande WalleVirginia Navajas-RomeroJohan LambeckVirginia Ramseyer WinterJohan LindqvistVirginia Sánchez RodríguezJohan MolenbroekVirginia Serrano GomezJohan MottelsonVirginia TancrediJohan NgVirginia ValentiniJohan P. M. SandersVirginia ZalazarJohan SvenssonVirginia ZweigenthalJohann ValentowitschVirginie FelizardoJohanna EspinVirginie LibanteJohanna FreidlVirtudes Pérez-JoverJohanna GrafVisasiri TantrakulJohanna Järvinen-TassopoulosVishma Pratap SurJohanna JohannssonVishnu A. AdoleJohanna KostenzerVishnu KhanalJohanna Lisa DegenVishnunarayan Girishan PrabhuJohanna NorderydVishwajeet SinghJohanna TakácsVishwanath PrabhuJohannes BalasVishwanath SankarasubramanianJohannes BellerVishwendra PatelJohannes DreesmanVisitación FernándezJohannes Ernst DrewesVisnja KaticJohannes JägerVissanu MeeyooJohannes LoddersViswanathan ShankarJohannes MüllerViswas Raja SolomonJohannes PernaaVit NovacekJohannes ReichVita JagričJohannes RodriguesVita JuknevicieneJohannes S. P. DoehlVitalii NitsenkoJohannes VölkerVitalii TimofeevJohannes WancataVitalija SimonaitytėJohannes WendscheVitaliy GarginJohn A. CaldwellVitaliy IshchenkoJohn A. D’EliaVitaly PostoevJohn A. St.CyrVithor Rosa FrancoJohn Amson CapitmanVito BiondiJohn B. LasekanVito BobekJohn BabrajVito ChiancaJohn BaldwinVito FioreJohn BarentineVitor Barreto ParavidinoJohn BartkowskiVitor CaldeirinhaJohn BartzisVítor De Sousa GabrielJohn BerketaVitor FerreiraJohn Bertram VincentVítor GonçalvesJohn BlakeVítor João Pereira Domingues MartinhoJohn BrooksVitor ParolaJohn BuckleyVitor RodriguesJohn ButlerVitor RoqueJohn BuxceyVítor SilvaJohn C. QuindryVitor William Batista MartinsJohn CarusoVitrai JózsefJohn Charles RyanVittoradolfo TamboneJohn ClunesVittoria Carnevale PellinoJohn CoulterVittoria CozzaJohn D. BulloughVittorio AprileJohn D. PerryVittorio DibelloJohn DadzieVittorio Emanuele BianchiJohn Dominic SmithVittorio MartoneJohn DowneyVittorio RamellaJohn DubeVittorio SaettoneJohn Edward ThornesVivek AnandJohn F. BlaikleyVivek KumarJohn F. NewmanViveka GuzmanJohn FischettiViveka Lyberg-ÅhlanderJohn FisherVivian De WaardJohn G. PappasVivian R. RamsdenJohn GalVivian ValdmanisJohn GodleskiVivian VimarlundJohn GuentherViviana GiampaoliJohn H. ArmstrongViviana Loria KohenJohn H. FosterViviana MucciJohn HowardViviana OnofreiJohn J. GuersVivien ChavanelleJohn J. SuschakVivienne M. HazzardJohn Jairo AraujoVjekoslav CigrovskiJohn K. McGuireVlad DionisieJohn Kelly SmithVlad RadoiasJohn KerrVlada I. PishchikJohn KratusVlada VitunskienėJohn L. McIntoshVlad-Alexandru MuntianuJohn L. MokiliVladiana TuriJohn LeakeVladimer Lado GamsakhurdiaJohn M. BurnheimerVladimir A. KorshunovJohn MallettVladimir A. PlotnikovJohn McLuskeyVladimir A. ShvartzJohn McNamaraVladimir CvetkovićJohn McNuttVladimir DodevskiJohn MohanVladimir DvoretskyJohn MonroVladimir HobzaJohn Muthini MaingiVladimir KasikJohn N. A. BrownVladimir KenisJohn N. Ng’ombeVladimír KinclJohn Nedson Ng’ombeVladimir M. CvetkovićJohn Ng’ombeVladimir ObolkinJohn O’HaganVladimír RogalewiczJohn OswaldVladimir SobolevJohn OukoVladimir Tale TakšićJohn P. BucciVladimir VukovićJohn PolimeniVladislav SvozilíkJohn R. WeinsteinVladislava GusarJohn RasmussenVlad-Olimpiu ButiurcaJohn RiceVlaho BrailoJohn Richard JenningsVlasios KasapakisJohn RobertsVlastimil ChytrýJohn RolloVlatka GvozdićJohn S. MitchellVo Truong Nhu NgocJohn S. SchieffelinVojkan LazićJohn SadarVojko KanicJohn SaucedaVojtěch HrdličkaJohn SkinnerVojtěch PeřinaJohn T. BatesVolkan ArisanJohn T. SchulzVolker GehrauJohn ThomasVolker HarthJohn W. YuenVolker ZschorlichJohn WaldVolodymyr FalshtynskyiJohn WaterworthVolodymyr ShymanskyiJohn WuVoula GeorgopoulosJohn ZhangVrushali AgasheJohnannes Jacob De SoetVsevolod ScherrerJohnson Chun-Sing CheungVui King Vincent-ChongJojo JiaoVukica JovanovićJojo Yan Yan KwokWachira WongtanasarasinJoko SarwonoWacław AndrusikiewiczJolan NisbetWadad Kathy TannousJolanda LindenbergWade TrappeJolanta AidukaiteWadim StrielkowskiJolanta DąbrowskaWael El-DeebJolanta DadonieneWael SaadeJolanta DzwierzynskaWael SemidaJolanta JoniecWafa DouziJolanta KarakulskaWageh-Sobhy DarwishJolanta KarpowiczWagih Mommtaz GhnnamJolanta KisielewskaWai C. ChongJolanta Klukowska-RötzlerWai Keung KAMJolanta Kostrzewa-JanickaWai Kin LauJolanta LatosińskaWai Ming ToJolanta LewkoWai Phyo AungJolanta MarszalekWai Yew YangJolanta Nawrocka-RutkowskaWai-Hang KwongJolanta RzymowskaWajaht A. ShahJolanta StrzeleckaWajeeh DaherJolanta SzymańskaWakiro SatoJolanta TkaczykWalaa NegmJolanta ZuzdaWaldemar AndrzejewskiJomar RabajanteWaldemar BrolaJon Anthony HyettWaldemar SpychalskiJon B. SuzukiWaldemar ŚwiderskiJon BrazierWaldemar TarczyńskiJon I. ElstadWaleed Hares ShetayaJon IrazustaWalid FakhouriJon M. JacobsWalid M. I. HassanJon Mikel Picabea ArburuWalid MarrouchJon Øyvind OdlandWalid OueslatiJon P. WoodsWalter Alexander Mata-LópezJon ReiersenWalter CastroJon Torres-UndaWalter ColangeliJonas A. PramuditaWalter CurrentiJonas BorellWalter DachagaJonas CrevecoeurWalter F. StewartJonas E. AnderssonWalter FilhoJonas LanderWalter G. DuffyJonatan García CamposWalter GotliebJonathan BayuoWalter HewerJonathan BishopWalter J. FlapperJonathan BroadbentWalter LamJonathan CaudillWalter MarkowitzJonathan D. NeukamWalter R. SchummJonathan DavidWalter SapuppoJonathan DeutschWalter SátyroJonathan DuvallWalter T. De VriesJonathan Frank AntinWalter TheseiraJonathan FryeWalter WenzelJonathan KahlWalton SumnerJonathan KershawWan Hasrulnizzam Wan MahmoodJonathan LecureuxWan MananJonathan MartínWan Zuha HasanJonathan Puente-RiveraWanchun SuJonathan RoundWanchun ZhangJonathan SchaeferWanderson SilvaJonathan WhiteWang HongpingJonathan WillisWang MinJonathon Paul LeiderWang TaoJonathon R. FowlesWangdo KimJone A. StanleyWanghe KunyuanJones Luís SchaeferWang-Kin Oscar ChiuJong Beom LimWangnan CaoJong Cheol ShinWangxiao HeJong In KimWanich SuksatanJongchul ParkWan-Jiun ChenJong-Chul YangWan-Ju Annabelle LeeJongeun LeeWanlin JiangJongho MoonWanlu OuyangJong-Hwa JangWansuk ChoiJong-koo KimWantana SiritaratiwatJong-woon ChoiWantanee PhanprasitJongwoon KimWanyu ChungJong-Wuu WuWaqas BangyalJonhatan SilvaWaranun BuajeebJoni JupestaWarawoot ChuangchaiJonida HaxhiWarren Raymond Lee CairnsJonivar SkullerudWasan KatipJonna OjalammiWaseem El-HuneidiJonna WilénWassan NoriJonnathan Guadalupe Santillán-BenítezWasu Pathom-AreeJoo-hun ParkWatcharapong MitsuwanJoohyung LeeWatson Scott SwailJoon Hyuk ChoiWawrzyniec CzubakJoon W. ShimWayne ThomasJoon Wah MakWayne WarburtonJoon Young LeeWee Meng Eric LeeJoonho MoonWei Boon YapJoop Van Den BerghWei Cheong NgeowJoost BierensWei HsuJoost BuysschaertWei HuaJootae KimWei HuangJooyeon HwangWei JiangJoo-Young OheWei LangJordan G. BarrWei LiJordan GibbsWei LiangJordan HalseyWei LinJordan PierceWei LiuJordana GeorginWei LyuJordi Arboix-AlióWei NiJordi CastellvíWei PanJordi ColomerFeliuWei ShaJordi Franch-ParellaWei Shin LeongJordi PortaWei SongJorg BahmWei SunJörg D. HoheiselWei Tong ChenJörg GerkeWei WangJörg HaierWei WeiJörg NiemannWei XingJorge Alberto PereiraWei XueJorge Alfaro-PérezWei ZhaiJorge AlonsoWei ZhangJorge AredeWei-Biao LiaoJorge BonitoWei-Chang HuangJorge Calvillo-ArbizuWeiche HuangJorge Casaña-MohedoWeicheng LuoJorge Cerqueiro-PequeñoWei-Chieh HuangJorge Diogo Da SilvaWei-Chih ChenJorge E. MoraisWei-Chun LinJorge Eduardo Martínez PérezWei-Chun TsengJorge Esquiche LéonWeici WangJorge Expósito LópezWeicong FuJorge Fernandez HerreroWeidan CaoJorge Fernandez ParraWeifang ZhangJorge Ferraz de AbreuWeihai XuJorge Gallardo-CamachoWeiheng XuJorge García-VillanuevaWei-Hong ZhangJorge GatoWei-Hsiang ChangJorge GonçalvesWei-Hsin SunJorge Góngora-RodríguezWei-hsiung YangJorge Herrera-TapiaWeilian LiJorge Humberto MartinsWei-Ling HsuJorge JuliaoWei-Min ChangJorge Limon-RomeroWei-Min ChenJorge LizandraWei-Min ChuJorge Lopes Cavalcante NetoWeiran ShanJorge López-FernándezWeisheng ChiuJorge López-GarcíaWeishi ZhangJorge Luis Zambrano MartinezWei-Shuo LoJorge Manuel Leitão FerreiraWeisi LiuJorge Mendez-AstudilloWeiwei ChenJorge Mongil-MansoWeiwei QiJorge Moya HiguerasWeiwei WangJorge Murillo-GonzálezWeiwei ZhengJorge N. R. MartinsWeiwen ZhangJorge O. MorenoWeixiang HuangJorge PérezWeixin LiJorge Pérez-GómezWeiye XiaoJorge PetroWei-Yi WuJorge R. SanchezWeizhen DongJorge RevezWeizhen HouJorge Ricardo Mejía-SalazarWells UtembeJorge Rodolfo BeingoleaWen LiuJorge Rodríguez-ArceWen Lun YuanJorge Rojo RamosWen Tzu TangJorge Salas HerranzWen WangJorge T. RibeiroWen WuJorge TrillerasWen ZhouJorge UmbelinoWenbin LiangJorge Velázquez-SaornilWenbing ZhaoJorge Vergara-MoralesWenbo CaiJorgedel Rosario Fernández SantosWenbo ZhuJorge-Manuel DueñasWenceslao PeñateJørgen Gustav BramnessWenchen HuangJorine NdoroWenchen WuJoris Van OuytselWen-Cheng LinJorma SormunenWencheng LiuJörn HeerenklageWen-Chun LiaoJörn TheuerkaufWen-Chung LeeJos H. T. RohlingWendee WechsbergJos M. B. M. Van Der VossenWendi WeimarJos TwiskWendy A. BeauvaisJose A. BetancourtWendy BonythonJosé A. Cano BuendíaWendy J. O’BrienJosé A. CernudaWendy Lynne GilleardJosé A. OrosaWendy M. WalwynJosé AfonsoWendy WuytsJose Agea ZafraWenfei ZhuJosé Alberto Batista RodríguezWeng Siew LamJose Alberto Gallegos-InfanteWengang HuJosé Alberto Gil CoriscoWeng-Kun LiuJosé Alberto Herrera-MeliánWen-Hsi ChengJosé Alberto Quitério FigueiredoWenhsiang ChiuJosé Alberto Ribeiro-GonçalvesWen-Hsien TsaiJose Alejandro Galaviz-AguilarWen-Hsu LinJose Alejandro Martinez-IbarraWenhui JiangJosé Alexandre ReisWenhui KuangJosé AlvarelhãoWenhui LiJosé Álvarez-GarcíaWenhui MaoJose Alves-GuerreiroWenjia WangJosé Angel AmorósWenjie LiJose Ángel Martínez-LópezWenjie RenJose Antonio Albaladejo-GarcíaWenjie WanJosé António Baptista Machado SoaresWenjing JiJosé António C. SantosWenjing YangJose Antonio CamuñezWenjuan CongJosé Antonio CasasWenjun JiaoJose Antonio de PazWen-Li HouJosé António Ferreira PorfírioWenliang SongJosé Antonio Garrote-AdradosWenlong ZhangJose Antonio Hurtado-SánchezWen-Ni HuangJosé Antonio LlosaWenping LiuJosé Antonio Marín-MarínWenqing WangJosé Antônio Menezes FilhoWenrui HaoJosé Antonio MingoranceWen-Sheng LinJosé Antonio Mirón-CaneloWentai LuoJosé Antonio Morales-GonzálezWentao DongJosé Antonio MorañoWentao FengJosé Antonio OrtegaWentao JiaoJosé Antonio Picó-MonllorWenting ChengJosé Antonio Ponce-BlandónWen-Wei Robert HsuJosé Antonio RodríguezWenxin HuaiJosé Antonio Vigara ZafraWenxin JiJose BaezaWenxue LinJosé Belda MedinaWenyen JuanJosé Benito Bouza-RodríguezWenying LuJosé BragadaWenyou HuJosé Bruno Nunes SilvaWenzhen CaoJosé Carlos CarvalhoWenzhen GeJosé Carlos Dias RoucoWenzhou YuJosé Carlos LazaroWenzhuo LiJosé Carlos Lopez-ArdaoWeon-Hee MoonJosé Carlos MoraisWerner BengerJosé Carlos Núñez PérezWerner CordierJose Carlos Piñar-FuentesWerner HofmannJosé Carlos RodríguezWeronika Korpysa-DzirbaJosé Carlos SáWeronika Martyna UrbańskaJosé Carlos Vázquez-ParraWesam H. BeitelmalJosé Carmelo Adsuar SalaWeslania Viviane NascimentoJosé D. UrchagaWesley KephartJosé Daniel Jiménez GarcíaWesley O’BrienJosé Duarte SantosWesley T. HoneycuttJosé Eduardo TeixeiraWhitney A. BullockJosé Egídio Barbosa OliveiraWieland ElgerJosé Enrique Moral-GarcíaWieslaw FialkiewiczJose Enrique Torres VaamondeWiesław FideckiJose Escribano MaciasWiesław GądekJosé Eugenio Rodríguez FernándezWiesław PrzybylskiJosé F. Gómez-ClavelWieslaw ZiolkowskiJosé Felipe Jiménez-GuerreroWiktor HaleckiJose Fernández-SáezWiktor JedrzejczakJose Fernando ValenciaWiktoria StaśkiewiczJosé Fortes LopesWiktoria WojniczJose Francisco Benavente CuevasWilco ZijlmansJose Francisco Gomez-SosaWiley BartonJosé Francisco IslasWilfred WalshJosé Francisco López-GilWilfredo De Jesús RojasJosé Francisco Rodríguez-VázquezWilfried CoolsJosé Garcia-ArroyoWill EvansJose Geraldo MillWill McConnellJosé Gilberto Dalfré FilhoWillard MunyokaJosé Gutiérrez-PérezWillem StockJosé Hernández OrtegaWillem VlakveldJose I. BaileWilliam B. GrantJosé Ignacio Calvo ArenillasWilliam B. MillerJose Ignacio Lijarcio CarcelWilliam CrownJosé Ignacio Nazif-MunozWilliam DonohueJose Irivaldo Alves Oliveira SilvaWilliam Douglas EvansJosé J. Castro-TorresWilliam E. AckermanJose J. Gil-CosanoWilliam FlackJosé J. Martínez-MagañaWilliam FungJose Jair Alves Mendes JuniorWilliam HammondJosé Javier Navarro PérezWilliam Hongsong WangJosé Joaquín MiraWilliam JangJose Joaquin Ramos-MirasWilliam Ka Shing CheungJosé Juan Carrión MartinezWilliam M. ByneJosé Juan Pereyra-RodríguezWilliam M. MeilJose L. García-SoidánWilliam MichelonJosé L. MartinhoWilliam NketsiaJosé Laerte BoechatWilliam P. BaumgarthJosé López-CastroWilliam P. BergJosé Luis Clúa EspunyWilliam PitmanJosé Luis Cueto AncelaWilliam PotterJosé Luis Hernández-VerdejoWilliam R. BerryJosé Luis Martín-ContyWilliam R. MarchandJosé Luís Ortega MartínWilliam R. ReedJosé Luis Paniza PradosWilliam RassmanJosé Luis Pastrana BrinconesWilliam RileyJose Luis Pérez-CastrillónWilliam Stephen MortonJose Luis Ramos-SanchezWilliam SullivanJosé Luis Rodríguez GutiérrezWilliam YoungJosé Luis Romero BéjarWilliam Yu Chung WangJosé Luis Sánchez-SánchezWilliams JaneJosé Luis SanzWillie PeijnenburgJosé Luis Sarasola Sánchez-SerranoWillie TanJosé Luis Ubago-JiménezWilly HauzerJosé Luis Vázquez-BurgueteWilma Maria Coelho AraújoJose Luis Vilas VilelaWilmer Carvache-FrancoJose M. GarcíaWilson K. GichuhiJosé M. Garrido-MolinaWilson Miguel Salas-PicónJose M. León-PérezWim KrijnenJosé M. Palacios-JaraquemadaWinfried AmoakuJose M. RamirezWinifred Chinyere UkohaJose M. Rodriguez FerrerWinifred E. NewmanJosé Manuel Almerich-SillaWinnie L. S. ChengJosé Manuel Barragán CasasWiola WeredaJosé Manuel Granada-LópezWioleta KrupowiczJosé Manuel Jaramillo OrtizWioletta BielJose Manuel Jimenez-OlmedoWioletta KilarJosé Manuel López GarciaWioletta Mędrzycka-DąbrowskaJosé Manuel MendesWioletta MikulakovaJosé Manuel Moreno VillaresWioletta PawlukowskaJosé Manuel Ortiz-MarcosWiraporn PothisiriJosé Manuel Otero-LópezWireen Leila DatorJose Manuel Saiz-AlvarezWirginia TomczakJosé Manuel Santos JaénWiriya MahikulJosé Manuel Tomás MiguelWisam Eldin MahammedJose Manuel Vilaca Maio AlvesWisnu AdihartonoJosé María Augusto-LandaWissam FarhatJosé María Biedma FerrerWissem GallalaJosé Maria Cancela CarralWitold Jan WardalJosé Maria F.J. Da SilveiraWitold ŚmigielskiJosé María FernándezWitold UhrynowskiJosé María Fernández-BataneroWitold WardalJosé María López GullónWitsanu SaisornJose Maria Lopez-DiazWiwandari HandayaniJosé Martín GamonalesWiwat ChancharoenthanaJosé MendesWladimir Bocca Vieira De Rezende PintoJosé Miguel Mansilla DomínguezWladimir Rafael BeckJosé Moleiro MartinsWłodzimierz A. WójcikJosé Montoya-BelmonteWłodzimierz KołodziejczakJose Navarro PedreñoWłodzimierz RembiszJosé NevesWojciech BednarzJosé Pablo Lara-ÁvilaWojciech BierzaJosé Paulo LousadoWojciech CzerskiJosé Pedro MartinhoWojciech DąbrowskiJosé Pérez-AlonsoWojciech DrygasJose PreciosoWojciech EliaszJose R. AlamedaWojciech GlinkowskiJosé Raduan Jaber MohamadWojciech J. CynarskiJosé Rafael De AlmeidaWojciech JachećJosé Ramón Calvo-FerrerWojciech Jerzy PietrońJosé Ramon Lillo-BeviáWojciech KiebzakJose RangelWojciech KieratJosé Roberto Castilho PiqueiraWojciech KisiałaJosé Rodrigo Da SilvaWojciech KochJosé Salgado RodriguesWojciech MaciejowskiJosé SilvaWojciech PaslawskiJosé Somoza MedinaWojciech SmułekJosé Tomas Mateos GarcíaWojciech SrokaJose Torres-PruñonosaWojciech Stefan ZgliczyńskiJose Ueleres BragaWojciech StykJosé V. PestanaWojciech TrzebińskiJose VaronaWojciech WalatJosé Verdú-SorianoWojciech WitkowskiJosé Vicente Andrés PeiróWojciech ZgłobickiJose ZariffaWolfgang BaumannJosé-Antonio Giménez-CostaWolfgang DürJose-Benito Rosales ChavezWolfgang LeisterJosef BotlikWolfgang LentzJosef KřečekWolfgang RascherJosef SmolleWolfgang SchoppekJosef ZelenkaWolfgang UterJosefa Gonzalez-SantosWon JungJosefin BoschWon LeeJosefin Thorslund ErikssonWon Seok LeeJosefine AtzendorfWon Sop ShinJosé-Jesús Jimenez-RejanoWon-Bae ParkJosélia FonsecaWonchung LimJose-Luis Montes-BotellaWondimagegn MengistJose-Manuel Guaita-MartinezWongkot WongsapaiJosé-María FigueredoWonho YangJosé-María JiménezWonjae JeonJosé-María León-RubioWon-Ju LeeJosemberg Moura De AndradeWon-Ju ParkJosep Arnabat-DomínguezWonsae LeeJosep Carles Benítez-MartínezWon-Sang JungJosep GalceranWonsuk AnJosep Maria BorràsWoo-Baek ChungJosep Maria Ramon TorrellWoochun JunJosep PetchaméWoo-Hwi YangJoseph A. AdeyemiWoohyun YooJoseph A. Di VerdiWoojin KangJoseph A. RocheWoojoo LeeJoseph Aduse-OpokuWook Jin ChoiJoseph AttiasWoon-Man KungJoseph BarnetWoorim KimJoseph BaumgardenWoraphon YamakaJoseph C. KidneyWorapol Alex PongpechJoseph Calvin GagnonWorasak KlongthongJoseph CuthbertsonWorawan PanpipatJoseph HollerWorku AbebeJoseph John KochmanskiWorradorn PhairuangJoseph KvedarWouter VanderplasschenJoseph L. SáenzWubin XieJoseph L. ServadioWubshet TesfayeJoseph M. PlasekWui-Chiang LeeJoseph MaroonWullianallur RaghupathiJoseph McIntyreWycliffe W. Simiyu NjororaiJoseph MolnarWysocka IzabelaJoseph MukuniXabier RíoJoseph NassifXavier Alvarez-SubielaJoseph Olusesan FadareXavier BarazaJoseph OrisaleyeXavier C.C. FungJoseph PierreXavier StruillouJoseph PintoXavier ThuruJoseph R. El-KhouryXavier VallèsJoseph RobertsXenia LatypovaJoseph SanfordXesús FeásJoseph SsenyongaXi HuJoseph T. SteensmaXi JiangJoseph UlatowskiXi LeiJosephine ConvertiniXi LiuJosephine RidleyXi PanJosephine S. AuXi YuJosé-Victor RodríguezXia HaoJosh ColstonXia LeeJoshua A. HagenXia PuJoshua BarzilayXia WangJoshua CarrXiamei GuoJoshua H. WestXiang LiJoshua J. PrasadXiang LiuJoshua J. ThomsonXiang LuoJoshua Kiddy K. AsoamoahXiang MaJoshua L. SantarpiaXiang RenJoshua MuscatXiang WuJoshua P. KellerXiangbo XuJoshua Porat-DahlerbruchXiangfeng ZengJoshua R. EdwardsXianghao ZhanJoshua SparksXiangjin ShenJoshy Joseph KarakunnelXiangli ZhaoJosiclêda GalvincioXianglong ZengJosie HenleyXiangmei WuJosip ĆirićXiangpeng LiaoJosip DelmisXiangqun JuJosip KranjčićXiangyang XuJosip MilasXiangyang ZhouJosip TomićXiangyu HouJosipa BukićXianhai ZengJosko BozicXianhong LiJosko MarkicXianhua LiuJoško OsredkarXianliang ZhangJoss GreeneXianwei CheJosue Aaron Lopez LeyvaXianwen WangJosue Ballesteros-AlvarezXiao GuoJosué G. AmiánXiao LiuJosue MartosXiao SuiJosue MbonigabaXiao YangJosué Prieto-PrietoXiao ZhangJosune Rodríguez-NegroXiaobin ZhangJota SamperXiaobing LiJotheeswari KothandaramanXiaobing PangJothi VargheseXiaobing YuJou-Fang DengXiaobo ZhaoJou-Man HuangXiaochen ShenJoung Yoon ChoiXiaochen ZhouJouni RäisänenXiaochuan PanJovan GardasevicXiaochuan SongJovan VukovicXiaodong GeJovana KojićXiaodong GongJovana NikolovXiaodong YangJovana PejovicXiaodong ZhangJovana PetrovićXiaofei HaoJovana TodorovicXiaofei LiJovani Taveira De SouzaXiaoge HanJowy Yi Hoong SeahXiaoguang JiangJoy C. MacDermidXiaoguang ZhangJoy ClancyXiaohe XuJoy HoneaXiaohong LiJoy L. HartXiaohua TengJoy MitraXiaohui HuJoy ParkinsonXiaojiong JiaJoy PerrierXiaojun DingJoyce BierboomsXiaojun LiJoyce OlusholaXiaojun LuJoyce PopoolaXiaojun ShanJoyce TsujiXiaojun WeiJoydeep ChakrabortyXiaojun YouJoyla FurlanoXiaokang WuJozef BudayXiaolei ZhangJozef KubásXiaoli DuanJozef MosiejXiaoliang QiJózef OberXiaoliang WangJožef ŠimenkoXiaolin HeJozef SowinskiXiaolin HouJózef W. KobosXiaolin YangJózefa DąbekXiaoling HuJózsef BanyárXiaoling JinJózsef BognárXiaoling PanJozsef KatonaXiaolu LiuJózsef LennertXiaomeng WangJózsef Márton PucsokXiaonan WangJózsef PiffkóXiaoping XieJu Hee KimXiaoqin WangJuan A. Ortega-GarcíaXiaoqing SongJuan Adolfo Chica RuizXiaoran HuangJuan Alfonso García-RocaXiaoshan GordyJuan Alfredo Jiménez-EguizabalXiaoshan ZhuJuan Algar-PinillaXiaotian GaoJuan Aníbal González-RiveraXiaowei GuoJuan Antonio Díaz-ManchaXiaowen DaiJuan Antonio Párraga MontillaXiaowen HuJuan Antonio Roche CárcelXiaowen JiJuan Antonio Sánchez SáezXiaowen ZhuJuan Antonio Valera-CaleroXiaoyan WangJuan Bautista De SanctisXiaoyang LiuJuan C. Guevara-PérezXiaoyi YangJuan C. Infante-MoroXiaoyong HuJuan Cándido Gómez-GallegoXiaoyu CheJuan Carlos Ángeles-HernándezXiaoyu ChenJuan Carlos CaicedoXiaoyu LanJuan Carlos Checa-OlmosXiaoyu NiuJuan Carlos De La Cruz-MárquezXiaoyu ZhangJuan Carlos Fernández GonzaloXiaoyue NiJuan Carlos López-GarcíaXiaoyun LiJuan Carlos Marzo-CamposXiaying XinJuan Carlos Meléndez RodríguezXiayu FengJuan Carlos Moran AlvarezXichen MouJuan Carlos PomaresXihui ChenJuan Carlos Redondo-CastánXijing WangJuan Carlos Sánchez-GarcíaXilei DaiJuan Carlosdela Cruz-CamposXilin ZhouJuan Castillo-MartinezXiling XiongJuan De Dios Sanchez-LopezXimena ArriagaJuan Diego Ramos-PichardoXimena GarzonJuan Durango CorderoXimena P. VergaraJuan Enrique Gonzálvez-VallésXimena PorcasiJuan EspinozaXimeng ChengJuan Evaristo Callejas JerónimoXin CuiJuan Fernando Cardenas GonzálezXin DengJuan Francisco ColomaXin LiJuan Francisco Rodríguez TestalXin LuoJuan Francisco Velarde-GarciaXin QiJuan García MeilánXin ShiJuan Gavala-GonzálezXin WangJuan Gerardo Reyes-GarcíaXin YeJuan González-MartínezXin ZhaoJuan González-PascualXinan WangJuan Hernández-ÁvilaXinchao LiuJuan Hernández-LougedoXin-Chen HongJuan Jesús García-IglesiasXinchuang ChenJuan José Criado-ÁlvarezXing ZhangJuan José GaglíardinoXingang LiJuan José Gonzalez GerezXingang YaoJuan José Rodríguez MercadoXingcai LiuJuan L. TerrasaXingchao WangJuan LagoXingcheng LuJuan López-NúñezXingcheng YanJuan Luis Delgado-GallegosXinghua LiuJuan Luis Hernández ArellanoXinglei LiuJuan Luis Sánchez GonzálezXinglong JuJuan M. PericàsXingmin ZhangJuan M. Santos GagoXingshun QiJuan M. Serrador PeiróXingwei LiJuan Manuel Carmona-TorresXingyu WuJuan Manuel García-CeberinoXingzhao LiuJuan Manuel Guzmán-FloresXingzhe SongJuan Manuel MachimbarrenaXingzhou TianJuan Manuel Martin AlvarezXinhua GaoJuan Manuel Medina Del RioXinhui WangJuan Manuel MendiveXining YangJuan Manuel Moreno-MansoXinjun LiJuan Manuel Muriel-SánchezXinke WangJuan Manuel NuñezXinqi ZhengJuan Manuel Trujillo TorresXinquan ChengJuan Miguel Martínez GalianoXin-Tang LinJuan Moreno-GutiérrezXinwei YuJuan Mozas-MorenoXinwen BaiJuan OyanedelXinxin FanJuan Pablo ArdilaXinxing WangJuan Pablo DamiánXinyang YuJuan Pablo Hervás PérezXinyi LuJuan Pablo Rey-LópezXinyuan LiangJuan Pedro López-SigueroXinzhong ZhangJuan Pedro Martínez-RamónXiping YangJuan R. CocaXiqiang XiaJuan Rabal-PelayXiuhua DingJuan Rendon SchneirXiuhua WengJuan Roberto Jiménez-PérezXiulian SunJuan Torres-MachoXiuyan ShaoJuan TorviscoXiwei MiJuan Vega-EscañoXiwei ShenJuan VesgaXixi ZhouJuan WangXixiong XuJuan ZhangXizhong ShenJuana Gómez BenitoXosé López-GarcíaJuana Inés Gallego-GómezXu XinJuana Macías SedaXu YangJuana María NavarroXuan LiJuana María Vázquez LaraXuan LiuJuan-Francisco Álvarez-HerreroXuan YuJuanita Romero-DíazXuantong WangJuan-Jose SeguraXuanyi NieJude OkolieXuanyi ZhouJudit BekeXuchu JiangJudit FinánczXue WangJudit HalászXue YangJudit Mendoza AguilarXue ZhangJudit SágiXuebing CaoJudit SulyokXue-Bo JinJudit T. KissXuechen LeiJudita KinkorováXuefei ZhouJudite GonçalvesXuefeng HuJudite VieiraXuefeng LiJudith BeckerXuejiao NiuJudith BruchfeldXuejun QianJudith Cavazos ArroyoXuekui ZhangJudith CorneliusXuelei ZhangJudith DavieXuemei HanJudith LukaszukXuening QinJudith M. ScottXuesong GuoJudith PettsXueting ZengJudith RogersXuexiang HeJudith SixsmithXueyan LiJudith SligoXueying YangJudith T. ZelikoffXufei YangJudy BowenXuguo ZhangJudy HavlicekXuhuiqun ZhangJudyta KabusXuliang ZhuangJue HuangXuming MaoJue WangXunqian LiuJuei-tang ChengXu-Qiao ChenJuergen Heinz SeufertXuqun YouJuha SalmiXuwang ZhangJuha TaavelaXuyin YuanJuhani H. MäättäXuying MaJuhee AhnYachao LiJui-Yang LaiYa-chi LinJukka PappinenYa-Ching ChouJukka UittiYacine JaïdiJulaine AllanYacine OuahchiJulia BujanYacob Mohd RusliJulia Chen-SankeyYacov FogelmanJulia Dickson-GomezYadav UpretyJulia DíezYadollah Abolfathi MomtazJulia DoitchinovaYadong HuangJulia DuranteYael Wolff SagyJulia FöckerYafa LevanonJulia GelattYafei WangJulia GonzálezYaghoub MansourpanahJulia González-VacaYahaya TajudeenJulia Guerrero-GironésYahia Z. HamadaJulia JockuschYahong DongJulia KobulskyYa-huei WangJulia KönigYahui WangJulia LischkaYahya SohrabiJulia MalitsYair BenyaminJulia NeumannYair SchwartzJulia ReifYaiza Taboada-IglesiasJulia RiddellYajian WangJulia SandersYajie ZouJulia Simões Corrêa GalendiYajun YuJulia SommerYakup Emre CoruhluJulia WalshYalçin TepeJulia Wojciechowska-SolisYalcin YildirimJulian FriebelYali FengJulian KünzelYalong LiJulian McDougallYalun TanJulian Nevado BlancoYam LimbuJulian RudischYamakawa MiyaeJulian S. RechbergerYameng LiJulián Solís Garcíadel PozoYamin Tauseef JahangirJulian TangYan HuangJuliana Cristina Dos Santos MonteiroYan MengJuliana F. Filgueiras MeirelesYan MuJuliana FernandesYan ShenJuliana Maria Navia-PelaezYan ShiJuliana Melo Teruel Biagi CamargoYan WangJuliana SáYan ZhangJuliane HeydenreichYana Roka-MoiiaJuliano BortoliniYanan ZhangJuliano GaravagliaYan-Bin HuangJuliano UczayYanbo HuangJulie Aitken SchermerYanchao FengJulie BeneshYaneth Bustos-TerronesJulie CôtéYanfang ZhaoJulie CousinsYang ChenJulie ErnstYang ChengJulie FitnessYang GaoJulie GiustinianiYang GongJulie HaesebaertYang JuJulie HagstrømYang LiJulie HernandezYang LuJulie Lorraine DarbyshireYang WangJulie RageulYang Woon ChungJulie RibaudoYang WuJulie RonnebaumYang XuJulie SchweitzerYang YangJulie TaylorYang YuJulie VaiopoulouYang Yun-jungJulie VrankenYang ZeyangJulieanne P. SeesYang ZhangJulien AnetYang-Ching ChenJulien IssaYangguang LiuJuliet Haarbauer-KrupaYangguo ZhaoJuliet WakefieldYanghoon JungJulieta Domínguez-SoberanesYangping ZhouJulija Cerovic SmolovicYangtian YiJulina Md-NoorYangyi WuJulio AcostaYangzhao SunJulio AlmenaraYanhua FuJulio Augusto Freyre-GonzalezYanhui MaoJúlio Barboza ChiquettoYanina BesstrashnovaJúlio Belo FernandesYaning YiJulio C. Aguila SánchezYanjie ZhangJulio C. De La Torre-MonteroYanjiong ChenJúlio C. LopesYanka KaramalakovaJulio C. Rivera-LeyvaYankho KaimilaJulio Calleja-GonzálezYanli MaJulio César Montañez-HernándezYanling SongJulio Cesar Perez SansalvadorYanmin HeJúlio CostaYannan ZhaoJulio DiazYannick HernandezJulio Parra-FloresYanning ZhaoJulio Penagos-CorzoYannis ParaskevopoulosJulio Plaza-DíazYanping LiuJulio Salcedo-CastroYan-qiong OuyangJulio SternYanqiu YuJulio Vena OyaYanru LyuJulio Vicente Figueroa-MillánYanting WuJulio-César MateusYanuar Farida WismayantiJulita Markiewicz-PatkowskaYanwei LiJulius MangisoniYanxian LinJulius NukpezahYanxin LiuJuliusz PrzysławskiYanyan YuJuliya Aleksandrovna MarakshinaYanyu SongJumadil SaputraYao SongJu-Mee RyooYao SunJumin ParkYao ZhaoJun (Justin) LiYao-Ching HungJun Cong GeYao-Chuen LiJun FengYao-Chung ChengJun H. ChoiYaodong GuJun Ho LeeYaofeng HanJun InoueYaohui LiuJun Jie Benjamin SengYao-Jen HsiaoJun KobayashiYaoping CuiJun KongYaoshan XuJun LiYaping XuJun LiuYaqian XuJun LuYaqian ZhaoJun NagayasuYara SaifiJun QiYari LongobuccoJun RenYaron FinziJun ShenYasamin IzadkhahJun SunYasemin M. AkayJun Sung HongYash GuptaJun SuzurikawaYashavantha VishweshwaraiahJun WangYashon OumaJun WatanabeYashu ChenJun XiaoYashvee DunneramJun YasudaYasin ToparlarJun YoshidaYasir ArafatJun YoshinagaYasir MehmoodJun YuYasir MughalJun ZhaiYasmin JahanJun ZhangYasmin VianaJun ZhaoYasodha RohanachandraJun ZhuYassir A. YassirJunaid RashidYasuharu NakajimaJunaidi JunaidiYasuhiro YamaguchiJunbang WangYasuka NakamuraJunbing HuangYasuki SekiguchiJunbum ShinYasuko FujitaJundi LiuYasunari MatsuzakaJune Andrews HorowitzYasushi ShimadaJune KloubecYasushi SuwazonoJune L. GinYasushige ShinguJung Hua ChouYasutaka KakinokiJung Hun ChoiYasutake TomataJung Hwa LimYasuyuki KonoJung SunwooYavar VafaeeJung-ah ChoiYawen WangJung-Eun ParkYayi ZhaoJung-hee LeeYa-Yun ChengJunghwa ChoiYazed AlsowaidaJung-hwan YoonYazed Saleh AlsowaidaJunghyun ChoiYazeed Yasin GhadiJunghyun LeeYazhen YangJungsuk KangYazmin Hernandez-DiazJungsuk SeoYe Hoon LeeJungtae LeeYe ShenJungtae LeemYebegaeshet T. ZerihunJunguo ShiYe-Chan LeeJungwon MinYee Guan NgJungwoo ChunYehuda D. NeumarkJungyeon SungYehui WangJung-Youn ParkYehya MohamadJun-Ho ChoiYeison Alberto Garcés-GómezJunhong HaoYejin MokJunhong XiaoYelena PopovaJunhui HuangYelena TarasenkoJun-Hyeok KangYenal Dündar‪Junia Nogueira De BritoYen-Cheng ChenJun-ichi KadokawaYen-Chien LeeJunjie LiYen-Kuang LinJunjie ZhangYen-Ming HuangJunko HondaYen-Tzu WuJunkui CuiYen-Ying KungJun-Lin LinYeo Hyung KimJunLong LimYeon Jung YuJunming HuangYeong Jun JuJunqi WangYeongbae ChoeJunrui ChengYeon-Ha KIMJunsik ParkYeonsoo KimJuntao ZhaoYexin ZhouJunxiang ChenYfke P. OngenaJunxuan MaYftach GepnerJunyi HuaYi HuangJu-Pi LiYi Hwa ChoiJuraj ChocholYi LiuJuraj CugYi LuoJuraj JagelcakYi QiJuraj JugYi SongJuraj NemecYi WangJuraj PrejacYi XieJurandir De SouzaYi XuJure MurovecYi ZhangJurema Corrêa Da MotaYi ZhenJuremalani Rameshbhai DharmeshYi ZuoJürgen SeitzYian ZhangJürgen WeberYiannis XenidisJurgita Lazauskaite-ZabielskeYi-Che ShihJurgita NarusyteYi-Chen HsiehJuri TaborriYi-Chen LanJurica FerenčinaYichuan ZhangJurica ŽučkoYichun ChenJurij HanzelYi-Chun ChenJuris BurlakovsYi-Fan WuJuš KšelaYifan ZhangJustin Dean SmithYifan ZhongJustin LawsonYifeng TangJustin LernerYifeng XueJustin MatusYih Yng NgJustin PennerYihang FangJustin R. FeeneyYihang ZhaoJustin VarneyYi-Hsien WangJustina RačaitėYi-Hsin ChenJustine LeavyYi-Hsing ChenJustine N. StefanelliYi-Hung ChiangJusto ArinesYiin Lih-MingJustyna BagińskaYijia ZhangJustyna BrzezichaYi-Ju ChenJustyna BrzezinskaYijuan XuJustyna Chojdak-ŁukasiewiczYijun PanJustyna Dunaj-MałyszkoYijun ZhangJustyna GodawskaYike ShenJustyna GodosYilang TangJustyna Grudziąż-SękowskaYilkal DessieJustyna Jolanta MiszkiewiczYilmaz BayarJustyna KleszczYimeng SongJustyna Kosydar-BochenekYimesker YihunJustyna KowalskaYiming HuoJustyna KujawskaYiming JiJustyna Michalek-KwiecienYiming SuJustyna MiszczykYin LiJustyna ModrzejewskaYin Ping WongJustyna MrózYin Ting CheungJustyna Opydo-SzymaczekYinan JiangJustyna PawlakYinan ZhangJustyna Podgórska-BednarzYin-Fan ChangJustyna RójYing JiangJustyna SikoraYing JieJustyna SteckoYing LiuJustyna SzulcYing XuJustyna TopolskaYing ZhangJustyna WitkowskaYing ZhouJustyna WoźniakYingbin DengJustyna ZamorskaYingdong HeJusuf ZeqiriYing-Guo ZhouJutta G. RichterYingli GuJuve J. CortésYingming YangJuyang XiongYingwei YangJuyeun LeeYingxia LiJu-Yi ChenYingying WangJuzer ShabbirYingying XuJyh-Jeng WuYingying ZhuJyotir Moy ChatterjeeYining JinK. E. SeetharamYinlin JiK. PrasannaYin-Lin WangK. S. AnandhYin-Yee LeongK. S. KasiviswanathanYiqing LiuK.M. AarifYiqing ZhaoKa Chun SiuYit Siew ChinKaan KalkanYitayal Addis AlemayehuKaan OrhanYi-Ting HwangKaboni Whitney GondweYi-Tui ChenKa-Chun ChongYiu Yan LeungKadarkarai MuruganYi-Wen ChiuKah Kheng GohYiwen ShihKahei LuiYixiao JiangKah-Hui WongYi-Yuan LinKahler StoneYi-zhi WangKa-Huen YipYlva MobergKai Cheng HsuYmkje Anna De VriesKai ErenliYo IwataKai GuoYodan RofeKai JonasYogesh NarkhedeKai LeichsenringYogesh SainiKai LiuYogi Tri PrasetyoKai Pong TongYohan NguyenKai W. MüllerYohei InabaKai WangYohei MatsumotoKai YangYohei SawayaKai ZhaoYohei TomiokaKaibao NieYoichi IinoKaigang LiYoichi MorofujiKaija CollinYoji KishiKai-Jen ChuangYoko FukuharaKaijian HouYoko HasegawaKai-Lan ChangYoko KawamuraKailash C. PandeyYolanda ArroyoKailash NarayanYolanda BaezKailash S. GairaYolanda Campos-UscangaKailash SinghYolanda EstrederKailu WangYolanda GarcíaKaiming BiYolanda Marcén-RománKai-Ping HuangYolanda Martinez BeneytoKai-Ren ChenYolanda PastorKairi HayashiYolanda TorresKaisa Leea SunelaYonah Hisbon MatembaKaisa PihlainenYonas BerhanuKaitlin ForsbergYonatal Mesfin TeferaKaitlin RokeYong ChengKaitlyn M. EckYong CuiKaivalya MoluguYong Hui Nee AuKaiway LiYong JiangKaiwen ManYong Jie WongKaiwu HuangYong JinKakuhiro FukaiYong Joo RheeKalaimani ElangoYong QiuKalavathy RajanYong WangKalle KärhäYong Won LeeKalliopi MegariYong XiaoKallyanashis PaulYong YangKálmán ImreYong-Chan HaKalman J. KaplanYongchel AhnKalman RajkaiYongchun LiuKaloyan StoichevYongfa YouKalpana NagpalYongguang YuKalpana NathanYonghe MaKalyan DasYongheng GaoKalyan RameshYonghong BiKalyan SaginalaYonghong MengKalyana ChakravarthyYongliang YanKamal AbdelrahmanYongna YuanKamal BhusalYongqiang ZhouKamalesh ChakravartyYongqing XuKam-Chau WuYong-Seok JeeKamen KanevYongsheng ChenKameron Y. SuginoYong-sheng QianKameryn DenaroYongsu YoonKamil BarańskiYong-Suk KimKamil GillYongwon MoKamil J. WronaYongyong SongKamil KaminskiYongzhao ShaoKamil KoszelaYongzhu XiongKamil Krzysztof RomanYoo Mi JeongKamil KrzyżanowskiYoon Jung JangKamil MichalikYoon Jung LeeKamil NelkeYoona KimKamil P. JaneczekYoonah JeongKamil Reza KhondakarYoonju LeeKamil SierżantYoonjung KimKamil WitaszekYoonsuk LeeKamil ZaworskiYoonsung JungKamil ŻyłaYoshifumi IkedaKamila Czepczor-BernatYoshiharu FukudaKamila Litwic-KaminskaYoshiharu MotooKamila Maliszewska-OlejniczakYoshihiko FurutaKamila MazurYoshihiko KadoyaKamila Pokorska-NiewiadaYoshihiko NaitoKamila R. IrvineYoshihiro FukumotoKamila RachubińskaYoshihiro SuzukiKamila RadlinskaYoshihisa HirakawaKamila Rybczyńska-TkaczykYoshihisa SugimuraKamila SzumilasYoshija WalterKamila Widziewicz-RzońcaYoshika SekineKamila Zglejc-WaszakYoshiki B. KurataKamila Ziółkowska-WeissYoshimitsu FujiiKamilė TaujanskaitėYoshinori MoriyamaKamilla Bargiel-MatusiewiczYoshiro HoraiKamilla PawłowskaYoshitaka NakayamaKamming MokYoshitaka SuzukiKamonwon IenghongYoshiteru MorioKamran IqbalYoshiumi KohnoKamyar MehranzamirYosra A. HelmyKan SaitoYosra CherniKan ZhouYosuke TomitaKanchan MarcusYou WuKandaswamy EswarYoucai ZhaoKang Sup KimYou-Cheng ShenKang WangYoufa LiuKang-Di LuYoufeng ZhuKangil ChoeYouhei SaitoKang-Ming ChangYouhong GuoKangwei LiYouko TakahatakeKanika MalikYounes ShekarianKanike Raghavendra KumarYoung Eun LeeKankan ZhangYoung Hye ChoKannadasan RajuYoung Jae KimKannikar Hannah WechkunanukulYoung Kyun KimKanokporn PinyopornpanishYoung Min JoKanthee AnantapongYoung Pyo JeonKaoruko SeinoYoung Ran ChaeKaoruko ShimizuYoung Woo SongKaowen Grace ChangYoung YiKapil Kiran AedmaYoungEun SongKapo WongYounghan YoonKara B. AndersonYounghee ParkKara P. WisemanYoungjo LeeKaran SinghYoung-joo AhnKarel D. CapekYoungjun ChoiKarel JandaYoung-Ki KimKarel MackůYoungshin SongKarel StaněkYoungsook BaeKarel SýkoraYoungtaek ParkKarel Van Den BoschYoungwon NamKarem A. CourtYoungwoong SongKaren AlexanderYoun-Hee ChoiKaren ChampenoisYounjae OhKaren Chapman-NovakofskiYousef AlthobaitiKaren FisherYousef FarhaouiKaren Fowler-WattYousif SubhiKaren H. MorinYoussoufa M. OusseineKaren HardeeYouyoun MoonKaren HawkeYu Ait BamaiKaren Hazell RaineYu GaoKaren HerbstYu GongKaren Heslop-MarshallYu HanKaren JakubowskiYu IshibashiKaren Kar Loen ChanYu KoyamaKaren KierYu LiKaren KinderYu Lun HsuKaren KopciukYu MengKaren KrugerYu QiaoKaren LillerYu SolovievaKaren LindsayYu SongKaren M. KedrowskiYu SunKaren MartinYu TaniguchiKaren MullinsYu XiaKaren Regina CastelliYu XiaoKaren Schelleman-OffermansYu YaoKaren SutherlandYu ZhangKaren ThorpeYu. S. SidorovaKaren Villar ZarraYuan GuKaren Walker-BoneYuan LiKari Bjerke Batt-RawdenYuan MengKari LippertYuan SuKari StoneYuan TianKari WeberYuan WangKarice HyunYuan XuKarien LabuschagneYuan YuanKarim DorghamYuan ZhouKarim TanjiYuane JiaKarime Chahuán-JiménezYuanmay ChangKarin AhlinYuan-sheng TzengKarin CadwellYuantao FangKarin HugeliusYuanyuan ShiKarin Louise Lenz DunkerYuan-Yuan WangKarin MagnussenYuanyuan YangKarin MandlYuanyuan ZhuKarin MatkoYuanzheng ZhaiKarin MeissnerYuba GautamKarin PetriniYubbu PutriKarin ŞeşetyanYubiao SunKarina AllenYubin LiKarina BatistaYu-Bing WangKarina CichaYubo HouKarine CharryYucheng LiuKarissa PeyerYu-Cheng WangKarl AndriessenYu-Chi LeeKarl BlanchetYuchi ZhangKarl HeleYu-Chieh HuangKarl HusaYu-Ching ChouKarl KingsleyYu-Chu HuangKarl KozlowskiYue CaoKarla A. RomeroYue ChenKarla BitencourthYue WangKarla De JesusYue WuKarla Dhungana SainjuYue ZhangKarla G. CedanoYuebing SunKarla Juarez-MorenoYue-Cune ChangKarla Paola García PelagioYuefei ZhuoKarla PassalacquaYueh-Juen HwuKarla Peraza-JiménezYuehong ChenKarl-Anton HillerYuepeng SunKarl-Arne JohannessenYueqi YanKarli June CerankowskiYueru XuKarlijn ThoonenYuewei LiuKarmaker Shamal ChandraYufei CuiKarmen LončarekYufei ShiKarol BandurskiYufen RenKarol BotYufeng ChenKarol BulskiYuhao WuKarol KarasiewiczYuhao ZhaoKarol KonaszewskiYuh-Chiang ShenKarol PilisYuhei MatsudaKarol PlesińskiYuhei OtobeKarol RycerzYuhki SatoKarolina Bajdak-RusinekYu-Horng ChenKarolina BarcauskaiteYuh-Shan HoKarolina BrylYu-Hsiang ChengKarolina Chilicka-HebelYu-Hsuan LinKarolina CzerwiecYu-Huei HuangKarolina Dudzic-GyurkovichYuh-Yuh LiKarolina Eszter KovácsYuichi AdachiKarolina HoffmannYuichi MurakiKarolina JasińskaYuichi MushimotoKarolina KosaYuichi SaitoKarolina KossakowskaYuichiro HatanoKarolina KostorzYuichiro OtsukaKarolina KotYuiko NagamineKarolina Krupa-KotaraYuji MatsudaKarolina Lewandowska-GwardaYuji NagatomoKarolina MaslakYuji ShimizuKarolina Matej-ŁukowiczYuji TaniKarolina OsowieckaYujie SuKarolina OszustYujin ParkKarolina PawlakYujun SongKarolina PuławskaYu-Jung LiuKarolina SierońYuk Shing ChengKarolina SobczykYuk Ting Hester ChowKarolina SzulcYuka KotozakiKarolina Szymaniec-MlickaYuka SumiKarolina TalarYu-Kai LinKarolina WieszczyckaYuki HanaiKarolina WiszumirskaYuki HitomiKarolina Wojtunik-KuleszaYuki IchisugiKaroline ReichYuki Ishikawa-IshiwataKarthik H. ShankarYuki KataokaKarthik SomasundaramYuki NagamatsuKarthikeyan SubbarayanYuki SeidlerKartik DhadukYuki ShiratoriKaruna DixitYukiko NishihamaKaryn J. RobertsYukio SekiKarzan IsmaelYukio TsugihashiKasemsan ManomaiphiboonYukitaka OhashiKasi VegesanaYuk-Kwan ChenKasidid RuksakietYuko OtakeKasper GórnyYuko YamaguchiKasper RosingYuksel PekerKasper SalinYukuang GuoKasper SipowiczYu-Kuei TengKassapa EllepolaYukun ShiKassian T.T. AmeshoYulei QianKátai-Urbán LajosYuli FradkinKatalin HegedűsYulia GendlerKatalin PappYulia MochalovaKatalin PetőYulia O. BobrovaKatalin VárnagyYulia Yu BozhkoKatariina Ala-RämiYulian KonechnyiKatarina Beljić IvanovićYuliang SunKatarina LalicYuliang ZhangKatarína Lestyánszka ŠkůrkováYuliia TrachKatarina MiloševićYuling WangKatarína ProcházkováYuling XieKatarina RoguljYuliya I. PyzhevaKatarina SmatanovaYuliya ShtaltovnaKatarina SmiljanićYulong ZhangKatarína TeplickáYu-Lung HsuKatarína ValčováYulya TushnovaKatarína VilinováYu-lyu YehKatarzyna Adamczewska-SowińskaYuma YokoiKatarzyna Anna DylagYuman LiKatarzyna BanachYumi YasuokaKatarzyna BroczekYu-Ming FeiKatarzyna BułkowskaYun HanKatarzyna Buńkowska-GawlikYun LiKatarzyna CampbellYun LiuKatarzyna CurzytekYun ShimKatarzyna Dybka-StępieńYun TongKatarzyna FischerYun ZhouKatarzyna GdowskaYuncai WangKatarzyna GodyńYun-Chen ChangKatarzyna GoralczykYunchen YangKatarzyna GrocholewiczYunduk JeongKatarzyna GrondysYuneng DuKatarzyna GrzelaYunfei LiKatarzyna HapYung YauKatarzyna HojanYung-Ching WengKatarzyna HolcmanYung-Kai LinKatarzyna IgnatowiczYungsheng ChenKatarzyna JAchYung-Tsan JouKatarzyna Janik-SupersonYunias SetiawatiKatarzyna Kajak-SiemaszkoYuning HuKatarzyna KodziszewskaYunja YooKatarzyna Kosinska-KaczynskaYunjia ChiKatarzyna KubiakYun-Ke ChangKatarzyna Kwiecień-JaguśYunle ChenKatarzyna LachowiczYunli BaiKatarzyna Leśniewska-NapierałaYunling HeKatarzyna Leszczyńska-SejdaYunlong PengKatarzyna LewtakYun-Shiuan ChuangKatarzyna Mańka-MalaraYunsun JeongKatarzyna Marciniak-ŁukasiakYunuen Socorro Rangel-LedezmaKatarzyna MarkiewiczYun-Yi YangKatarzyna Miszta-KrukYupeng LiKatarzyna Mocny-PachońskaYupeng LiuKatarzyna MorkaYuqi JinKatarzyna NaylorYuri G. GordienkoKatarzyna Nowakowska-LipiecYuridiana Rocio Galindo-LunaKatarzyna Ogrodzka-CiechanowiczYuriko HamadaKatarzyna Okulicz-KozarynYuriko IkedaKatarzyna PactwaYuriy L. Vasil’evKatarzyna PankiewiczYuriy S. BronshteynKatarzyna PawęskaYuriy SlyvkaKatarzyna PawlikowskaYuriy Vasil’evKatarzyna Pietrucha-UrbanikYury KapelyushinKatarzyna Piwowar-SulejYury LoikaKatarzyna Plagens-RotmanYuryi KlapkivKatarzyna PotyrałaYu-Sheng ShenKatarzyna PrzybyłaYusheng ShiKatarzyna Pukowiec-KurdaYu-Sheng YangKatarzyna RolfYushin KimKatarzyna Rydel-CiszekYusriadi YusriadiKatarzyna Sienkiewicz-MałyjurekYusuf SayedKatarzyna SkrypnikYusuf SermetKatarzyna SnarskaYusuf SoyluKatarzyna StankiewiczYusuf WibisonoKatarzyna StasiukYusuke EndoKatarzyna SutkowskaYusuke KajiwaraKatarzyna SygitYusuke KatayamaKatarzyna SzałapataYusuke MaedaKatarzyna SzalonkaYusuke MurataKatarzyna SzepietowskaYusuke NishimuraKatarzyna SzymczykYusuke OhsakiKatarzyna TarnowskaYusuke OsawaKatarzyna TomaszekYusuke UenoKatarzyna TomaszewskaYusuke YamadaKatarzyna TurońYuta SuzukiKatarzyna UmiejewskaYuta TerabeKatarzyna Utnik-BanaśYuta TsubouchiKatarzyna Walicka-CuprysYutaka InoueKatarzyna WalkiewiczYutaka MifuneKatarzyna Wesołowska-GórniakYutaka OkabeKatarzyna WiatrowskaYutaka OwariKatarzyna Wojciechowska-DurczynskaYutaka WatanabeKatarzyna WolnickaYutaka WatanobeKatarzyna Wolny-KoładkaYutian PangKatarzyna ZawadaYu-Tien HsuKatarzyna ZdanowiczYuting ChenKatarzyna ŻmijaYutong DongKatarzyna ZorenaYuval KriegerKate AndersonYuvarat NgernyenKate BeardYuwei FanKate BrownYuwei WangKate BurrowsYuwu JiangKate CheneyYuxi ZhangKate DaviesYuxia MaKate De MedeirosYuya KashiwazakiKate E. MurrayYuyan WangKate FrazerYuyang WeiKate FreireYuye TanKate GuastaferroYuyin ZhouKate GunnYuyuan ZhangKate JudithYuyue QinKate K. ChappellYuzhe LiuKate KingsburyYuzhi XiKate KlootYuzuru IsodaKate M. AnnunziatoYvan DutilKate McBrideYves CouturierKate Schwartzkopf-PhiferYves JacksonKate SherryYvonne Adu-AgyeiwaahKate TudorYvonne DimitropoulosKatelyn F. RommYvonne EfeberaKatelyn RommYvonne GörlichKateřina BerkováYvonne TeuschlKaterina BougiatiotiYvonne ThaiKaterina GeorgantaYvonne van der MeerKateřina IvanováZacarías Sánchez MiláKateřina JančaříkováZaccheus AhonleKateřina KaňkováZacharias PapadakisKaterina KatsarakiZachary K. WinkelmannKaterina KavalidouZachary M. GillenKaterina KotzampassiZachary PrudowskyKaterina NikolitchZaffar Ahmed ShaikhKaterina PapadimitriouZaharah Binti HussinKateřina PodolskáZaheer AhmedKaterina TzafilkouZahid AsgharKaterina-Marina PilalaZahid ButtKateryna KolesnikovaZahid YousafKateryna Lysenko-RybaZahoor Ahmed SoomroKath GodberZahra BagheriKatharina BeyerZahra BeyzaeiKatharina DiehlZahra FarahnakKatharina HametnerZahra HooshyariKatharina HüfnerZahra MojtahediKatharina K. RallZahra PanjaliKatharina KusejkoZahra Shams EsfandabadiKatharina OrnsteinZaid YounesKatharina SchimmelZaiga Nora-KrukleKatherene AnguahZaihua DuanKatherine A. BirchenallZaijun LiKatherine A. KohZaila OliveiraKatherine AppletonZain Anwer MemonKatherine BrookfieldZainab Ahmed AlkaissiKatherine Chiu Man LeungZakariya AlgamalKatherine ConigraveZaki HakamiKatherine Gajardo EspinozaZala KolencKatherine H. IngramZalba SaraKatherine H. LeithZalina Abu ZaidKatherine J. BarrettZanda RubeneKatherine JacksonZander VenterKatherine Ka Pik ChangZane Vincēviča-GaileKatherine M. BoydellŻaneta Anna MierzejewskaKatherine OgurtsovaŽaneta GerhátováKatherine Ott WalterZaneta StaszakKatherine P. AdamsZar Chi SoeKatherine PeelerZargari Marandi RamtinKatherine S. SalamonZarin ZainulKatherine SandersŽarko KevrešanKatherine SwardZbârcea Cristina ElenaKathi R. TrawverZbigniew BujakKathleen CarterZbigniew IzdebskiKathleen CooneyZbigniew JastrzębskiKathleen DavisZbigniew KamockiKathleen FarrandZbigniew MakiełaKathleen KerrZbigniew PopekKathleen KevanyZbigniew RespondekKathleen LeemansZbigniew WisniewskiKathleen MonahanZbigniew ZiembikKathleen PishasZbigniew ZyliczKathrin EberharterZbynek JanourKathrin KohakeZbyněk StraňákKathrine Jáuregui-RenaudZbysław DobrowolskiKathrine WrightZdenko DezelakKathryn A. McFarlaneZeba FarooquiKathryn C. EdwardsZebensui Morales-ReyesKathryn GreeneZeenat Kotval-KaramchandaniKathryn L. HavensZeeshan HamidKathryn LeyvaZeeshan UllahKathryn M. JandaZehra EdisKathryn ShowalterZeinab AliyasKathryn ThorburnZeinab HazbaviKathryn UnderwoodZekai HeKathy BeltonZeki Atıl BulutKathy HibbertZélia MuggliKathy JacksonZeljka LoncaricKathy WiningsZeljka MinicKatia A. Figueroa-RodríguezŽeljka TrumbićKatia ComteZeljko DespotovicKatia Delrahim HowlettŽeljko PavićKatia M. Costa-BlackŽeljko StevićKatia PinelloZemede M. NigatuKatia Regina SilvaZena MelloKatia SidaliZengchuan DongKatie CrowleyZengliang RuanKatie Fitton DaviesZengmiao WangKatie Louise BarfootZeng-Yei HseuKatie M. HeinrichZengzeng FanKatie M. VinopalZenon FoltynowiczKatie McGibbonZenon GajdzicaKatie N. BrownZenon NogalskiKatja CrnogajZenon WisniewskiKatja I. BraamZesergio MeloKatja KröllerZetian ZhangKatja MohorcicZeynep Alpay SavasanKatja ParschatZezhen ChengKatja PynnönenZezhong TianKatja SchrøderZhan LiKatri JalavaZhang YuKatrien De WildeZhanna MingalevaKatrin CunitzZhanqiang ZhuKatrin SchmittZhao GuozhenKatrina Bell McDonaldZhao RenKatrina BrowneZhao YangKatrina LambertZhao YunKatrina McLaughlinZhaobin SunKatrina RadfordZhaohui SuKatsuhiro ItoZhaohui WangKatsuki TsuchiyamaZhaolong YuKatsuma HayashiZhaorong LiuKatsuo OshimaZhaowei KongKaty CampbellZhaoxiong WanKaty GreenlandZhaoyang DingKausar Sadia FakhruddinZhaoyun YinKaushik PaulZhe FengKaushik SridharZhe HanKaustuv BhattacharyaZhe LiuKaustuv MukherjeeZhemeng WuKavalipurapu Venkata TejaZhen QuKaveesha WijesingheZhen WangKavita BatraZhen XuKavitha HaldoraiZhen’an YangKavitha S. RaoZhenbang MaKawee SrikulkitZhenbo LuKawoun SeoZhenbo WangKay L. EscuyerZhenchun YangKay OwenZheng LiKayode Adesina AdegokeZheng Zachory WeiKayode Kolawole EluwoleZheng ZhaoKayvan AghabaykZhengdong ZhaoKazi Md. Amran HossainZhenghao DingKazım KöseZhenglu WangKazimierz KarolewskiZhengning PuKazimierz KuliczkowskiZhengqiu FanKazuaki OyakeZhengyuan ZhaoKazue IshitsukaZhengyun WuKazuhiko KotaniZhengzhong ZhouKazuhiko KumeZhenhong HeKazuhiro HoriZhenhong WangKazuhiro MiyataZhenhua ZhangKazuhiro P. IzawaZhenhua ZhengKazuhisa KodZheni WangKazuki FukuiZhenjie WangKazuki HiraoZhenmin DingKazuki IdeZhenni ZhuKazuki IwaokaZhenwei LiKazuki KugaZhenxing LiKazuki ShimizuZhenxing ShenKazuki TakasakiZhenxuan ZhangKazuki TokumasuZhenyang ZhangKazuki YokoyamaZhenyi XuKazuma NoguchiZhenyu HanKazushige OshitaZhenyuan WangKazuya TateishiZhenzhen QinKazuyoshi NakanishiZhenzhen ZhengKe FanZheqing ZhangKeane WheelerZhi YangKeaton FletcherZhibin RenKebogile MokwenaZhichao CaoKedar N. PrasadZhichao XueKedong YinZhicheng DuKedra WallaceZhifan LuoKeechilat PavithranZhifei YanKeehoon LeeZhi-Fu WuKeeren Sundara RajooZhigang LyuKees DoevendansZhiguo MengKefa K. OnchokeZhiguo ZhangKei Hang Katie ChanZhihan LiuKei NagashimaZhihao ChenKei NakajimaZhiheng XuKeiichi AkahaneZhihong PangKeiichi KoshinakaZhihong ZhangKeiichi KubotaZhihua TangKeiko NakamuraZhihui WangKeila Acevedo-VillanuevaZhijie WangKeisuke FujiiZhi-Li ZhangKeisuke MurakamiZhiming MaKeisuke NodaZhiming YangKeita HonjoZhiming ZhangKeita WagatsumaZhipeng LuKeith Da SilvaZhiping YuKeith MartinZhiqiang WuKeith RochfortZhiqing ZhouKeith W. PecorZhitao QiKeith WarrinerZhiwu ZhouKejiang LiZhixiong TanKele DingZhixiong ZhouKelei XueZhiying LiuKelli SandersonZhiyuan FengKellia HansmannZhiyuan WangKelly BenderZhiyun JiangKelly Cristina StéfaniZhizhou ZhangKelly De JesusZhizhu YuanKelly E. JohnsonZhongchang SunKelly JohnstoneZhongfeng YeKelly S. HavilandZhonghai LiKelse Tibau De AlbuquerqueZhongju LiaoKelsey HudsonZhongkai WangKelvin Ian AfrashtehfarZhongkang YangKemal Emre ÖzenZhongqiang LiKemal Maulana AlhasaZhongquan GaoKemal SümserZhongwei HuangKemi WrightZhongwei LiuKeming WuZhongzhi ChenKen BataiZhou YuKen Ing Cherng OngZhuanzhuan MaKen KatoZhuen RuanKen LodewykZhujin YangKen MastersZhuolin TaoKen NahZhuonan WangKen SilverZhuoyao FangKen TakasawaZhuoying WangKen TammingaZia Ul MustafaKendall HartleyZia Ur RehmanKendra JonesZiad Al-DwairiKeng Yinn WongZiad D. BaghdadiKeng Yu LinZiad HunaitiKenichi HasegawaZiarno MałgorzataKenichi KobaraZichen JiKenichi KumagaiZidian XieKenichi YamadaŽiga KozincKenichiro SakataZigmantas GudžinskasKenioua MouloudZihan KanKenji HayashidaZihao AnKenneth A. EatonZihe GaoKenneth BlumZiheng ShangguanKenneth BradburyZiho KangKenneth ButlerZijia LiKenneth Edward ChristianZijuan LiuKenneth HandelmanZiliang HuangKenneth IzuoraZilu LiangKenneth Ka-Hei LoZinaida M. KuznetsovaKenneth LandZiqian ZengKenneth ZhouZiqiang HanKennio PaimZishu LiuKennon SheldonZita FerencziKensaku TakaseZiwei MaKentaro KawabeZixian JiaKentaro MatsuiZixin WangKentaro MisakiZiyad AbunadaKentaro OkazakiZiyang LiKentaro TojoZiying YouKentaro YoshiokaZiyue LiKenza E. BenzeroualZizi GoschinKeren Agay-ShayZlatoslav ArabadzhievKeren Cohen-HagaiZoárd Tíbor KrasznaiKeren GrinbergZobia NoreenKerim ÇíçekZoë FritzKerroum DerbalZoe KaranikolaKerry BrooksZofia Koloszko-ChomentowskaKerry L. MarshZofia SzarotaKerry McGawleyZograb MakiyanKerry RedicanZohar EviatarKerry WilsonZoi NikiforidouKerryn Butler-HendersonZoka B. MilanKerstin Persson WayeZoltan BaracskaiKerstin SellZoltán BujdosóKerstin Stieber RogerZoltan KatzKerstin WitteZoltan KekecsKerusha GovenderZoltán TánczosKeshari ThakaliZoltan TorokKeshav BhattaraiZoltan VajoKeshrie NaidooZongbiao HuKeskanya SubbalekhaZongfeng ChenKeum Hwan ParkZongshuan DuanKeumseok KohZoraida Sosa FerreraKeun Ho LeeZoran CicaKeun Hwa LeeZoran MijicKeun Ok AnZoran StevićKeunhye LeeZoran TodorovićKeunje YooZorana LuzaninKeun-Soo ParkZorana MilanovicKevan Guilherme Nóbrega BarbosaZorica Stanojević-RistićKevin B. WrightZornitsa Ivanova ZlatarovaKe-Vin ChangZoubir DjeradaKevin Chien-Chang WuZoya MarinovaKevin DanielsZozan GulekenKevin FonsecaZsofia VerzarKevin GlintonZsolt MurlasitsKevin HallinanZsolt NemethKevin KinserZsolt RadicsKevin M. KellyZsolt SzakályKevin MeestersZsombor LaczaKevin ModestoZsuzsa KalmárKevin NjaboZsuzsanna ElekesKevin P. UribeZsuzsanna KöviKevin PottieZsuzsanna MiklósKevin RaaphorstZsuzsanna MirnicsKevin ReillyZsuzsanna RecsanKevin SchrothZsuzsanna SzaboKevin T. BainZubair Ahmed RatanKevin T. FujiZubair KabirKevin WillyZubeda SheikhKeya SenZubir AzharKeyvan AghababaiyanZufarzaana ZulkefleeKh LotfyZuheir KhlaifKhadim HussainZul’Fiya Sagdullaevna KhodzhaevaKhai Wah KhawZulfaqar Sa’adiKhairuddin IdrisZulfiqarali G. AbbasKhaldoon DhouZülkif TanrıverdiKhaled Abbas El-TarabilyZulkifli Mohd NopiahKhaled Elsayed AhmedZunyao WangKhaled EmaraZuohua HuangKhalfalla AwedatZuojin YuKhalid Abed DahleezZuoxu XieKhalid AlmatarZurab AzmaiparashviliKhalid Al-SheibaniZuyi FangKhalid ElwakeelZuzana BělinováKhalid HashimZuzana HajduováKhalid HussainZuzana HudákováKhalid KarrouchiZuzana LušňákováKhalid KheirallahZuzana MelichováKhalid KoserZuzana ŠkodováKhalid MehmoodZuzana ŠpacírováKhalid Walid BibiZuzana SzabovaKhalid Z. MasoodiZuzana VišňovcováKhalifa Al-KhalifaZuzanna BielanKhalil IbrahimiZuzanna ChrzastekKhalil TamersitZvi LermanKhaliq Ur RehmanZygmunt KowalskiKhashayar KazemzadehZygmunt Kruczek


